# †Kenyaichthyidae fam. nov. and †*Kenyaichthys* gen. nov. – First Record of a Fossil Aplocheiloid Killifish (Teleostei, Cyprinodontiformes)

**DOI:** 10.1371/journal.pone.0123056

**Published:** 2015-04-29

**Authors:** Melanie Altner, Bettina Reichenbacher

**Affiliations:** Department of Earth- and Environmental Sciences, Palaeontology & Geobiology, Ludwig-Maximilians-University, Munich, Germany; NYIT College of Osteopathic Medicine, UNITED STATES

## Abstract

The extant Cyprinodontiformes (killifishes) with their two suborders Cyprinodontoidei and Aplocheiloidei represent a diverse and well-studied group of fishes. However, their fossil record is comparatively sparse and has so far yielded members of the Cyprinodontoidei only. Here we report on cyprinodontiform fossils from the upper Miocene Lukeino Formation in the Tugen Hills of the Central Rift Valley of Kenya, which represent the first fossil record of an aplocheiloid killifish. A total of 169 specimens - mostly extraordinarily well preserved - and a sample of ten extant cyprinodontiform species were studied on the basis of morphometrics, meristics and osteology. A phylogenetic analysis using PAUP was also conducted for the fossils. Both the osteological data and the phylogenetic analysis provide strong evidence for the assignment of the fossils to the Aplocheiloidei, and justify the definition of the new family †Kenyaichthyidae, the new genus †*Kenyaichthys* and the new species †*K*. *kipkechi* sp. nov. The phylogenetic analysis unexpectedly places †*Kenyaichthys* gen. nov. in a sister relationship to the Rivulidae (a purely Neotropical group), a probable explanation might be lack of available synapomorphies for the Rivulidae, Nothobranchiidae and Aplocheilidae. The specimens of †*K*. *kipkechi* sp. nov. show several polymorphic characters and large overlap in meristic traits, which justifies their interpretation as a species flock *in statu nascendi*. Patterns of variation in neural and haemal spine dimensions in the caudal vertebrae of †*Kenyaichthys* gen. nov. and the extant species studied indicate that some previously suggested synapomorphies of the Cyprinodontoidei and Aplocheiloidei need to be revised.

## Introduction

The extant order Cyprinodontiformes contains about 1,120 species [1] and displays a virtually worldwide circumtropical distribution, with the exception of Australia [2]. According to Parenti [3] the order consists of two suborders, the Cyprinodontoidei and the Aplocheiloidei, with a total of ten families. The families of the Cyprinodontoidei include the Cyprinodontidae (United States, Central and South America, the West Indies, Africa, Europe, and Asia), Poeciliidae (United States, Central and South America, and Africa), Fundulidae (United States, Central America, and Canada), Profundulidae (Central America), Anablepidae (southern Mexico to southern South America), Goodeidae (United States), and Valenciidae (Mediterranean region) [4–6]. The families of the Aplocheiloidei can be separated into the Neotropical Rivulidae (South America) and the Old World Nothobranchiidae and Aplocheilidae (Africa, Madagascar, India, and South Asia) [2].

Given the huge diversity of the living Cyprinodontiformes, their fossil record is comparatively poor and is so far restricted to the Cyprinodontoidei. The highest species diversity is known for the extinct †*Prolebias* SAUVAGE, 1874, from the Oligocene and Miocene of Europe and Asia, which has recently been identified as a paraphylum and now includes several additional genera [1, 7]. Another extinct genus known from the Miocene of Europe is †*Aphanolebias* Reichenbacher and Gaudant, 2003 [8]. In addition, a few fossil species of the extant genus *Aphanius* NARDO, 1827 have been reported from the Miocene and Pliocene of Europe and Asia [9–15]. Furthermore, a single fossil species of *Cyprinodon* LACÉPÈDE, 1803 from the late Pliocene and several species of *Fundulus* LACÉPÈDE, 1803 from the middle Miocene to early Pleistocene have been reported from the United States (see review by [16, 17]). Fossil species of the Anablepidae such as †*Carrionellus* WHITE, 1927 from the early Miocene [18] and *Jenynsia* GÜNTHER, 1866 from the late middle Pleistocene [19] have been reported from South America [20], whereas *Empetrichthys* GILBERT, 1893 has been reported from the Pliocene of the United States [21]. Additionally, several fossil taxa of the Goodeidae such as *Alloophorus*, *Goodea*, *Chapalichthys*, *Ameca*, *Girardinichthys*, *Xenotoca*, and †*Tapatia* have been reported from the late Miocene to late Pliocene of North America (see review by [22, 23]). Undetermined species of the Poeciliidae have been described from the Eocene Lumbrera Formation [20, 24–26] and the Miocene San José and Rio Salí Formations of Argentina [20, 24, 27], and *Poeciliopsis* is known from the Pleistocene of Mexico [23]. In contrast to this comparatively rich record of Cyprinodontoidei, no fossil species of the Aplocheiloidei have yet been described.

Most authors argue that the origin of the Cyprinodontiformes dates to the Cretaceous at least (see [28]). Some authors assume that they had an ancient Gondwana-wide distribution and that their present-day distribution is linked to the break-up of Gondwana (vicariance hypothesis) [6, 29, 30]. Others argue that the Cyprinodontiformes originated in South America and that their radiation is linked to dispersal in the middle or late Cretaceous [28, 31]. However, previously reported records of Cyprinodontiformes from strata older than Oligocene are scarce and some of them are now regarded as doubtful. Thus the identification of fossil specimens from the upper Cretaceous Molino Formation of Bolivia, South America as cf. Cyprinodontiformes [32, 33] is not supported in Gayet and Meunier [34]. Fossil scales of a putative species of †*Cyprinodon* (*C*. (?) *primulus*) from the upper Paleocene to lower Eocene Maíz Gordo Formation of Argentina described by Cockerell [35] have been re-interpreted as Cyprinodontiformes indet [20, 24] or Poeciliidae indet [36]. However, these scales do appear to represent the first secure fossil record of Cyprinodontiformes, indicating that the order is of late Paleocene (56–59 Ma) age at least.

The objective of this study is to describe newly discovered fossils of killifishes from the upper Miocene Lukeino Formation in Kenya. †Kenyaichthyidae nov. fam., †*Kenyaichthys* nov. gen., and †*K*. *kipkechi* sp. nov. are introduced. †*Kenyaichthys* is the first fossil record of the Aplocheiloidei.

### Stratigraphic and sedimentological context

#### Study area

**Fig 1:**
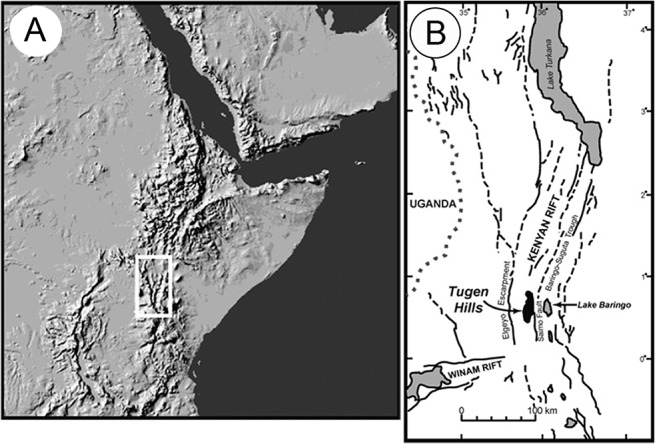
Geographic overview of the East African Rift System (EARS) and the study site (Reprinted from [37] with permission from Elsevier). **A** Map of East Africa with the location of the EARS; **B** Detail of the EARS with the location of the Tugen Hills.

The study area is located in the Tugen Hills in the Central Rift Valley of Kenya (Fig 1A–1B). One of the most complete Neogene successions in Africa is found here, with exposures consisting of sedimentary strata that alternate with volcanic rocks of middle Miocene to Pleistocene age [38]. The fish-bearing diatomaceous shales belong to the Lukeino Formation, which is about 110 m thick and of late Miocene (5.7–6 Ma) age (see [39, 40]). Among the fossils previously described from the Lukeino Formation are freshwater faunal elements (gastropods, bivalves, crocodiles, hippos and turtles) and terrestrial remains such as dicotyledonous leaves [41], as well as one of the earliest hominids known [38, 42, 43].

The fossils described here come from Inoswa Kamelon (0°45'43.71"N 35°50'7.98"E; locality 2/215N in [38]) near “a small hillside east of the Bartabwa-Yatya road” ([38] page 77) and from Koibochepkweny (00°50'10.6"N 35°54'29.2"E; locality 2/222 in [38]) about 3 km north of Inoswa Kamelon, “east of the Yatya-Bartabwa road” ([38] page 80). All fossils were collected by a research team led by M. Pickford and B. Senut (both Muséum National d´Histoire Naturelle Paris) in 2004–2005, in collaboration with the members of the local Orrorin-Community-Organisation.

## Materials and Methods

### Fossil Material

Inoswa Kamelon yielded 164 fish specimens, almost half of which (72) are complete. Koibochepkweny yielded five complete specimens. All fossils have been deposited in the Museum in Kipsaraman, Kenya, which is affiliated with the National Museum in Nairobi. Fossils are labelled with the prefix BAR (for Baringo) and the following numbers: 1141´04–1237´04, 1324´04, 1325´04, 1192a/b´05, 1203a/b´05, 1204´05, 1209a/b´05, and 1218a/b´05. Silicone casts of 49 selected specimens are kept in the Bavarian State Collection for Palaeontology and Geology, Munich, Germany (BSPG) under the numbers BSPG 2013 XXV 1–49. All extinct taxa are indicated with †.

### Comparative Material Examined

Suborder Cyprinodontoidei, Family Cyprinodontidae:

- *Aphanius sophiae* (Heckel, 1847), Zoological Museum of Shiraz University, Collection of Biology Department, Shiraz, Iran (ZM-CBSU) 281, 283, 284, 6171, 6193, 8296, 8401, 10883, 10884, 10962, C227, C295, C316, Zoological Museum of Shiraz University, Collection of Biology Department, Shiraz, Iran (ZM-CBSUZG) 177, 178, 183–185, 188 (17 cleared and stained specimens (c&s) and two x-rayed specimens (xr) from the Kor Basin, SW Iran; see [44]),
- *Aphanius farsicus* (Teimori, Esmaeili, Reichenbacher, 2011), ZM-CBSUZG 1, 8, 13, 140, 141, 142 (six xr from the Marharlu Basin, SW Iran; see [44]),
- *Aphanius arakensis* (Teimori, Esmaeili, Gholami, Zarei, Reichenbacher, 2012), ZM-CBSUZG 350, 352, 354, 356, 359, 361 (two c&s and four xr from the Namek Basin, SW Iran; see [44]),
- *Aphanius mesopotamicus* (Coad, 2009), ZM-CBSUZG 362, 363, 364, 365 (four c&s from the Karun Basin, SW Iran; see [44]).

Suborder Aplocheiloidei, Family Aplocheilidae:

- *Pachypanchax playfairii* (GÜNTHER, 1866), Musée Royal de l´Afrique Centrale, Tervuren, Belgium (MRAC) P.188937-188938 (two c&s from Les Canelles, Mahé Sud, Seychelles).

Suborder Aplocheiloidei, Family Nothobranchiidae:

- *Aphyosemion castaneum* Myers, 1924, MRAC 91-080-P-0063-0064 (two c&s from the Masendula River, Haut-Zaire, Zaire);
- *Epiplatys sexfasciatus* Gill, 1862, MRAC 92-052-P-0512-0513 (two c&s from a side channel of the Sombreiro River at the new Ahoada bridge, Nigeria);
- *Foerschichthys flavipinnis* (Meinken, 1932), MRAC 91-001-P-0378-0379 (two c&s from Taylor Creek, Biseni, Niger Delta, Nigeria);
- *Fundulopanchax sjoestedti* (Lönnberg, 1895), MRAC 91-100-P-0050-0051 (two c&s from drying swampforest waters 2–3 km east of Kaiama, on East-West road near the turn-off to Kalama village, Nigeria);
- *Nothobranchius orthonotus* (Peters, 1844), MRAC A4-039-P-0133-0134 (two c&s from a site on the road from Nicoladala to Caia Ferry, Mozambique).

### Methods

Obscuring sediment matrix was removed from fossil specimens under a stereomicroscope, using dissecting needles, and peels based on 49 selected specimens were produced by applying dyed silicone in thin coats. Extant specimens (see Comparative Material) were cleared and stained for cartilage and bone following the protocol of Taylor and Van Dyke [45].

**Fig 2:**
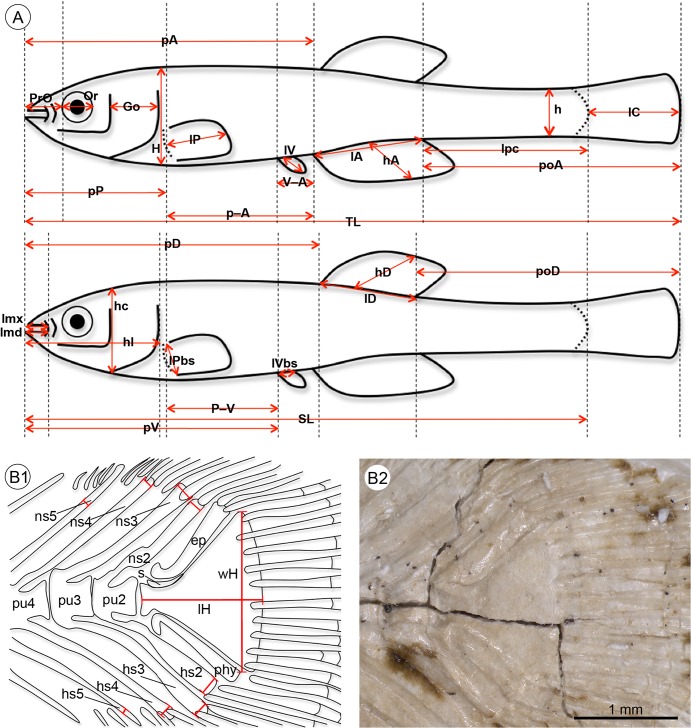
Schematic drawings indicating measurements used in this study. **A**, morphometric parameters; **B1–B2** measurements of hypural plate length and width and measurements of the spine widths on the caudal skeleton of †*K*. *kipkechi* sp. nov. (paratype 1200´04); note that the proximal part of the parhypural (with an anteroventral projection) does not overlap the terminal centrum, left lateral view. Abbreviations: ep, epural; Go, gill opening; H, maximum body depth; h, minimum body depth; hA, depth of anal fin; hc, head depth; hD, depth of dorsal fin; hs2–5, haemal spine of preural vertebrae 2–5; lA, length of anal fin base; lc, length of head; lC, length of caudal fin; lD, length of dorsal fin base; lmd, lower jaw length, i.e. distance from anteriormost point of lower jaw symphysis to posteriormost margin of mandibular joint; lH, length of hypural plate; lmx, upper jaw length, i.e. distance between anteriormost point of premaxillary and posteriormost point of maxillary; lP, length of pectoral fin; lPbs, length of pectoral fin base; lpc, length of caudal peduncle; lV, length of pelvic fin; lVbs, length of pelvic fin base; ns2–5, neural spine of preural vertebrae 2–5; Or, eye diameter; pA, preanal distance; P–A, distance between pectoral fin base and anal fin base; pD, predorsal distance; phy, parhypural; poA, postanal distance, i.e. from posterior end of anal fin to end of caudal fin rays; poD, postdorsal distance, i.e. from posterior end of dorsal fin to end of caudal fin rays; pP, prepectoral distance; prO, preorbital distance; pu2–4, preural vertebrae 2–4; pV, prepelvic distance; P–V, distance between pectoral fin base and pelvic fin base; s, stegural; SL, standard length; TL, total length; V–A, distance between pelvic fin base and anal fin base; wH, width of hypural plate.

Osteological, meristic and morphometric characters of the fossil and extant specimens were studied under a stereomicroscope equipped with a digital camera. The standard length (SL) and total length (TL) were measured based on digital images using ImageJ version 1.49a [46] and recorded to the nearest 0.1 mm. All other measurements were recorded to the nearest 0.01 mm. Morphometric and meristic characters follow Holcik [47] (Fig 2A), apart from dorsal and anal fin ray counts, which included every detectable ray, whether supported by a proximal radial (pterygiophore) or not. In the case of individuals that were represented by part and counterpart, both parts were considered in the character analysis, while only one value (the maximal value) of the respective measurement or count was included in the statistical analyses. All measurements were standardized based on the standard length. Data from the literature were taken into account in the interpretation of osteological characters [1, 3, 48–50].

The widths of the spines of the preural vertebrae (PU) PU2–PU5 were measured and, as spine ratios are considered to be important for phylogenetic analysis within Cyprinodontiformes, ratios for PU2/PU4, PU2/PU5, and PU3/PU5 were calculated based on both neural and haemal spines (see S4 and S8 Tables). Measurements of spines attached to PU2, PU3, and PU4 were based on the width of the most distal part of the respective neural or haemal spine (Fig 2B1 and S1 Fig); in the case that the distal tip of a spine was obscured by caudal fin rays, its width was measured just before these rays. Measurements of spines attached to PU5 were based on the maximal width in the distal third of the respective neural or haemal spine (calculation of the distal third was based on the entire spine length including the arch). If spines were split or duplicated, we measured the broader of the two spines. For comparison with extant material, we determined PU2/PU4 and PU2/PU5 haemal spine ratios of ≥1.0 and <2.0 as “slightly wider” and ratios of ≥2.0 as “distinctively wider”.

Phylogenetic reconstructions were performed using PAUP version 4.0b10 [51], characters with unknown state were coded as question marks; all character states were treated as unordered and unweighted. Selection of outgroups (Atherinomorpha and Beloniformes) followed Costa [48]. Statistical analyses were performed using SPSS version 21.0 [52]. All necessary permits were obtained for the described study, which complied with all relevant regulations. The research clearance permit was obtained from the National Council for Science and Technology.

## Results

### Systematic Palaeontology

Order Cyprinodontiformes Berg, 1940

Suborder Aplocheiloidei Parenti, 1981

Family **†**Kenyaichthyidae fam. nov.

### Type Genus. †*Kenyaichthys*, gen. nov.

#### Diagnosis

Differs from other known families of the Aplocheiloidei in the combination of the following characters: first vertebra with distinctive neural spine vs. neural spine of first vertebra absent in Aplocheilidae; first vertebra with two long and narrow neuroapophyses of equal length and width lateral to the narrow neural spine vs. first vertebra with two short lateral neuroapophyses and broad neural spine in some Rivulidae and all Nothobranchiidae vs. first vertebra with distinctive neural spine and neuroapophyses absent in some Rivulidae vs. first vertebra with median neural spine and neuroapophyses absent in remaining Rivulidae; pelvic girdle with laterally pointed process vs. no laterally pointed process in those Rivulidae, in which this character has been examined; five or six preural vertebrae vs. four or five preural vertebrae in all Rivulidae, Nothobranchiidae and Aplocheilidae; rod-shaped epipleural ribs vs. bifid epipleural ribs in Nothobranchiidae and some Rivulidae; long ventral portion of autopalatinum reaching the quadratum vs. short autopalatinum not reaching quadratum in Rivulidae; robust, L-shaped preoperculum vs. thin, C-shaped preoperculum in Rivulidae; lateral rim of frontals not reduced vs. lateral rim of frontals reduced in Rivulidae; posterior tip of the ascending process of the premaxilla not medially curved vs. posterior tip of the ascending process of the premaxilla medially curved in Aplocheilidae and Nothobranchiidae.

### †*Kenyaichthys* gen. nov.

(Figs 3–12)

**Fig 3:**
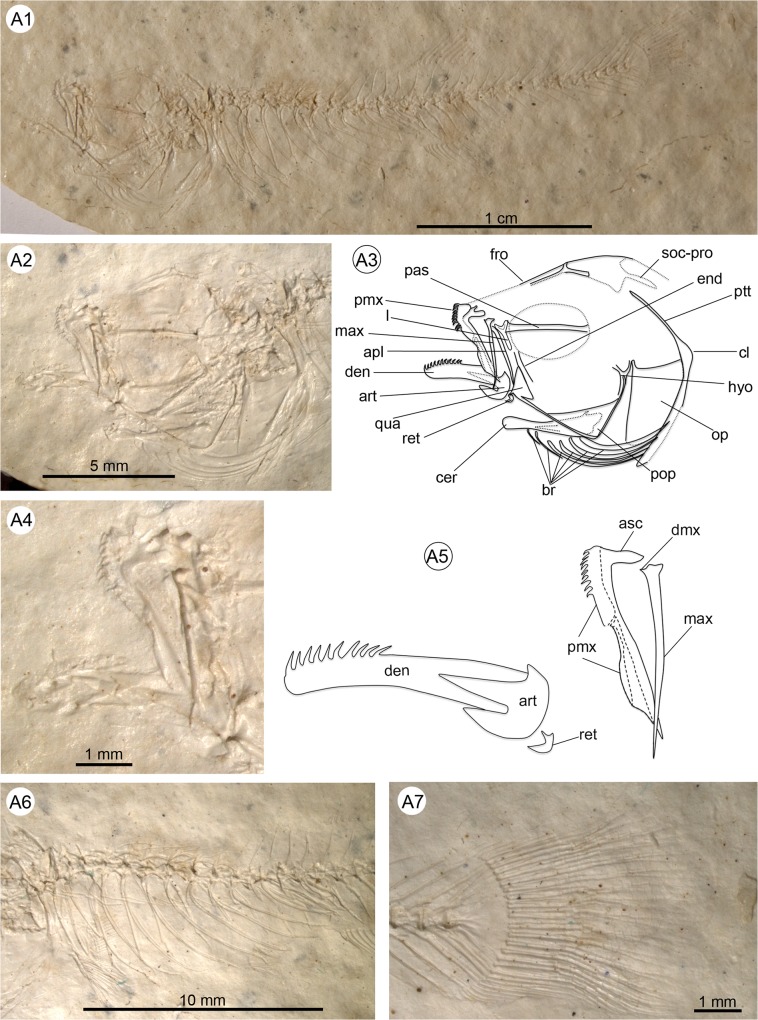
Anatomical details of †*Kenyaichthys* nov. gen. (holotype 1209a´05, †*K*. *kipkechi*), left lateral view. **A1** General overview (photograph by Dr. W. Altner); **A2–A3** Close up of the head and pectoral girdle (the lacrimal is from the counterpart and mirrored for clarity); **A4–A5** Close-up of lower and upper jaw; **A6** Detail of abdominal part showing vertebrae, pleural ribs and epipleural ribs; **A7** Truncate to rounded caudal fin. Abbreviations: apl, autopalatinum; art, anguloarticular; asc, premaxillary ascending process; br, branchiostegal rays; cer, ceratohyal; cl, cleithrum; den, dentary; dmx, dorsal maxillary process; end, endopterygoid; fro, frontal; hyo, hyomandibula; l, lacrimal; max, maxilla; op, operculum; pas, parasphenoid; pmx, premaxilla; pop, preoperculum; ptt, posttemporal; qua, quadratum; ret, retroarticular; soc-pro, supraoccipital process.

**Fig 4:**
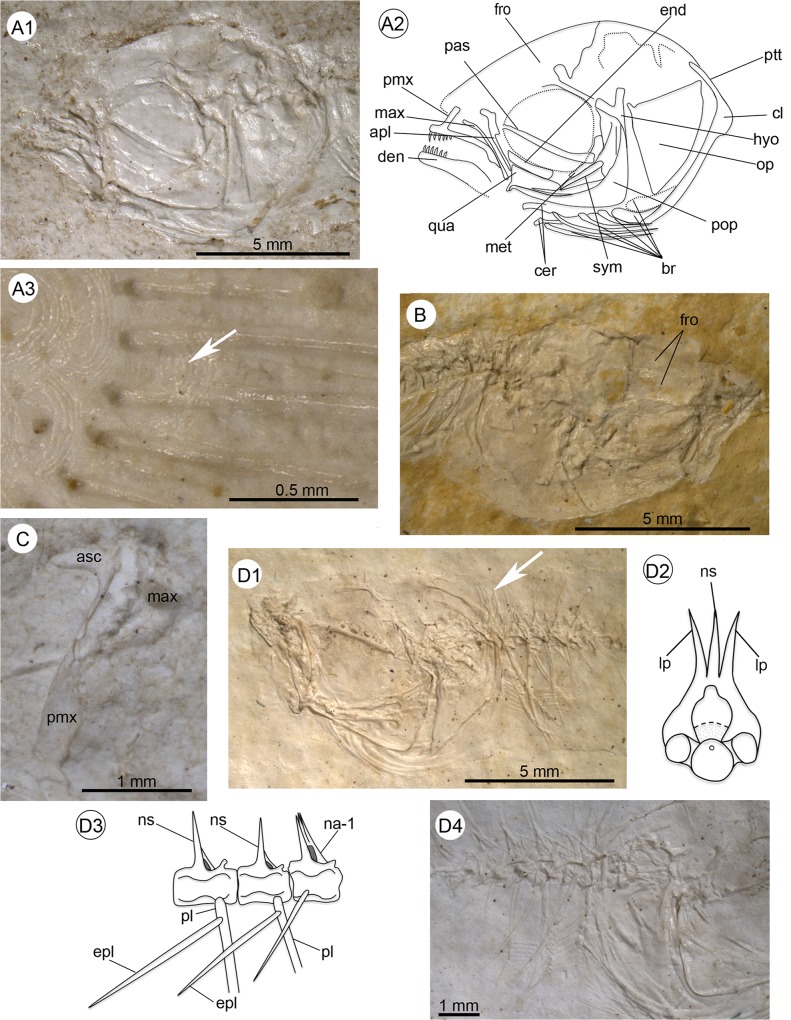
Anatomical details of †*Kenyaichthys*. **A1–A3** Anatomical details of **†** *K*. *kipkechi* sp. nov. (paratype 1237R(1)´04): **A1–A2** Head and pectoral girdle, left lateral view; **A3** Caudal fin rays covered with a single scale (arrow), left lateral view; **B** Head of **†** *K*. *kipkechi* sp. nov. (paratype 1160b´04), right dorsolateral view; **C** Disarticulated premaxilla and maxilla of **†** *Kenyaichthys* cf. *kipkechi* (1226a(1)´04), right lateral view; **D1–D4** Anatomical details of **†** *K*. *kipkechi* (paratype 1209a/b´05): **D1** Head and anterior part of body (1192a´05), arrow indicates lateral processes and spine of the first vertebra, left lateral view; **D2** Schematic reconstruction of the first vertebra (paratype 1192a/b´05), anterior view; **D3** Reconstruction of vertebrae 1–3 with pleural ribs and rod-shaped epipleural ribs (1192b´05), left lateral view; **D4** Head and anterior part of body (1192b´05) with epipleural ribs on vertebrae 1–5, left lateral view. Abbreviations: apl, autopalatinum; br, branchiostegal rays; cer, ceratohyal; cl, cleithrum; den, dentary; end, endopterygoid; epl, epipleural rib; fro, frontal; hyo, hyomandibula; lp, lateral process; max, maxilla; met, metapterygoid; na-1, neural arch of first vertebra; ns, neural spine; op, operculum; pas, parasphenoid; pl, pleural rib; pmx, premaxilla; pop, preoperculum; ptt, posttemporal; qua, quadratum; sym, symplectic.

**Fig 5:**
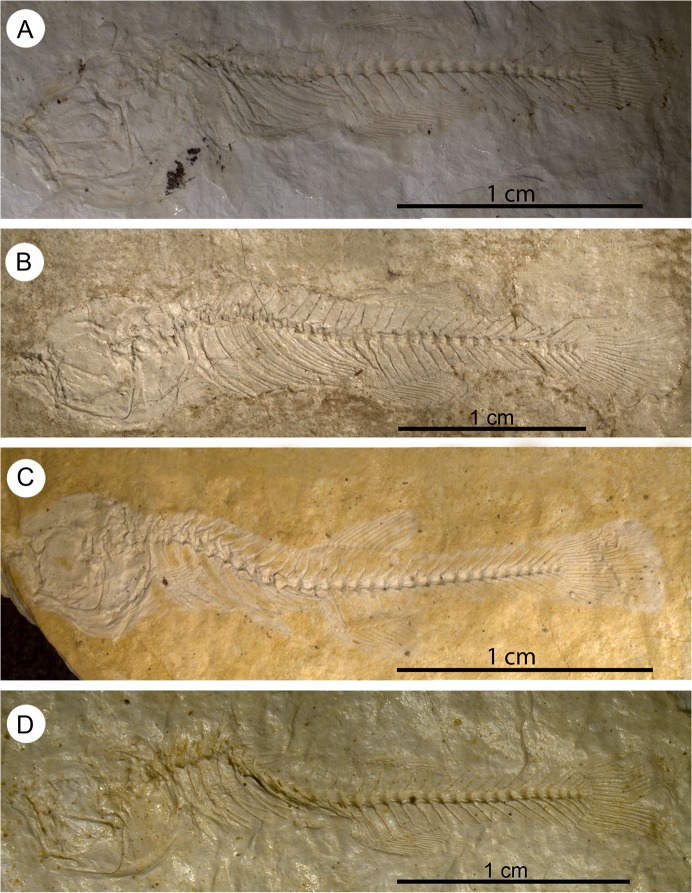
General view of four specimens of †*K*. *kipkechi* sp. nov. showing the varying extent of curvature of the vertebral column. **A**, straight (paratype 1146´04, mirrored); **B**, almost straight (paratype 1228(1)´04, mirrored); **C**, strongly curved (paratype 1168´04); **D**, strongly curved (paratype 1206(1)´04).

**Fig 6:**
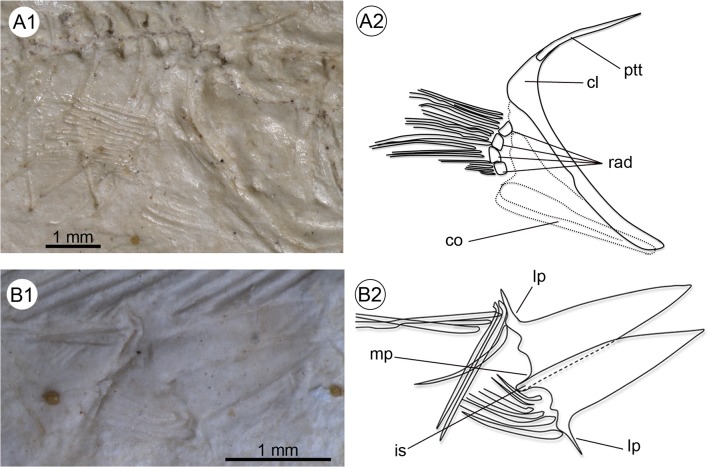
Details of the pectoral and pelvic girdles and fins seen in †*Kenyaichthys kipkechi* sp. nov., right lateral views. **A1–A2** Pectoral girdle and fin (paratype 1192´04); **B1–B2** Pelvic girdle and fin (paratype 1218a´05). Abbreviations: cl, cleithrum; co, coracoid; is, ischial process; lp, lateral process; mp, medial process; ptt, posttemporal; rad, pectoral radials.

**Fig 7:**
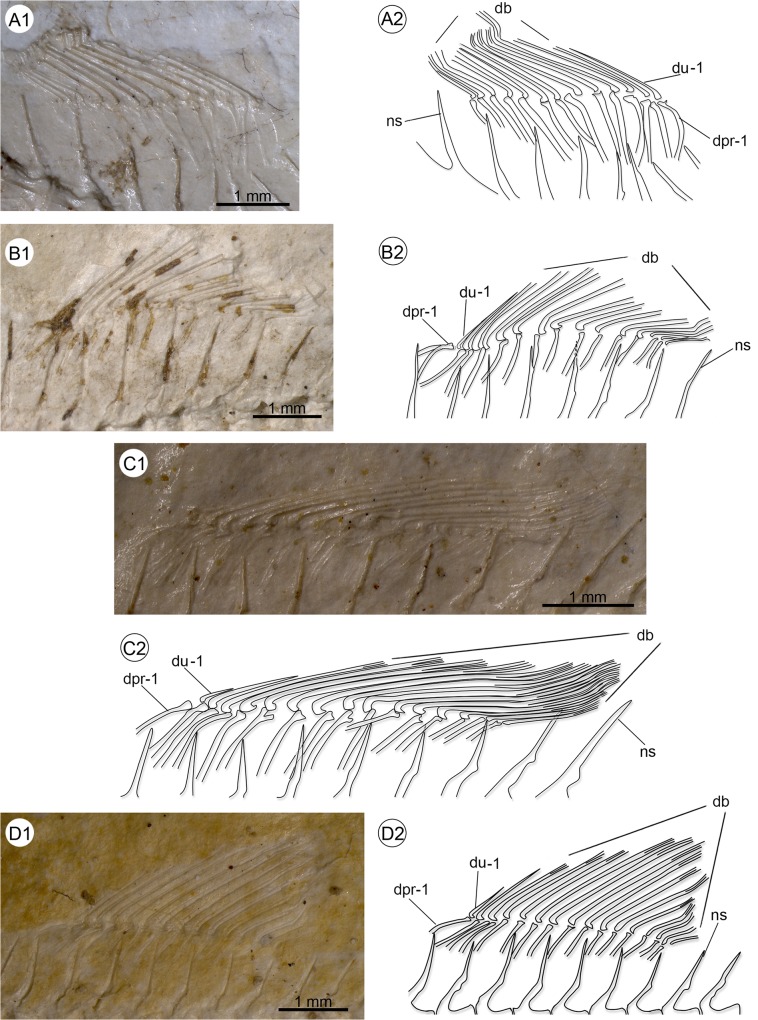
Polymorphism in the arrangement of the proximal radials of the dorsal fin seen in †*Kenyaichthys* nov. gen. **A1–A2** †*Kenyaichthys kipkechi* sp. nov. (paratype 1154a´04): dorsal fin with a single short ray and 12 long rays; a single proximal radial supports the first short ray, two proximal radials support the first long ray, and one proximal radial supports all remaining rays, right lateral view; **B1–B2** †*K*. *kipkechi* sp. nov. (paratype 1152´04): dorsal fin with two short rays and 11 long rays; two proximal radials support the first short ray and one proximal radial supports all other rays with the exception of the last ray, left lateral view; **C1–C2** †*K*. *kipkechi* sp. nov. (paratype 1206(1)´04): dorsal fin with two short rays and 13 long rays; two proximal radials support the second short ray and one proximal radial supports all other rays, left lateral view; **D1–D2** †*K*. *kipkechi* sp. nov. (paratype 1168´04): dorsal fin with two short rays and 14 long rays; two proximal radials support the first long ray and one proximal radial supports all other rays with the exception of the last ray, left lateral view. Abbreviations: db, branched rays of dorsal fin; dpr-1, first dorsal proximal radial; du-1, first unbranched dorsal fin ray; ns, neural spine.

**Fig 8:**
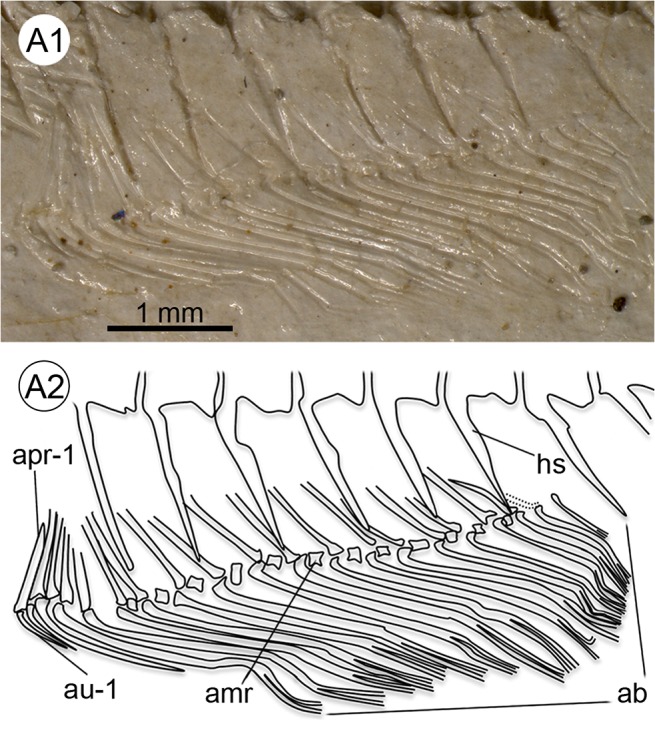
Details of the anal fin of †*Kenyaichthys kipkechi* sp. nov. (paratype 1177´04). **A1–A2** Anal fin with three short rays, 17 long rays and 19 proximal radials (last ray not supported by proximal radial), left lateral view. Abbreviations: ab, branched rays of anal fin; amr, anal medial radial; apr-1, first anal proximal radial; au-1, first unbranched anal fin ray; hs, haemal spine.

**Fig 9:**
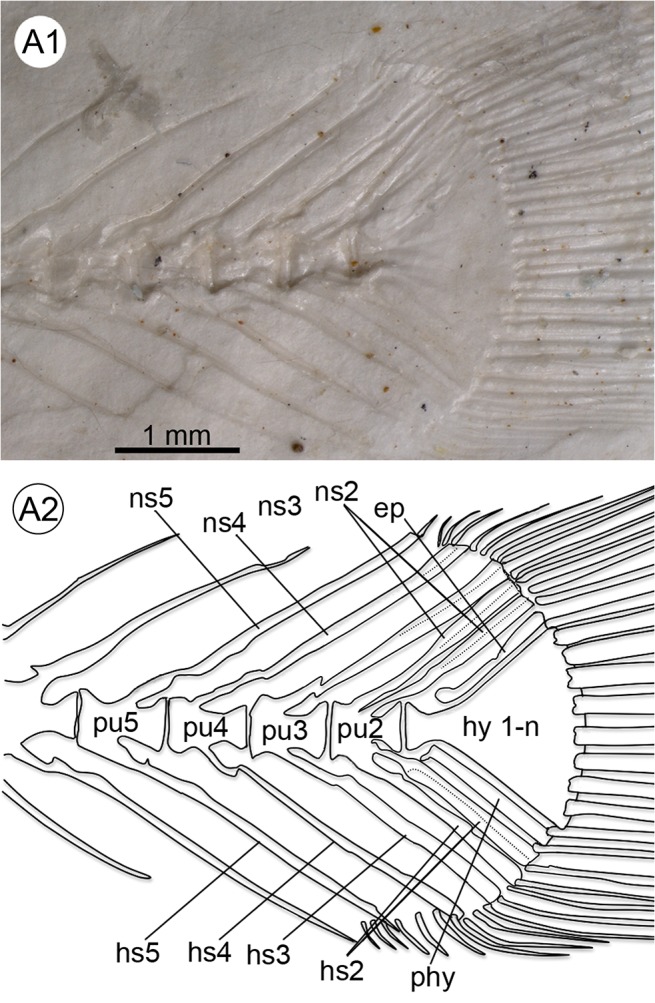
Details of the caudal skeleton of †*K*. *kipkechi* sp. nov. (holotype 1209a´05). **A1–A2** Caudal fin with fused hypural plates, one parhypural, one epural, five preural vertebrae (pu2–5) and duplicated spines of PU2, left lateral view. Abbreviations: ep, epural; hs2–5, haemal spine of preural vertebrae 2–5; hy 1–n, hypural plates 1–n; ns2–5, neural spine of preural vertebrae 2–5; phy, parhypural.

**Fig 10:**
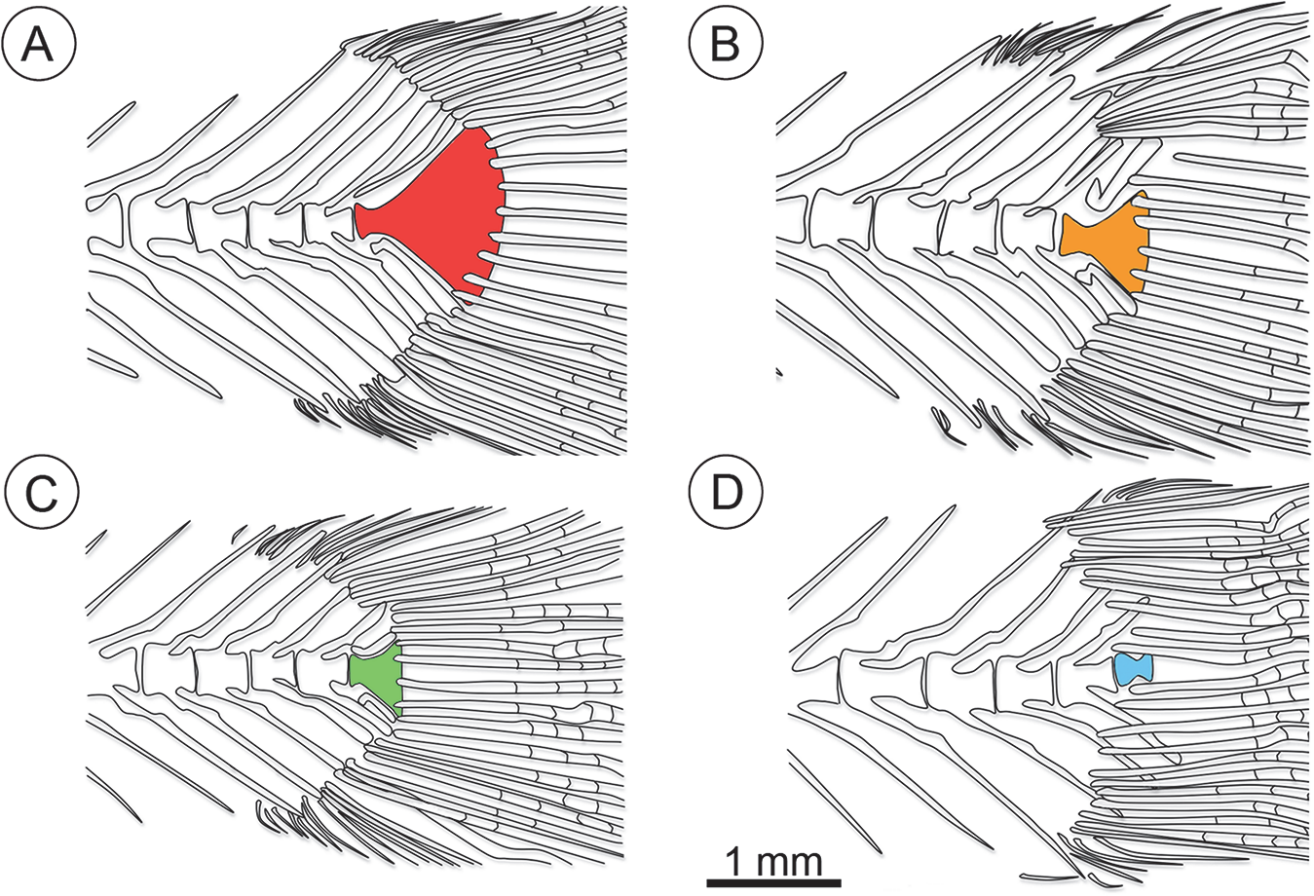
Reconstructions of the caudal skeletons of four specimens of †*K*. *kipkechi* sp. nov. showing the polymorphism in the hypural plate dimensions. **A**, paratype 1237R(1)´04; **B**, paratype 1206(1)´04; **C**, paratype 1168´04; **D**, paratype 1146´04 (mirrored).

**Fig 11:**
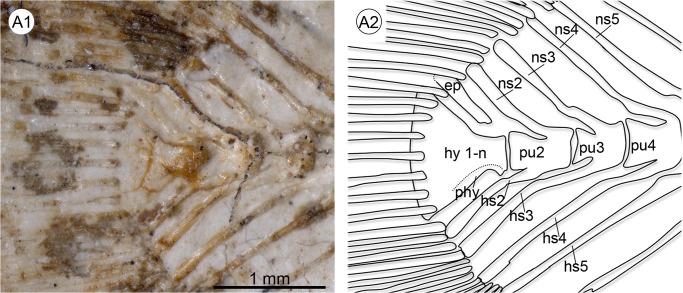
Details of the caudal skeleton of †*K*. *kipkechi* sp. nov. (paratype 1220´04). **A1–2** Caudal fin with overlap between the proximal part of the parhypural, the terminal centrum and the hypural plate, right lateral view. Abbreviations: ep, epural; hs2–5, haemal spine of preural vertebrae 2–5; hy 1–n, hypural plates 1–n; ns2–5, neural spine of preural vertebrae 2–5; phy, parhypural; pu2–4, preural vertebrae 2–4.

**Fig 12:**
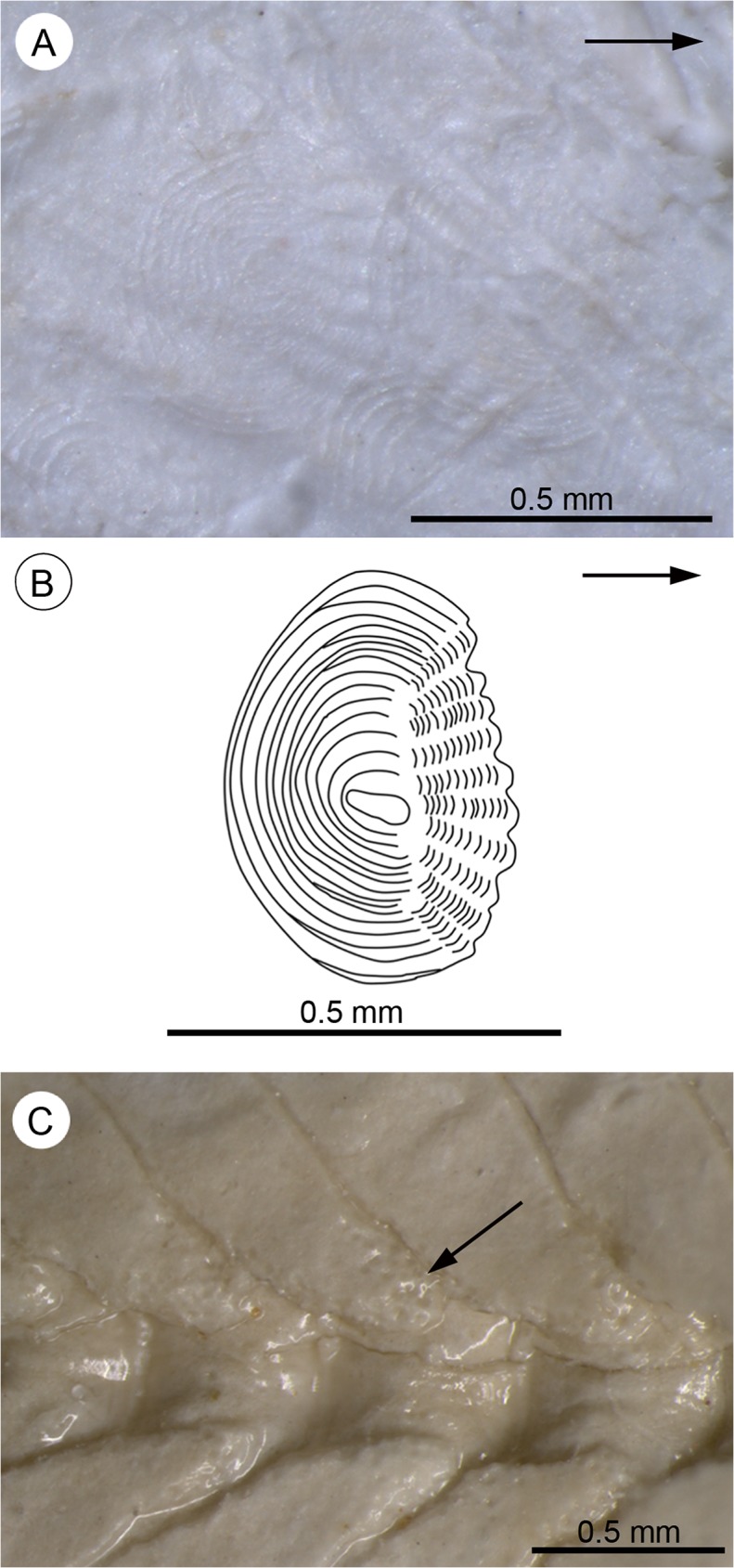
Details of squamation and granulation structures seen in †*Kenyaichthys* nov. gen. **A** Cycloid scales on the pectoral girdle of †*Kenyaichthys* cf. *kipkechi* (1223R´04), right lateral view (arrow points anteriorly); **B** Reconstruction of a cycloid scale on the operculum (based on paratype of †*K*. *kipkechi* sp. nov., specimen 1237R(1)´04), mirrored for better comparison (arrow points anteriorly). **C** Granulation (arrow) on neural spine of caudal vertebra no. 10 (based on †*K*. *kipkechi* sp. nov., paratype 1147´04), right lateral view.

### Type and only known species. †*Kenyaichthys kipkechi* sp. nov.

#### Etymology

Named for the country in which the specimens were found (Kenya), and *ichthys* (Greek) used to refer to fishes; gender feminine

#### Diagnosis

As for the family.

### †*Kenyaichthys kipkechi* sp. nov.

(Figs 3–12)

#### Holotype

1209a/b´05.

#### Referred Specimens

77 specimens, designated by the prefix BAR and the following numbers [(1)–(2) indicate individual specimens when more than one specimen is preserved on the same slab, and R indicates specimens on the rear side of the slab; “/” indicates presence of part and counterpart]: 1142´04, 1144/1146´04, 1145´04, 1147´04, 1148(1)´04, 1149´04, 1150´04, 1151/1152´04, 1153´04, 1154a/b´04, 1155´04, 1156´04, 1157(1)/1158(1)´04, 1159a(1)/b(1)´04, 1159a(2)/b(2)´04, 1160a/1161b´04, 1160b/1161a´04, 1162´04, 1163a(1)/b(2)´04, 1163a(2)/b(1)´04, 1164a/b´04, 1165a/b´04, 1166a´04, 1166b´04, 1167´04, 1168´04, 1170´04, 1171´04, 1172´04,1174´04, 1175´04, 1176a/b´04, 1177´04, 1178(1)´04, 1180(1)´04, 1181(1)´04, 1181(2)/1183(1)´04, 1182´04, 1184(1)´04, 1185/1186´04, 1187´04–1189´04, 1190´04, 1192a/b´05, 1192´04, 1193´04, 1194´04, 1198a/b´04, 1199a/b´04, 1200´04, 1202´04, 1203a/b´05, 1204´05, 1204´04, 1206(1)/1211´04, 1209´04, 1213(1)´04, 1215(1)´04, 1217a(1)/b(1)´04, 1218´04, 1218a/b´05,1219(1)´04, 1220(1)´04, 1220R´04, 1221(1)´04, 1227(1)´04, 1228(1)/1237R(1)´04, 1233/1234(1)´04, 1234(2)´04, 1234R´04, 1236(1)´04, 1237(1)´04, 1237(2)´04, 1324´04, 1325´04. In addition, 92 further specimens are tentatively assigned as †*K*. cf. *kipkechi* because of incomplete or fragmentary preservation (see S3 Table).

#### Age

Late Miocene, about 5.7–6 Ma.

#### Locality

Tugen Hills, Inoswa Kamelon (72 specimens) and Koibochepkweny (5 specimens), Lukeino Formation, Kenya.

#### Etymology

Named for Joseph Kipkech, Kenya, in recognition of his long-time devoted commitment to the development of education and science in Baringo County.

#### Diagnosis

As for the family.

#### Description

Small fishes, ranging in size from 22 to 40 mm total length (TL), and 20 to 36 mm standard length (SL) (see Table 1 and S1 Table for measurements). Most specimens are preserved in lateral view, indicating that the body is elongate and laterally compressed. Body height reaches a maximum between the posterior margin of the head and the origin of the pectoral fin, and ranges from 16–28% of SL. The minimum body height, ranging from 6–12% SL, lies at the middle of the caudal peduncle. Head length ranges from 25–34% SL; the lateral profile from the snout to the occiput is asymmetrical, with a weakly rising long anterior part, and a curving short posterior part (Fig 3). The snout is slightly pointed, with the lower jaw slightly longer than the upper (superior mouth) (see Table 1, S1 Table and Fig 3). The caudal peduncle is long and elongate (22–30% SL), and reveals a slightly concave ventral and dorsal profile (Fig 3A1). The caudal fin ranges in size from 8 to 17% SL and is rounded to truncate (Fig 3A7). Cycloid scales are visible on the whole body and parts of the head (operculum, preoperculum, frontals), but not on the fins, with the exception of a few scales on the caudal fin base (Fig 4A3).

**Table 1:** Morphometric characters (given in mm and in % of SL) for †*Kenyaichthys kipkechi* sp. nov. based on the holotype (1209a/b´04) and all other specimens.

**Characters**	**Holotype**		**All other specimens (mean values ± SD and ranges)**	
**Morphometrics**	mm	% SL	mm	% SL
**Total length (73)**	39.70	111.20	30.09 ± 3.20 (22.10–38.50)	112.50 ± 2.02 (107.09–115.91)
**Standard length (77)**	35.70	–	26.83 ± 2.80 (20.40–33.40)	–
**Ratio of head length to head depth (68)**	1.45	–	1.53 ± 0.15 (1.27–1.97)	–
**Length of head (76)**	10.46	29.30	7.92 ± 1.05 (5.73–10.85)	29.61 ± 2.28 (24.73–33.94)
**Head depth (68)**	7.19	20.14	5.22 ± 0.80 (3.60–7.05)	19.52 ± 2.38 (15.16–24.41)
**Eye diameter (72)**	2.78	7.79	2.32 ± 0.38 (1.41–3.26)	8.65 ± 1.09 (5.72–12.21)
**Gill opening (74)**	2.73	7.65	2.29 ± 0.35 (1.36–3.34)	8.56 ± 0.98 (6.05–10.31)
**Preorbital distance (72)**	3.53	9.89	2.35 ± 0.55 (1.07–3.89)	8.50 ± 1.67 (4.04–12.44)
**Length of dorsal fin base (70)**	3.93	11.01	3.51 ± 0.55 (2.49–4.89)	13.15 ± 1.52 (9.83–16.16)
**Depth of dorsal fin (58)**	–	–	2.67 ± 0.51 (1.25–3.63)	10.02 ± 1.65 (6.13–14.83)
**Length of anal fin base (73)**	4.92	13.78	4.01 ± 0.60 (1.91–5.65)	14.97 ± 1.82 (9.05–19.49)
**Depth of anal fin (62)**	–	–	2.52 ± 0.48 (1.13–3.33)	9.38 ± 1.55 (5.42–12.87)
**Length of pectoral fin (60)**	2.52	7.06	2.45 ± 0.57 (1.09–3.70)	9.04 ± 1.92 (4.61–13.31)
**Length of pectoral fin base (67)**	1.48	4.15	1.15 ± 0.29 (0.56–1.97)	4.29 ± 0.94 (2.17–7.16)
**Length of pelvic fin (65)**	1.13	3.17	1.26 ± 0.30 (0.48–1.92)	4.69 ± 1.00 (2.35–6.96)
**Length of pelvic fin base (66)**	0.24	0.67	0.25 ± 0.06 (0.13–0.44)	0.93 ± 0.24 (0.48–1.72)
**Length of caudal fin (71)**	3.94	11.04	3.59 ± 0.49 (1.99–4.71)	13.50 ± 1.63 (8.21–16.77)
**Minimum body depth (72)**	2.51	7.03	2.30 ± 0.38 (1.62–3.25)	8.60 ± 1.12 (6.41–11.82)
**Maximum body depth (61)**	7.35	20.59	5.56 ± 0.84 (3.80–7.67)	20.60 ± 2.54 (16.42–27.89)
**Predorsal distance (75)**	21.68	60.73	15.63 ± 1.70 (11.43–19.38)	58.38 ± 2.12 (50.04–65.05)
**Preanal distance (77)**	22.07	61.82	15.99 ± 1.96 (11.54–20.81)	59.52 ± 2.53 (52.91–66.05)
**Postdorsal distance (66)**	13.29	37.23	11.03 ± 1.25 (7.87–13.76)	41.56 ± 2.75 (34.56–49.80)
**Postanal distance (67)**	12.65	35.43	10.47 ± 1.21 (7.49–13.62)	39.32 ± 3.18 (31.15–53.83)
**Length of caudal peduncle (74)**	9.09	25.46	6.98 ± 0.75 (5.33–8.69)	26.10 ± 1.60 (21.51–29.72)
**Distance between Pectoral-Anal fins (72)**	10.34	28.96	7.06 ± 0.93 (5.15–9.74)	26.24 ± 2.40 (20.36–31.92)
**Distance between Pelvic-Anal fins (70)**	3.93	11.01	2.30 ± 0.46 (1.18–3.26)	8.59 ± 1.52 (4.61–11.91)
**Distance between Pectoral-Pelvic fins (69)**	6.38	17.87	4.74 ± 0.67 (3.17–6.19)	17.71 ± 1.89 (12.93–21.25)
**Prepelvic distance (71)**	18.29	51.23	13.70 ± 1.79 (9.44–18.69)	51.04 ± 2.70 (45.17–57.69)
**Prepectoral distance (72)**	11.89	33.31	8.99 ± 1.37 (6.32–13.42)	33.28 ± 2.54 (28.26–40.18)
**Lower jaw length (64)**	3.80	10.64	2.81 ± 0.40 (1.95–3.87)	10.44 ± 1.30 (7.53–14.05)
**Upper jaw length (58)**	3.68	10.31	2.65 ± 0.42 (1.75–3.56)	9.81 ± 1.15 (7.17–13.00)
**Hypural plate length (77)**	1.39	3.89	0.78 ± 0.35 (0.11–1.41)	2.85 ± 1.15 (0.48–4.85)
**Hypural plate width (74)**	1.50	4.20	0.91 ± 0.39 (0.30–1.71)	3.34 ±1.27 (1.14–5.60)

(), number of specimens; SD, standard deviation.

#### Neurocranium

The frontals are large and rectangular (Fig 4B). The parietal is not clearly assignable in any of the specimens. The lacrimal is best recognizable in the three counterparts of the paratypes 1203b´05, 1209b´05 and 1218a´05; it is laterally reduced, i.e. longer than wide and Y-shaped (Fig 3A3). It is unclear whether the lacrimal is twisted. The supraoccipital is pentagonal with two parallel horn-like processes at the posterior margin. The parasphenoid is long and elongate, and crosses the orbit approximately at its middle (Fig 3A2–3A3).

#### Branchiocranium

The shape of the dentary is elongate; its upper limb is probably as deep as the lower limb (Fig 3A4–3A5). A single row of slightly recurved conical teeth is present on the anterior half of the dentary. The anguloarticular has a median process that is pointed and clearly longer than the ventral process, which is transversely expanded and truncated (Fig 3A4–3A5). The coronoid process of the anguloarticular is pointed and small, and displays a slight concavity at the junction with the median process; the retroarticular is short (Fig 3A4–3A5).

The toothless maxilla is long and slender and has three tiny processes at its anterior end. The first of these is anteriorly directed and represents the dorsal process; the two other structures probably comprise the ventral process (Fig 3A4–3A5).

The premaxilla and maxilla are of nearly equal length (pmx: 7.0–11.8% SL, mean 8.7±1.1% vs. mx: 7.0–10.1% SL, mean 8.2±0.9%; see S2 Table). The premaxilla is considerably wider and bears teeth on the anterior third of the bone. The straight alveolar arm shows an anterior expansion and a straight posterior border (Fig 3A4–3A5). The ascending process is prominent, but relatively short, i.e. about one-sixth to one-fifth of the alveolar arm length. As far as can be discerned, the ascending process is not medially curved (Figs 3A4–3A5 and 4A1–4C).

The autopalatinum is long, slender, but clearly thicker than the maxilla and overlaps the upper portion of the quadratum; its head is bent anteriorly (Figs 3A2–3A4 and 4A1–4A2). The quadratum is triangular in shape, with an approximate angle of 110° between its dorsal and anterior margin and a long, almost straight or slightly concave, posterior margin (Figs 3A2–3A3 and 4A1–4A2). The endopterygoid is slender with the dorsal margin reduced and not in contact with the metapterygoid. The symplectic is as long and as wide as the metapterygoid (Fig 4A1–4A2).

The operculum is triangular in shape, with an angle of about 90° between its dorsal and anterior margin (Fig 4A1–4A2). Its posterior margin is slightly rounded and the dorsal articular process is extended and pointed. A rounded and half-moon-shaped suboperculum is recognizable only in some disarticulated specimens. The preoperculum is robust and L-shaped (Figs 3A2–3A3 and 4A1–4A2).

The ceratohyal is long and distally widened, and displays six branchiostegal rays. These show a stepwise increase in width from the first two rays (which are slender) posteriorly (Figs 3A2–3A3 and 4A1–4A2). Notably, no scales appear in the region of the branchiostegal rays, whereas the adjacent regions (preoperculum, operculum, pectoral girdle) are covered with scales.

The gill arches are not clearly recognizable, but one specimen (1212a/b´04) does show a pharyngobranchial tooth-plate that bears multiple rows of molariform teeth.

#### Vertebral column

In 50% of the specimens, where the vertebral column is preserved until the end of the dorsal fin, the vertebral column is straight to slightly curved, whereas in the remainder, the abdominal part of the vertebral column displays a hunchback-like curve (Fig 5A–5D). The total number of vertebrae varies from 29 to 33, of which 11–15 are abdominal (i.e. lie anterior to the first anal pterygiophore) and 17–21 are caudal (including the terminal centrum; see Table 2 and S3 Table). The first vertebra bears a distinctive median neural spine (recognizable in 36 specimens see S6 Table). Three equally long and narrow neural processes appear in specimen 1192a/b´05 (Fig 4D1–4D4); the median process probably corresponds to the median neural spine, whereas the two lateral processes represent neuroapophyses. The neural spines of the abdominal vertebrae are approximately upright, long, and reach almost to the dorsal margin of the body (Figs 3A1, 3A7, 4D1 and 4D4). Small prezygapophyses are present on the abdominal, but not on the caudal vertebrae. Thick lateral parapophyses for connection to the ribs appear on the abdominal vertebrae. Eight to 13 pairs of long ribs, starting at the second vertebra and extending to the ventral margin of the abdominal cavity, are present (S3 Table); the first up to nine pairs of ribs bears long, thin, rod-shaped epipleurals (Figs 3A6 and 4D3–4D4).

**Table 2:** Meristic values for †*Kenyaichthys* gen. et sp. nov. based on the holotype (1209a/b´04) and all other specimens.

**Characters**	**Holotype**	**All other specimens**
**Dorsal fin rays**	14	13–17
**Anal fin rays**	18	16–22
**Pectoral fin rays**	15	11–16
**Pelvic fin rays**	6	5–7
**Principal caudal fin rays**	?+8	16–22
**Procurrent dorsal caudal fin rays**	–	5–15
**Procurrent ventral caudal fin rays**	11	5–15
**Total Vertebrae**	33 (15+18)	29–33 (11–14+17–21)

The neural and haemal spines of the anterior caudal vertebrae are upright, whereas those of the following ones are posteriorly inclined. All spines of the caudal vertebrae are long and almost reach the dorsal (ventral) border of the body (see also the description of the caudal axial skeleton).

#### Girdles

The pectoral girdle displays a prominent cleithrum with an extended dorso-posterior portion, and a comparatively slender ventral portion. The posttemporal is long, thin and unforked (Fig 6A1–6A2); a supracleithrum is not clearly recognizable. It is possible that the two bones were fused. A postcleithrum is also not evident. The coracoid is long, and probably incompletely preserved; its posterior region is slightly indented below the fourth radial. The radials are robust and cubical in shape and all are approximately of the same size (Fig 6A1–6A2). The outline of the scapula is not clearly recognizable.

The pelvic bones are relatively long and triangular. An anteromedial process is lacking. The medial process and the ischial process are minute (Fig 6B1–6B2). A peculiar feature is the presence of long and pointed lateral processes.

#### Paired fins

The rounded pectoral fins are ventrolaterally inserted and of moderate size. The number of rays is 11–16; the tips of the rays do not reach the origin of the pelvic fins (Tables 1 and 2 and S3 Table, Figs 4I and 6A1).

The pelvic fins are small and round, insert beneath vertebrae 8–12 and are positioned closer to the anal fin than to the pectoral fins (Table 1: P–A vs. V–A, and Table 2). The number of rays is 5–7 (Table 2 and S3 Table, Fig 6B1–6B2).

#### Dorsal fin

The relatively small dorsal fin is inserted behind the middle of the standard length (predorsal distance 58.4 ± 2.1, see Table 1). It consists of 13–17 rays, of which the first one or two are clearly discernible as short and unbranched (Table 2 and S3 Table, Fig 7). Apart from the last ray, a long proximal radial supports each of the rays, whereas two proximal radials support one of the anteriormost rays. The last ray is not supported by a proximal radial in most cases.

The arrangement of the proximal radials in the dorsal fin is recognizable in 53 specimens of †*K*. *kipkechi* and shows pronounced polymorphism (see S6 Table). Where only one short ray is present, two proximal radials support this ray (seen in 11 specimens) or the first long ray (seen in 12 specimens; Fig 7A1–7A2). If two short rays are present, two proximal radials can either support the first short ray (seen in 15 specimens; Fig 7B1–7B2), or the second short ray (seen in 13 specimens; Fig 7C1–7C2), or the first long ray (seen in two specimen; Fig 7D1–7D2). An exception may occur in 1184R´04; it seems to show each ray supported by a single proximal radial, but putative remains of a second proximal radial are recognizable near the first short fin ray. It is therefore coded as 2/1? in S6 Table.

### Anal fin

The anal fin is slightly larger than the dorsal, and is inserted opposite, slightly behind or in front of the dorsal fin insertion (preanal distance 59.6 ± 2.5, see Table 1). It comprises 16–22 rays, of which only the first up to three are clearly seen to be short and unbranched; each ray (branched or unbranched), generally with the exception of the last, is supported by a single long proximal radial, small medial radials are also recognizable (Table 2 and S3 Table, Fig 8).

#### Caudal fin

The caudal fin is small and rounded or truncate in shape (Fig 3A7). It consists of 16–21 segmented principal rays (including the branched rays plus the first unbranched ray dorsally and ventrally) and 5–15 short procurrent rays dorsally and ventrally (Table 2 and S3 Table). The segmented and branched principal rays that are supported by the hypural plate form a coherent, uninterrupted array, without any gap in the middle of the plate (Figs 3A7 and 9). The caudal fin formula for the principal rays is 8–11/8–11 (S3 Table). The principal rays can extend to the neural and haemal spines of PU2–PU4.

#### Caudal skeleton

The axial skeleton is symmetrical: the terminal centrum is fused to a single triangular hypural plate and joined by a single parhypural and a single epural, each of which provides support for one or two segmented principal rays. The hypural plate is recognizable in all specimens of †*K*. *kipkechi* and shows polymorphism in its dimensions (see Table 1 and S1 Table), with the length ranging from 0.5–4.9% of SL and the width from 1.1–5.6% of SL. It is <1.0% of SL in eight specimens, 1.3–2.0% in 12 specimens, 2.1–2.9% in 18 specimens and 3.0–4.9% of SL in 39 specimens (Fig 10A–10D).

The extension of the procurrent rays and number of preural vertebrae (PU) is recognizable in 90 specimens; 49 of them possess five preural vertebrae, the remainder have six (see S3 Table). The preural vertebrae are characterized by long neural and haemal spines supporting the caudal rays; the neural and haemal spines of the preceding vertebrae are clearly shorter (Fig 3A7). The proximal portions of the neural and haemal spines of PU2 do not show a constriction.

The uroneural (= stegural) is usually not visible, but in some specimens it is recognizable as a short and tiny structure closely attached to the proximal portion of the upper segment of the hypural plate and terminal centrum (Fig 2B1–2B2); lateral processes are not recognizable.

Supernumerary neural and/ or haemal spines can be observed in 32 out of 127 specimens in which neural and/ or haemal spines are visible in the caudal region (see S3 Table). The additional spine was identified as duplicated if each of the two spines had an individual base (observed in 23 specimens), and as split when both spines shared the same base (nine specimens). Twenty specimens show duplicated haemal and/ or neural spines of PU2. Two specimens show the neural spines of PU5 duplicated, and a single specimen shows duplicated neural and haemal spines on PU3. Five specimens show split neural and/or haemal spines of PU2, and two further specimens display split neural spines of PU3. The remaining two specimens show a split spine of PU2, but it is unclear whether it is the haemal or the neural spine (see S3 Table).

The proximal part of the parhypural is recognizable in 78 specimens and is polymorphic, i.e. it may either be reduced and lack contact with the terminal centrum and hypural plate (seen in 64 specimens; Figs 2B1–2B2 and 9A1–9A2), or articulate with the terminal centrum and at least partially with the hypural plate (seen in two specimens; Fig 11A1–11A2), or may even be reduced and lack contact with the terminal centrum, but is at least partially fused to the hypural plate (seen in 12 specimens; see S6 Table). If the parhypural is reduced, the proximal part can display a projection that faces away from the terminal centrum and the major part of the bone can be straight (26 specimens) or curved (three specimens); in the remaining 42 specimens the proximal part is continuous with the main axis of the parhypural and the remaining part of the parhypural is straight (33 specimens) or curved (nine specimens). The condition of the parhypural in the remainder five specimens is not recognizable due to insufficient preservation (see S6 Table).

The epural is clearly recognizable in 82 specimens and does not make contact with the terminal centrum in any of the specimens, but can be fused to the hypural plate (seen in one specimen). 55 specimens show an epural without an anterodorsal projection; the epural is curved in 35, and straight in 20 of these specimens. The 27 remaining specimens display a straight (19 specimens) or curved (eight specimens) epural with an anterodorsal projection. Additional six specimens have the caudal skeleton preserved, but the orientation of the specimen is unclear (see S6 Table).

#### Spine ratios

Both the neural spine PU2/PU4 and PU2/PU5 ratios were >1.0 in most specimens (Table 3 and S4 Table). The haemal spine of PU2 is slightly wider than that of PU4 and PU5 in most specimens (ratios >1.0 and <2.0), but several specimens revealed also ratios <1.0 and ≥2.0 (Table 3 and S4 Table). Furthermore, both the neural and haemal spine PU3/PU5 ratios were >1.0 in most specimens (Table 2 and S4 Table).

**Table 3:** Spine ratios (means and ranges) of †*Kenyaichthys* gen. et sp. nov. and the recent cyprinodontiform species used for comparison.

**Species**	**NS2/NS4**	**NS2/NS5**	**HS2/HS4**	**HS2/HS5**	**NS3/NS5**	**HS3/HS5**
***†Kenyaichthys* gen. et sp. nov. (all specimens)**	1.6±0.5 / 1.1–4.3 (75) w	1.8±0.5 / 1.1–3.1 (66) w	2.4±0.5 / 2.0–3.7 (13) dw	2.3±0.4 / 2.0–3.7 (19) dw	1.8±0.5 / 1.1–3.3 (70) w	1.6±0.5 / 1.1–3.0 (70) w
			1.4±0.2 1.1–1.9 (48) sw	1.5±0.2 1.2–1.8 (47) sw		
	0.8±0.2 / 0.5–1.0 (7) ne	0.9±0.2 / 0.6–1.0 (13) ne	0.9±0.1 / 0.6–1.0 (15) ne	0.8±0.1 / 0.6–1.0 (8) ne	0.9±0.2 / 0.3–1.0 (15) ne	0.8±0.2 / 0.5–1.0 (14) ne
***Aphanius sophiae***	3.2±1.3 / 1.6–5.4 (19) w	4.3±1.3 / 2.6–8.0 (19) w	3.1±0.8 / 2.0–4.4 (17) dw	4.3±1.2 / 2.8–7.2 (19) dw	4.0±1.3 / 2.3–6.5 (19) w	3.9±1.3 / 2.2–6.6 (19) w
			1.6±0.3 / 1.5–1.8 (2) sw	–		
	–	–	–	–	–	–
***Aphanius farsicus***	4.1±2.0 / 2.3–7.0 (6) w	5.0±1.4 / 3.5–7.5 (6) w	3.6±2.0 / 2.0–6.2 (5) dw	5.4±1.6 / 3.5–7.8 (6) dw	4.3±1.5 / 1.5–5.8 (6) w	4.8±1.7 / 2.7–7.3 (6) w
			1.9 (1) sw	–		
	–	–	–	–	–	–
***Aphanius arakensis***	4.4±3.9 / 2.1–12.0 (6) w	5.4±1.9 / 2.8–7.7 (5) w	4.6±3.4 / 2.1–10.5 (5) dw	6.0±2.2 / 4.0–10.0 (6) dw	5.4±1.9 / 3.0–7.8 (5) w	5.5±1.5 / 3.3–7.2 (6) w
			1.7 (1) sw	–		
	–	–	–	–	–	–
***Aphanius mesopotamicus***	3.1±0.7 / 2.3–3.5 (3) w	3.5±1.5 / 2.1–5.7 (4) w	2.8±0.2 / 2.6–5.3 (3) dw	3.7±1.2 / 2.7–5.3 (4) dw	3.1±0.7 / 2.4–4.0 (4) w	3.7±1.5 / 2.3–5.7 (4) w
			–	–		
	1.0 (1) ne	–	1.0 (1) ne	–	–	–
***Pachypanchax playfairii***	3.2±1.8 / 1.9–4.4 (2) w	5.0±1.8 / 3.8–6.3 (2) w	2.3 (1) dw	3.2±0.6 / 2.8–3.6 (2) dw	3.2±0.0 / 3.1–3.2 (2) w	2.8±0.8 / 2.3–3.4 (2) w
			1.6 (1) sw	–		
	–	–	–	–	–	–
***Nothobranchiidae***	2.6±0.6 / 1.8–3.7 (10) w	2.9±1.1 / 1.4–5.4 (9) w	2.9±0.7 / 2.0–4.0 (7) dw	3.4±1.4 / 2.0–5.3 (6) dw	1.9±0.8 / 1.2–3.6 (7) w	2.2±1.1 / 1.2–4.5 (9) w
			1.5±0.1 / 1.4–1.5 (3) sw	1.5±0.2 / 1.3–1.8 (3) sw		
	–	–	–	1.0 (1) ne	0.9±0.1 / 0.8–1.0 (2) ne	1.0 (1) ne

(), number of specimens; dw, distinctively wider; HS, haemal spine of preural centrum; ne, narrower or equal; NS, neural spine of preural centrum; w, wider; sw, slightly wider. Blank cells indicate unassigned character state.

#### Scales

Cycloid scales (Fig 12A–12B) are visible on different parts of the body in 98 specimens, of which 22 display scales on the whole body from the preoperculum to the hypural plate. Scales are generally absent from the caudal fin base, with the exception of four specimens that show one to four scales here (1153´04, 1 scale; 1175´04, 3–4 scales; 1206(1)/1211´04, 1 scale; 1228(1)/1237R(1)´04, 1–2 scales) (Fig 4B). The rostral field is only recognizable in the scales on the operculum and the pectoral girdle, which show 7 to 13 radii (Fig 12A–12B). The squamation pattern on the head that is indicative for the Rivulidae [48] cannot be identified. Most scales on the body and some scales on the head and hypural plate show an abnormal shape of the central portion, i.e. the scale focus is large and irregular (for scale terminology see [44]) (Fig 12B). The estimated number of scales in the lateral series is 37–40 (based on 1237R(1)´04). Mean dimensions of eight key scales (from the third or fourth rows below the dorsal fin) from four different specimens (1171R´04, 1199b´04, 1223R´04, 1237R(1)´04; two scales each) are: 0.44 ± 0.05 mm length (range 0.36–0.52 mm) and 0.43 ± 0.07 mm width (range 0.36–0.53 mm) (see S5 Table).

#### Granulation structures

7% (n = 11) of the specimens show a regularly distributed granulation-like structure between the spines and rays of all fins, around the entire vertebral column, on individual vertebrae, and sometimes also on the head (Fig 12C). Most likely these structures can be interpreted as corrosion of bones in the course of the fossilisation process.

### Analysis of extant material

The fossil specimens show variation in the length and width of the hypural plate, in the numbers of preural vertebrae, and also in the width of the haemal and neural spines of the preural vertebrae. In order to understand the taxonomic meaning of this variation, we therefore asked whether these characters show a similar tendency to vary in extant killifish.

#### Hypural plate dimensions

We used four species of *Aphanius* (see comparative material) that all belong to the same young (Holocene) evolutionary lineage based on molecular data [53] and therefore represent an excellent model to compare intra- and interspecific variation in closely related species. In all, ten females (8 c&s and 2 xr) and nine males (all c&s) of *A*. *sophiae*, three females and three males (all xr) of *A*. *farsicus*, three females (all xr) and three males (2 c&s and 1 xr) of *A*. *arakensis*, and two females and two males (all c&s) of *A*. *mesopotamicus* were analysed with regard to the hypural plate dimensions in the two sexes (Table 4 and S7 Table, mean values and ranges are given in % of SL). The measurements reveal that the hypural plate has a large size range within these four species. However, in *A*. *sophiae* and *A*. *arakensis* the hypural plate length is significantly different between males and females (T-Test, p<0.05, see Table 4). No unambiguous signals were obtained for such sex dimorphism in *A*. *farsicus* and *A*. *mesopotamicus*.

**Table 4:** Hypural plate dimensions of the four species of *Aphanius* used for comparison.

**Species**	**Sex**	**n**	**Lh**		**wH**	
			Mean	range	mean	range
***Aphanius sophiae***	m	9 (c&s)	5.83% ± 0.36*	5.15–6.31%	8.45% ± 0.48	7.71–9.27%
	w	10 (8 c&s, 2 xr)	5.31% ± 0.64*	4.22–6.49%	8.03% ± 0.77	6.99–9.73%
***Aphanius farsicus***	m	3 (xr)	5.54% ± 0.48	4.99–5.87%	8.12% ± 0.72	7.62–8.95%
	w	3 (xr)	5.43% ± 0.21	5.26–5.66%	8.07% ± 0.89	7.41–9.08%
***Aphanius arakensis***	m	3 (2 c&s, 1 xr)	5.85% ± 0.22*	5.60–6.02%	7.79% ± 0.79	7.31–8.71%
	w	3 (xr)	5.13% ± 0.30*	4.85–5.45%	7.50% ± 0.77	6.92–8.38%
***Aphanius mesopotamicus***	m	2 (c&s)	4.93% ± 0.04	4.90–4.95%	7.19% ± 0.66	6.72–7.65%
	w	2 (c&s)	4.47% ± 0.16	4.36–4.58%	7.05% ± 0.09	6.98–7.11%

Significant differences between sexes are indicated with

* (T-Test, p<0.05).

n, number of specimens; c&s, cleared and stained; lH, length of hypural plate; wH, width of hypural plate; xr, x-ray.

#### Number of preural vertebrae

**Fig 13:**
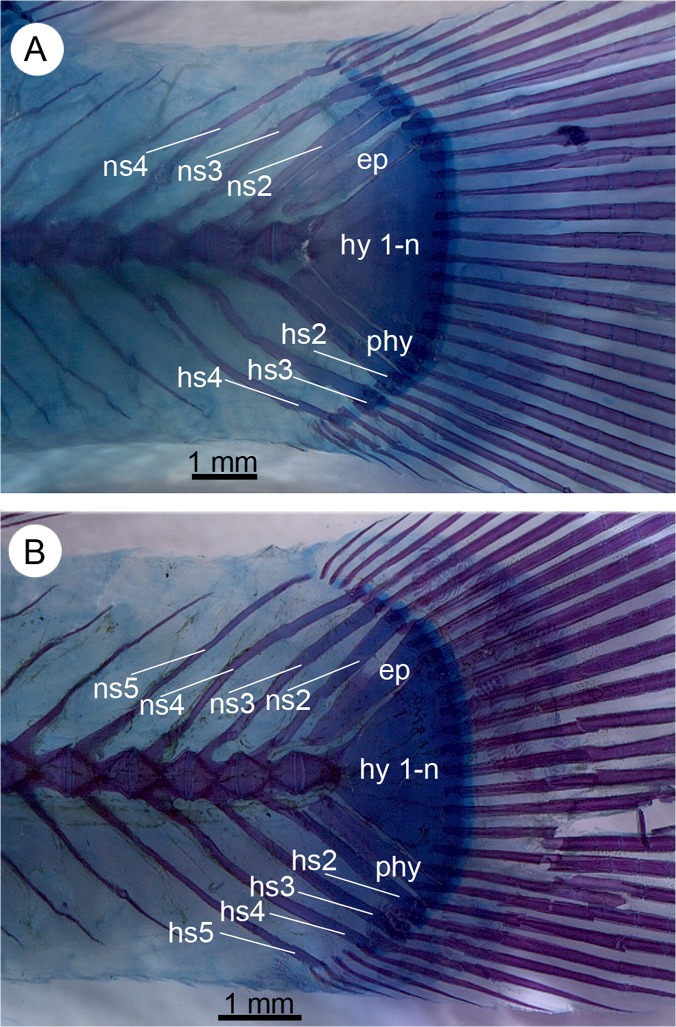
Intraspecific variation in the number of preural vertebrae in *Nothobranchius orthonotus* (MRAC A4-039-P-0133-0134). **A** Four preural vertebrae; **B** five preural vertebrae © Royal Museum for Central Africa Tervuren (Belgium). Abbreviations: ep, epural; hs2–5, haemal spine of preural vertebrae 2–5; hy 1-n, hypural plates 1–n; ns2–5, neural spine of preural vertebrae 2–5; phy, parhypural.

The degree of within-species variation in preural vertebrae number was examined based on two specimens of the Aplocheilidae (*Pachypanchax playfairii;* two c&s), a total of 10 specimens of the Nothobranchiidae (*Aphyosemion castaneum*, *Epiplatys sexfasciatus*, *Foerschichthys flavipinnis*, *Fundulopanchax sjoestedti*, *Nothobranchius orthonotus*; two c&s each), and a total of 29 specimens of the Cyprinodontidae (*Aphanius sophiae*, 13 seven c&s specimens out of the specimens used above with sufficient preservation of the caudal fin rays; *A*. *farsicus*, six xr; *A*. *arakensis*, four xr, two c&s; *A*. *mesopotamicus*, four c&s). We found intraspecific variation of the preural vertebrae number in *N*. *orthonotus* (four and five preural vertebrae, see Fig 13) and in the four examined species of *Aphanius* (three and four preural vertebrae). The remaining specimens consistently displayed four preural vertebrae (S7 Table). It is therefore clear that intraspecific variation of preural vertebrae number, as observed in †*Kenyaichthys*, is not exceptional as it is also present in extant species.

#### Ratios of neural and haemal spines of preural vertebrae

The ratios of the widths of haemal and neural spines of PU2/PU4, PU2/PU5 and PU3/PU5 were considered to be important at multiple taxonomic levels in previous studies (see Discussion). They include:

- a synapomorphy for Cyprinodontiformes vs. Adrianichthyidae (Beloniformes) (neural spine of PU2 wider than neural spines of PU4 and PU5 vs. about equal);
- a synapomorphy for Cyprinodontoidei vs. Aplocheiloidei (neural and haemal spines of PU3 wider than spines of PU5 vs. about equal);
- a synapomorphy for Nothobranchiidae/Rivulidae vs. all other cyprinodontiform families (haemal spine of PU2 slightly wider than haemal spines of PU4 and PU5 vs. distinctively wider).

In the case of (iii), however, it is not clear from previous studies how “slightly wider” and “distinctively wider” should be defined. We consider here ratios of >1.0 and <2.0 as slightly wider and ratios of ≥2.0 as distinctively wider.

We used the comparative material described above to verify the phylogenetic significance of these characters. Ratios between spines were calculated based on the maximal width of the respective spine (see Fig 2B and S1 Fig).

- The neural spine of PU2 was wider than the neural spines of PU4 and PU5 in almost all specimens studied (Table 3 and S8 Table), as expected for a cyprinodontiform species (see Table 5). The single exception is specimen ZM-CBSUZG 363 of *Aphanius mesopotamicus*, which reveals the neural spine of PU2 as wide as the neural spine of PU4.
- The neural and haemal spines of PU3 were wider than those of PU5 in the cyprinodontoid specimens (Tables 3 and S8). However, PU3 neural and haemal spines were also wider than PU5 spines in nine and 11 of the aplocheiloid specimens, respectively (Tables 3 and S8), rather than being about equal as expected for the Aplocheiloidei from previous work (see Table 6). The mean values of the aplocheiloid specimens are significantly smaller than that of the cyprinodontoid specimens (T-Test, p<0.0001 for neural and haemal spine ratios), however, the ranges of PU3/PU5 ratios overlap between the two groups (Tables 3 and S8).
- In seven of the ten studied extant nothobranchiid specimens, the haemal spine of PU2 is distinctively wider (ratio ≥2.0) than those of PU4. Moreover, six of the ten specimens show a ratio of ≥2.0 for PU2/PU5. However, only the character state “slightly wider” (1.0< ratio <2.0) is expected for Nothobranchiidae and Rivulidae from previous work (see above and Table 7). Moreover, ranges for PU2/PU4 and PU2/PU5 ratios display overlap between all studied species (Table 3 and S8 Table), and the mean values of the nothobranchiid specimens are not significantly smaller than seen in the aplocheilid species *P*. *playfairii* (T-Test, p>0.05). On the other hand, the PU2/PU5 mean value for the nothobranchiid species is significantly smaller than in the studied cyprinodontoid species (T-Test, p<0.01), as expected from literature data.

**Table 5:** Summary of the osteological synapomorphies for the Cyprinodontiformes and comparisons with †*Kenyaichthys* gen. et sp. nov. compiled from [1], [3], [48], [50], [56] and [57].

**Synapomorphy (author and character number)**	**Cypr**	**†*Ken***
Distinct expansion of the alveolar arm of premaxilla ([3]; [48], char. 13)	+	+
Dorsal edge of mesopterygoid reduced ([48], char. 32)	+	+
Urohyal deep ([48], char. 37)	+	n.a.
Ventral process of the lateral portion of second epibranchial absent ([48], char. 55)	+	n.a.
Mesethmoid region slightly anterior to lateral ethmoid ([48], char. 70)	+	n.a.
Ventrolateral pectoral fin insertion ([3]; [48], char. 74)	+	+
First postcleithrum scale-like ([3]; [48], char. 78)	+	n.a.
Anteromedial process of pelvic girdle absent ([48], char. 84)	+	+
Caudal fin skeleton symmetrical ([3]; [48], char. 86; [50], char. 37; [55])	+	+
Caudal fin truncate or rounded ([3]; [48], char. 87; [56]; [57])	+	+
Caudal fin rays continuously arranged ([1], char. 3)	+	+
Complete ankylosis of upper hypurals and terminal centrum ([1], char. 7)	+	+
Stegural minute ([1], char. 5)	+	+
One single epural ([1], char. 1; [3]; [56])	+	+
Blade-like epural ([1], char. 2; [56])	+	?
First pleural rib on second vertebra ([3]; [48], char. 95; [56]; [57])	+	+
Preural vertebra 2, well-developed neural spine with distal tip acting in support of caudal fin rays ([1], char. 4)	+	+
Preural vertebra 2, neural spine wider than neural spines of preural vertebrae 4 and 5 ([1], char. 6)	+	P
12–16 or 20–25 radii on anterior abdominal scales ([48], char. 105)	+	?

+, present;

P, polymorphic; n.a., not applicable;?, uncertain; Cypr = Cyprinodontiformes;

†*Ken* = †*Kenyaichthys*.

**Table 6:** Summary of the osteological synapomorphies for the Cyprinodontoidei and Aplocheiloidei and comparisons with †*Kenyaichthys* gen. et sp. nov. compiled from [1], [3], [48] and [50].

**Synapomorphy (author and character number)**	**Cypr**	**Apl**	**†*Ken***
Posterior indentation of the alveolar arm of premaxilla (vs. absent) ([3]; [48], char. 14)	+	0	0
Dentary deep (vs. slender) ([3]; [48], char. 19)	+	0	0
Head of autopalatinum bent anteriorly, displaced laterally relative to the main axis of the bone (vs. continuous with the main longitudinal axis of the bone) ([48], char. 27)	+	0	I
Metapterygoid absent (vs. present) ([3]; [48], char. 34)	+	0	0
Dorsal hypohyal absent (vs. present) ([3]; [48], char. 41)	+	0	n.a.
First basibranchial absent (vs. present) ([3]; [48], char. 45)	+	0	n.a.
Ventral process of fourth ceratobranchial expanded medially (vs. short) ([48], char. 48)	+	0	n.a.
Ventral process of lateral portion of second epibranchial absent (vs. present) ([48], char. 55)	+	0	n.a.
Second pharyngobranchial expanded ventrally (vs. not expanded) ([48], char. 58)	+	0	n.a.
Lacrimal approximately rectangular (vs. approximately triangular) ([48], char. 71)	+	0	0
Neuroapophyses on the first vertebra separated (vs. united) ([48], char. 96)	+	0	n.a.
Stegural, ventral portion with lateral spine-like process (vs. no spine-like process) ([1], char. 10)	+	0	0
Neural and haemal spines of PU3 wider than spines anterior to PU4 vs. about equal ([1], char. 9) (but see also text)	+	0	P
Dorsal process of maxilla short, anteriorly directed, not parallel to ventral process (vs. long, medially directed or vestigial) ([48], char. 1)	0	+	+
Main axis of the ventral process of maxilla slightly curved, tip directed posteriorly (vs. directed anteriorly) ([48], char. 4)	0	+	n.a.
Coronoid process of anguloarticular reduced (vs. not reduced) ([48], char. 23; [50], char. 7)	0	+	+
Lateral flange of hyomandibula expanded posterodorsally (vs. short) ([48], char. 35)	0	+	n.a.
Anterior portion of basihyal widened (vs. slender) ([48], char. 43)	0	+	n.a.
A distinct anteromedial process on second hypobranchial directed toward second basibranchial (vs. absent) ([48], char. 46)	0	+	n.a.
A distinct posterior process on fourth epibranchial (vs. absent) ([48], char. 57)	0	+	n.a.
Dentition on second pharyngobranchial reduced (vs. not reduced) ([48], char. 59)	0	+	n.a.
Vomerine teeth present (vs. absent) ([3]; [48], char. 60)	0	+	n.a.
Wide process on the anterior portion of lateral ethmoid (vs. narrow or no process) ([48], char. 63)	0	+	n.a.
Neurocranium flattened (vs. not flattened) ([48], char. 66; [50], char. 30)	0	+	+
Dermosphenotic short (vs. elongate or minute) ([48], char. 73)	0	+	n.a.
Medial process of pelvic girdle short (vs. long) ([3]; [48], char. 83)	0	+	+
Distal radial of anal fin with an expanded posteroventral rim (vs. without ventral extensions) ([48], char. 93)	0	+	n.a.
Supraorbital canals open with neuromasts exposed externally (vs. closed) ([3]; [48], char. 98)	0	+	n.a.
Anterior naris opening at the tip of a distinctively cylindrical structure (vs. flat, no fleshy structure or situated on prominent fleshy structure) ([48], char. 100)	0	+	n.a.
20 to 25 radii on anterior abdominal scales (vs. 12 to 16) ([48], char. 105)	0	+	?

+, present;

0, absent;

P, polymorphic; n.a., not applicable; I, intermediate;?, uncertain; Cypr = Cyprinodontoidei; Apl = Aplocheiloidei;

†*Ken* = †*Kenyaichthys*.

**Table 7:** Summary of the osteological synapomorphies for the Aplocheilidae (sensu [48]), Nothobranchiidae and Rivulidae and comparisons with †*Kenyaichthys* gen. et sp. nov. compiled from [1], [3], [48], [49], [50], [55], and [61].

**Synapomorphy (author and character number)**	**Apl**	**Noth**	**Riv**	**†*Ken***
Ventral process of anguloarticular expanded (vs. not expanded) ([48], char. 22; [61])	+	+	0	+
Supracleithrum and posttemporal coossified (vs. not fused) ([3]; [48], char. 76; [50], char. 48)	+	+	0	+?
Posterior tip of the ascending process of premaxilla curved medially (vs. plan) ([3]; [48], char. 15; [50], char. 2)	+	+	0	0
Bifid epipleural ribs (vs. rod-shaped epipleural ribs) ([3]; [49], char. 98; [55], char. 18)	0	+	0	0
Keel-shaped lateral process on middle part of terminal centrum (vs. smooth terminal centrum) ([1], char. 20)	0	+	0	0
Twisted and reduced lacrimal (vs. flat with wide posterior rim) ([3]; [50], char. 31)	0	+	+	+
Distinctive neural spine on first vertebra narrow or broad (vs. neural spine on first vertebra absent) ([49]; [50]; char. 34; [61])	0	+	+	+
Shortened laminar proximal end of parhypural (vs. not reduced, overlapping terminal centrum) ([1], char. 12; [50], char. 39)	0	+	+	P
Long first dorsal fin ray attached to two proximal radials, preceded by one or two short fin rays (vs. single long first dorsal fin ray attached to two proximal radials) ([3]; [48], char. 94; [50], char. 44)	0	+	+	+
Completely attached orbital rim (vs. ventrally attached) ([3]; [48], char. 103; [50], char. 58)	P	+	+	n.a.
Preural vertebra 2, haemal spine slightly wider than haemal spines of preural vertebrae 4 and 5 (vs. distinctively wider) ([1], char. 19; [50], char.43) (but see also text)	0	+	+	P
Posterior border of distal portion of premaxilla straight (vs. indented) ([49], char. 2; [50], char. 1)	0	0	+	+
Ventral process of maxilla bent (vs. straight to slightly curved) ([48], char. 4)	0	0	+	n.a.
Coronoid process of dentary narrowed (vs. broad) ([49], char. 13; [50], char. 5)	0	0	+	0
Ventral tip of autopalatinum not reaching the quadratum (vs. long, reaching quadratum) ([3]; [48], char. 28; [50], char. 8)	0	0	+	0
Thin, C-shaped preoperculum (vs. robust and L-shaped preoperculum) ([48], char. 36; [50], char. 13; [61])	0	0	+	0
Reduced uncinate process of third epibranchial (vs. elongate process) ([3]; [48], char. 56; [61])	0	0	+	n.a.
Pronounced retrorse process of lateral ethmoid (vs. narrow, wide or absent) ([48], char. 63)	0	0	+	n.a.
Reduced lateral rim of frontal (vs. not reduced) ([48], char. 69; [50], char. 29)	0	0	+	0
Minute dermosphenotic (vs. elongate or short) ([3]; [48], char. 73)	0	0	+	n.a.
First postcleithrum absent (vs. present) ([3]; [48], char. 77; [61])	0	0	+	n.a.
Branchiostegal and opercular membranes united (vs. separated) ([3]; [48], char. 102; [49], char. 164; [50], char. 57; [61])	0	0	+	0
Frontal scales arranged circularly around a central A-scale (vs. transversely arranged) ([3]; [48], char. 104)	0	0	+	n.a.

+, present;

0, absent; P, polymorphic; n.a., not applicable;?, uncertain; Apl = Aplocheiloidei; †*Ken* = †*Kenyaichthys*; Noth, Nothobranchiidae; Riv, Rivulidae.

Apart from those few specimens that show the neural spine smaller to equal to those of PU4 and/or PU5, all specimens of †*Kenyaichthys* exhibit a neural spine on PU2 that is wider than those of PU4 and PU5 (see above), like the studied extant cyprinodontiform species, with the exception of one specimen of *Aphanius mesopotamicus* (see Table 3). Furthermore, †*Kenyaichthys* displays mean values and ranges of PU3/PU5 neural and haemal spine ratios that are comparatively close to the mean values of the studied aplocheiloid specimens (see Table 3). In addition, the haemal spine PU2/PU4 mean value of †*Kenyaichthys* is closer to the respective value of the studied aplocheilid specimens, whereas the haemal spine PU2/PU5 mean value of †*Kenyaichthys* is closer to the respective value of the studied nothobranchiid specimens (see Table 3).

### Phylogenetic reconstruction

**Fig 14:**
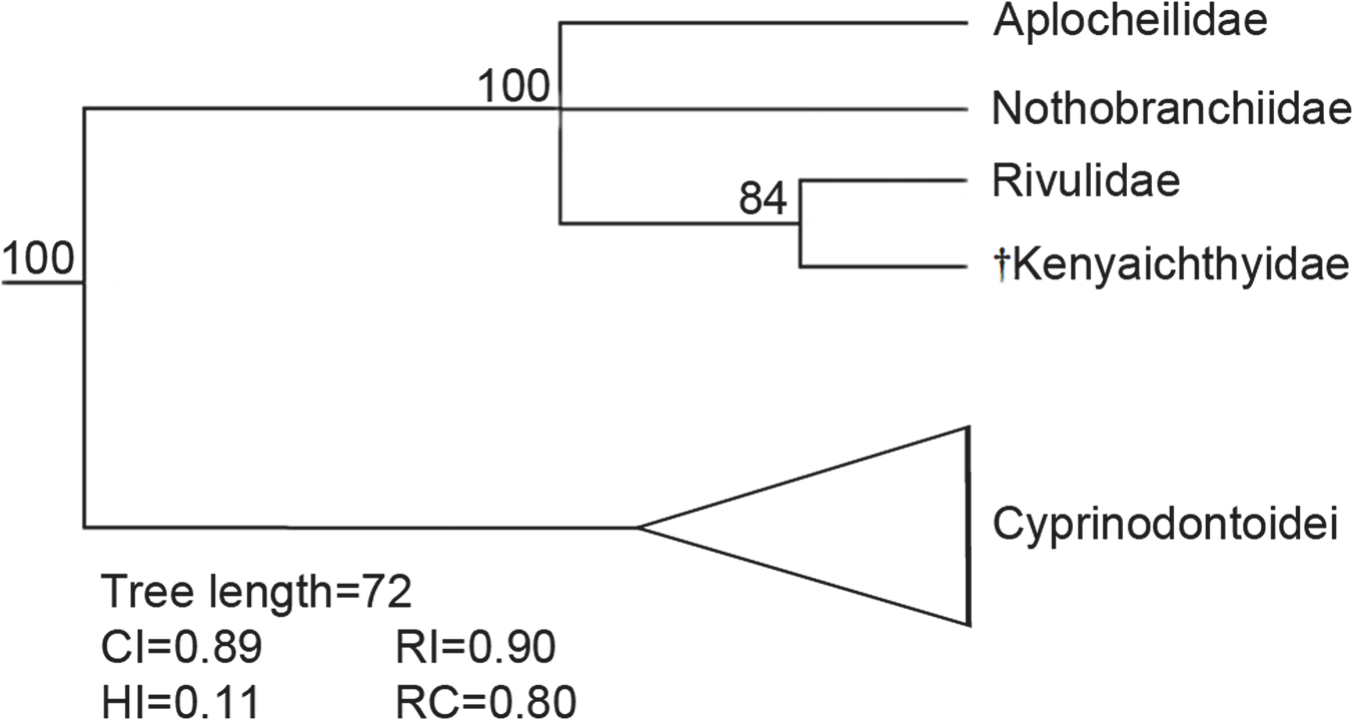
50% majority-rule consensus tree for the Cyprinodontiformes and †*Kenyaichthys* gen. et sp. nov. (red arrow) based on 72 morphological characters as used in the studies of Costa [1, 48, 49, 50, 55] created using PAUP [51]. Numbers above nodes refer to bootstrap values (based on 1000 replicates). Abbreviations: CI, consistency index; HI, homoplasy index; RI, retention index; RC, rescaled consistency index.

**Fig 15:**
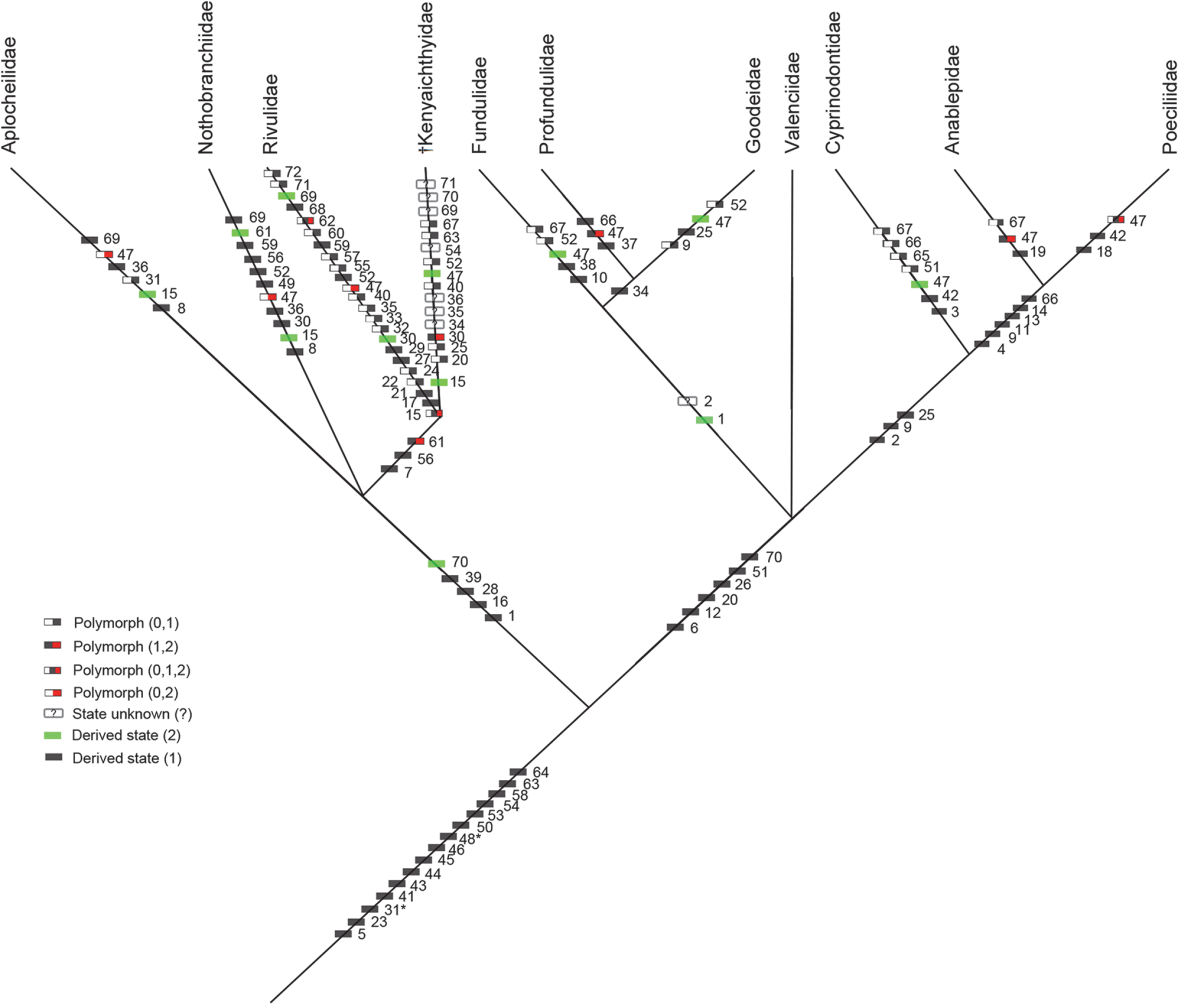
50% majority-rule consensus tree for the Cyprinodontiformes and †*Kenyaichthys* gen. et sp. nov. with all the 72 morphological characters mapped. * Indicates character reversals: *character: 31 synapomorphy for all Cyprinodontiformes, but reversal in some Poeciliidae and *Aplocheilus*; *character 48: synapomorphy for all Cyprinodontiformes with reversal in some Nothobranchiidae and Aplocheilidae.

To elucidate the systematic position of †*Kenyaichthys*, a phylogenetic analysis based on 72 morphological characters was conducted (according to [1, 48, 50]; see S9 and S10 Tables and S1 NEXUS File) (Figs 14 and 15). The character “spines of PU3 wider vs. equal compared to the spines of PU5” [1] was not used because our data obtained from the extant specimens indicated that the PU3/PU5 ratios of neural and haemal spines cannot be reliably used to separate cyprinodontoid from aplocheiloid species. The character “haemal spine of PU2 slightly vs. distinctively wider compared to PU4 and PU5” [1] was also omitted because our data showed that the PU2/PU4 and PU2/PU5 ratios of the haemal spines overlap between the Aplocheilidae and Nothobranchiidae (see above). Furthermore, we discarded the character “mouth position superior vs. terminal” because, based on the studies of Costa [48, 50, 54], it has not been unambiguously determined whether the superior mouth position is an apomorphic or plesiomorphic trait. Furthermore, the character “12 to 16 and 20 to 25 radii vs. four to six radii on the anterior abdominal scales” [48] could not be checked in the fossils because †*Kenyaichthys* only sparsely revealed radii on its scales, and such scales were exclusively found near the shoulder girdle and the operculum. The condition of this character in †*Kenyaichthys* was therefore coded as “?” in the character matrix (see also Tables 5 and 6). It should be noted that the presence of a pelvic girdle lateral process could not yet be used in phylogenetic analyses because this character remains to be explored for most extant groups.

The outcome of the phylogenetic analysis clearly places †*Kenyaichthys* within the Aplocheiloidei (Figs 14 and 15). It emerges as sister to the Rivulidae, while †*Kenyaichthys* together with Rivulidae are sister to the Aplocheilidae and Nothobranchiidae, which appear as unresolved polytomy. The Cyprinodontoidei display the same topology as in previous studies.

## Discussion

### Relationship of †*Kenyaichthys* to Cyprinodontiformes

The foregoing investigation of our †*Kenyaichthys* material reveals that it exhibits 12 of the 19 synapomorphies that are diagnostic for the Cyprinodontiformes (Table 5), among them the well-developed neural spine of PU2. While most of the studied fossil specimens of †*Kenyaichthys* possess a neural spine of PU2 that is wider than the neural spines of PU4 and PU5, seven specimens displayed the neural spine of PU2 not wider than that of PU4, and 13 specimens showed the neural spine of PU2 not wider than the neural spine of PU5 (see Table 3). This condition has only been described for the Adrianichthyidae (Order Beloniformes) [1], which is sister to the Cyprinodontiformes [5, 58, 59]. However, our data reveal that exceptions may occur as observed in *Aphanius mesopotamicus* for the neural spine PU2/PU4 ratio. In Atheriniformes and other Beloniformes the neural spine of PU2 is not fully developed [1]. Beloniformes are characterized by (among other traits) a “lower lobe of the caudal fin with more principal fin rays than [the] upper lobe” ([60] page 156). This is definitely not the case in six specimens, (1177´04, 1194´04, 1206(1)/1211´04, 1218´04, 1227´04, and 1228(1)/1237R(1)´04) and cannot be ascertained in the remaining specimens. We therefore confidently assign the genus †*Kenyaichthys* to the Cyprinodontiformes.

### †*Kenyaichthys*–A member of Aplocheiloidei or Cyprinodontoidei?

Costa [1, 48, 50, 55] provided a series of synapomorphies for the further classification of killifishes (Tables 6–7) of which several were based on osteological characters and are thus in principle applicable to fossils. Seventeen osteological synapomorphies define the Aplocheiloidei and 13 osteological synapomorphies are diagnostic for the Cyprinodontoidei (Table 6), but 12 and six of them, respectively, refer to delicate structures that are not preserved in the fossils studied here (Table 6). We have found that †*Kenyaichthys* displays four synapomorphies of the Aplocheiloidei, namely the presence of a short dorsal process on the maxilla that is anteriorly directed and probably not parallel to the ventral process (Fig 3A4–3A5), a reduced coronoid process on the anguloarticular (Fig 3A4–3A5), a flattened neurocranium (Figs 3A2, 4B, 4C1 and 4D1), and a short medial process of the pelvic girdle (Fig 6B1–6B2). Of the 13 osteological synapomorphies that define the Cyprinodontoidei, one concerns the width of the spines of PU3 relative to those of PU5. According to Costa [1], in Cyprinodontoidei the spines of PU3 are wider than those on PU5 (apomorphic condition), whereas the Aplocheiloidei have narrow neural and haemal spines of PU3 that are no wider than their counterparts on PU5 (plesiomorphic condition). However, our data derived from the extant specimens clearly indicate that (i) species assigned to the Aplocheiloidei do not consistently exhibit narrow neural and haemal spines of PU3, and that (ii) the PU3/PU5 ratios can overlap with those of the Cyprinodontoidei (see above). In †*Kenyaichthys* the spines of PU3 are wider than those of PU5 (with the exception of 16 and 14 specimens, for neural and haemal spines respectively), but the range of the PU3/PU5 ratios is comparatively close to that of the studied aplocheiloid specimens (see above and Table 3). This might be an additional hint that the fossil material belongs to the Aplocheiloidei, as deduced from the other synapomorphies mentioned above.

Moreover, †*Kenyaichthys* displays two characters that appear to be intermediate between the extant Cyprinodontoidei and Aplocheiloidei, one at subordinal level, the other at family level. One is the curvature of the autopalatinum head, which is not straight as seen in the Aplocheiloidei (= plesiomorphic state), but also not as sharply bent as is typical for the Cyprinodontoidei (= apomorphic state; see [48] page 542 and Fig 4F). The second feature is the shape of the posterior margin of the quadratum, which does not show the strongly concave form seen in the two cyprinodontoid families Profundulidae and Goodeidae (= apomorphic state; see [48] page 544 and Fig 4E–4F), but is not as rounded as in the aplocheiloid and remaining cyprinodontoid families. The presence of such “intermediate” characters could indicate that †*Kenyaichthys* is in a “premature” evolutionary state (see below).

Another interesting character of †*Kenyaichthys* is the presence of five or six preural vertebrae, whereas Aplocheiloidei and Cyprinodontoidei usually possess four or five preural vertebrae. Six preural vertebrae have only been reported for three distantly related cyprinodontoids, i.e. the anablepid *Anableps*, the cyprinodontid *Orestias* and the fundulid *Fundulus* [1]. Costa [1] argued that the increase in the number of preural vertebrae has evolved independently in these genera. It is therefore possible that this trait is an independent acquisition in †*Kenyaichthys* as well and has no taxonomic meaning.

In summary, †*Kenyaichthys* displays a unique combination of characters, four of which are apomorphic for the Aplocheiloidei, two are intermediate, and one may represent parallel evolution. Together with the phylogenetic analysis (Figs 14 and 15), these data support the interpretation of †*Kenyaichthys* as a member of the Aplocheiloidei.

### Relationships of †*Kenyaichthys* within the Aplocheiloidei

We have also considered the synapomorphic characters of the extant aplocheiloid families, i.e. Aplocheilidae, Nothobranchiidae and Rivulidae (Table 7). However, the only synapomorphy for the Aplocheilidae *sensu strictu* (according to [50]) is a black spot on the dorsal fin of the females, a character that cannot be assessed in a fossil. Therefore we have used here and in Table 7 the synapomorphies that were defined for the clade of the Aplocheilidae sensu Costa [48], which comprises the Nothobranchiidae + Aplocheilidae as used in later studies.

1. Apomorphic characters of †*Kenyaichthys* shared with the Aplocheilidae sensu Costa [48] are an expanded ventral process of the anguloarticular (Fig 4A1–4A2) and (probably) a supracleithrum fused to the post-temporal (Fig 6A1–6A2). On the other hand, †*Kenyaichthys* lacks the medially curved premaxillary ascending process that is also diagnostic for this group (this process is flat in †*Kenyaichthys*, see Fig 4C).
2. †*Kenyaichthys* does not display the two osteological autapomorphies for the Nothobranchiidae, i.e. bifid epipleural ribs (rod-shaped in †*Kenyaichthys*, Figs 3A6 and 4D3) and a keel-shaped lateral process on the middle part of the terminal centrum (smooth in †*Kenyaichthys*, Figs 2B1–2, 9, 10 and 11).
3. †*Kenyaichthys* displays three derived characters of the Nothobranchiidae and Rivulidae, i.e. a probably twisted and reduced lacrimal, a distinctive neural spine on the first vertebra (Fig 4D1–4D4), and a dorsal fin with one or two short rudimentary rays in front of the first long ray (Fig 7A1–7D2). A further synapomorphy for the Nothobranchiidae and Rivulidae defined by Costa [1, 50] is a narrow haemal spine of PU2, which is only slightly wider than the haemal spines of PU4 and PU5 (vs. distinctively wider in Aplocheilidae and Cyprinodontoidei). However, the phylogenetic value of this character remains to be explored in more detail, because the studied extant species of the Cyprinodontoidei, Aplocheilidae and Nothobranchiidae show a large degree of overlap in the respective ratios (see above).
4. †*Kenyaichthys* shares one autapomorphy with the Rivulidae, i.e. a premaxilla with a straight posterior border, whereas other characters clearly do not display the apomorphic state of the Rivulidae (Table 7). These comprise the broad coronoid process of the dentary (Fig 3A4–3A5) vs. a narrow coronoid process in Rivulidae; the ventral tip of the autopalatinum, which is long and extends to the quadratum (Figs 3A2–3A3 and 4A1–4A2) vs. shortened and not reaching the quadratum in Rivulidae; the robust and approximately L-shaped preoperculum (Fig 4A1–4A2) vs. thin and C-shaped in Rivulidae; and the lack of reduction in the lateral rim of the frontal (Fig 4B) vs. reduced in Rivulidae. In addition, it is possible that the branchiostegal and opercular membranes were separated in †*Kenyaichthys* (vs. united in Rivulidae) because †*Kenyaichthys* does not display scales on the branchiostegal rays (Fig 4A1), whereas continuous squamation on the ventral side of the head would be expected if the two membranes were united ([3] page 376).

Clearly, †*Kenyaichthys* possesses a combination of apomorphic characters that is not diagnostic for any of the extant aplocheiloid families. The possession of one or two short dorsal fin rays in front of the first long ray indicates that †*Kenyaichthys* is nearer to the Nothobranchiidae and Rivulidae than to any other extant family, which is supported by the phylogenetic analysis (Figs 14 and 15). Notably, and in contrast to our expectation, the phylogenetic tree places †*Kenyaichthys* closer to the Rivulidae, which represents a purely Neotropical group, than to the Aplocheilidae or Nothobranchiidae, which are widespread on the African continent (and on Madagascar and in India). This is probably due to the mutual possession of the distal portion of the premaxilla with a straight posterior border (in Rivulidae and †*Kenyaichthys*), the presence of a single mutual synapomorphy with the Aplocheilidae + Nothobranchiidae (= Aplocheilidae sensu [48]), and the lack of shared synapomorphies with the Nothobranchiidae or Aplocheilidae alone (see Table 7). We therefore consider the sister relationship of †*Kenyaichthys* and Rivulidae suggested by the phylogenetic analysis to be biased, due to the lack of equally available synapomorphies for the Rivulidae, Nothobranchiidae and Aplocheilidae. Additional apomorphies for the Aplocheilidae and Nothobranchiidae, found in future work, may well shift the phylogenetic position of †*Kenyaichthys* towards these two families, as would be expected based on their present biogeography on the African continent, Madagascar and India. On the other hand, the hitherto complete lack of information on the aplocheiloid fossil record may mean that current phylogenetic reconstructions are misleading in some respects. An alternative explanation for the observed tree topology is that the premaxilla character described above is not an apomorphy for the Rivulidae, but was in the past shared with lineages of aplocheiloids that are no longer extant.

### Polymorphism in †*Kenyaichthys*

†*Kenyaichthys* reveals a remarkable degree of polymorphism with regard to the character states of the parhypural, the arrangement of the proximal radials in the dorsal fin and the size dimensions of the hypural plate.

As described above, extant species of killifish can show considerable variation in their hypural plate dimensions (Table 4 and S7 Table). In contrast to the recent species, where the hypural plates are at least 4% of SL in length and 5% of SL in width, the here described fossils show smaller and sometimes also very tiny hypural plates (<1.0% of SL, n = 8), which is a condition not found in any of the examined extant killifish specimens. In conclusion, the great size variation of the hypural plate in †*K*. *kipkechi* is higher compared to those of the extant species and may hint to the presence of more than one species, which, however, could not be confirmed based on other characters.

Two conditions of the proximal part of the parhypural are known. The plesiomorphic state is a parhypural that overlaps the terminal centrum and displays a well-developed hypurapophysis; this is the condition seen in the Aplocheilidae *sensu strictu* and in most cyprinodontoid families [1, 50]. The apomorphic state is a reduced parhypural that does not overlap with the terminal centrum and possesses a rudimentary hypurapophysis at most [1, 50]; this is the condition found in the Nothobranchiidae and Rivulidae, and also in the Cyprinodontidae, some Fundulidae, and most Goodeidae (all Cyprinodontoidei) [1, 50]. Among the specimens of †*Kenyaichthys kipkechi*, some specimens show a reduced parhypural (Figs 2B1–2B2 and 9), while others have a parhypural that overlaps the terminal centrum (Fig 11); the hypurapophysis is usually absent. Such intraspecific polymorphism of the parhypural character state has not previously been reported for any extant or fossil species of killifish.

In addition, most specimens of †*Kenyaichthys* reveal a parhypural, which is autogenous, but in some specimens of †*K*. *kipkechi* the parhypural is fused to the ventral portion of the hypural plate to a variable extent (S6 Table).

A comparable polymorphism regarding the parhypural is found in the atheriniform species *Pseudomugil signifer*, which shows an autogenous parhypural in the majority of the individuals, but a parhypural fused to the ventral portion of the lower hypural plate in some specimens [62]. The character “parhypural fused to the lower hypural plate” is consistently present only in the Melanotaeniidae and in some members of the Bedotiidae (see [1, 3, 56, 62–74]), but it is difficult to discern the evolutionary state (apomorph or plesiomorph) of the character.

In the dorsal fins of killifishes, a single proximal radial (pterygiophore) generally supports each dorsal fin ray, but two proximal radials support the anteriormost long ray (regardless of whether preceding short rays are present or not). In some specimens of †*Kenyaichthys*, one or two short rays have been recognized, and the two proximal radials support the first long ray (Fig 7A1–7A2 and 7D1–7D2), as in Nothobranchiidae and Rivulidae. In other specimens, however, the first or second short dorsal fin ray is supported by two proximal radials (Fig 7B1–7B2 and 7C1–7C2). This condition is not known from any extant killifish species.

### The species concept used for †*Kenyaichthys*

We found a distinct overlap between the meristic values of the described species, and meristic characters alone were not useful for species diagnosis. The high level of variation in meristic characters might be related to sexual dimorphism, as sexual dimorphism is usually present in killifishes [3]. For example, in some rivulid species (*Austrolebias* and *Cynolebias*), the males possess more rays in anal and dorsal fins than the females [75–79].

Furthermore, we did not use differences in numbers of preural vertebrae to discriminate between species because this number can vary within a single species (this study and unpublished data of W. Costa, pers. communication, May 2013). While Costa [1] assumed that cyprinodontiform species possess four to six preural vertebrae, our data derived from the four species of *Aphanius* indicate that the number may be as low as three in some specimens (S7 Table).

Also the hypural plate length and width were not considered as taxonomically meaningful characters for species discrimination in †*Kenyaichthys*, because intraspecific variation in hypural plate dimensions was also found in the examined extant killifish specimens and, furthermore, because the four closely related studied species of *Aphanius* displayed overlap in their hypural plate lengths and widths. A part of the variation seen within the individual hypural plate dimensions may perhaps reflect sexual dimorphism since the males of the four species of *Aphanius* studied here showed higher mean values for the length and width of the hypural plates than the females. Such a sexual dimorphism in the hypural plate size could result from different swimming activity in females and males, because aggressive behaviour of males during courtship is known for *Aphanius* and several other killifish species [80–83]. A larger hypural plate probably helps to create a more effective tail strike during “tail beating” behaviours of territorial males, as in *Fundulus waccamensis* [84] or *Cyprinodon macularius* [85]. At the same time, a bigger hypural plate might impair the swimming performance of the males, because of higher drag, as in *Poecilia reticulata* [86].

### Taxonomic implications: Does †*Kenyaichthys* represent a species flock?

One possible explanation for the huge intraspecific variation seen in †*K*. *kipkechi* is that †*K*. *kipkechi* may contain several species “*in statu nascendi*” [87] or might represent a species flock. The differentiation between a species *in statu nascendi* and a “real” species is based on the degree of sexual isolation; species *in statu nascendi* are located between complete panmixis and complete sexual isolation. Examples include the *Aphanius anatoliae* and the *Cyprinodon variegatus* group [88], the individual populations of which are easily distinguishable in their external morphology, but reveal a gradient in their degree of hybrid sterility and sexual isolation. A species flock, on the other hand, is a monophyletic group of closely related species coexisting in the same area [89, 90] such as the species of *Cyprinodon* in Laguna Chichancanab in Mexico [91], or the littoral species of *Orestias* in Lake Titicaca in Peru [92, 93]. The only fossil species flock known from Africa is the cichlid species flock of *Mahengechromis* from the Eocene lake Mahenge [94].

We consider the concept of the species flock to be quite applicable to †*Kenyaichthys*. The species of the modern *Orestias*- and *Cyprinodon* species flocks, like the fossil species studied here, show a high level of overlap in their meristics and morphometrics [93, 95]. In the case of the *Cyprinodon* species flock, a single species (*C*. *maya*) was found to be sexually isolated, whereas the remainder exhibited different grades of hybridization and represent different evolutionary stages [91]. Horstkotte and Strecker [91] assumed that the flock evolved due to adaptive radiation because of trophic differentiation and in the absence of competitors. A further report with similar implications is the study on Nicaraguan Midas cichlids (*Amphilophus* cf. *citrinellus*) from the Crater Lake Apoyo [96]. The authors identified six species with different levels of reproductive isolation and interpreted them as a species flock *in statu nascendi*. Based on the level of overlap in morphometric and meristic characters we assume that the assemblage of the specimens of †*Kenyaichthys* also represents a species flock *in statu nascendi*.

### Environmental implications

Previous paleoenvironmental reconstructions for the Lukeino area suggest freshwater conditions and no environmental disturbances [38, 42]. However, the scarcity of typical freshwater fishes such as cyprinids in our samples indicates that some environmental factors prohibited the presence of other fish species. The most likely explanation is that seasonal aridity, which has been reported for the Late Miocene of Eastern Africa based on (amongst others) palynological and paleobotanical remains and changes in herbivorous mammal diversity [97, 98], increased the salinity from time to time, and may eventually have led to episodes of drought. Only extremely euryhaline and eurytherm fish species that are capable of producing eggs that are resistant to drying can survive such adverse periods. Indeed, some genera of the Nothobranchiidae and Rivulidae provide modern examples of such species [29], and perhaps some fossil groups of killifishes, maybe even the †Kenyaichthyidae discussed here, possessed such survival skills.

It appears that †*Kenyaichthys* was well adapted to its environment. This is additionally supported by the relatively low incidence of supernumerary spines (25% of 127 specimens of †*Kenyaichthys*, in which this character could be examined) in the caudal skeleton, which is comparable to that seen in hatchery-reared fish species (23%) [99], but higher compared to species living in pristine natural environments (12%) [100]. In the case of environmental pressures, a relatively higher percentage of caudal skeletons with supernumerary spines would be likely. This phenomenon is usually explained by the fusion of two vertebral centra owing to unfavourable conditions such as vitamin C deficiency, excess supply of vitamin A, or parasite infection [101–105].

An additional hint to some environmental disturbances is the hunchback curvature of the vertebral column in 50% of †*Kenyaichthys*, as described above. This abnormality can be provoked by elevated concentration of heavy metals such as cadmium, copper and zinc or significant variations of environmental parameters such as temperature [106–110]. The percentage of specimens of †*Kenyaichthys*, in which such a vertebral deformity is present, is more than three times higher compared to reports on spinal deformities in polluted waters (15.63–17.67% polluted vs. 1.96–4.58% non-polluted [109,110]). However, the extent of the hunchback seen in the fossils (Fig 5A and 5C) is less extreme than seen in extant specimens (see [109, 110] pages 363 and 554).

Spinal deformations in extant specimens are usually explained by the adverse influence of zinc and copper, which impair the neuromuscular system [110]. Moreover cadmium can disrupt the calcium metabolism, resulting in hypocalcaemia and destabilization of bones [111]. The natural source of zinc, copper and cadmium is weathering of rocks and soil [112], as well as volcanic emissions [113–114]. As the Lukeino area was influenced by volcanic activity [115], the most likely explanation for the vertebral deformations in †*Kenyaichthys* is that the aquatic environment was in close proximity to an active volcano delivering ashes into the water.

### Biogeographic implications

The vicariance hypothesis and the dispersalism theory describe the evolutionary history of the killifishes in two different ways. According to the vicariance hypothesis, the Cyprinodontiformes could be found on the whole continent of Gondwana during the Cretaceous, and their present-day distribution is mainly due to the later break-up of the super-continent [6, 29]. In contrast to this, the dispersalists argue that the Neotropics bear the highest generic diversity and therefore should be taken as the centre of origin of all Cyprinodontiformes, from where they spread out during the middle or late Cretaceous by crossing marine waters, as most cyprinodontoids and some aplocheiloids are considered to be secondary freshwater fish [28, 31]. However, there is no evidence for fossil Aplocheiloidei prior to †*Kenyaichthys* (Late Miocene), whereas the Cyprinodontoidei have a good fossil record since the Paleocene. †*Kenyaichthys* currently is the only and oldest representative of a fossil Aplocheiloidei, but future findings of fossil Aplocheiloidei are necessary to understand whether the vicariance or the dispersalism model provide a reliable scenario for the evolutionary history of the killifishes.

## Conclusion

The here studied fish fossils from the Upper Miocene Lukeino Formation document the first appearance of representatives of the Aplocheiloidei in the fossil record, which we assign to †Kenyaichthyidae nov. fam. and †*Kenyaichthys* nov. gen. †*Kenyaichthys* shows remarkable polymorphism of the hypural plate dimensions, the parhypural and the dorsal fin pterygiophores and displays a combination of apomorphic characters that is not diagnostic for any of the extant aplocheiloid families. It appears that †*Kenyaichthys* was an annual fish that belonged to an ancient clade that was related to the present-day lineage of the Nothobranchiidae.

Patterns of variation in neural and haemal spine dimensions in the caudal vertebrae of †*Kenyaichthys* and the extant species studied here indicate that previously described synapomorphies for the Cyprinodontoidei (i.e. “neural and haemal spines of PU3 wider than spines of preural vertebrae anterior to PU4 vs. about equal”) and the Nothobranchiidae+Rivulidae (i.e. “haemal spine of PU2 slightly wider than haemal spines of PU4 and PU5 vs. distinctively wider”) need to be revised.

The here described new species †*Kenyaichthys kipkechi* most likely represents an ancient killifish species flock *in statu nascendi*. This indicates that species flocks in the fossil record, which have only rarely been recognized in previous work, may not be as exceptional as previously thought. Such knowledge is essential for a better understanding of the species diversity in the fossil record.

The presence of a killifish assemblage in the Lukeino Formation, while typical freshwater fish are extremely rare, is not in conflict with the reconstruction of the Lukeino area as a freshwater-dominated environment, but indicates an environment in the Late Miocene of Eastern Africa that was influenced by seasonal aridity.

## Supporting Information

S1 Table Morphometric characters of †*Kenyaichthys kipkechi* sp. nov., given in mm (top) and in % of SL (below). (DOC)

S2 Table Measurements of the premaxilla and maxilla of †*Kenyaichthys kipkechi* sp. nov. (DOC)

S3 Table Meristic values of all specimens of †*Kenyaichthys* gen. et sp. nov. (DOC)

S4 Table Spine-ratios of †*Kenyaichthys* gen. et sp. nov. (DOC)

S5 Table Dimensions of key scales of four specimens of †*Kenyaichthys* gen. et sp. nov. (DOC)

S6 Table Polymorph characters of †*Kenyaichthys* gen. et sp. nov. and detectability of the neural spine on the first vertebra (NS 1). (DOC)

S7 Table Hypural plate length (lH) and width (wH), and numbers of preural vertebrae obtained from the extant cyprinodontoid and aplocheiloid specimens used for comparison. (DOC)

S8 Table Spine-ratios of the recent cyprinodontoid and aplocheiloid specimens used in this study. (DOC)

S9 Table Description of characters used for phylogenetic analysis, and distribution of character states. Characters are compiled from the following literature: [1], [48], [49], [50], and [55].
(DOC)

S10 Table Character-taxon matrix used in the phylogenetic analysis shown in Figs 14 and 15 based on 72 characters of 13 terminal taxa and two outgroups. (DOC)

S1 NEXUS File (NEX)

S1 Fig Measurements of spines. **A** on a rounded tip; **B** on a blunt tip; **C** on spines with tip not covered by caudal fin rays and on spine covered by fin rays.
(TIF)
