# Supplementary material for: †Kenyaichthyidae fam. nov. and †Kenyaichthys gen. nov. – First Record of a Fossil Aplocheiloid Killifish (Teleostei, Cyprinodontiformes)
Source: PLoS One. 2015 Apr 29;10(4):e0123056. doi: 10.1371/journal.pone.0123056 (PMC4414574; doi:10.1371/journal.pone.0123056)
Supplement: S1 Table — (DOC) [file pone.0123056.s001.doc]

**S1 Table. Morphometric characters of †*Kenyaichthys kipkechi* sp. nov.** given in mm (top) and in % of SL (below).

| ID | TL | | SL (mm) | | lc/hc | | lc | | hc | | Or | | GO | | prO | | LD | | hD |
| --- | --- | --- | --- | --- | --- | --- | --- | --- | --- | --- | --- | --- | --- | --- | --- | --- | --- | --- | --- |
| 1142´04 | 24.10 | | 20.90 | | 1.42 | | 5.90 | | 4.16 | | 1.88 | | 1.66 | | 1.42 | | 2.94 | | 2.09 |
|  | 115.31 | |  | |  | | 28.23 | | 19.90 | | 9.00 | | 7.94 | | 6.79 | | 14.07 | | 10.00 |
| 1144´04 | 26.80 | | 23.60 | | 1.41 | | 7.90 | | 5.60 | | 2.06 | | 2.22 | | 2.09 | | 3.83 | | 2.32 |
|  | 113.56 | |  | |  | | 33.47 | | 23.73 | | 8.73 | | 9.41 | | 8.86 | | 16.23 | | 9.83 |
| 1146´04 | 26.90 | | 23.70 | | 1.44 | | 7.17 | | 4.97 | | 2.33 | | 2.15 | | 1.94 | | 3.80 | | 2.34 |
|  | 113.50 | |  | |  | | 30.25 | | 20.97 | | 9.83 | | 9.07 | | 8.19 | | 16.03 | | 9.87 |
| **1144/1146´04** | **26.90** | | **23.70** | | **1.41** | | **7.90** | | **5.60** | | **2.33** | | **2.22** | | **2.09** | | **3.83** | | **2.34** |
|  | **113.50** | |  | |  | | **33.33** | | **23.63** | | **9.83** | | **9.37** | | **8.82** | | **16.16** | | **9.87** |
| 1145´04 | 25.40 | | 22.10 | | 1.45 | | 7.37 | | 5.10 | | 2.11 | | 2.18 | | 2.22 | | 2.57 | | 1.46 |
|  | 114.93 | |  | |  | | 33.35 | | 23.08 | | 9.55 | | 9.86 | | 10.05 | | 11.63 | | 6.61 |
| 1147´04 | 24.90 | | 21.50 | | 1.46 | | 5.94 | | 4.06 | | 1.61 | | 1.75 | | 1.67 | | 2.57 | | 1.53 |
|  | 115.81 | |  | |  | | 27.63 | | 18.88 | | 7.49 | | 8.14 | | 7.77 | | 11.95 | | 7.12 |
| 1148(1)´04 | 30.10 | | 26.70 | | 1.54 | | 8.09 | | 5.26 | | 3.26 | | 1.98 | | 2.36 | | 3.83 | | 2.47 |
|  | 112.73 | |  | |  | | 30.30 | | 19.70 | | 12.21 | | 7.42 | | 8.84 | | 14.34 | | 9.25 |
| 1149´04 | 32.70 | | 28.70 | | 1.48 | | 8.67 | | 5.87 | | 2.34 | | 2.53 | | 2.62 | | 3.91 | | 3.04 |
|  | 113.94 | |  | |  | | 30.21 | | 20.45 | | 8.15 | | 8.82 | | 9.13 | | 13.62 | | 10.59 |
| 1150´04 | 31.00 | | 28.00 | | 1.60 | | 8.75 | | 5.48 | | 2.78 | | 2.11 | | 2.35 | | 3.82 | | 2.84 |
|  | 110.71 | |  | |  | | 31.25 | | 19.57 | | 9.93 | | 7.54 | | 8.39 | | 13.64 | | 10.14 |
| 1151´04 | 25.50 | | 23.10 | | – | | 6.29 | | – | | 1.80 | | 1.95 | | – | | 3.01 | | – |
|  | 110.39 | |  | |  | | 27.23 | | – | | 7.79 | | 8.44 | | – | | 13.03 | | – |
| 1152´04 | 26.40 | | 23.50 | | 1.33 | | 6.99 | | 5.25 | | 2.22 | | 1.80 | | 2.10 | | 3.42 | | – |
|  | 112.34 | |  | |  | | 29.74 | | 22.34 | | 9.45 | | 7.66 | | 8.94 | | 14.55 | | – |
| **1151/1152´04** | **26.40** | | **23.50** | | **1.33** | | **6.99** | | **5.25** | | **2.22** | | **1.95** | | **2.10** | | **3.42** | | – |
|  | | **112.34** | |  | |  | | **29.74** | | **22.34** | | **9.45** | | **8.30** | | **8.94** | | **14.55** | **–** |
| 1153´04 | | 25.60 | | 23.00 | | 1.53 | | 7.65 | | 4.99 | | 2.36 | | 2.07 | | 1.89 | | 2.58 | 1.81 |
|  | | 111.30 | |  | |  | | 33.26 | | 21.70 | | 10.26 | | 9.00 | | 8.22 | | 11.22 | 7.87 |
| 1154a´04 | | 31.80 | | 28.40 | | 1.35 | | 7.93 | | 5.89 | | 2.26 | | 2.48 | | 2.34 | | 2.81 | 1.83 |
|  | | 111.97 | |  | |  | | 27.92 | | 20.74 | | 7.96 | | 8.73 | | 8.24 | | 9.89 | 6.44 |
| 1154b´04 | | 31.70 | | 28.60 | | 1.57 | | 8.52 | | 5.44 | | 2.18 | | 2.61 | | 2.51 | | 2.76 | 2.23 |

S1 Table. (Continued)

|  | | 110.84 | |  | |  | | 29.79 | | 19.02 | | 7.62 | | 9.13 | | 8.78 | 9.65 | 7.80 |
| --- | --- | --- | --- | --- | --- | --- | --- | --- | --- | --- | --- | --- | --- | --- | --- | --- | --- | --- |
| **1154a/b´04** | | **31.80** | | **28.60** | | **1.45** | | **8.52** | | **5.89** | | **2.26** | | **2.61** | | **2.51** | **2.81** | **2.23** |
|  | | **111.19** | |  | |  | | **29.79** | | **20.59** | | **7.90** | | **9.13** | | **8.78** | **9.83** | **7.80** |
| 1155´04 | | 29.00 | | 26.50 | | 1.88 | | 8.26 | | 4.40 | | 2.30 | | 2.53 | | 2.51 | 3.15 | 3.00 |
|  | | 109.43 | |  | |  | | 31.17 | | 16.60 | | 8.68 | | 9.55 | | 9.47 | 11.89 | 11.32 |
| 1156´04 | | 28.90 | | 26.30 | | 1.51 | | 7.65 | | 5.08 | | 2.28 | | 2.24 | | 2.15 | – | – |
|  | | 109.89 | |  | |  | | 29.09 | | 19.32 | | 8.67 | | 8.52 | | 8.17 | – | – |
| 1157(1)´04 | | 31.60 | | 27.90 | | 1.52 | | 9.47 | | 6.21 | | 2.54 | | 2.27 | | 3.20 | 4.12 | 3.37 |
|  | | 113.26 | |  | |  | | 33.94 | | 22.26 | | 9.10 | | 8.14 | | 11.47 | 14.77 | 12.08 |
| 1158(1)´04 | | 30.80 | | 27.10 | | 1.46 | | 9.34 | | 6.38 | | 2.49 | | 2.42 | | 3.29 | 3.97 | 3.04 |
|  | | 113.65 | |  | |  | | 34.46 | | 23.54 | | 9.19 | | 8.93 | | 12.14 | 14.65 | 11.22 |
| **1157(1)/1158(1)´04** | | **31.60** | | **27.90** | | **1.48** | | **9.47** | | **6.38** | | **2.54** | | **2.42** | | **3.29** | **4.12** | **3.37** |
|  | | **113.26** | |  | |  | | **33.94** | | **22.87** | | **9.10** | | **8.67** | | **11.79** | **14.77** | **12.08** |
| 1159a(1)´04 | | 28.30 | | 24.90 | | – | | 7.36 | | – | | – | | 1.99 | | – | 3.67 | 1.53 |
|  | | 113.65 | |  | |  | | 29.56 | | – | | – | | 7.99 | | – | 14.74 | 6.14 |
| 1159b(1)´04 | | 28.20 | | 25.30 | | – | | 7.85 | | – | | 2.41 | | 2.31 | | 1.78 | 3.33 | 2.37 |
|  | | 111.46 | |  | |  | | 31.03 | | – | | 9.53 | | 9.13 | | 7.04 | 13.16 | 9.37 |
| **1159a(1)/b(1)´04** | | **28.30** | | **25.30** | | **–** | | **7.85** | | – | | **2.41** | | **2.31** | | **1.78** | **3.67** | **2.37** |
|  | | **111.86** | |  | |  | | **31.03** | | – | | **9.53** | | **9.13** | | **7.04** | **14.51** | **9.37** |
| 1159a(2)´04 | | 32.40 | | 28.60 | | 1.34 | | 9.01 | | 6.71 | | 3.15 | | 2.71 | | 2.31 | 3.96 | 2.99 |
|  | | 113.29 | |  | |  | | 31.50 | | 23.46 | | 11.01 | | 9.48 | | 8.08 | 13.85 | 10.45 |
| 1159b(2)´04 | | 32.50 | | 29.00 | | 1.34 | | 8.74 | | 6.50 | | 2.56 | | 2.74 | | 2.27 | 4.00 | 3.44 |
|  | | 112.07 | |  | |  | | 30.14 | | 22.41 | | 8.83 | | 9.45 | | 7.83 | 13.79 | 11.86 |
| **1159a(2)/b(2)´04** | | **32.50** | | **29.00** | | **1.34** | | **9.01** | | **6.71** | | **3.15** | | **2.74** | | **2.31** | **4.00** | **3.44** |
|  | **112.07** | |  | |  | | **31.07** | | **23.14** | | **10.86** | | **9.45** | | **7.97** | | **13.79** | **11.86** |
| 1160a´04 | 30.20 | | 26.60 | | 1.76 | | 7.87 | | 4.46 | | 2.33 | | 2.31 | | 2.26 | | 3.76 | 2.97 |
|  | 113.53 | |  | |  | | 29.59 | | 16.77 | | 8.76 | | 8.68 | | 8.50 | | 14.14 | 11.17 |
| 1161a´04 | 29.50 | | 26.20 | | 1.66 | | 8.37 | | 5.04 | | 2.04 | | 2.03 | | 2.44 | | 4.23 | 2.76 |
|  | 112.60 | |  | |  | | 31.95 | | 19.24 | | 7.79 | | 7.75 | | 9.31 | | 16.15 | 10.53 |
| **1160a/61a´04** | **30.20** | | **26.60** | | **1.66** | | **8.37** | | **5.04** | | **2.33** | | **2.31** | | **2.44** | | **4.23** | **2.97** |

S1 Table. (Continued)

|  | **113.53** |  |  | **31.47** | **18.95** | **8.76** | **8.68** | **9.17** | **15.90** | **11.17** |
| --- | --- | --- | --- | --- | --- | --- | --- | --- | --- | --- |
| 1160b´04 | 31.70 | 27.40 | 1.63 | 8.06 | 4.94 | 2.59 | 2.39 | 1.96 | 2.81 | 2.43 |
|  | 115.69 |  |  | 29.42 | 18.03 | 9.45 | 8.72 | 7.15 | 10.26 | 8.87 |
| 1161b´04 | 31.70 | 27.70 | 1.77 | 8.02 | 4.52 | 2.49 | 2.55 | 2.18 | 2.64 | 2.39 |
|  | 114.44 |  |  | 28.95 | 16.32 | 8.99 | 9.21 | 7.87 | 9.53 | 8.63 |
| **1160b/1161b´04** | **31.70** | **27.70** | **1.63** | **8.06** | **4.94** | **2.59** | **2.55** | **2.18** | **2.81** | **2.43** |
|  | **114.44** |  |  | **29.10** | **17.83** | **9.35** | **9.21** | **7.87** | **10.14** | **8.77** |
| 1162´04 | 36.70 | 32.60 | 1.63 | 10.10 | 6.19 | 2.31 | 2.80 | 3.21 | 4.80 | 3.25 |
|  | 112.58 |  |  | 30.98 | 18.99 | 7.09 | 8.59 | 9.85 | 14.72 | 9.97 |
| 1163a(1)´04 | – | 25.50 | 1.37 | 6.93 | 5.07 | 2.19 | 2.32 | 1.59 | 2.86 | – |
|  |  |  |  | 27.18 | 19.88 | 8.59 | 9.10 | 6.24 | 11.22 | – |
| 1163b(2)´04 | – | 25.70 | 1.62 | 6.89 | 4.25 | 2.18 | 2.14 | 1.58 | 2.64 | – |
|  |  |  |  | 26.81 | 16.54 | 8.48 | 8.33 | 6.15 | 10.27 | – |
| **1163a(1)/b(2)´04** | **–** | **25.70** | **1.37** | **6.93** | **5.07** | **2.19** | **2.32** | **1.59** | **2.86** | **–** |
|  |  |  |  | **26.96** | **19.73** | **8.52** | **9.03** | **6.19** | **11.13** | **–** |
| 1163a(2)´04 | 27.20 | 24.20 | 1.57 | 6.84 | 4.36 | 1.96 | 1.61 | 2.41 | 3.38 | 2.94 |
|  | 112.40 |  |  | 28.26 | 18.02 | 8.10 | 6.65 | 9.96 | 13.97 | 12.15 |
| 1163b(1)´04 | 27.00 | 24.10 | 1.57 | 7.42 | 4.72 | 2.30 | 2.17 | 2.45 | 3.34 | 2.43 |
|  | 112.03 |  |  | 30.79 | 19.59 | 9.54 | 9.00 | 10.17 | 13.86 | 10.08 |
| **1163a(2)/b(1)´04** | **27.20** | **24.20** | **1.57** | **7.42** | **4.72** | **2.30** | **2.17** | **2.45** | **3.38** | **2.94** |
|  | **112.40** |  |  | **30.66** | **19.50** | **9.50** | **8.97** | **10.12** | **13.97** | **12.15** |
| 1164a´04 | 36.40 | 32.40 | 1.71 | 10.85 | 6.35 | 2.92 | 3.34 | 3.89 | – | – |
|  | 112.35 |  |  | 33.49 | 19.60 | 9.01 | 10.31 | 12.01 | – | – |
| 1164b´04 | – | – | – | – | – | – | – | – | – | – |
|  |  |  |  | – | – | – | – | – | – | – |
| **1164a/b´04** | **36.40** | **32.40** | **1.71** | **10.85** | **6.35** | **2.92** | **3.34** | **3.89** | **–** | – |
|  | **112.35** |  |  | **33.49** | **19.60** | **9.01** | **10.31** | **12.01** | – | – |
| 1165a´04 | 34.30 | 30.40 | 1.51 | 9.90 | 6.54 | 2.59 | 2.77 | 2.89 | 4.89 | 3.43 |
|  | 112.83 |  |  | 32.57 | 21.51 | 8.52 | 9.11 | 9.51 | 16.09 | 11.28 |
| 1165b´04 | 33.90 | 29.90 | 1.39 | 9.79 | 7.05 | 2.35 | 2.12 | 3.24 | 4.63 | 3.61 |

S1 Table. (Continued)

|  | 113.38 |  |  | 32.74 | 23.58 | 7.86 | 7.09 | 10.84 | 15.48 | 12.07 |
| --- | --- | --- | --- | --- | --- | --- | --- | --- | --- | --- |
| **1165a/b´04** | **34.30** | **30.40** | **1.40** | **9.90** | **7.05** | **2.59** | **2.77** | **3.24** | **4.89** | **3.61** |
|  | **112.83** |  |  | **32.57** | **23.19** | **8.52** | **9.11** | **10.66** | **16.09** | **11.88** |
| 1166a´04 | 24.30 | 21.10 | 1.44 | 5.73 | 3.98 | 1.79 | 1.55 | 1.41 | 2.70 | 3.13 |
|  | 115.17 |  |  | 27.16 | 18.86 | 8.48 | 7.35 | 6.68 | 12.80 | 14.83 |
| 1166b´04 | 29.10 | 26.20 | 1.45 | 7.34 | 5.05 | 2.17 | 2.44 | 1.45 | 3.18 | 2.46 |
|  | 111.07 |  |  | 28.02 | 19.27 | 8.28 | 9.31 | 5.53 | 12.14 | 9.39 |
| 1167´04 | 30.40 | 27.20 | – | 8.67 | – | 2.54 | 2.69 | 2.33 | 4.18 | 2.81 |
|  | 111.76 |  |  | 31.88 | – | 9.34 | 9.89 | 8.57 | 15.37 | 10.33 |
| 1168´04 | 25.50 | 22.00 | 1.27 | 5.82 | 4.59 | 1.41 | 1.76 | 1.65 | 2.97 | 2.37 |
|  | 115.91 |  |  | 26.45 | 20.86 | 6.41 | 8.00 | 7.50 | 13.50 | 10.77 |
| 1170´04 | 27.60 | 24.00 | – | 7.50 | – | 1.91 | 2.39 | 2.21 | 3.67 | 2.70 |
|  | 115.00 |  |  | 31.25 | – | 7.96 | 9.96 | 9.21 | 15.29 | 11.25 |
| 1171´04 | 30.30 | 27.60 | 1.58 | 7.49 | 4.73 | 1.76 | 2.21 | 2.40 | 3.25 | 2.65 |
|  | 109.78 |  |  | 27.14 | 17.14 | 6.38 | 8.01 | 8.70 | 11.78 | 9.60 |
| 1172´04 | 30.80 | 27.20 | 1.48 | 7.78 | 5.26 | 2.19 | 2.21 | 1.88 | 2.68 | 2.76 |
|  | 113.24 |  |  | 28.60 | 19.34 | 8.05 | 8.13 | 6.91 | 9.85 | 10.15 |
| 1174´04 | 33.70 | 29.30 | 1.73 | 8.34 | 4.82 | 2.57 | 2.11 | 2.24 | 4.02 | 2.95 |
|  | 115.02 |  |  | 28.46 | 16.45 | 8.77 | 7.20 | 7.65 | 13.72 | 10.07 |
| 1175´04 | 29.50 | 26.10 | 1.38 | 8.16 | 5.93 | 2.32 | 2.51 | 2.26 | 3.86 | 2.68 |
|  | 113.03 |  |  | 31.26 | 22.72 | 8.89 | 9.62 | 8.66 | 14.79 | 10.27 |
| 1176a´04 | 27.70 | 24.90 | – | 7.35 | – | 2.43 | 2.24 | 1.49 | 3.02 | 2.15 |
|  | 111.24 |  |  | 29.52 | – | 9.76 | 9.00 | 5.98 | 12.13 | 8.63 |
| 1176b´04 | 28.10 | 25.60 | 1.30 | 8.13 | 6.25 | 2.05 | 2.31 | 2.26 | 3.01 | 2.36 |
|  | 109.77 |  |  | 31.76 | 24.41 | 8.01 | 9.02 | 8.83 | 11.76 | 9.22 |
| **1176a/b´04** | **28.10** | **25.60** | **1.30** | **8.13** | **6.25** | **2.43** | **2.31** | **2.26** | **3.02** | **2.36** |
|  | **109.77** |  |  | **31.76** | **24.41** | **9.49** | **9.02** | **8.83** | **11.80** | **9.22** |
| 1177´04 | 28.90 | 25.50 | 1.49 | 6.79 | 4.56 | 2.07 | 2.52 | 1.56 | 3.36 | 2.17 |
|  | 113.33 |  |  | 26.63 | 17.88 | 8.12 | 9.88 | 6.12 | 13.18 | 8.51 |
| 1178(1)´04 | 26.50 | 23.50 | 1.38 | 6.39 | 4.62 | 1.65 | 2.27 | 1.67 | 3.08 | – |

S1 Table. (Continued)

|  | 112.77 |  |  | 27.19 | 19.66 | 7.02 | 9.66 | 7.11 | 13.11 | – |
| --- | --- | --- | --- | --- | --- | --- | --- | --- | --- | --- |
| 1180(1)´04 | 26.50 | 24.00 | 1.66 | 7.47 | 4.49 | 1.95 | 1.65 | 2.55 | 3.26 | 2.44 |
|  | 110.42 |  |  | 31.13 | 18.71 | 8.13 | 6.88 | 10.63 | 13.58 | 10.17 |
| 1181(1)´04 | 34.90 | 30.30 | 1.56 | 8.43 | 5.40 | 2.75 | 2.06 | 2.92 | 4.37 | 3.26 |
|  | 115.18 |  |  | 27.82 | 17.82 | 9.08 | 6.80 | 9.64 | 14.42 | 10.76 |
| 1181(2)´04 | 34.40 | 30.40 | – | 6.77 | – | 1.85 | 1.92 | 2.05 | 3.42 | 2.76 |
|  | 113.16 |  |  | 22.27 | – | 6.09 | 6.32 | 6.74 | 11.25 | 9.08 |
| 1183(1)´04 | 34.80 | 30.90 | 1.56 | 8.37 | 5.36 | 2.48 | 2.43 | 2.10 | 3.66 | 2.94 |
|  | 112.62 |  |  | 27.09 | 17.35 | 8.03 | 7.86 | 6.80 | 11.84 | 9.51 |
| **1181(2)/1183(1)´04** | **34.80** | **30.90** | **1.56** | **8.37** | **5.36** | **2.48** | **2.43** | **2.10** | **3.66** | **2.94** |
|  | **112.62** |  |  | **27.09** | **17.35** | **8.03** | **7.86** | **6.80** | **11.84** | **9.51** |
| 1182´04 | 31.10 | 27.30 | 1.30 | 8.13 | 6.27 | 2.63 | 1.95 | 2.01 | 4.37 | 2.82 |
|  | 113.92 |  |  | 29.78 | 22.97 | 9.63 | 7.14 | 7.36 | 16.01 | 10.33 |
| 1184(1)´04 | 28.70 | 25.80 | – | 6.92 | – | – | – | – | – | – |
|  | 111.24 |  |  | 26.82 | – | – | – | – | – | – |
| 1185´04 | 31.30 | 27.50 | – | 7.71 | – | 2.12 | 2.11 | 1.75 | 3.46 | 3.45 |
|  | 113.82 |  |  | 28.04 | – | 7.71 | 7.67 | 6.36 | 12.58 | 12.55 |
| 1186´04 | 31.60 | 27.80 | 1.84 | 7.86 | 4.27 | 2.46 | 2.07 | 2.27 | 4.25 | 3.23 |
|  | 113.67 |  |  | 28.27 | 15.36 | 8.85 | 7.45 | 8.17 | 15.29 | 11.62 |
| **1185/1186´04** | **31.80** | **28.10** | **1.84** | **7.86** | **4.27** | **2.46** | **2.11** | **2.27** | **4.25** | **3.45** |
|  | **113.67** |  |  | **28.27** | **15.36** | **8.85** | **7.59** | **8.17** | **15.29** | **12.41** |
| 1187´04 | 31.10 | 27.20 | 1.56 | 7.76 | 4.98 | 2.63 | 2.22 | 2.50 | 3.65 | 3.02 |
|  | 114.34 |  |  | 28.53 | 18.31 | 9.67 | 8.16 | 9.19 | 13.42 | 11.10 |
| 1188´04 | – | 28.40 | 1.71 | 8.06 | 4.71 | 2.91 | 2.44 | 1.89 | 3.62 | – |
|  |  |  |  | 28.38 | 16.58 | 10.25 | 8.59 | 6.65 | 12.75 | – |
| 1189´04 | 27.10 | 24.40 | 1.74 | 6.45 | 3.70 | 1.86 | 2.04 | 1.55 | 2.69 | 2.25 |
|  | 111.07 |  |  | 26.43 | 15.16 | 7.62 | 8.36 | 6.35 | 11.02 | 9.22 |
| 1190´04 | 32.70 | 29.40 | 1.78 | 8.54 | 4.80 | 2.46 | 2.32 | 2.80 | 3.80 | – |
|  | 111.22 |  |  | 29.05 | 16.33 | 8.37 | 7.89 | 9.52 | 12.93 | – |
| 1192´04 | 32.20 | 28.20 | 1.56 | 7.06 | 4.52 | 2.02 | 2.32 | 1.89 | 3.42 | – |

S1 Table. (Continued)

|  | 114.18 |  |  | 25.04 | 16.03 | 7.16 | 8.23 | 6.70 | 12.13 | – |
| --- | --- | --- | --- | --- | --- | --- | --- | --- | --- | --- |
| 1192a´05 | 34.40 | 31.40 | 1.36 | 8.32 | 6.12 | 2.35 | 2.14 | 2.19 | – | – |
|  | 109.55 |  |  | 26.50 | 19.49 | 7.48 | 6.82 | 6.97 | – | – |
| 1192b´05 | 34.10 | 31.50 | 1.38 | 7.87 | 5.70 | 2.26 | 1.81 | 2.24 | – | – |
|  | 108.25 |  |  | 24.98 | 18.10 | 7.17 | 5.75 | 7.11 | – | – |
| **1192a/b´05** | **34.40** | **31.50** | **1.36** | **8.32** | **6.12** | **2.35** | **2.14** | **2.24** | – | – |
|  | **109.21** |  |  | **26.41** | **19.43** | **7.46** | **6.79** | **7.11** | – | – |
| 1193´04 | 29.70 | 27.10 | 1.54 | 7.04 | 4.57 | 1.99 | 2.17 | 1.59 | 3.60 | – |
|  | 109.59 |  |  | 25.98 | 16.86 | 7.34 | 8.01 | 5.87 | 13.28 | – |
| 1194´04 | 29.50 | 25.70 | 1.44 | 7.26 | 5.03 | 2.29 | 2.14 | 1.91 | 3.47 | 2.55 |
|  | 114.79 |  |  | 28.25 | 19.57 | 8.91 | 8.33 | 7.43 | 13.50 | 9.92 |
| 1198a´04 | 25.40 | 22.30 | 1.45 | 6.47 | 4.47 | 1.82 | 1.79 | 1.89 | 3.42 | 2.36 |
|  | 113.90 |  |  | 29.01 | 20.04 | 8.16 | 8.03 | 8.48 | 15.34 | 10.58 |
| 1198b´04 | 25.70 | 22.40 | 1.49 | 6.60 | 4.42 | 1.81 | 2.17 | 1.75 | 3.35 | 2.55 |
|  | 114.73 |  |  | 29.46 | 19.73 | 8.08 | 9.69 | 7.81 | 14.96 | 11.38 |
| **1198a/b´04** | **25.70** | **22.40** | **1.48** | **6.60** | **4.47** | **1.82** | **2.17** | **1.89** | **3.42** | **2.55** |
|  | **114.73** |  |  | **29.46** | **19.96** | **8.13** | **9.69** | **8.44** | **15.27** | **11.38** |
| 1199a´04 | 31.70 | 27.50 | 1.57 | 9.08 | 5.80 | 2.24 | 2.75 | 2.90 | 3.61 | 3.07 |
|  | 115.27 |  |  | 33.02 | 21.09 | 8.15 | 10.00 | 10.55 | 13.13 | 11.16 |
| 1199b´04 | 30.50 | 26.20 | 1.57 | 8.90 | 5.66 | 2.97 | 2.47 | 2.17 | 3.65 | 3.14 |
|  | 116.41 |  |  | 33.97 | 21.60 | 11.34 | 9.43 | 8.28 | 13.93 | 11.98 |
| **1199a/b´04** | **31.70** | **27.50** | **1.57** | **9.08** | **5.80** | **2.97** | **2.75** | **2.90** | **3.65** | **3.14** |
|  | **115.27** |  |  | **33.02** | **21.09** | **10.80** | **10.00** | **10.55** | **13.27** | **11.42** |
| 1200´04 | – | 31.10 | 1.66 | 8.72 | 5.24 | 2.76 | 2.54 | 2.03 | – | – |
|  |  |  |  | 28.04 | 16.85 | 8.87 | 8.17 | 6.53 | – | – |
| 1202´04 | 31.70 | 29.60 | 1.61 | 7.32 | 4.55 | – | 1.79 | – | 3.13 | – |
|  | 107.09 |  |  | 24.73 | 15.37 | – | 6.05 | – | 10.57 | – |
| 1203a´05 | 31.40 | 28.60 | 1.65 | 8.08 | 4.91 | 2.22 | 2.06 | 2.56 | – | 1.96 |
|  | 109.79 |  |  | 28.25 | 17.17 | 7.76 | 7.20 | 8.95 | – | 6.85 |
| 1203b´05 | 31.10 | 28.60 | 1.53 | 8.02 | 5.24 | 2.23 | 2.22 | 2.54 | 3.75 | 1.96 |

S1 Table. (Continued)

|  | 108.74 |  |  | 28.04 | 18.32 | 7.80 | 7.76 | 8.88 | 13.11 | 6.85 |
| --- | --- | --- | --- | --- | --- | --- | --- | --- | --- | --- |
| **1203a/b´05** | **31.40** | **28.60** | **1.54** | **8.08** | **5.24** | **2.23** | **2.22** | **2.56** | **3.75** | **1.96** |
|  | **109.79** |  |  | **28.25** | **18.32** | **7.80** | **7.76** | **8.95** | **13.11** | **6.85** |
| 1204´04 | 31.60 | 29.10 | 1.46 | 8.40 | 5.75 | 2.56 | 2.70 | 2.30 | 3.60 | 2.78 |
|  | 108.59 |  |  | 28.87 | 19.76 | 8.80 | 9.28 | 7.90 | 12.37 | 9.55 |
| 1204´05 | 22.30 | 20.40 | 1.65 | 5.95 | 3.60 | 1.53 | 1.36 | 1.84 | 2.49 | 1.25 |
|  | 109.31 |  |  | 29.17 | 17.65 | 7.50 | 6.67 | 9.02 | 12.21 | 6.13 |
| 1206(1)´04 | 29.00 | 25.40 | 1.39 | 7.29 | 5.26 | 1.94 | 2.22 | 1.88 | 3.55 | 2.69 |
|  | 114.17 |  |  | 28.70 | 20.71 | 7.64 | 8.74 | 7.40 | 13.98 | 10.59 |
| 1211´04 | 28.80 | 25.30 | 1.36 | 7.62 | 5.59 | 2.03 | 2.08 | 2.52 | 3.31 | 3.24 |
|  | 113.83 |  |  | 30.12 | 22.09 | 8.02 | 8.22 | 9.96 | 13.08 | 12.81 |
| **1206(1)/1211´04** | **29.00** | **25.40** | **1.36** | **7.62** | **5.59** | **2.03** | **2.22** | **2.52** | **3.55** | **3.24** |
|  | **114.17** |  |  | **30.00** | **22.01** | **7.99** | **8.74** | **9.92** | **13.98** | **12.76** |
| 1209´04 | 28.40 | 25.30 | – | 7.26 | – | – | 2.59 | – | – | – |
|  | 112.25 |  |  | 28.70 | – | – | 10.24 | – | – | – |
| 1209a´05 | 39.70 | 35.70 | 1.43 | 10.25 | 7.19 | 2.78 | 2.32 | 3.53 | 3.93 | – |
|  | 111.20 |  |  | 28.71 | 20.14 | 7.79 | 6.50 | 9.89 | 11.01 | – |
| 1209b´05 | 39.30 | 35.70 | 1.61 | 10.46 | 6.51 | 2.70 | 2.73 | 3.47 | 3.86 | – |
|  | 110.08 |  |  | 29.30 | 18.24 | 7.56 | 7.65 | 9.72 | 10.81 | – |
| **1209a/b´05** | **39.70** | **35.70** | **1.45** | **10.46** | **7.19** | **2.78** | **2.73** | **3.53** | **3.93** | – |
|  | **111.20** |  |  | **29.30** | **20.14** | **7.79** | **7.65** | **9.89** | **11.01** | – |
| 1213(1)´04 | 32.10 | 28.80 | 1.85 | 9.72 | 5.24 | 2.78 | 2.26 | 2.82 | 3.57 | 2.45 |
|  | 111.46 |  |  | 33.75 | 18.19 | 9.65 | 7.85 | 9.79 | 12.40 | 8.51 |
| 1215(1)´04 | 28.00 | 24.40 | 1.97 | 7.80 | 3.96 | 2.24 | 1.95 | 2.59 | 3.35 | 2.57 |
|  | 114.75 |  |  | 31.97 | 16.23 | 9.18 | 7.99 | 10.61 | 13.73 | 10.53 |
| 1217a(1)´04 | – | 26.50 | 1.71 | 7.43 | 4.35 | 1.95 | 2.36 | 1.07 | 3.18 | – |
|  |  |  |  | 28.04 | 16.42 | 7.36 | 8.91 | 4.04 | 12.00 | – |
| 1217b(1)´04 | – | – | – | – | – | – | – | – | – | – |
|  | – | – | – | – | – | – | – | – | – | – |
| **1217a(1)/b(1)´04** | **–** | **26.50** | **1.71** | **7.43** | **4.35** | **1.95** | **2.36** | **1.07** | **3.18** | – |

S1 Table. (Continued)

|  |  | |  | |  | | **28.04** | | **16.42** | | **7.36** | **8.91** | **4.04** | **12.00** | – |
| --- | --- | --- | --- | --- | --- | --- | --- | --- | --- | --- | --- | --- | --- | --- | --- |
| 1218´04 | 26.60 | | 23.90 | | 1.77 | | 6.99 | | 3.96 | | 1.92 | 2.06 | 2.16 | 3.02 | 3.05 |
|  | 111.30 | |  | |  | | 29.25 | | 16.57 | | 8.03 | 8.62 | 9.04 | 12.64 | 12.76 |
| 1218a´05 | 38.50 | | 33.40 | | – | | – | | – | | – | – | 3.68 | 4.08 | 2.84 |
|  | 115.27 | |  | |  | | – | | – | | – | – | 11.02 | 12.22 | 8.50 |
| 1218b´05 | 37.60 | | 32.90 | | – | | – | | – | | 1.91 | – | 3.38 | 4.36 | 2.87 |
|  | 114.29 | |  | |  | | – | | – | | 5.81 | – | 10.27 | 13.25 | 8.72 |
| **1218a/b´05** | **38.50** | | **33.40** | | **–** | | **–** | | **–** | | **1.91** | **–** | **3.68** | **4.36** | **2.87** |
|  | **115.27** | |  | |  | | **–** | | **–** | | **5.72** | **–** | **11.02** | **13.05** | **8.59** |
| 1219(1)´04 | 29.20 | | 25.60 | | – | | 7.98 | | – | | – | – | – | 3.13 | – |
|  | 114.06 | |  | |  | | 31.17 | | – | | – | – | – | 12.23 | – |
| 1220(1)´04 | 31.00 | | 27.20 | | 1.41 | | 7.42 | | 5.26 | | 2.63 | 1.92 | 1.83 | 3.69 | – |
|  | 113.97 | |  | |  | | 27.28 | | 19.34 | | 9.67 | 7.06 | 6.73 | 13.57 | – |
| 1220R´04 | 25.60 | | 23.10 | | 1.28 | | 6.88 | | 5.38 | | 2.02 | 1.87 | 2.00 | 3.09 | 2.09 |
|  | 110.82 | |  | |  | | 29.78 | | 23.29 | | 8.74 | 8.10 | 8.66 | 13.38 | 9.05 |
| 1221(1)´04 | 31.80 | | 29.10 | | 1.51 | | 9.39 | | 6.22 | | 2.60 | 2.94 | 2.65 | 3.57 | 2.53 |
|  | 109.28 | |  | |  | | 32.27 | | 21.37 | | 8.93 | 10.10 | 9.11 | 12.27 | 8.69 |
| 1227(1)´04 | 32.30 | | 28.40 | | 1.50 | | 7.79 | | 5.21 | | – | 2.05 | – | 4.06 | 3.31 |
|  | 113.73 | |  | |  | | 27.43 | | 18.35 | | – | 7.22 | – | 14.30 | 11.65 |
| 1228(1)´04 | 35.90 | | 31.30 | | 1.44 | | 9.75 | | 6.76 | | 2.36 | 2.87 | 3.00 | 4.26 | 3.63 |
|  | 114.70 | |  | |  | | 31.15 | | 21.60 | | 7.54 | 9.17 | 9.58 | 13.61 | 11.60 |
| 1237R(1)´04 | 35.70 | | 31.20 | | 1.57 | | 9.90 | | 6.30 | | 2.76 | 3.04 | 2.89 | 4.36 | 3.52 |
|  | 114.42 | |  | |  | | 31.73 | | 20.19 | | 8.85 | 9.74 | 9.26 | 13.97 | 11.28 |
| **1228(1)/1237R(1)´04** | **35.90** | | **31.30** | | **1.46** | | **9.90** | | **6.76** | | **2.76** | **3.04** | **3.00** | **4.36** | **3.63** |
|  | | **114.70** | |  | |  | | **31.63** | | **21.60** | **8.82** | **9.71** | **9.58** | **13.93** | **11.60** |
| 1233´04 | | 30.60 | | 27.50 | | 1.49 | | 8.62 | | 5.79 | 2.14 | 2.53 | 2.96 | – | – |
|  | | 111.27 | |  | |  | | 31.35 | | 21.05 | 7.78 | 9.20 | 10.76 | – | – |
| 1234(1)´04 | | 30.80 | | 27.70 | | 1.46 | | 8.99 | | 6.15 | 2.31 | 2.44 | 3.05 | 3.30 | 2.15 |
|  | | 111.19 | |  | |  | | 32.45 | | 22.20 | 8.34 | 8.81 | 11.01 | 11.91 | 7.76 |
| **1233/1234(1)´04** | | **30.80** | | **27.70** | | **1.46** | | **8.99** | | **6.15** | **2.14** | **2.53** | **3.05** | **3.30** | **2.15** |

S1 Table. (Continued)

|  | **111.19** | |  | |  | | **32.45** | | **22.20** | | **7.73** | | **9.13** | | **11.01** | | **11.91** | **7.76** |
| --- | --- | --- | --- | --- | --- | --- | --- | --- | --- | --- | --- | --- | --- | --- | --- | --- | --- | --- |
| 1234(2)´04 | 31.40 | | 28.40 | | 1.39 | | 9.11 | | 6.55 | | 2.56 | | 2.53 | | 3.17 | | 3.68 | 2.35 |
|  | 110.56 | |  | |  | | 32.08 | | 23.06 | | 9.01 | | 8.91 | | 11.16 | | 12.96 | 8.27 |
| 1234R´04 | 31.70 | | 28.30 | | 1.53 | | 9.13 | | 5.97 | | 2.31 | | 2.43 | | 2.80 | | 2.88 | 3.00 |
|  | 112.01 | |  | |  | | 32.26 | | 21.10 | | 8.16 | | 8.59 | | 9.89 | | 10.18 | 10.60 |
| 1236(1)´04 | 32.90 | | 28.90 | | – | | 8.25 | | – | | 2.24 | | 2.54 | | 2.19 | | 4.02 | 2.84 |
|  | 113.84 | |  | |  | | 28.55 | | – | | 7.75 | | 8.79 | | 7.58 | | 13.91 | 9.83 |
| 1237(1)´04 | 31.60 | | 28.00 | | 1.43 | | 8.35 | | 5.82 | | 2.54 | | 2.67 | | 2.49 | | 3.44 | 2.64 |
|  | 112.86 | |  | |  | | 29.82 | | 20.79 | | 9.07 | | 9.54 | | 8.89 | | 12.29 | 9.43 |
| 1237(2)´04 | 30.30 | | 27.50 | | 1.44 | | 9.16 | | 6.36 | | 2.68 | | 2.33 | | 3.42 | | 3.24 | 2.62 |
|  | 110.18 | |  | |  | | 33.31 | | 23.13 | | 9.75 | | 8.47 | | 12.44 | | 11.78 | 9.53 |
| 1324´04 | 31.60 | | 27.80 | | – | | 8.05 | | – | | 2.58 | | 2.74 | | 1.62 | | 4.01 | 2.88 |
|  | 113.67 | |  | |  | | 28.96 | | – | | 9.28 | | 9.86 | | 5.83 | | 14.42 | 10.36 |
| 1325´04 | 30.40 | | 26.90 | | 1.47 | | 7.36 | | 5.00 | | 2.22 | | 2.09 | | 2.09 | | – | – |
|  | 113.01 | |  | |  | | 27.36 | | 18.59 | | 8.25 | | 7.77 | | 7.77 | | – | – |
| ID | lA | | hA | | lP | | lPbs | | lV | | lVbs | | lC | | h | | H | pD |
| 1142´04 | 3.41 | | 2.36 | | 1.50 | | 0.60 | | – | | – | | 3.34 | | 1.62 | | 4.32 | 11.43 |
|  | 16.32 | | 11.29 | | 7.18 | | 2.87 | | – | | – | | 15.98 | | 7.75 | | 20.67 | 54.69 |
| 1144´04 | 4.52 | | 2.30 | | 2.51 | | – | | 1.12 | | 0.20 | | 3.41 | | 2.28 | | 6.11 | 13.38 |
|  | 19.15 | | 9.75 | | 10.64 | | – | | 4.75 | | 0.85 | | 14.45 | | 9.66 | | 25.89 | 56.69 |
| 1146´04 | 4.62 | | 2.21 | | 1.77 | | 1.00 | | 1.48 | | 0.37 | | 3.62 | | 2.48 | | 6.00 | 13.87 |
|  | 19.49 | | 9.32 | | 7.47 | | 4.22 | | 6.24 | | 1.56 | | 15.29 | | 10.46 | | 25.32 | 58.52 |
| **1144/1146´04** | **4.62** | | **2.30** | | **2.51** | | **1.00** | | **1.48** | | **0.37** | | **3.62** | | **2.48** | | **6.11** | **13.87** |
|  | **19.49** | | **9.70** | | **10.59** | | **4.22** | | **6.24** | | **1.56** | | **15.27** | | **10.46** | | **25.78** | **58.52** |
| 1145´04 | | 3.17 | | 1.36 | | 2.34 | | 0.59 | | 1.25 | | 0.38 | | 3.49 | | 1.88 | 4.36 | 13.74 |
|  | | 14.34 | | 6.15 | | 10.59 | | 2.67 | | 5.66 | | 1.72 | | 15.79 | | 8.51 | 19.73 | 62.17 |
| 1147´04 | | 3.36 | | 1.86 | | 1.09 | | 0.91 | | 0.79 | | 0.16 | | 3.56 | | 1.65 | 3.80 | 12.56 |
|  | | 15.63 | | 8.65 | | 5.07 | | 4.23 | | 3.67 | | 0.74 | | 16.56 | | 7.67 | 17.67 | 58.42 |
| 1148(1)´04 | | 4.06 | | 2.36 | | 2.23 | | 1.25 | | 1.11 | | 0.33 | | 3.79 | | 2.39 | 5.94 | 16.10 |
|  | | 15.21 | | 8.84 | | 8.35 | | 4.68 | | 4.16 | | 1.24 | | 14.19 | | 8.95 | 22.25 | 60.30 |

S1 Table. (Continued)

| 1149´04 | 4.44 | 2.84 | 2.94 | 0.88 | 1.63 | 0.44 | 4.19 | 2.61 | 6.67 | 17.43 |
| --- | --- | --- | --- | --- | --- | --- | --- | --- | --- | --- |
|  | 15.47 | 9.90 | 10.24 | 3.07 | 5.68 | 1.53 | 14.60 | 9.09 | 23.24 | 60.73 |
| 1150´04 | 4.43 | 2.37 | 2.35 | 1.04 | 1.20 | 0.19 | 3.57 | 2.48 | 5.79 | 16.10 |
|  | 15.82 | 8.46 | 8.39 | 3.71 | 4.29 | 0.68 | 12.75 | 8.86 | 20.68 | 57.50 |
| 1151´04 | 3.61 | 2.24 | 2.74 | 1.32 | 1.17 | 0.23 | 2.75 | 2.14 | 5.64 | 13.36 |
|  | 15.63 | 9.70 | 11.86 | 5.71 | 5.06 | 1.00 | 11.90 | 9.26 | 24.42 | 57.84 |
| 1152´04 | 3.83 | 2.35 | 2.34 | 1.02 | 1.36 | 0.20 | 3.01 | 2.15 | 5.69 | 12.98 |
|  | 16.30 | 10.00 | 9.96 | 4.34 | 5.79 | 0.85 | 12.81 | 9.15 | 24.21 | 55.23 |
| **1151/1152´04** | **3.83** | **2.35** | **2.74** | **1.32** | **1.36** | **0.23** | **3.01** | **2.15** | **5.69** | **13.36** |
|  | **16.30** | **10.00** | **11.66** | **5.62** | **5.79** | **0.98** | **12.81** | **9.15** | **24.21** | **56.85** |
| 1153´04 | 3.57 | 2.33 | – | 0.76 | 0.74 | 0.34 | 3.05 | 1.84 | 4.75 | 14.59 |
|  | 15.52 | 10.13 | – | 3.30 | 3.22 | 1.48 | 13.26 | 8.00 | 20.65 | 63.43 |
| 1154a´04 | 4.03 | – | 2.58 | 1.51 | 1.78 | 0.18 | 3.65 | – | 5.80 | 16.77 |
|  | 14.19 | – | 9.08 | 5.32 | 6.27 | 0.63 | 12.85 | – | 20.42 | 59.05 |
| 1154b´04 | 3.74 | – | 3.25 | 1.13 | 1.35 | 0.22 | 3.32 | 2.11 | 5.79 | 16.57 |
|  | 13.08 | – | 11.36 | 3.95 | 4.72 | 0.77 | 11.61 | 7.38 | 20.24 | 57.94 |
| **1154a/b´04** | **4.03** | – | **3.25** | **1.51** | **1.78** | **0.22** | **3.65** | **2.11** | **5.80** | **16.77** |
|  | **14.09** | – | **11.36** | **5.28** | **6.22** | **0.77** | **12.76** | **7.38** | **20.28** | **58.64** |
| 1155´04 | 3.89 | 2.60 | 3.01 | 1.23 | 1.32 | 0.29 | 3.57 | – | 5.01 | 16.20 |
|  | 14.68 | 9.81 | 11.36 | 4.64 | 4.98 | 1.09 | 13.47 | – | 18.91 | 61.13 |
| 1156´04 | – | – | 2.09 | 1.32 | 1.23 | 0.41 | 3.21 | – | 6.13 | 15.80 |
|  | – | – | 7.95 | 5.02 | 4.68 | 1.56 | 12.21 | – | 23.31 | 60.08 |
| 1157(1)´04 | 4.17 | 2.96 | 1.30 | 1.23 | 1.22 | 0.28 | 3.81 | 2.88 | 7.59 | 16.36 |
|  | 14.95 | 10.61 | 4.66 | 4.41 | 4.37 | 1.00 | 13.66 | 10.32 | 27.20 | 58.64 |
| 1158(1)´04 | 4.40 | 2.83 | 2.40 | 1.39 | 1.14 | 0.21 | 3.81 | 2.88 | 7.50 | 16.74 |
|  | 16.24 | 10.44 | 8.86 | 5.13 | 4.21 | 0.77 | 14.06 | 10.63 | 27.68 | 61.77 |
| **1157(1)/1158(1)´04** | **4.40** | **2.96** | **2.40** | **1.39** | **1.22** | **0.28** | **3.81** | **2.88** | **7.59** | **16.74** |
|  | **15.77** | **10.61** | **8.60** | **4.98** | **4.37** | **1.00** | **13.66** | **10.32** | **27.20** | **60.00** |
| 1159a(1)´04 | 3.91 | 2.86 | 1.33 | 1.41 | 1.44 | 0.28 | 3.40 | 2.28 | – | 14.76 |
|  | 15.70 | 11.49 | 5.34 | 5.66 | 5.78 | 1.12 | 13.65 | 9.16 | – | 59.28 |

S1 Table. (Continued)

| 1159b(1)´04 | – | – | 2.17 | – | – | – | 3.19 | 2.22 | – | 13.04 |
| --- | --- | --- | --- | --- | --- | --- | --- | --- | --- | --- |
|  | – | – | 8.58 | – | – | – | 12.61 | 8.77 | – | 51.54 |
| **1159a(1)/b(1)´04** | **3.91** | **2.86** | **2.17** | **1.41** | **1.44** | **0.28** | **3.40** | **2.28** | – | **14.76** |
|  | **15.45** | **11.30** | **8.58** | **5.57** | **5.69** | **1.11** | **13.44** | **9.01** | – | **58.34** |
| 1159a(2)´04 | 4.19 | 3.12 | 2.81 | 1.76 | 1.41 | 0.25 | 3.97 | 2.94 | 7.52 | 16.20 |
|  | 14.65 | 10.91 | 9.83 | 6.15 | 4.93 | 0.87 | 13.88 | 10.28 | 26.29 | 56.64 |
| 1159b(2)´04 | 4.03 | 2.98 | 2.92 | 1.60 | 1.58 | 0.28 | 3.96 | 2.76 | 7.47 | 16.29 |
|  | 13.90 | 10.28 | 10.07 | 5.52 | 5.45 | 0.97 | 13.66 | 9.52 | 25.76 | 56.17 |
| **1159a(2)/b(2)´04** | **4.19** | **3.12** | **2.92** | **1.76** | **1.58** | **0.28** | **3.97** | **2.94** | **7.52** | **16.29** |
|  | **14.45** | **10.76** | **10.07** | **6.07** | **5.45** | **0.97** | **13.69** | **10.14** | **25.93** | **56.17** |
| 1160a´04 | 3.64 | 2.67 | 2.25 | 0.86 | 1.23 | 0.24 | 3.67 | 2.31 | 5.11 | 15.19 |
|  | 13.68 | 10.04 | 8.46 | 3.23 | 4.62 | 0.90 | 13.80 | 8.68 | 19.21 | 57.11 |
| 1161a´04 | 3.82 | 2.58 | 2.49 | 0.93 | 1.01 | 0.22 | 3.51 | 2.39 | 5.15 | 15.36 |
|  | 14.58 | 9.85 | 9.50 | 3.55 | 3.85 | 0.84 | 13.40 | 9.12 | 19.66 | 58.63 |
| **1160a/61a´04** | **3.82** | **2.67** | **2.49** | **0.93** | **1.23** | **0.24** | **3.67** | **2.39** | **5.15** | **15.36** |
|  | **14.36** | **10.04** | **9.36** | **3.50** | **4.62** | **0.90** | **13.80** | **8.98** | **19.36** | **57.74** |
| 1160b´04 | 3.58 | 2.14 | 2.13 | 0.83 | 1.07 | 0.25 | 4.12 | 2.11 | 5.02 | 16.51 |
|  | 13.07 | 7.81 | 7.77 | 3.03 | 3.91 | 0.91 | 15.04 | 7.70 | 18.32 | 60.26 |
| 1161b´04 | 3.85 | 1.94 | 0.92 | 0.66 | 1.30 | 0.27 | 3.91 | 2.03 | 5.01 | 16.14 |
|  | 13.90 | 7.00 | 3.32 | 2.38 | 4.69 | 0.97 | 14.12 | 7.33 | 18.09 | 58.27 |
| **1160b/1161b´04** | **3.85** | **2.14** | **2.13** | **0.83** | **1.30** | **0.27** | **4.12** | **2.11** | **5.02** | **16.51** |
|  | **13.90** | **7.73** | **7.69** | **3.00** | **4.69** | **0.97** | **14.87** | **7.62** | **18.12** | **59.60** |
| 1162´04 | 5.65 | 3.13 | 2.67 | 1.56 | 0.95 | 0.25 | 4.18 | 3.01 | 5.83 | 19.10 |
|  | 17.33 | 9.60 | 8.19 | 4.79 | 2.91 | 0.77 | 12.82 | 9.23 | 17.88 | 58.59 |
| 1163a(1)´04 | 3.35 | – | 2.20 | 0.97 | 0.78 | 0.25 | – | 2.95 | 5.01 | 15.58 |
|  | 13.14 | – | 8.63 | 3.80 | 3.06 | 0.98 | – | 11.57 | 19.65 | 61.10 |
| 1163b(2)´04 | 3.42 | – | 2.75 | 0.98 | 0.91 | 0.24 | – | 2.41 | 5.34 | 15.51 |
|  | 13.31 | – | 10.70 | 3.81 | 3.54 | 0.93 | – | 9.38 | 20.78 | 60.35 |
| **1163a(1)/b(2)´04** | **3.42** | **–** | **2.75** | **0.98** | **0.91** | **0.25** | **–** | **2.95** | **5.34** | **15.58** |
|  | **13.31** | **–** | **10.70** | **3.81** | **3.54** | **0.97** | – | **11.48** | **20.78** | **60.62** |

S1 Table. (Continued)

| 1163a(2)´04 | 3.78 | 2.56 | 1.26 | 1.11 | 1.15 | 0.22 | 3.00 | 1.95 | 3.19 | 14.34 |
| --- | --- | --- | --- | --- | --- | --- | --- | --- | --- | --- |
|  | 15.62 | 10.58 | 5.21 | 4.59 | 4.75 | 0.91 | 12.40 | 8.06 | 13.18 | 59.26 |
| 1163b(1)´04 | 3.75 | 2.25 | 2.15 | 1.02 | 1.14 | 0.16 | 3.07 | 1.75 | 2.96 | 14.18 |
|  | 15.56 | 9.34 | 8.92 | 4.23 | 4.73 | 0.66 | 12.74 | 7.26 | 12.28 | 58.84 |
| **1163a(2)/b(1)´04** | **3.78** | **2.56** | **2.15** | **1.11** | **1.15** | **0.22** | **3.07** | **1.95** | **4.86** | **14.34** |
|  | **15.62** | **10.58** | **8.88** | **4.59** | **4.75** | **0.91** | **12.69** | **8.06** | **20.08** | **59.26** |
| 1164a´04 | 5.36 | 3.21 | 3.04 | 1.49 | 1.37 | 0.22 | 3.76 | – | 6.07 | – |
|  | 16.54 | 9.91 | 9.38 | 4.60 | 4.23 | 0.68 | 11.60 | – | 18.73 | – |
| 1164b´04 | – | – | – | – | – | – | – | – | – | – |
|  | – | – | – | – | – | – | – | – | – | – |
| **1164a/b´04** | **5.36** | **3.21** | **3.04** | **1.49** | **1.37** | **0.22** | **3.76** | – | **6.07** | – |
|  | **16.54** | **9.91** | **9.38** | **4.60** | **4.23** | **0.68** | **11.60** | – | **18.73** | – |
| 1165a´04 | 4.86 | 2.63 | 3.38 | 1.50 | 1.57 | 0.32 | 4.19 | 2.69 | 6.74 | 17.69 |
|  | 15.99 | 8.65 | 11.12 | 4.93 | 5.16 | 1.05 | 13.78 | 8.85 | 22.17 | 58.19 |
| 1165b´04 | 4.27 | 2.41 | 1.50 | 1.34 | 1.09 | 0.31 | 3.91 | 2.83 | 6.69 | 17.90 |
|  | 14.28 | 8.06 | 5.02 | 4.48 | 3.65 | 1.04 | 13.08 | 9.46 | 22.37 | 59.87 |
| **1165a/b´04** | **4.86** | **2.63** | **3.38** | **1.50** | **1.57** | **0.32** | **4.19** | **2.83** | **6.74** | **17.90** |
|  | **15.99** | **8.65** | **11.12** | **4.93** | **5.16** | **1.05** | **13.78** | **9.31** | **22.17** | **58.88** |
| 1166a´04 | 1.91 | 2.32 | 1.83 | 0.87 | 0.99 | 0.23 | 3.20 | 1.62 | 4.15 | 12.11 |
|  | 9.05 | 11.00 | 8.67 | 4.12 | 4.69 | 1.09 | 15.17 | 7.68 | 19.67 | 57.39 |
| 1166b´04 | 4.22 | 2.27 | 2.19 | 1.14 | 1.16 | 0.24 | 3.70 | 2.22 | 5.20 | 16.37 |
|  | 16.11 | 8.66 | 8.36 | 4.35 | 4.43 | 0.92 | 14.12 | 8.47 | 19.85 | 62.48 |
| 1167´04 | 3.80 | 2.49 | 2.67 | 1.18 | 1.29 | 0.24 | 3.50 | 2.76 | 6.54 | 15.95 |
|  | 13.97 | 9.15 | 9.82 | 4.34 | 4.74 | 0.88 | 12.87 | 10.15 | 24.04 | 58.64 |
| 1168´04 | 3.72 | 1.82 | 1.76 | 1.11 | 0.85 | 0.23 | 3.69 | 2.10 | 4.37 | 12.60 |
|  | 16.91 | 8.27 | 8.00 | 5.05 | 3.86 | 1.05 | 16.77 | 9.55 | 19.86 | 57.27 |
| 1170´04 | 4.66 | – | – | – | – | – | 3.57 | 2.55 | – | 13.69 |
|  | 19.42 | – | – | – | – | – | 14.88 | 10.63 | – | 57.04 |
| 1171´04 | 3.42 | – | – | – | 1.92 | 0.26 | – | 2.36 | 5.94 | 16.66 |
|  | 12.39 | – | – | – | 6.96 | 0.94 | – | 8.55 | 21.52 | 60.36 |

S1 Table. (Continued)

| 1172´04 | 3.99 | 2.10 | – | – | – | – | 3.20 | 2.24 | 6.43 | 13.61 |
| --- | --- | --- | --- | --- | --- | --- | --- | --- | --- | --- |
|  | 14.67 | 7.72 | – | – | – | – | 11.76 | 8.24 | 23.64 | 50.04 |
| 1174´04 | 4.61 | 2.06 | 1.78 | 1.15 | 1.26 | 0.14 | 4.35 | 2.15 | 5.70 | 16.24 |
|  | 15.73 | 7.03 | 6.08 | 3.92 | 4.30 | 0.48 | 14.85 | 7.34 | 19.45 | 55.43 |
| 1175´04 | 4.31 | 2.96 | 2.28 | 1.12 | 1.20 | 0.21 | 3.25 | 3.04 | 6.19 | 15.38 |
|  | 16.51 | 11.34 | 8.74 | 4.29 | 4.60 | 0.80 | 12.45 | 11.65 | 23.72 | 58.93 |
| 1176a´04 | 4.19 | – | 2.66 | 0.79 | 1.47 | 0.21 | 2.72 | 1.93 | 4.90 | 15.07 |
|  | 16.83 | – | 10.68 | 3.17 | 5.90 | 0.84 | 10.92 | 7.75 | 19.68 | 60.52 |
| 1176b´04 | 3.62 | – | 2.96 | 0.58 | 1.30 | 0.19 | 3.50 | 1.97 | – | 14.85 |
|  | 14.14 | – | 11.56 | 2.27 | 5.08 | 0.74 | 13.67 | 7.70 | – | 58.01 |
| **1176a/b´04** | **4.19** | **–** | **2.96** | **0.79** | **1.47** | **0.21** | **3.50** | **1.97** | **4.90** | **15.07** |
|  | **16.37** | **–** | **11.56** | **3.09** | **5.74** | **0.82** | **13.67** | **7.70** | **19.14** | **58.87** |
| 1177´04 | 4.43 | 2.35 | 2.82 | 1.08 | 1.08 | 0.27 | 3.48 | 1.84 | 4.83 | 14.85 |
|  | 17.37 | 9.22 | 11.06 | 4.24 | 4.24 | 1.06 | 13.65 | 7.22 | 18.94 | 58.24 |
| 1178(1)´04 | 3.49 | 1.93 | – | – | 0.71 | 0.21 | 2.87 | 1.92 | – | 13.47 |
|  | 14.85 | 8.21 | – | – | 3.02 | 0.89 | 12.21 | 8.17 | – | 57.32 |
| 1180(1)´04 | 3.29 | 2.51 | – | 1.01 | 1.07 | 0.24 | 3.32 | 2.12 | 5.14 | 13.79 |
|  | 13.71 | 10.46 | – | 4.21 | 4.46 | 1.00 | 13.83 | 8.83 | 21.42 | 57.46 |
| 1181(1)´04 | 4.40 | 2.73 | 2.66 | 1.22 | 1.23 | 0.27 | 4.44 | 2.66 | 5.97 | 17.34 |
|  | 14.52 | 9.01 | 8.78 | 4.03 | 4.06 | 0.89 | 14.65 | 8.78 | 19.70 | 57.23 |
| 1181(2)´04 | 3.47 | 2.07 | 1.80 | 1.09 | – | – | 4.48 | 2.15 | – | 17.13 |
|  | 11.41 | 6.81 | 5.92 | 3.59 | – | – | 14.74 | 7.07 | – | 56.35 |
| 1183(1)´04 | – | 2.61 | 2.55 | 1.15 | – | – | 4.21 | 2.47 | 5.53 | 18.36 |
|  | – | 8.45 | 8.25 | 3.72 | – | – | 13.62 | 7.99 | 17.90 | 59.42 |
| **1181(2)/1183(1)´04** | **3.47** | **2.61** | **2.55** | **1.15** | – | – | **4.48** | **2.47** | **5.53** | **18.36** |
|  | **11.23** | **8.45** | **8.25** | **3.72** | – | – | **14.50** | **7.99** | **17.90** | **59.42** |
| 1182´04 | 5.03 | 2.72 | – | 1.62 | 1.42 | 0.27 | 4.07 | 2.73 | – | 15.22 |
|  | 18.42 | 9.96 | – | 5.93 | 5.20 | 0.99 | 14.91 | 10.00 | – | 55.75 |
| 1184(1)´04 | – | – | 2.22 | 0.56 | 0.98 | 0.13 | 3.48 | 2.09 | – | 15.00 |
|  | – | – | 8.60 | 2.17 | 3.80 | 0.50 | 13.49 | 8.10 | – | 58.14 |
| 1185´04 | 4.54 | 2.65 | 2.60 | 0.88 | 1.88 | 0.25 | 3.83 | 2.33 | 4.90 | 15.29 |

S1 Table. (Continued)

|  | 16.51 | 9.64 | 9.45 | 3.20 | 6.84 | 0.91 | 13.93 | 8.47 | 17.82 | 55.60 |
| --- | --- | --- | --- | --- | --- | --- | --- | --- | --- | --- |
| 1186´04 | 4.70 | 2.89 | 2.54 | 0.93 | 1.33 | 0.30 | 3.92 | 2.46 | 5.04 | 16.31 |
|  | 16.91 | 10.40 | 9.14 | 3.35 | 4.78 | 1.08 | 14.10 | 8.85 | 18.13 | 58.67 |
| **1185/1186´04** | **4.70** | **2.89** | **2.60** | **0.93** | **1.88** | **0.30** | **3.92** | **2.46** | **5.04** | **16.31** |
|  | **16.91** | **10.40** | **9.35** | **3.35** | **6.76** | **1.08** | **14.10** | **8.85** | **18.13** | **58.67** |
| 1187´04 | 3.77 | 3.33 | 2.28 | 1.37 | 1.56 | 0.28 | 4.00 | 2.46 | 5.43 | 15.11 |
|  | 13.86 | 12.24 | 8.38 | 5.04 | 5.74 | 1.03 | 14.71 | 9.04 | 19.96 | 55.55 |
| 1188´04 | 4.01 | 2.10 | – | 0.98 | 1.21 | 0.19 | – | 1.82 | 4.94 | 16.57 |
|  | 14.12 | 7.39 | – | 3.45 | 4.26 | 0.67 | – | 6.41 | 17.39 | 58.35 |
| 1189´04 | 3.65 | 1.58 | – | – | – | – | 3.05 | 1.84 | – | 14.60 |
|  | 14.96 | 6.48 | – | – | – | – | 12.50 | 7.54 | – | 59.84 |
| 1190´04 | 3.94 | 2.83 | – | – | 1.21 | 0.26 | 3.58 | 2.57 | 6.11 | 16.70 |
|  | 13.40 | 9.63 | – | – | 4.12 | 0.88 | 12.18 | 8.74 | 20.78 | 56.80 |
| 1192´04 | 4.16 | 2.97 | 2.27 | 1.22 | 1.12 | 0.19 | 3.93 | 2.31 | 5.05 | 16.86 |
|  | 14.75 | 10.53 | 8.05 | 4.33 | 3.97 | 0.67 | 13.94 | 8.19 | 17.91 | 59.79 |
| 1192a´05 | – | – | 1.21 | 1.06 | – | – | 3.04 | 2.46 | 5.49 | 17.88 |
|  | – | – | 3.85 | 3.38 | – | – | 9.68 | 7.83 | 17.48 | 56.94 |
| 1192b´05 | – | – | 1.60 | 1.09 | – | – | 2.81 | 2.26 | 5.43 | 17.99 |
|  | – | – | 5.08 | 3.46 | – | – | 8.92 | 7.17 | 17.24 | 57.11 |
| **1192a/b´04** | – | – | **1.60** | **1.09** | – | – | **3.04** | **2.46** | **5.49** | **17.99** |
|  | – | – | **5.08** | **3.46** | – | – | **9.65** | **7.81** | **17.43** | **57.11** |
| 1193´04 | 3.42 | – | 1.25 | 0.64 | 0.84 | 0.22 | 3.04 | 1.99 | 4.45 | 15.69 |
|  | 12.62 | – | 4.61 | 2.36 | 3.10 | 0.81 | 11.22 | 7.34 | 16.42 | 57.90 |
| 1194´04 | 3.67 | 2.25 | 1.80 | 1.14 | 0.90 | 0.20 | 3.81 | 1.99 | 5.38 | 14.49 |
|  | 14.28 | 8.75 | 7.00 | 4.44 | 3.50 | 0.78 | 14.82 | 7.74 | 20.93 | 56.38 |
| 1198a´04 | 3.47 | 2.47 | 2.32 | 1.22 | 1.34 | 0.24 | 3.22 | 2.01 | 4.24 | 12.82 |
|  | 15.56 | 11.08 | 10.40 | 5.47 | 6.01 | 1.08 | 14.44 | 9.01 | 19.01 | 57.49 |
| 1198b´04 | 3.43 | 2.51 | 1.67 | 1.00 | 1.21 | 0.19 | 3.26 | 1.95 | 4.36 | 12.78 |
|  | 15.31 | 11.21 | 7.46 | 4.46 | 5.40 | 0.85 | 14.55 | 8.71 | 19.46 | 57.05 |
| **1198a/b´04** | **3.47** | **2.51** | **2.32** | **1.22** | **1.34** | **0.24** | **3.26** | **2.01** | **4.36** | **12.82** |

S1 Table. (Continued)

|  | **15.49** | **11.21** | **10.36** | **5.45** | **5.98** | **1.07** | **14.55** | **8.97** | **19.46** | **57.23** |
| --- | --- | --- | --- | --- | --- | --- | --- | --- | --- | --- |
| 1199a´04 | 4.15 | 3.12 | 2.43 | 1.21 | 1.45 | 0.21 | 4.48 | 2.55 | 6.85 | 15.98 |
|  | 15.09 | 11.35 | 8.84 | 4.40 | 5.27 | 0.76 | 16.29 | 9.27 | 24.91 | 58.11 |
| 1199b´04 | 3.77 | 2.34 | 3.60 | 1.00 | 0.99 | 0.19 | 4.16 | 2.48 | 6.76 | 15.78 |
|  | 14.39 | 8.93 | 13.74 | 3.82 | 3.78 | 0.73 | 15.88 | 9.47 | 25.80 | 60.23 |
| **1199a/b´04** | **4.15** | **3.12** | **3.60** | **1.21** | **1.45** | **0.21** | **4.48** | **2.55** | **6.85** | **15.98** |
|  | **15.09** | **11.35** | **13.09** | **4.40** | **5.27** | **0.76** | **16.29** | **9.27** | **24.91** | **58.11** |
| 1200´04 | 4.06 | 3.00 | 2.87 | 1.60 | 1.30 | 0.26 | – | 2.56 | 5.98 | 17.18 |
|  | 13.05 | 9.65 | 9.23 | 5.14 | 4.18 | 0.84 | – | 8.23 | 19.23 | 55.24 |
| 1202´04 | 3.55 | 2.71 | 1.74 | 1.06 | 1.50 | 0.23 | 2.43 | 1.90 | 5.65 | 16.39 |
|  | 11.99 | 9.16 | 5.88 | 3.58 | 5.07 | 0.78 | 8.21 | 6.42 | 19.09 | 55.37 |
| 1203a´05 | – | 1.46 | 1.73 | 0.94 | 1.06 | 0.20 | 2.54 | – | – | 16.78 |
|  | – | 5.10 | 6.05 | 3.29 | 3.71 | 0.70 | 8.88 | – | – | 58.67 |
| 1203b´05 | 3.99 | 1.55 | 1.73 | 1.20 | 0.74 | 0.22 | 2.79 | 2.15 | – | 15.92 |
|  | 13.95 | 5.42 | 6.05 | 4.20 | 2.59 | 0.77 | 9.76 | 7.52 | – | 55.66 |
| **1203a/b´05** | **3.99** | **1.55** | **1.73** | **1.20** | **1.06** | **0.22** | **2.79** | **2.15** | – | **16.78** |
|  | **13.95** | **5.42** | **6.05** | **4.20** | **3.71** | **0.77** | **9.76** | **7.52** | – | **58.67** |
| 1204´04 | 4.76 | – | – | – | 1.10 | 0.23 | – | – | – | 17.31 |
|  | 16.36 | – | – | – | 3.78 | 0.79 | – | – | – | 59.48 |
| 1204´05 | 2.86 | 1.13 | – | 0.69 | 0.48 | 0.15 | 1.99 | 1.74 | – | 11.91 |
|  | 14.02 | 5.54 | – | 3.38 | 2.35 | 0.74 | 9.75 | 8.53 | – | 58.38 |
| 1206(1)´04 | 3.89 | 3.27 | 2.21 | 1.25 | 1.27 | 0.21 | 3.37 | 2.11 | 5.45 | 15.05 |
|  | 15.31 | 12.87 | 8.70 | 4.92 | 5.00 | 0.83 | 13.27 | 8.31 | 21.46 | 59.25 |
| 1211´04 | 3.91 | 2.60 | 2.33 | 1.05 | 1.41 | 0.19 | 3.49 | 2.15 | 5.12 | 15.29 |
|  | 15.45 | 10.28 | 9.21 | 4.15 | 5.57 | 0.75 | 13.79 | 8.50 | 20.24 | 60.43 |
| **1206(1)/1211´04** | **3.91** | **3.27** | **2.33** | **1.25** | **1.41** | **0.21** | **3.49** | **2.15** | **5.45** | **15.29** |
|  | **15.39** | **12.87** | **9.17** | **4.92** | **5.55** | **0.83** | **13.74** | **8.46** | **21.46** | **60.20** |
| 1209´04 | – | – | 2.47 | 1.23 | – | – | 3.75 | 2.23 | – | – |
|  | – | – | 9.76 | 4.86 | – | – | 14.82 | 8.81 | – | – |
| 1209a´05 | 4.92 | – | 2.52 | 1.45 | 1.13 | 0.24 | 3.94 | 2.50 | 7.35 | 21.68 |

S1 Table. (Continued)

|  | 13.78 | – | 7.06 | 4.06 | 3.17 | 0.67 | 11.04 | 7.00 | 20.59 | 60.73 |
| --- | --- | --- | --- | --- | --- | --- | --- | --- | --- | --- |
| 1209b´05 | 4.13 | – | 1.83 | 1.48 | 1.00 | 0.19 | 3.86 | 2.51 | 7.12 | 21.65 |
|  | 11.57 | – | 5.13 | 4.15 | 2.80 | 0.53 | 10.81 | 7.03 | 19.94 | 60.64 |
| **1209a/b´05** | **4.92** | – | **2.52** | **1.48** | **1.13** | **0.24** | **3.94** | **2.51** | **7.35** | **21.68** |
|  | **13.78** | – | **7.06** | **4.15** | **3.17** | **0.67** | **11.04** | **7.03** | **20.59** | **60.73** |
| 1213(1)´04 | 3.56 | 2.89 | – | – | 1.50 | 0.25 | 3.24 | 2.54 | 6.17 | 16.57 |
|  | 12.36 | 10.03 | – | – | 5.21 | 0.87 | 11.25 | 8.82 | 21.42 | 57.53 |
| 1215(1)´04 | 3.92 | 2.80 | 2.58 | 1.15 | 1.16 | 0.26 | 3.74 | 2.22 | – | 14.44 |
|  | 16.07 | 11.48 | 10.57 | 4.71 | 4.75 | 1.07 | 15.33 | 9.10 | – | 59.18 |
| 1217a(1)´04 | 3.45 | – | 2.13 | 1.27 | 1.32 | 0.20 | – | 1.87 | 4.77 | 14.93 |
|  | 13.02 | – | 8.04 | 4.79 | 4.98 | 0.75 | – | 7.06 | 18.00 | 56.34 |
| 1217b(1)´04 | – | – | – | – | – | – | – | – | – | – |
|  | – | – | – | – | – | – | – | – | – | – |
| **1217a(1)/b(1)´04** | **3.45** | – | **2.13** | **1.27** | **1.32** | **0.20** | – | **1.87** | **4.77** | **14.93** |
|  | **13.02** | – | **8.04** | **4.79** | **4.98** | **0.75** | – | **7.06** | **18.00** | **56.34** |
| 1218´04 | 3.76 | – | – | 1.13 | – | – | 3.10 | 1.81 | 4.48 | 13.42 |
|  | 15.73 | – | – | 4.73 | – | – | 12.97 | 7.57 | 18.74 | 56.15 |
| 1218a´05 | 4.54 | 2.76 | – | – | 1.35 | 0.27 | 4.59 | 3.05 | – | 19.38 |
|  | 13.59 | 8.26 | – | – | 4.04 | 0.81 | 13.74 | 9.13 | – | 58.02 |
| 1218b´05 | 4.43 | 2.82 | – | – | 1.81 | 0.19 | 4.71 | 3.03 | – | 19.09 |
|  | 13.47 | 8.57 | – | – | 5.50 | 0.58 | 14.32 | 9.21 | – | 58.01 |
| **1218a/b´05** | **4.54** | **2.82** | – | – | **1.81** | **0.27** | **4.71** | **3.05** | – | **19.37** |
|  | **13.59** | **8.44** | – | – | **5.42** | **0.81** | **14.10** | **9.13** | – | **57.99** |
| 1219(1)´04 | 4.11 | – | – | 0.92 | 1.13 | 0.24 | 3.58 | 2.09 | – | 14.84 |
|  | 16.05 | – | – | 3.59 | 4.41 | 0.94 | 13.98 | 8.16 | – | 57.97 |
| 1220(1)´04 | 4.17 | 2.72 | 2.41 | 1.33 | – | – | 4.24 | 2.16 | 5.25 | 15.87 |
|  | 15.33 | 10.00 | 8.86 | 4.89 | – | – | 15.59 | 7.94 | 19.30 | 58.35 |
| 1220R´04 | 3.23 | 1.83 | – | 0.95 | 0.90 | 0.25 | 3.25 | 1.98 | – | 13.53 |
|  | 13.98 | 7.92 | – | 4.11 | 3.90 | 1.08 | 14.07 | 8.57 | – | 58.57 |
| 1221(1)´04 | 3.72 | 2.57 | 3.55 | – | 0.98 | 0.23 | 3.46 | 2.54 | 6.20 | 16.65 |

S1 Table. (Continued)

|  | 12.78 | 8.83 | 12.20 | – | 3.37 | 0.79 | 11.89 | 8.73 | 21.31 | 57.22 |
| --- | --- | --- | --- | --- | --- | --- | --- | --- | --- | --- |
| 1227(1)´04 | 4.71 | 2.63 | 1.77 | 0.67 | – | – | 3.91 | 2.61 | – | 16.91 |
|  | 16.58 | 9.26 | 6.23 | 2.36 | – | – | 13.77 | 9.19 | – | 59.54 |
| 1228(1)´04 | 4.55 | 3.10 | 2.94 | 1.34 | 1.30 | 0.26 | 4.52 | 2.24 | 5.83 | 18.61 |
|  | 14.54 | 9.90 | 9.39 | 4.28 | 4.15 | 0.83 | 14.44 | 7.16 | 18.63 | 59.46 |
| 1237R(1)´04 | 4.58 | 2.87 | 3.34 | 1.45 | 1.72 | 0.28 | 4.63 | 2.49 | 5.74 | 18.47 |
|  | 14.68 | 9.20 | 10.71 | 4.65 | 5.51 | 0.90 | 14.84 | 7.98 | 18.40 | 59.20 |
| **1228(1)/1237R(1)´04** | **4.58** | **3.10** | **3.34** | **1.45** | **1.72** | **0.28** | **4.63** | **2.49** | **5.83** | **18.61** |
|  | **14.63** | **9.90** | **10.67** | **4.63** | **5.50** | **0.89** | **14.79** | **7.96** | **18.63** | **59.46** |
| 1233´04 | – | – | 2.42 | 1.29 | – | – | 3.09 | – | 5.34 | – |
|  | – | – | 8.80 | 4.69 | – | – | 11.24 | – | 19.42 | – |
| 1234(1)´04 | 3.04 | 2.09 | 2.75 | 1.22 | – | – | 3.39 | – | – | 16.64 |
|  | 10.97 | 7.55 | 9.93 | 4.40 | – | – | 12.24 | – | – | 60.07 |
| **1233/1234(1)´04** | **3.04** | **2.09** | **2.75** | **1.29** | – | – | **3.39** | – | **5.34** | **16.64** |
|  | **10.97** | **7.55** | **9.93** | **4.66** | – | – | **12.24** | – | **19.28** | **60.07** |
| 1234(2)´04 | 4.49 | 3.14 | 2.37 | 1.29 | 1.20 | 0.14 | 3.35 | 2.71 | 6.15 | 17.47 |
|  | 15.81 | 11.06 | 8.35 | 4.54 | 4.23 | 0.49 | 11.80 | 9.54 | 21.65 | 61.51 |
| 1234R´04 | 3.79 | 2.47 | 3.43 | 0.94 | 1.24 | 0.22 | 3.73 | 2.15 | 5.79 | 16.78 |
|  | 13.39 | 8.73 | 12.12 | 3.32 | 4.38 | 0.78 | 13.18 | 7.60 | 20.46 | 59.29 |
| 1236(1)´04 | 4.72 | 2.24 | 2.34 | 1.02 | 1.10 | 0.29 | 3.95 | 2.44 | – | 17.09 |
|  | 16.33 | 7.75 | 8.10 | 3.53 | 3.81 | 1.00 | 13.67 | 8.44 | – | 59.13 |
| 1237(1)´04 | 4.80 | 2.92 | 2.54 | 1.43 | 1.90 | 0.27 | 3.69 | 2.41 | 6.44 | 16.14 |
|  | 17.14 | 10.43 | 9.07 | 5.11 | 6.79 | 0.96 | 13.18 | 8.61 | 23.00 | 57.64 |
| 1237(2)´04 | 4.10 | 2.73 | 2.61 | 1.97 | 1.54 | 0.24 | 3.38 | 3.25 | 7.67 | 17.89 |
|  | 14.91 | 9.93 | 9.49 | 7.16 | 5.60 | 0.87 | 12.29 | 11.82 | 27.89 | 65.05 |
| 1324´04 | 4.78 | 2.94 | 3.70 | 1.55 | 1.75 | 0.26 | 3.96 | 2.54 | 5.57 | 15.90 |
|  | 17.19 | 10.58 | 13.31 | 5.58 | 6.29 | 0.94 | 14.24 | 9.14 | 20.04 | 57.19 |
| 1325´04 | 4.06 | 2.24 | 2.01 | 1.24 | – | 0.21 | 3.44 | 1.73 | 4.55 | 15.34 |
|  | 15.09 | 8.33 | 7.47 | 4.61 | – | 0.78 | 12.79 | 6.43 | 16.91 | 57.03 |
|  |  |  |  |  |  |  |  |  |  |  |

S1 Table. (Continued)

| ID | pA | poD | poA | lpc | P–A | V–A | P–V | pV | pP | lmd |
| --- | --- | --- | --- | --- | --- | --- | --- | --- | --- | --- |
| 1142´04 | 11.54 | 9.59 | 8.86 | 5.55 | 5.24 | 2.00 | 3.17 | 9.44 | 6.38 | 2.56 |
|  | 55.22 | 45.89 | 42.39 | 26.56 | 25.07 | 9.57 | 15.17 | 45.17 | 30.53 | 12.25 |
| 1144´04 | 13.98 | 9.45 | 8.69 | 5.33 | – | 2.18 | – | 11.74 | – | 3.33 |
|  | 59.24 | 40.04 | 36.82 | 22.58 | – | 9.24 | – | 49.75 | – | 14.11 |
| 1146´04 | 13.99 | 9.82 | 8.59 | 5.15 | 5.95 | 2.58 | 3.35 | 11.48 | 8.04 | 2.67 |
|  | 59.03 | 41.43 | 36.24 | 21.73 | 25.11 | 10.89 | 14.14 | 48.44 | 33.92 | 11.27 |
| **1144/1146´04** | **13.99** | **9.82** | **8.69** | **5.33** | **5.95** | **2.58** | **3.35** | **11.74** | **8.04** | **3.33** |
|  | **59.03** | **41.43** | **36.67** | **22.49** | **25.11** | **10.89** | **14.14** | **49.54** | **33.92** | **14.05** |
| 1145´04 | 13.56 | 8.90 | 9.23 | 5.90 | 6.10 | 1.84 | 4.30 | 11.56 | 7.36 | – |
|  | 61.36 | 40.27 | 41.76 | 26.70 | 27.60 | 8.33 | 19.46 | 52.31 | 33.30 | – |
| 1147´04 | 12.39 | 9.91 | 9.36 | 5.81 | 5.85 | 1.72 | 4.13 | 10.50 | 6.32 | 2.10 |
|  | 57.63 | 46.09 | 43.53 | 27.02 | 27.21 | 8.00 | 19.21 | 48.40 | 29.40 | 9.77 |
| 1148(1)´04 | 15.03 | 10.77 | 10.48 | 7.04 | 6.95 | 1.88 | 5.06 | 14.26 | 9.16 | 2.95 |
|  | 56.29 | 40.34 | 39.25 | 26.37 | 26.03 | 7.04 | 18.95 | 53.41 | 34.31 | 11.05 |
| 1149´04 | 17.04 | 11.81 | 11.62 | 7.54 | 7.48 | 2.76 | 4.62 | 14.38 | 9.32 | 2.74 |
|  | 59.37 | 41.15 | 40.49 | 26.27 | 26.06 | 9.62 | 16.10 | 50.10 | 32.47 | 9.55 |
| 1150´04 | 16.00 | 10.97 | 10.15 | 6.85 | 6.38 | 1.89 | 4.49 | 14.21 | 9.29 | 3.11 |
|  | 57.14 | 39.18 | 36.25 | 24.46 | 22.79 | 6.75 | 16.04 | 50.75 | 33.18 | 11.11 |
| 1151´04 | 13.29 | 9.27 | 8.40 | 5.91 | 6.12 | 2.09 | 3.97 | 11.25 | 7.11 | – |
|  | 57.53 | 40.13 | 36.36 | 25.58 | 26.49 | 9.05 | 17.19 | 48.70 | 30.78 | – |
| 1152´04 | 13.68 | 9.48 | 8.33 | 5.97 | 6.05 | 1.96 | 4.04 | 11.65 | 7.57 | 2.61 |
|  | 58.21 | 40.34 | 35.45 | 25.40 | 25.74 | 8.34 | 17.19 | 49.57 | 32.21 | 11.11 |
| **1151/1152´04** | **13.68** | **9.48** | **8.40** | **5.97** | **6.12** | **2.09** | **4.04** | **11.65** | **7.57** | **2.61** |
|  | **58.21** | **40.34** | **35.74** | **25.40** | **26.04** | **8.89** | **17.19** | **49.57** | **32.21** | **11.11** |
| 1153´04 | 14.49 | 9.28 | 8.57 | 6.20 | 6.45 | 2.03 | 4.39 | 12.41 | 8.01 | – |
|  | 63.00 | 40.35 | 37.26 | 26.96 | 28.04 | 8.83 | 19.09 | 53.96 | 34.83 | – |
| 1154a´04 | 17.70 | 11.45 | 10.59 | 7.10 | 8.50 | 3.04 | 5.53 | 14.71 | 9.17 | 2.87 |
|  | 62.32 | 40.32 | 37.29 | 25.00 | 29.93 | 10.70 | 19.47 | 51.80 | 32.29 | 10.11 |
| 1154b´04 | 18.20 | 11.08 | 10.29 | 6.91 | 8.70 | 3.09 | 5.68 | 15.17 | 9.39 | 3.24 |

S1 Table. (Continued)

|  | | 63.64 | | 38.74 | | 35.98 | | 24.16 | | 30.42 | | 10.80 | | 19.86 | | 53.04 | 32.83 | 11.33 |
| --- | --- | --- | --- | --- | --- | --- | --- | --- | --- | --- | --- | --- | --- | --- | --- | --- | --- | --- |
| **1154a/b´04** | | **18.20** | | **11.45** | | **10.59** | | **7.10** | | **8.70** | | **3.09** | | **5.68** | | **15.17** | **9.39** | **3.24** |
|  | | **63.64** | | **40.03** | | **37.03** | | **24.83** | | **30.42** | | **10.80** | | **19.86** | | **53.04** | **32.83** | **11.33** |
| 1155´04 | | 16.93 | | 9.90 | | 9.65 | | 7.20 | | 7.31 | | 2.61 | | 4.70 | | 14.29 | 9.69 | 2.84 |
|  | | 63.89 | | 37.36 | | 36.42 | | 27.17 | | 27.58 | | 9.85 | | 17.74 | | 53.92 | 36.57 | 10.72 |
| 1156´04 | | 15.74 | | – | | – | | – | | 6.70 | | 2.71 | | 3.98 | | 12.99 | 8.84 | 2.92 |
|  | | 59.85 | | – | | – | | – | | 25.48 | | 10.30 | | 15.13 | | 49.39 | 33.61 | 11.10 |
| 1157(1)´04 | | 16.97 | | 10.47 | | 10.14 | | 6.45 | | 7.68 | | 1.53 | | 5.93 | | 15.35 | 9.30 | 3.37 |
|  | | 60.82 | | 37.53 | | 36.34 | | 23.12 | | 27.53 | | 5.48 | | 21.25 | | 55.02 | 33.33 | 12.08 |
| 1158(1)´04 | | 16.91 | | 10.29 | | 9.98 | | 6.21 | | 6.46 | | 1.42 | | 5.07 | | 15.42 | 10.29 | – |
|  | | 62.40 | | 37.97 | | 36.83 | | 22.92 | | 23.84 | | 5.24 | | 18.71 | | 56.90 | 37.97 | – |
| **1157(1)/1158(1)´04** | | **16.97** | | **10.47** | | **10.14** | | **6.45** | | **7.68** | | **1.53** | | **5.93** | | **15.42** | **10.29** | **3.37** |
|  | | **60.82** | | **37.53** | | **36.34** | | **23.12** | | **27.53** | | **5.48** | | **21.25** | | **55.27** | **36.88** | **12.08** |
| 1159a(1)´04 | | 15.19 | | 12.60 | | 13.62 | | 6.50 | | 6.61 | | 1.95 | | 4.66 | | 13.20 | 8.43 | – |
|  | | 61.00 | | 50.60 | | 54.70 | | 26.10 | | 26.55 | | 7.83 | | 18.71 | | 53.01 | 33.86 | – |
| 1159b(1)´04 | | – | | 10.27 | | – | | – | | – | | – | | – | | – | 8.87 | – |
|  | | – | | 40.59 | | – | | – | | – | | – | | – | | – | 35.06 | – |
| **1159a(1)/b(1)´04** | | **15.19** | | **12.60** | | **13.62** | | **6.50** | | **6.61** | | **1.95** | | **4.66** | | **13.20** | **8.87** | – |
|  | | **60.04** | | **49.80** | | **53.83** | | **25.69** | | **26.13** | | **7.71** | | **18.42** | | **52.17** | **35.06** | – |
| 1159a(2)´04 | | 16.66 | | 12.37 | | 11.83 | | 7.87 | | 6.48 | | 2.24 | | 4.22 | | 14.31 | 10.00 | 3.87 |
|  | | 58.25 | | 43.25 | | 41.36 | | 27.52 | | 22.66 | | 7.83 | | 14.76 | | 50.03 | 34.97 | 13.53 |
| 1159b(2)´04 | | 16.77 | | 12.10 | | 11.63 | |  | | 6.93 | | 2.35 | | 4.57 | | 14.25 | 9.62 | 3.50 |
|  | | 57.83 | | 41.72 | | 40.10 | | 0.00 | | 23.90 | | 8.10 | | 15.76 | | 49.14 | 33.17 | 12.07 |
| **1159a(2)/b(2)´04** | | **16.77** | | **12.37** | | **11.83** | | **8.03** | | **6.93** | | **2.35** | | **4.57** | | **14.31** | **10.00** | **3.87** |
|  | **57.83** | | **42.66** | | **40.79** | | **27.69** | | **23.90** | | **8.10** | | **15.76** | | **49.34** | | **34.48** | **13.34** |
| 1160a´04 | 15.78 | | 11.10 | | 10.46 | | 6.95 | | 6.62 | | 1.83 | | 4.69 | | 13.80 | | 9.08 | 2.58 |
|  | 59.32 | | 41.73 | | 39.32 | | 26.13 | | 24.89 | | 6.88 | | 17.63 | | 51.88 | | 34.14 | 9.70 |
| 1161a´04 | 15.91 | | 11.09 | | 10.05 | | 6.62 | | 6.70 | | 1.82 | | 4.88 | | 14.06 | | 9.14 | 3.10 |
|  | 60.73 | | 42.33 | | 38.36 | | 25.27 | | 25.57 | | 6.95 | | 18.63 | | 53.66 | | 34.89 | 11.83 |
| **1160a/61a´04** | **15.91** | | **11.10** | | **10.46** | | **6.95** | | **6.70** | | **1.83** | | **4.88** | | **14.06** | | **9.14** | **3.10** |

S1 Table. (Continued)

|  | **59.81** | **41.73** | **39.32** | **26.13** | **25.19** | **6.88** | **18.35** | **52.86** | **34.36** | **11.65** |
| --- | --- | --- | --- | --- | --- | --- | --- | --- | --- | --- |
| 1160b´04 | 16.57 | 12.24 | 11.80 | 7.38 | 7.37 | 2.24 | 5.15 | 14.25 | 9.10 | 3.09 |
|  | 60.47 | 44.67 | 43.07 | 26.93 | 26.90 | 8.18 | 18.80 | 52.01 | 33.21 | 11.28 |
| 1161b´04 | 16.58 | 12.22 | 12.03 | 7.70 | 6.79 | 2.05 | 4.67 | 14.51 | 9.82 | 2.88 |
|  | 59.86 | 44.12 | 43.43 | 27.80 | 24.51 | 7.40 | 16.86 | 52.38 | 35.45 | 10.40 |
| **1160b/1161b´04** | **16.58** | **12.24** | **12.03** | **7.70** | **7.37** | **2.24** | **5.15** | **14.51** | **9.82** | **3.09** |
|  | **59.86** | **44.19** | **43.43** | **27.80** | **26.61** | **8.09** | **18.59** | **52.38** | **35.45** | **11.16** |
| 1162´04 | 19.68 | 13.63 | 12.44 | 8.41 | 7.61 | 2.95 | 4.68 | 14.91 | 11.68 | 3.36 |
|  | 60.37 | 41.81 | 38.16 | 25.80 | 23.34 | 9.05 | 14.36 | 45.74 | 35.83 | 10.31 |
| 1163a(1)´04 | 15.40 | – | – | – | 8.04 | 2.88 | 5.13 | 12.63 | 7.51 | 2.71 |
|  | 60.39 | – | – | – | 31.53 | 11.29 | 20.12 | 49.53 | 29.45 | 10.63 |
| 1163b(2)´04 | 15.95 | – | – | 6.82 | 7.79 | 3.06 | 4.70 | 12.78 | 7.99 | 2.27 |
|  | 62.06 | – | – | 26.54 | 30.31 | 11.91 | 18.29 | 49.73 | 31.09 | 8.83 |
| **1163a(1)/b(2)´04** | **15.95** | **–** | **–** | **6.82** | **8.04** | **3.06** | **5.13** | **12.78** | **7.99** | **2.71** |
|  | **62.06** | – | – | **26.54** | **31.28** | **11.91** | **19.96** | **49.73** | **31.09** | **10.54** |
| 1163a(2)´04 | 15.05 | 9.41 | 8.74 | 5.85 | 6.90 | 2.33 | 4.65 | 12.60 | 7.92 | 2.87 |
|  | 62.19 | 38.88 | 36.12 | 24.17 | 28.51 | 9.63 | 19.21 | 52.07 | 32.73 | 11.86 |
| 1163b(1)´04 | 14.60 | 9.14 | 8.79 | 5.91 | 6.58 | 2.09 | 4.40 | 12.40 | 7.83 | 2.82 |
|  | 60.58 | 37.93 | 36.47 | 24.52 | 27.30 | 8.67 | 18.26 | 51.45 | 32.49 | 11.70 |
| **1163a(2)/b(1)´04** | **15.05** | **9.41** | **8.79** | **5.91** | **6.90** | **2.33** | **4.65** | **12.60** | **7.92** | **2.87** |
|  | **62.19** | **38.88** | **36.32** | **24.42** | **28.51** | **9.63** | **19.21** | **52.07** | **32.73** | **11.86** |
| 1164a´04 | 20.81 | – | 11.48 | 7.57 | 7.99 | 2.11 | 5.72 | 18.69 | 12.23 | 3.10 |
|  | 64.23 | – | 35.43 | 23.36 | 24.66 | 6.51 | 17.65 | 57.69 | 37.75 | 9.57 |
| 1164b´04 | – | – | – | – | – | – | – | – | – | – |
|  | – | – | – | – | – | – | – | – | – | – |
| **1164a/b´04** | **20.81** | – | **11.48** | **7.57** | **7.99** | **2.11** | **5.72** | **18.69** | **12.23** | **3.10** |
|  | **64.23** | – | **35.43** | **23.36** | **24.66** | **6.51** | **17.65** | **57.69** | **37.75** | **9.57** |
| 1165a´04 | 19.13 | 11.91 | 11.10 | 7.09 | 8.28 | 2.41 | 6.19 | 16.40 | 10.15 | 2.99 |
|  | 62.93 | 39.18 | 36.51 | 23.32 | 27.24 | 7.93 | 20.36 | 53.95 | 33.39 | 9.84 |
| 1165b´04 | 20.08 | 12.05 | 11.30 | 7.33 | 8.77 | 3.26 | 5.82 | 16.99 | 11.35 | 2.93 |

S1 Table. (Continued)

|  | 67.16 | 40.30 | 37.79 | 24.52 | 29.33 | 10.90 | 19.46 | 56.82 | 37.96 | 9.80 |
| --- | --- | --- | --- | --- | --- | --- | --- | --- | --- | --- |
| **1165a/b´04** | **20.08** | **12.05** | **11.30** | **7.33** | **8.77** | **3.26** | **6.19** | **16.99** | **11.35** | **2.99** |
|  | **66.05** | **39.64** | **37.17** | **24.11** | **28.85** | **10.72** | **20.36** | **55.89** | **37.34** | **9.84** |
| 1166a´04 | 12.82 | 9.77 | 9.31 | 6.27 | 6.17 | 1.80 | 4.31 | 10.97 | 6.59 | 2.22 |
|  | 60.76 | 46.30 | 44.12 | 29.72 | 29.24 | 8.53 | 20.43 | 51.99 | 31.23 | 10.52 |
| 1166b´04 | 15.54 | 10.19 | 9.96 | 6.87 | 7.51 | 2.65 | 4.79 | 12.91 | 8.04 | 2.94 |
|  | 59.31 | 38.89 | 38.02 | 26.22 | 28.66 | 10.11 | 18.28 | 49.27 | 30.69 | 11.22 |
| 1167´04 | 16.24 | 11.28 | 11.04 | 7.33 | 6.50 | 1.93 | 4.65 | 14.34 | 9.72 | 3.45 |
|  | 59.71 | 41.47 | 40.59 | 26.95 | 23.90 | 7.10 | 17.10 | 52.72 | 35.74 | 12.68 |
| 1168´04 | 12.87 | 10.33 | 9.87 | 6.17 | 6.46 | 2.08 | 4.42 | 10.92 | 6.40 | 1.95 |
|  | 58.50 | 46.95 | 44.86 | 28.05 | 29.36 | 9.45 | 20.09 | 49.64 | 29.09 | 8.86 |
| 1170´04 | 14.88 | 10.02 | 9.35 | 5.47 | – | – | – | – | – | – |
|  | 62.00 | 41.75 | 38.96 | 22.79 | – | – | – | – | – | – |
| 1171´04 | 16.70 | – | – | 7.64 | – | 2.26 | – | 14.38 | – | 2.71 |
|  | 60.51 | – | – | 27.68 | – | 8.19 | – | 52.10 | – | 9.82 |
| 1172´04 | 15.04 | 10.30 | 9.12 | 5.85 | – | – | – | – | – | 2.73 |
|  | 55.29 | 37.87 | 33.53 | 21.51 | – | – | – | – | – | 10.04 |
| 1174´04 | 16.42 | 13.01 | 12.34 | 8.20 | 6.68 | 1.61 | 5.21 | 14.89 | 9.67 | 3.16 |
|  | 56.04 | 44.40 | 42.12 | 27.99 | 22.80 | 5.49 | 17.78 | 50.82 | 33.00 | 10.78 |
| 1175´04 | 15.34 | 10.54 | 10.06 | 6.78 | 6.82 | 2.84 | 3.96 | 12.41 | 8.41 | 2.89 |
|  | 58.77 | 40.38 | 38.54 | 25.98 | 26.13 | 10.88 | 15.17 | 47.55 | 32.22 | 11.07 |
| 1176a´04 | 16.24 | 10.74 | 9.12 | 6.26 | 7.48 | 2.43 | 5.01 | 13.78 | 8.70 | – |
|  | 65.22 | 43.13 | 36.63 | 25.14 | 30.04 | 9.76 | 20.12 | 55.34 | 34.94 | – |
| 1176b´04 | 15.41 | 10.68 | 9.76 | 6.76 | 6.88 | 2.22 | 4.72 | 13.18 | 8.39 | 2.86 |
|  | 60.20 | 41.72 | 38.13 | 26.41 | 26.88 | 8.67 | 18.44 | 51.48 | 32.77 | 11.17 |
| **1176a/b´04** | **16.24** | **10.74** | **9.76** | **6.76** | **7.48** | **2.43** | **5.01** | **13.78** | **8.70** | **2.86** |
|  | **63.44** | **41.95** | **38.13** | **26.41** | **29.22** | **9.49** | **19.57** | **53.83** | **33.98** | **11.17** |
| 1177´04 | 15.01 | 10.85 | 10.90 | 7.28 | 7.26 | 2.56 | 4.79 | 12.46 | 7.74 | – |
|  | 58.86 | 42.55 | 42.75 | 28.55 | 28.47 | 10.04 | 18.78 | 48.86 | 30.35 | – |
| 1178(1)´04 | 13.05 | 9.58 | 8.91 | 6.25 | – | 2.08 | – | 10.97 | – | 2.30 |

S1 Table. (Continued)

|  | 55.53 | 40.77 | 37.91 | 26.60 | – | 8.85 | – | 46.68 | – | 9.79 |
| --- | --- | --- | --- | --- | --- | --- | --- | --- | --- | --- |
| 1180(1)´04 | 14.39 | 9.01 | 9.01 | 6.01 | 5.90 | 1.42 | 4.47 | 12.94 | 8.41 | 2.68 |
|  | 59.96 | 37.54 | 37.54 | 25.04 | 24.58 | 5.92 | 18.63 | 53.92 | 35.04 | 11.17 |
| 1181(1)´04 | 17.89 | 12.82 | 12.07 | 7.55 | 7.85 | 2.76 | 5.25 | 15.51 | 10.14 | 3.28 |
|  | 59.04 | 42.31 | 39.83 | 24.92 | 25.91 | 9.11 | 17.33 | 51.19 | 33.47 | 10.83 |
| 1181(2)´04 | 17.84 | 12.69 | 12.08 | 7.77 | 9.74 | – | – | – | 8.05 | – |
|  | 58.68 | 41.74 | 39.74 | 25.56 | 32.04 | – | – | – | 26.48 | – |
| 1183(1)´04 | 19.32 | 13.33 | 12.05 | 7.53 | – | – | – | – | 9.23 | – |
|  | 62.52 | 43.14 | 39.00 | 24.37 | – | – | – | – | 29.87 | – |
| **1181(2)/1183(1)´04** | **19.32** | **13.33** | **12.08** | **7.77** | **9.74** | – | – | – | **9.23** | – |
|  | **62.52** | **43.14** | **39.09** | **25.15** | **31.52** | – | – | – | **29.87** | **–** |
| 1182´04 | 15.92 | 10.95 | 10.14 | 6.50 | 6.46 | 2.26 | 4.20 | 13.65 | 9.42 | 3.20 |
|  | 58.32 | 40.11 | 37.14 | 23.81 | 23.66 | 8.28 | 15.38 | 50.00 | 34.51 | 11.72 |
| 1184(1)´04 | 13.65 | 9.82 | – | 6.68 | 5.84 | 2.11 | 3.96 | 11.92 | 7.29 | – |
|  | 52.91 | 38.06 | – | 25.89 | 22.64 | 8.18 | 15.35 | 46.20 | 28.26 | – |
| 1185´04 | 15.76 | 12.38 | 11.23 | 7.14 | 7.24 | 1.83 | 5.48 | 13.92 | 8.41 | 2.65 |
|  | 57.31 | 45.02 | 40.84 | 25.96 | 26.33 | 6.65 | 19.93 | 50.62 | 30.58 | 9.64 |
| 1186´04 | 16.89 | 11.94 | 11.13 | 7.10 | 7.08 | 1.71 | 5.42 | 15.16 | 9.74 | 2.70 |
|  | 60.76 | 42.95 | 40.04 | 25.54 | 25.47 | 6.15 | 19.50 | 54.53 | 35.04 | 9.71 |
| **1185/1186´04** | **16.89** | **12.38** | **11.23** | **7.14** | **7.24** | **1.83** | **5.48** | **15.16** | **9.74** | **2.70** |
|  | **60.76** | **44.53** | **40.40** | **25.68** | **26.04** | **6.58** | **19.71** | **54.53** | **35.04** | **9.71** |
| 1187´04 | 16.43 | 11.58 | 11.34 | 7.40 | 7.39 | 2.36 | 5.10 | 14.14 | 8.93 | 2.83 |
|  | 60.40 | 42.57 | 41.69 | 27.21 | 27.17 | 8.68 | 18.75 | 51.99 | 32.83 | 10.40 |
| 1188´04 | 17.11 | – | – | 7.23 | 7.61 | 2.31 | 5.19 | 14.81 | 9.49 | 2.88 |
|  | 60.25 | – | – | 25.46 | 26.80 | 8.13 | 18.27 | 52.15 | 33.42 | 10.14 |
| 1189´04 | 13.80 | 9.52 | 9.12 | 6.45 | – | – | – | – | – | 1.96 |
|  | 56.56 | 39.02 | 37.38 | 26.43 | – | – | – | – | – | 8.03 |
| 1190´04 | 17.48 | 11.94 | 10.47 | 7.18 | 7.28 | 2.54 | 6.13 | 15.37 | 9.21 | 2.75 |
|  | 59.46 | 40.61 | 35.61 | 24.42 | 24.76 | 8.64 | 20.85 | 52.28 | 31.33 | 9.35 |
| 1192´04 | 16.57 | 11.93 | 11.38 | 7.39 | 8.33 | 2.99 | 5.37 | 13.58 | 8.10 | 2.53 |

S1 Table. (Continued)

|  | 58.76 | 42.30 | 40.35 | 26.21 | 29.54 | 10.60 | 19.04 | 48.16 | 28.72 | 8.97 |
| --- | --- | --- | --- | --- | --- | --- | --- | --- | --- | --- |
| 1192a´05 | 17.50 | – | – | – | 8.07 | – | – | – | 8.44 | 3.49 |
|  | 55.73 | – | – | – | 25.70 | – | – | – | 26.88 | 11.11 |
| 1192b´05 | 17.64 | – | – | – | 8.32 | – | – | – | 9.22 | 3.21 |
|  | 56.00 | – | – | – | 26.41 | – | – | – | 29.27 | 10.19 |
| **1192a/b´04** | **17.64** | **–** | **–** | **–** | **8.32** | **–** | **–** | **–** | **9.22** | **3.49** |
|  | **56.00** | **–** | **–** | **–** | **26.41** | **–** | **–** | **–** | **29.27** | **11.08** |
| 1193´04 | 16.22 | 10.09 | 10.55 | 7.56 | 7.66 | 2.73 | 4.91 | 13.38 | 8.47 | 2.04 |
|  | 59.85 | 37.23 | 38.93 | 27.90 | 28.27 | 10.07 | 18.12 | 49.37 | 31.25 | 7.53 |
| 1194´04 | 14.87 | 11.24 | 10.85 | 7.04 | 6.85 | 2.21 | 4.67 | 12.66 | 7.92 | 2.41 |
|  | 57.86 | 43.74 | 42.22 | 27.39 | 26.65 | 8.60 | 18.17 | 49.26 | 30.82 | 9.38 |
| 1198a´04 | 13.09 | 9.57 | 9.30 | 5.80 | 5.85 | 2.02 | 3.86 | 11.05 | 7.27 | 2.47 |
|  | 58.70 | 42.91 | 41.70 | 26.01 | 26.23 | 9.06 | 17.31 | 49.55 | 32.60 | 11.08 |
| 1198b´04 | 13.18 | 9.77 | 8.97 | 5.73 | 5.68 | 1.95 | 3.84 | 11.25 | 7.36 | 2.24 |
|  | 58.84 | 43.62 | 40.04 | 25.58 | 25.36 | 8.71 | 17.14 | 50.22 | 32.86 | 10.00 |
| **1198a/b´04** | **13.18** | **9.77** | **9.30** | **5.80** | **5.85** | **2.02** | **3.86** | **11.25** | **7.36** | **2.47** |
|  | **58.84** | **43.62** | **41.52** | **25.89** | **26.12** | **9.02** | **17.23** | **50.22** | **32.86** | **11.03** |
| 1199a´04 | 16.34 | 11.83 | 11.39 | 6.91 | 6.01 | 2.69 | 3.29 | 13.61 | 10.19 | 2.43 |
|  | 59.42 | 43.02 | 41.42 | 25.13 | 21.85 | 9.78 | 11.96 | 49.49 | 37.05 | 8.84 |
| 1199b´04 | 15.95 | 11.68 | 11.41 | 7.40 | 6.66 | 2.62 | 4.07 | 13.26 | 9.17 | 2.28 |
|  | 60.88 | 44.58 | 43.55 | 28.24 | 25.42 | 10.00 | 15.53 | 50.61 | 35.00 | 8.70 |
| **1199a/b´04** | **16.34** | **11.83** | **11.41** | **7.40** | **6.66** | **2.69** | **4.07** | **13.61** | **10.19** | **2.43** |
|  | **59.42** | **43.02** | **41.49** | **26.91** | **24.22** | **9.78** | **14.80** | **49.49** | **37.05** | **8.84** |
| 1200´04 | 18.66 | – | – | 8.69 | 8.30 | 2.96 | 5.37 | 15.51 | 9.96 | 2.35 |
|  | 60.00 | – | – | 27.94 | 26.69 | 9.52 | 17.27 | 49.87 | 32.03 | 7.56 |
| 1202´04 | 16.64 | 10.23 | 9.22 | 7.70 | 8.10 | 2.25 | 5.87 | 14.46 | 8.57 | 2.27 |
|  | 56.22 | 34.56 | 31.15 | 26.01 | 27.36 | 7.60 | 19.83 | 48.85 | 28.95 | 7.67 |
| 1203a´05 | 17.16 | – | – | – | 8.30 | 3.02 | 5.28 | 14.20 | 8.86 | 3.03 |
|  | 60.00 | – | – | – | 29.02 | 10.56 | 18.46 | 49.65 | 30.98 | 10.59 |
| 1203b´05 | 16.21 | 10.91 | 10.42 | 7.97 | 7.11 | 2.71 | 4.37 | 13.47 | 8.87 | 2.76 |

S1 Table. (Continued)

|  | 56.68 | 38.15 | 36.43 | 27.87 | 24.86 | 9.48 | 15.28 | 47.10 | 31.01 | 9.65 |
| --- | --- | --- | --- | --- | --- | --- | --- | --- | --- | --- |
| **1203a/b´05** | **17.16** | **10.91** | **10.42** | **7.97** | **8.30** | **3.02** | **5.28** | **14.20** | **8.87** | **3.03** |
|  | **60.00** | **38.15** | **36.43** | **27.87** | **29.02** | **10.56** | **18.46** | **49.65** | **31.01** | **10.59** |
| 1204´04 | 16.81 | – | – | 7.39 | 8.69 | 2.64 | 5.75 | 14.98 | 9.10 | – |
|  | 57.77 | – | – | 25.40 | 29.86 | 9.07 | 19.76 | 51.48 | 31.27 | – |
| 1204´05 | 11.74 | 7.87 | 7.49 | 5.80 | 5.15 | 1.83 | 3.32 | 9.89 | 6.52 | 2.29 |
|  | 57.55 | 38.58 | 36.72 | 28.43 | 25.25 | 8.97 | 16.27 | 48.48 | 31.96 | 11.23 |
| 1206(1)´04 | 14.84 | 10.59 | 10.31 | 6.50 | 6.65 | 2.35 | 4.33 | 12.49 | 7.95 | 2.83 |
|  | 58.43 | 41.69 | 40.59 | 25.59 | 26.18 | 9.25 | 17.05 | 49.17 | 31.30 | 11.14 |
| 1211´04 | 15.07 | 10.60 | 10.11 | 6.49 | 6.35 | 2.23 | 4.13 | 12.74 | 8.63 | 2.62 |
|  | 59.57 | 41.90 | 39.96 | 25.65 | 25.10 | 8.81 | 16.32 | 50.36 | 34.11 | 10.36 |
| **1206(1)/1211´04** | **15.07** | **10.60** | **10.31** | **6.50** | **6.65** | **2.35** | **4.33** | **12.74** | **8.63** | **2.83** |
|  | **59.33** | **41.73** | **40.59** | **25.59** | **26.18** | **9.25** | **17.05** | **50.16** | **33.98** | **11.14** |
| 1209´04 | 15.27 | – | – | – | 6.63 | – | – | – | 8.27 | – |
|  | 60.36 | – | – | – | 26.21 | – | – | – | 32.69 | – |
| 1209a´05 | 22.07 | 13.29 | 12.65 | 8.48 | 10.14 | 3.82 | 6.30 | 18.29 | 11.89 | 3.80 |
|  | 61.82 | 37.23 | 35.43 | 23.75 | 28.40 | 10.70 | 17.65 | 51.23 | 33.31 | 10.64 |
| 1209b´05 | 21.96 | 12.62 | 12.13 | 9.09 | 10.34 | 3.93 | 6.38 | 18.04 | 11.54 | 3.10 |
|  | 61.51 | 35.35 | 33.98 | 25.46 | 28.96 | 11.01 | 17.87 | 50.53 | 32.32 | 8.68 |
| **1209a/b´05** | **22.07** | **13.29** | **12.65** | **9.09** | **10.34** | **3.93** | **6.38** | **18.29** | **11.89** | **3.80** |
|  | **61.82** | **37.23** | **35.43** | **25.46** | **28.96** | **11.01** | **17.87** | **51.23** | **33.31** | **10.64** |
| 1213(1)´04 | 18.06 | 11.77 | 10.61 | 7.33 | 7.19 | 2.18 | 5.02 | 15.97 | 11.01 | 2.93 |
|  | 62.71 | 40.87 | 36.84 | 25.45 | 24.97 | 7.57 | 17.43 | 55.45 | 38.23 | 10.17 |
| 1215(1)´04 | 13.69 | 10.68 | 10.41 | 7.11 | 5.93 | 1.78 | 4.08 | 11.74 | 7.66 | – |
|  | 56.11 | 43.77 | 42.66 | 29.14 | 24.30 | 7.30 | 16.72 | 48.11 | 31.39 | – |
| 1217a(1)´04 | 16.92 | – | – | 6.80 | 8.46 | 2.86 | 5.59 | 14.00 | 8.35 | – |
|  | 63.85 | – | – | 25.66 | 31.92 | 10.79 | 21.09 | 52.83 | 31.51 | – |
| 1217b(1)´04 | – | – | – | – | – | – | – | – | – | – |
|  | – | – | – | – | – | – | – | – | – | – |
| **1217a(1)/b(1)´04** | **16.92** | **–** | **–** | **6.80** | **8.46** | **2.86** | **5.59** | **14.00** | **8.35** | **–** |

S1 Table. (Continued)

|  | **63.85** | **–** | **–** | **25.66** | **31.92** | **10.79** | **21.09** | **52.83** | **31.51** | **–** |
| --- | --- | --- | --- | --- | --- | --- | --- | --- | --- | --- |
| 1218´04 | 13.59 | 10.42 | 9.34 | 6.43 | 5.97 | 2.00 | 3.86 | 11.55 | 7.51 | 2.40 |
|  | 56.86 | 43.60 | 39.08 | 26.90 | 24.98 | 8.37 | 16.15 | 48.33 | 31.42 | 10.04 |
| 1218a´05 | 20.08 | 13.45 | 12.67 | 8.47 | 6.80 | 2.79 | 4.32 | 17.62 | 13.42 | 3.04 |
|  | 60.11 | 40.27 | 37.93 | 25.36 | 20.35 | 8.35 | 12.94 | 52.75 | 40.18 | 9.10 |
| 1218b´05 | 20.39 | 13.76 | 12.53 | 7.97 | – | 2.22 | – | 17.65 | – | 2.83 |
|  | 61.98 | 41.82 | 38.09 | 24.22 | – | 6.75 | – | 53.65 | – | 8.60 |
| **1218a/b´05** | **20.39** | **13.76** | **12.67** | **8.47** | **6.80** | **2.79** | **4.32** | **17.65** | **13.42** | **3.04** |
|  | **61.05** | **41.20** | **37.93** | **25.36** | **20.36** | **8.35** | **12.93** | **52.84** | **40.18** | **9.10** |
| 1219(1)´04 | 14.55 | 11.74 | 10.69 | 7.23 | 5.70 | 1.18 | 4.31 | 13.18 | 8.74 | – |
|  | 56.84 | 45.86 | 41.76 | 28.24 | 22.27 | 4.61 | 16.84 | 51.48 | 34.14 | – |
| 1220(1)´04 | 16.66 | 12.06 | 10.75 | 6.85 | 7.84 | 2.61 | 5.22 | 14.06 | 8.76 | 2.89 |
|  | 61.25 | 44.34 | 39.52 | 25.18 | 28.82 | 9.60 | 19.19 | 51.69 | 32.21 | 10.63 |
| 1220R´04 | 13.46 | 10.25 | 9.28 | 6.15 | 5.77 | 1.62 | 4.07 | 11.80 | 7.61 | 2.66 |
|  | 58.27 | 44.37 | 40.17 | 26.62 | 24.98 | 7.01 | 17.62 | 51.08 | 32.94 | 11.52 |
| 1221(1)´04 | 17.69 | 12.09 | 11.09 | 7.44 | 6.46 | 1.78 | 4.92 | 16.17 | 11.21 | 3.06 |
|  | 60.79 | 41.55 | 38.11 | 25.57 | 22.20 | 6.12 | 16.91 | 55.57 | 38.52 | 10.52 |
| 1227(1)´04 | 16.37 | 11.65 | 11.67 | 7.59 | 7.39 | 3.11 | 4.22 | 13.20 | 8.91 | – |
|  | 57.64 | 41.02 | 41.09 | 26.73 | 26.02 | 10.95 | 14.86 | 46.48 | 31.37 | – |
| 1228(1)´04 | 19.06 | 13.00 | 12.27 | 7.66 | 8.08 | 2.36 | 5.72 | 16.95 | 11.21 | 3.24 |
|  | 60.89 | 41.53 | 39.20 | 24.47 | 25.81 | 7.54 | 18.27 | 54.15 | 35.81 | 10.35 |
| 1237R(1)´04 | 18.99 | 12.30 | 12.40 | 7.89 | 7.88 | 2.16 | 5.59 | 16.70 | 11.07 | 3.02 |
|  | 60.87 | 39.42 | 39.74 | 25.29 | 25.26 | 6.92 | 17.92 | 53.53 | 35.48 | 9.68 |
| **1228(1)/1237R(1)´04** | **19.06** | **13.00** | **12.40** | **7.89** | **8.08** | **2.36** | **5.72** | **16.95** | **11.21** | **3.24** |
|  | **60.89** | **41.53** | **39.62** | **25.21** | **25.81** | **7.54** | **18.27** | **54.15** | **35.81** | **10.35** |
| 1233´04 | – | – | – | – | – | – | 5.06 | 14.34 | 9.23 | 2.59 |
|  | – | – | – | – | – | – | 18.40 | 52.15 | 33.56 | 9.42 |
| 1234(1)´04 | 16.78 | 11.11 | 11.23 | 7.83 | 6.71 | – | – | – | 9.84 | 2.92 |
|  | 60.58 | 40.11 | 40.54 | 28.27 | 24.22 | – | – | – | 35.52 | 10.54 |
| **1233/1234(1)´04** | **16.78** | **11.11** | **11.23** | **7.83** | **6.71** | – | **5.06** | **14.34** | **9.84** | **2.92** |

S1 Table. (Continued)

|  | **60.58** | | **40.11** | | **40.54** | | **28.27** | | **24.22** | | – | | **18.27** | | **51.77** | | **35.52** | | **10.54** |
| --- | --- | --- | --- | --- | --- | --- | --- | --- | --- | --- | --- | --- | --- | --- | --- | --- | --- | --- | --- |
| 1234(2)´04 | 17.33 | | 10.96 | | 10.05 | | 6.56 | | 6.55 | | 2.14 | | 4.67 | | 15.10 | | 10.13 | | 3.44 |
|  | 61.02 | | 38.59 | | 35.39 | | 23.10 | | 23.06 | | 7.54 | | 16.44 | | 53.17 | | 35.67 | | 12.11 |
| 1234R´04 | 17.46 | | 12.73 | | 11.10 | | 7.59 | | 7.61 | | 2.11 | | 5.37 | | 15.40 | | 9.99 | | 2.65 |
|  | 61.70 | | 44.98 | | 39.22 | | 26.82 | | 26.89 | | 7.46 | | 18.98 | | 54.42 | | 35.30 | | 9.36 |
| 1236(1)´04 | 16.39 | | 11.70 | | 11.40 | | 7.25 | | 7.68 | | 3.04 | | 4.44 | | 13.44 | | 9.09 | | 2.30 |
|  | 56.71 | | 40.48 | | 39.45 | | 25.09 | | 26.57 | | 10.52 | | 15.36 | | 46.51 | | 31.45 | | 7.96 |
| 1237(1)´04 | 16.17 | | 11.57 | | 10.70 | | 6.78 | | 6.25 | | 2.15 | | 4.04 | | 14.03 | | 9.85 | | 2.90 |
|  | 57.75 | | 41.32 | | 38.21 | | 24.21 | | 22.32 | | 7.68 | | 14.43 | | 50.11 | | 35.18 | | 10.36 |
| 1237(2)´04 | 17.97 | | 11.27 | | 10.56 | | 7.19 | | 7.69 | | 2.31 | | 5.38 | | 15.43 | | 9.97 | | 2.99 |
|  | 65.35 | | 40.98 | | 38.40 | | 26.15 | | 27.96 | | 8.40 | | 19.56 | | 56.11 | | 36.25 | | 10.87 |
| 1324´04 | 16.29 | | 12.20 | | 11.76 | | 7.66 | | 7.05 | | 2.21 | | 4.86 | | 14.06 | | 9.12 | | 2.81 |
|  | 58.60 | | 43.88 | | 42.30 | | 27.55 | | 25.36 | | 7.95 | | 17.48 | | 50.58 | | 32.81 | | 10.11 |
| 1325´04 | 15.31 | | – | | 10.75 | | 7.34 | | 6.36 | | 2.00 | | 4.30 | | 13.11 | | 8.80 | | 2.46 |
|  | 56.91 | | – | | 39.96 | | 27.29 | | 23.64 | | 7.43 | | 15.99 | | 48.74 | | 32.71 | | 9.14 |
| ID | | lmx | | lH | | wH | |  | |  | |  | |  | |  | |  |  |
| 1142´04 | | 2.09 | | 0.41 | | 0.58 | |  | |  | |  | |  | |  | |  |  |
|  | | 10.00 | | 1.96 | | 2.78 | |  | |  | |  | |  | |  | |  |  |
| 1144´04 | | 3.08 | | 0.22 | | 0.31 | |  | |  | |  | |  | |  | |  |  |
|  | | 13.05 | | 0.93 | | 1.31 | |  | |  | |  | |  | |  | |  |  |
| 1146´04 | | 2.76 | | 0.12 | | 0.24 | |  | |  | |  | |  | |  | |  |  |
|  | | 11.65 | | 0.51 | | 1.01 | |  | |  | |  | |  | |  | |  |  |
| **1144/1146´04** | | **3.08** | | **0.22** | | **0.31** | |  | |  | |  | |  | |  | |  |  |
|  | | **13.00** | | **0.93** | | **1.31** | |  | |  | |  | |  | |  | |  |  |
| 1145´04 | | – | | 0.96 | | 1.17 | |  | |  | |  | |  | |  | |  |  |
|  | | – | | 4.34 | | 5.29 | |  | |  | |  | |  | |  | |  |  |
| 1147´04 | | 1.81 | | 0.47 | | 0.51 | |  | |  | |  | |  | |  | |  |  |
|  | | 8.42 | | 2.19 | | 2.37 | |  | |  | |  | |  | |  | |  |  |
| 1148(1)´04 | | 2.79 | | 1.11 | | 1.22 | |  | |  | |  | |  | |  | |  |  |
|  | | 10.45 | | 4.16 | | 4.57 | |  | |  | |  | |  | |  | |  |  |

S1 Table. (Continued)

| 1149´04 | 2.52 | 0.87 | 0.87 |  |  |  |  |  |  |  |
| --- | --- | --- | --- | --- | --- | --- | --- | --- | --- | --- |
|  | 8.78 | 3.03 | 3.03 |  |  |  |  |  |  |  |
| 1150´04 | 2.61 | 0.98 | 1.12 |  |  |  |  |  |  |  |
|  | 9.32 | 3.50 | 4.00 |  |  |  |  |  |  |  |
| 1151´04 | – | 0.67 | 0.77 |  |  |  |  |  |  |  |
|  | – | 2.90 | 3.33 |  |  |  |  |  |  |  |
|  |  |  |  |  |  |  |  |  |  |  |
| 1152´04 | 2.14 | 0.54 | 0.60 |  |  |  |  |  |  |  |
|  | 9.11 | 2.30 | 2.55 |  |  |  |  |  |  |  |
| **1151/1152´04** | **2.14** | **0.67** | **0.77** |  |  |  |  |  |  |  |
|  | **9.11** | **2.85** | **3.28** |  |  |  |  |  |  |  |
| 1153´04 | – | 0.11 | 0.31 |  |  |  |  |  |  |  |
|  | – | 0.48 | 1.35 |  |  |  |  |  |  |  |
| 1154a´04 | 2.72 | 0.15 | 0.35 |  |  |  |  |  |  |  |
|  | 9.58 | 0.53 | 1.23 |  |  |  |  |  |  |  |
| 1154b´04 | 2.99 | 0.10 | 0.33 |  |  |  |  |  |  |  |
|  | 10.45 | 0.35 | 1.15 |  |  |  |  |  |  |  |
| **1154a/b´04** | **2.99** | **0.15** | **0.35** |  |  |  |  |  |  |  |
|  | **10.45** | **0.52** | **1.22** |  |  |  |  |  |  |  |
| 1155´04 | 2.39 | 1.03 | 1.26 |  |  |  |  |  |  |  |
|  | 9.02 | 3.89 | 4.75 |  |  |  |  |  |  |  |
| 1156´04 | 2.37 | 0.21 | 0.30 |  |  |  |  |  |  |  |
|  | 9.01 | 0.80 | 1.14 |  |  |  |  |  |  |  |
| 1157(1)´04 | 3.26 | 0.36 | 0.37 |  |  |  |  |  |  |  |
|  | 11.68 | 1.29 | 1.33 |  |  |  |  |  |  |  |
| 1158(1)´04 | 3.51 | 0.30 | 0.33 |  |  |  |  |  |  |  |
|  | 12.95 | 1.11 | 1.22 |  |  |  |  |  |  |  |
| **1157(1)/1158(1)´04** | **3.51** | **0.36** | **0.37** |  |  |  |  |  |  |  |
|  | **12.58** | **1.29** | **1.33** |  |  |  |  |  |  |  |
| 1159a(1)´04 | – | 0.95 | 1.09 |  |  |  |  |  |  |  |
|  | – | 3.82 | 4.38 |  |  |  |  |  |  |  |

S1 Table. (Continued)

| 1159b(1)´04 | | – | | 1.00 | | 1.08 | |  | |  | |  | |  | |  | |  |  |
| --- | --- | --- | --- | --- | --- | --- | --- | --- | --- | --- | --- | --- | --- | --- | --- | --- | --- | --- | --- |
|  | | – | | 3.95 | | 4.27 | |  | |  | |  | |  | |  | |  |  |
| **1159a(1)/b(1)´04** | | – | | **1.00** | | **1.09** | |  | |  | |  | |  | |  | |  |  |
|  | | – | | **3.95** | | **4.31** | |  | |  | |  | |  | |  | |  |  |
| 1159a(2)´04 | | – | | 0.82 | | 1.00 | |  | |  | |  | |  | |  | |  |  |
|  | | – | | 2.87 | | 3.50 | |  | |  | |  | |  | |  | |  |  |
| 1159b(2)´04 | | 3.37 | | 0.81 | | 0.89 | |  | |  | |  | |  | |  | |  |  |
|  | | 11.62 | | 2.79 | | 3.07 | |  | |  | |  | |  | |  | |  |  |
| **1159a(2)/b(2)´04** | | **3.37** | | **0.82** | | **1.00** | |  | |  | |  | |  | |  | |  |  |
|  | | **11.62** | | **2.83** | | **3.45** | |  | |  | |  | |  | |  | |  |  |
| 1160a´04 | | – | | 0.96 | | 1.14 | |  | |  | |  | |  | |  | |  |  |
|  | | – | | 3.61 | | 4.29 | |  | |  | |  | |  | |  | |  |  |
| 1161a´04 | | 2.50 | | 0.90 | | 0.88 | |  | |  | |  | |  | |  | |  |  |
|  | | 9.54 | | 3.44 | | 3.36 | |  | |  | |  | |  | |  | |  |  |
| **1160a/61a´04** | | **2.50** | | **0.96** | | **1.14** | |  | |  | |  | |  | |  | |  |  |
|  | | **9.40** | | **3.61** | | **4.29** | |  | |  | |  | |  | |  | |  |  |
| 1160b´04 | | 2.85 | | 1.15 | | 1.01 | |  | |  | |  | |  | |  | |  |  |
|  | | 10.40 | | 4.20 | | 3.69 | |  | |  | |  | |  | |  | |  |  |
| 1161b´04 | | 2.71 | | 1.09 | | 1.23 | |  | |  | |  | |  | |  | |  |  |
|  | | 9.78 | | 3.94 | | 4.44 | |  | |  | |  | |  | |  | |  |  |
| **1160b/1161b´04** | | **2.85** | | **1.15** | | **1.23** | |  | |  | |  | |  | |  | |  |  |
|  | | **10.29** | | **4.15** | | **4.44** | |  | |  | |  | |  | |  | |  |  |
| 1162´04 | | – | | 1.35 | | 1.69 | |  | |  | |  | |  | |  | |  |  |
|  | – | | 4.14 | | 5.18 | |  | |  | |  | |  | |  | |  | |  |
| 1163a(1)´04 | 2.65 | | 0.87 | | 0.99 | |  | |  | |  | |  | |  | |  | |  |
|  | 10.39 | | 3.41 | | 3.88 | |  | |  | |  | |  | |  | |  | |  |
| 1163b(2)´04 | 2.37 | | 0.87 | | 1.14 | |  | |  | |  | |  | |  | |  | |  |
|  | 9.22 | | 3.39 | | 4.44 | |  | |  | |  | |  | |  | |  | |  |
| **1163a(1)/b(2)´04** | **2.65** | | **0.87** | | **1.14** | |  | |  | |  | |  | |  | |  | |  |
|  | **10.31** | | **3.39** | | **4.44** | |  | |  | |  | |  | |  | |  | |  |

S1 Table. (Continued)

| 1163a(2)´04 | 2.43 | 0.33 | 0.35 |  |  |  |  |  |  |  |
| --- | --- | --- | --- | --- | --- | --- | --- | --- | --- | --- |
|  | 10.04 | 1.37 | 1.45 |  |  |  |  |  |  |  |
| 1163b(1)´04 | 2.40 | 0.25 | 0.29 |  |  |  |  |  |  |  |
|  | 9.96 | 1.04 | 1.20 |  |  |  |  |  |  |  |
| **1163a(2)/b(1)´04** | **2.43** | **0.33** | **0.35** |  |  |  |  |  |  |  |
|  | **10.04** | **1.36** | **1.45** |  |  |  |  |  |  |  |
| 1164a´04 | 3.18 | 1.20 | 1.36 |  |  |  |  |  |  |  |
|  | 9.81 | 3.70 | 4.20 |  |  |  |  |  |  |  |
| 1164b´04 | – | – | – |  |  |  |  |  |  |  |
|  | – | – | – |  |  |  |  |  |  |  |
| **1164a/b´04** | **3.18** | **1.20** | **1.36** |  |  |  |  |  |  |  |
|  | **9.81** | **3.70** | **4.20** |  |  |  |  |  |  |  |
| 1165a´04 | – | 1.18 | 1.06 |  |  |  |  |  |  |  |
|  | – | 3.88 | 3.49 |  |  |  |  |  |  |  |
| 1165b´04 | 2.63 | 1.01 | 1.10 |  |  |  |  |  |  |  |
|  | 8.80 | 3.38 | 3.68 |  |  |  |  |  |  |  |
| **1165a/b´04** | **2.63** | **1.18** | **1.10** |  |  |  |  |  |  |  |
|  | **8.65** | **3.88** | **3.62** |  |  |  |  |  |  |  |
| 1166a´04 | 1.89 | 0.48 | 0.55 |  |  |  |  |  |  |  |
|  | 8.96 | 2.27 | 2.61 |  |  |  |  |  |  |  |
| 1166b´04 | 2.46 | 0.53 | 0.58 |  |  |  |  |  |  |  |
|  | 9.39 | 2.02 | 2.21 |  |  |  |  |  |  |  |
| 1167´04 | 3.19 | 0.50 | 0.52 |  |  |  |  |  |  |  |
|  | 11.73 | 1.84 | 1.91 |  |  |  |  |  |  |  |
| 1168´04 | 2.10 | 0.40 | 0.47 |  |  |  |  |  |  |  |
|  | 9.55 | 1.82 | 2.14 |  |  |  |  |  |  |  |
| 1170´04 | – | 0.69 | 0.94 |  |  |  |  |  |  |  |
|  | – | 2.88 | 3.92 |  |  |  |  |  |  |  |
| 1171´04 | 2.81 | 0.77 | 0.96 |  |  |  |  |  |  |  |
|  | 10.18 | 2.79 | 3.48 |  |  |  |  |  |  |  |

S1 Table. (Continued)

| 1172´04 | 2.59 | 0.21 | 0.38 |  |  |  |  |  |  |  |
| --- | --- | --- | --- | --- | --- | --- | --- | --- | --- | --- |
|  | 9.52 | 0.77 | 1.40 |  |  |  |  |  |  |  |
| 1174´04 | 2.57 | 1.09 | 1.12 |  |  |  |  |  |  |  |
|  | 8.77 | 3.72 | 3.82 |  |  |  |  |  |  |  |
| 1175´04 | 2.77 | 0.46 | 0.56 |  |  |  |  |  |  |  |
|  | 10.61 | 1.76 | 2.15 |  |  |  |  |  |  |  |
| 1176a´04 | – | 0.37 | – |  |  |  |  |  |  |  |
|  | – | 1.49 | – |  |  |  |  |  |  |  |
| 1176b´04 | 2.70 | 0.38 | 0.53 |  |  |  |  |  |  |  |
|  | 10.55 | 1.48 | 2.07 |  |  |  |  |  |  |  |
| **1176a/b´04** | **2.70** | **0.38** | **0.53** |  |  |  |  |  |  |  |
|  | **10.55** | **1.48** | **2.07** |  |  |  |  |  |  |  |
| 1177´04 | – | 1.03 | 1.31 |  |  |  |  |  |  |  |
|  | – | 4.04 | 5.14 |  |  |  |  |  |  |  |
| 1178(1)´04 | – | 0.74 | 0.84 |  |  |  |  |  |  |  |
|  | – | 3.15 | 3.57 |  |  |  |  |  |  |  |
| 1180(1)´04 | – | 0.62 | 0.92 |  |  |  |  |  |  |  |
|  | – | 2.58 | 3.83 |  |  |  |  |  |  |  |
| 1181(1)´04 | 2.58 | 1.16 | 1.43 |  |  |  |  |  |  |  |
|  | 8.51 | 3.83 | 4.72 |  |  |  |  |  |  |  |
| 1181(2)´04 | – | 1.13 | 1.25 |  |  |  |  |  |  |  |
|  | – | 3.72 | 4.11 |  |  |  |  |  |  |  |
| 1183(1)´04 | – | 1.17 | 1.21 |  |  |  |  |  |  |  |
|  | – | 3.79 | 3.92 |  |  |  |  |  |  |  |
| **1181(2)/1183(1)´04** | – | **1.17** | **1.25** |  |  |  |  |  |  |  |
|  | **–** | **3.79** | **4.05** |  |  |  |  |  |  |  |
| 1182´04 | 3.03 | 0.69 | 0.73 |  |  |  |  |  |  |  |
|  | 11.10 | 2.53 | 2.67 |  |  |  |  |  |  |  |
| 1184(1)´04 | – | 0.53 | 0.90 |  |  |  |  |  |  |  |
|  | – | 2.05 | 3.49 |  |  |  |  |  |  |  |

S1 Table. (Continued)

| 1185´04 | 2.33 | 1.03 | 1.13 |  |  |  |  |  |  |  |
| --- | --- | --- | --- | --- | --- | --- | --- | --- | --- | --- |
|  | 8.47 | 3.75 | 4.11 |  |  |  |  |  |  |  |
| 1186´04 | 2.53 | 1.16 | 1.49 |  |  |  |  |  |  |  |
|  | 9.10 | 4.17 | 5.36 |  |  |  |  |  |  |  |
| **1185/1186´04** | **2.53** | **1.16** | **1.49** |  |  |  |  |  |  |  |
|  | **9.10** | **4.17** | **5.36** |  |  |  |  |  |  |  |
| 1187´04 | 2.82 | 1.03 | 1.15 |  |  |  |  |  |  |  |
|  | 10.37 | 3.79 | 4.23 |  |  |  |  |  |  |  |
| 1188´04 | 2.40 | 1.21 | 1.58 |  |  |  |  |  |  |  |
|  | 8.45 | 4.26 | 5.56 |  |  |  |  |  |  |  |
| 1189´04 | 1.75 | 0.88 | 0.87 |  |  |  |  |  |  |  |
|  | 7.17 | 3.61 | 3.57 |  |  |  |  |  |  |  |
| 1190´04 | 2.87 | 0.73 | 0.82 |  |  |  |  |  |  |  |
|  | 9.76 | 2.48 | 2.79 |  |  |  |  |  |  |  |
| 1192´04 | 2.26 | 0.95 | 1.02 |  |  |  |  |  |  |  |
|  | 8.01 | 3.37 | 3.62 |  |  |  |  |  |  |  |
| 1192a´05 | 2.76 | 1.28 | 1.53 |  |  |  |  |  |  |  |
|  | 8.79 | 4.08 | 4.87 |  |  |  |  |  |  |  |
| 1192b´05 | 2.59 | 1.32 | 1.65 |  |  |  |  |  |  |  |
|  | 8.22 | 4.19 | 5.24 |  |  |  |  |  |  |  |
| **1192a/b´04** | **2.76** | **1.32** | **1.65** |  |  |  |  |  |  |  |
|  | **8.76** | **4.19** | **5.24** |  |  |  |  |  |  |  |
| 1193´04 | – | 0.98 | 0.94 |  |  |  |  |  |  |  |
|  | – | 3.62 | 3.47 |  |  |  |  |  |  |  |
| 1194´04 | 2.30 | 0.37 | 0.43 |  |  |  |  |  |  |  |
|  | 8.95 | 1.44 | 1.67 |  |  |  |  |  |  |  |
| 1198a´04 | 2.38 | 0.80 | 0.76 |  |  |  |  |  |  |  |
|  | 10.67 | 3.59 | 3.41 |  |  |  |  |  |  |  |
| 1198b´04 | 2.16 | 0.72 | 0.73 |  |  |  |  |  |  |  |
|  | 9.64 | 3.21 | 3.26 |  |  |  |  |  |  |  |

S1 Table. (Continued)

| **1198a/b´04** | **2.38** | **0.80** | **0.76** |  |  |  |  |  |  |  |
| --- | --- | --- | --- | --- | --- | --- | --- | --- | --- | --- |
|  | **10.63** | **3.57** | **3.39** |  |  |  |  |  |  |  |
| 1199a´04 | 2.87 | 0.84 | 0.98 |  |  |  |  |  |  |  |
|  | 10.44 | 3.05 | 3.56 |  |  |  |  |  |  |  |
| 1199b´04 | 2.19 | 0.88 | 0.95 |  |  |  |  |  |  |  |
|  | 8.36 | 3.36 | 3.63 |  |  |  |  |  |  |  |
| **1199a/b´04** | **2.87** | **0.88** | **0.98** |  |  |  |  |  |  |  |
|  | **10.44** | **3.20** | **3.56** |  |  |  |  |  |  |  |
| 1200´04 | – | 1.37 | 1.71 |  |  |  |  |  |  |  |
|  | – | 4.41 | 5.50 |  |  |  |  |  |  |  |
| 1202´04 | 2.37 | 1.09 | 1.04 |  |  |  |  |  |  |  |
|  | 8.01 | 3.68 | 3.51 |  |  |  |  |  |  |  |
| 1203a´05 | 2.69 | 0.93 | 1.01 |  |  |  |  |  |  |  |
|  | 9.41 | 3.25 | 3.53 |  |  |  |  |  |  |  |
| 1203b´05 | 2.50 | 0.97 | 1.07 |  |  |  |  |  |  |  |
|  | 8.74 | 3.39 | 3.74 |  |  |  |  |  |  |  |
| **1203a/b´05** | **2.69** | **0.97** | **1.07** |  |  |  |  |  |  |  |
|  | **9.41** | **3.39** | **3.74** |  |  |  |  |  |  |  |
| 1204´04 | – | 0.96 | – |  |  |  |  |  |  |  |
|  | – | 3.30 | – |  |  |  |  |  |  |  |
| 1204´05 | 1.85 | 0.84 | 0.90 |  |  |  |  |  |  |  |
|  | 9.07 | 4.12 | 4.41 |  |  |  |  |  |  |  |
| 1206(1)´04 | 2.85 | 0.56 | 0.72 |  |  |  |  |  |  |  |
|  | 11.22 | 2.20 | 2.83 |  |  |  |  |  |  |  |
| 1211´04 | – | 0.65 | 0.80 |  |  |  |  |  |  |  |
|  | – | 2.57 | 3.16 |  |  |  |  |  |  |  |
| **1206(1)/1211´04** | **2.85** | **0.65** | **0.80** |  |  |  |  |  |  |  |
|  | **11.22** | **2.56** | **3.15** |  |  |  |  |  |  |  |
| 1209´04 | – | 0.52 | 0.76 |  |  |  |  |  |  |  |
|  | – | 2.06 | 3.00 |  |  |  |  |  |  |  |

S1 Table. (Continued)

| 1209a´05 | 3.68 | 1.39 | 1.49 |  |  |  |  |  |  |  |
| --- | --- | --- | --- | --- | --- | --- | --- | --- | --- | --- |
|  | 10.31 | 3.89 | 4.17 |  |  |  |  |  |  |  |
| 1209b´05 | 2.97 | 1.33 | 1.50 |  |  |  |  |  |  |  |
|  | 8.32 | 3.73 | 4.20 |  |  |  |  |  |  |  |
| **1209a/b´05** | **3.68** | **1.39** | **1.50** |  |  |  |  |  |  |  |
|  | **10.31** | **3.89** | **4.20** |  |  |  |  |  |  |  |
| 1213(1)´04 | 3.25 | 1.14 | 0.92 |  |  |  |  |  |  |  |
|  | 11.28 | 3.96 | 3.19 |  |  |  |  |  |  |  |
| 1215(1)´04 | – | 0.19 | 0.33 |  |  |  |  |  |  |  |
|  | – | 0.78 | 1.35 |  |  |  |  |  |  |  |
| 1217a(1)´04 | – | 0.50 | 0.73 |  |  |  |  |  |  |  |
|  | – | 1.89 | 2.75 |  |  |  |  |  |  |  |
| 1217b(1)´04 | – | – | – |  |  |  |  |  |  |  |
|  | – | – | – |  |  |  |  |  |  |  |
| **1217a(1)/b(1)´04** | **–** | **0.50** | **0.73** |  |  |  |  |  |  |  |
|  | **–** | **1.89** | **2.75** |  |  |  |  |  |  |  |
| 1218´04 | 2.15 | 0.42 | 0.56 |  |  |  |  |  |  |  |
|  | 9.00 | 1.76 | 2.34 |  |  |  |  |  |  |  |
| 1218a´05 | 3.56 | 0.95 | 1.08 |  |  |  |  |  |  |  |
|  | 10.66 | 2.84 | 3.23 |  |  |  |  |  |  |  |
| 1218b´05 | 3.12 | 0.88 | 0.95 |  |  |  |  |  |  |  |
|  | 9.48 | 2.67 | 2.89 |  |  |  |  |  |  |  |
| **1218a/b´05** | **3.56** | **0.95** | **1.08** |  |  |  |  |  |  |  |
|  | **10.66** | **2.84** | **3.23** |  |  |  |  |  |  |  |
| 1219(1)´04 | – | 0.96 | 1.15 |  |  |  |  |  |  |  |
|  | – | 3.75 | 4.49 |  |  |  |  |  |  |  |
| 1220(1)´04 | 2.71 | 0.69 | 1.05 |  |  |  |  |  |  |  |
|  | 9.96 | 2.54 | 3.86 |  |  |  |  |  |  |  |
| 1220R´04 | 2.28 | 0.34 | 0.38 |  |  |  |  |  |  |  |
|  | 9.87 | 1.47 | 1.65 |  |  |  |  |  |  |  |

S1 Table. (Continued)

| 1221(1)´04 | 2.92 | 1.41 | 1.63 |  |  |  |  |  |  |  |
| --- | --- | --- | --- | --- | --- | --- | --- | --- | --- | --- |
|  | 10.03 | 4.85 | 5.60 |  |  |  |  |  |  |  |
| 1227(1)´04 | – | 1.08 | 1.27 |  |  |  |  |  |  |  |
|  | – | 3.80 | 4.47 |  |  |  |  |  |  |  |
| 1228(1)´04 | 3.10 | 1.34 | 1.67 |  |  |  |  |  |  |  |
|  | 9.90 | 4.28 | 5.34 |  |  |  |  |  |  |  |
| 1237R(1)´04 | 2.88 | 1.22 | 1.43 |  |  |  |  |  |  |  |
|  | 9.23 | 3.91 | 4.58 |  |  |  |  |  |  |  |
| **1228(1)/1237R(1)´04** | **3.10** | **1.34** | **1.67** |  |  |  |  |  |  |  |
|  | **9.90** | **4.28** | **5.34** |  |  |  |  |  |  |  |
| 1233´04 | 2.16 | 0.24 | 0.26 |  |  |  |  |  |  |  |
|  | 7.85 | 0.87 | 0.95 |  |  |  |  |  |  |  |
| 1234(1)´04 | 3.06 | 0.18 | 0.36 |  |  |  |  |  |  |  |
|  | 11.05 | 0.65 | 1.30 |  |  |  |  |  |  |  |
| **1233/1234(1)´04** | **3.06** | **0.24** | **0.36** |  |  |  |  |  |  |  |
|  | **11.05** | **0.87** | **1.30** |  |  |  |  |  |  |  |
| 1234(2)´04 | 3.39 | 1.08 | – |  |  |  |  |  |  |  |
|  | 11.94 | 3.80 | – |  |  |  |  |  |  |  |
| 1234R´04 | 2.59 | 0.70 | 0.83 |  |  |  |  |  |  |  |
|  | 9.15 | 2.47 | 2.93 |  |  |  |  |  |  |  |
| 1236(1)´04 | 2.53 | 0.84 | – |  |  |  |  |  |  |  |
|  | 8.75 | 2.91 | – |  |  |  |  |  |  |  |
| 1237(1)´04 | 2.72 | 0.15 | 0.33 |  |  |  |  |  |  |  |
|  | 9.71 | 0.54 | 1.18 |  |  |  |  |  |  |  |
| 1237(2)´04 | – | 0.58 | 0.50 |  |  |  |  |  |  |  |
|  | – | 2.11 | 1.82 |  |  |  |  |  |  |  |
| 1324´04 | 2.99 | 0.79 | 0.95 |  |  |  |  |  |  |  |
|  | 10.76 | 2.84 | 3.42 |  |  |  |  |  |  |  |
| 1325´04 | 2.39 | 1.00 | 1.20 |  |  |  |  |  |  |  |
|  | 8.88 | 3.72 | 4.46 |  |  |  |  |  |  |  |

Bold values indicate the combination of part and counterpart measurements. Abbreviations: GO, gill opening; h, minimum body depth; H, maximum body depth; hA, depth of anal fin; hc, head depth; hD, depth of dorsal fin; lA, length of anal fin; lc, length of head; lC, length of caudal fin; lc/hc, ratio of head length to head depth; LD, length of dorsal fin; lH, length of hypural plate; lmd, length of lower jaw; lmx, length of upper jaw; lP, length of pectoral fin; lPbs, length of pectoral fin base; lpc, length of caudal peduncle; lV, length of pelvic fin; lVbs, length of pelvic fin base; Or, diameter of orbit; pA, preanal distance; pD, predorsal distance; poA, postanal distance; poD, postdorsal distance; pP, prepectoral distance; prO, preorbital distance; pV, prepelvic distance; P–A, distance between pectoral and anal fin base; P–V, distance between pectoral and pelvic fin base; TL, total length; V–A, distance between pelvic and anal fin base; wH, width of hypural plate.
