# Supplementary material for: †Kenyaichthyidae fam. nov. and †Kenyaichthys gen. nov. – First Record of a Fossil Aplocheiloid Killifish (Teleostei, Cyprinodontiformes)
Source: PLoS One. 2015 Apr 29;10(4):e0123056. doi: 10.1371/journal.pone.0123056 (PMC4414574; doi:10.1371/journal.pone.0123056)
Supplement: S2 Table — (DOC) [file pone.0123056.s002.doc]

**S2 Table. Measurements of the premaxilla and maxilla of †*Kenyaichthys kipkechi* sp. nov.**

| ID | SL (mm) | pmx | | mx | |
| --- | --- | --- | --- | --- | --- |
|  |  | mm | % of SL | mm | % of SL |
| 1147´04 | 21.50 | 1.76 | 8.19 | 1.67 | 7.77 |
| 1150´04 | 28.00 | 2.66 | 9.50 | 2.42 | 8.64 |
| 1151/1152´04 | 23.50 | 1.69 | 7.19 | 1.64 | 6.98 |
| 1155´04 | 26.50 | 1.86 | 7.02 | 2.03 | 7.66 |
| 1157(1)/1158(1)´04 | 27.90 | 3.28 | 11.76 | 2.81 | 10.07 |
| 1160a/1161a´04 | 26.60 | 2.24 | 8.42 | 1.98 | 7.44 |
| 1160b/1161b´04 | 27.70 | 2.13 | 7.69 | 2.03 | 7.33 |
| 1171´04 | 27.60 | 2.35 | 8.51 | 2.03 | 7.36 |
| 1172´04 | 27.20 | 2.35 | 8.64 | 2.32 | 8.53 |
| 1174´04 | 29.30 | 2.44 | 8.33 | 2.24 | 7.65 |
| 1190´04 | 29.40 | 2.59 | 8.81 | 2.53 | 8.61 |
| 1192a/b´05 | 31.50 | 2.41 | 8.44 | 2.66 | 7.65 |
| 1198a´04 | 22.40 | 2.17 | 9.69 | 2.14 | 9.55 |
| 1203a/b´05 | 28.60 | 2.37 | 8.29 | 2.28 | 7.97 |
| 1204´05 | 20.40 | 1.75 | 8.58 | 1.90 | 9.31 |
| 1209a/b´05 | 35.70 | 3.46 | 9.69 | 2.86 | 8.01 |
| 1218´04 | 23.90 | 1.70 | 7.11 | 1.78 | 7.45 |
| 1218a/b´05 | 33.40 | 3.24 | 9.70 | 3.06 | 9.16 |
| 1221(1)´04 | 29.10 | 2.79 | 9.59 | 2.24 | 7.70 |
| 1228(1)/1237R(1)´04 | 31.30 | 2.67 | 8.53 | 2.38 | 7.60 |
| 1233/1234(1)´04 | 27.70 | 2.68 | 9.68 | 2.65 | 9.57 |
