# Supplementary material for: †Kenyaichthyidae fam. nov. and †Kenyaichthys gen. nov. – First Record of a Fossil Aplocheiloid Killifish (Teleostei, Cyprinodontiformes)
Source: PLoS One. 2015 Apr 29;10(4):e0123056. doi: 10.1371/journal.pone.0123056 (PMC4414574; doi:10.1371/journal.pone.0123056)
Supplement: S3 Table — (DOC) [file pone.0123056.s003.doc]

**S3 Table. Meristic values of all specimens of †*Kenyaichthys*** gen. et sp. nov.

| ID | | Species | | CFF | | DFR | | AFR | | AV | CV | TV | PO | VFR | PFR | R | PU# | PU Spines | |
| --- | --- | --- | --- | --- | --- | --- | --- | --- | --- | --- | --- | --- | --- | --- | --- | --- | --- | --- | --- |
| 1141´04 | | †*K.* cf. *kipkechi* | | – | | 13+ | | 9+ | | 11 | 18 | 29 | 10 | 4+ | 4+ | 8? | – | – |  |
| 1142´04 | | †*K. kipkechi* | | ?+?+9+11 | | 14 | | 18 | | 11? | 18 | 29? | 10 | 3+ | 10? | 9 or 10 | 5 | NS2+HS2 | D |
| 1143´04 | | †*K.* cf. *kipkechi* | | – | | 14? | | 18 | | 13 | 18 | 31 | 11 | 6 | 11? | 11? | 5 | – |  |
| 1169´04 | | †*K.* cf*. kipkechi* | | – | | 14 | | 18 | | 13 | 18 | 31 | 11 | 6 | 11? | 9+ | 5 | – |  |
| 1144´04 | | †*K. kipkechi* | | 8+9+9+8 | | 14+ | | 20 | | 11 | 18 | 29 | 9 | 6 | 8+ | 9 | 6 | – |  |
| 1146´04 | | †*K. kipkechi* | | 5+9+9+8 | | 14+ | | 21? | | 11 | 18 | 29 | 9 | 6 | 15 | 9 | 6 | – |  |
| 1145´05 | | †*K. kipkechi* | | 12+10+?+? | | 12+ | | 14+ | | 13? | 19 | 32? | 11? | 5? | 8+ | 11? | 6 | – |  |
| 1147´04 | | †*K. kipkechi* | | 10+10+10+9 | | 14 | | 18 | | 12 | 20 | 32 | 10 | 6 | 12+ | 10+ | 6 | – |  |
| 1148(1)´04 | | †*K. kipkechi* | | 13+9+?+? | | 17 | | 18 | | 12 | 19 | 31 | 11 | 5? | 15? | 10 | 6 | – |  |
| 1148(2)´04 | | †*K.* cf*. kipkechi* | | – | | 13 | | 18 | | 3+ | 18 | 21+ | – | 7? | – | 9+ | 5 | NS2+HS2 | D |
| 1149´04 | | †*K. kipkechi* | | 3?+10+?+? | | 11+ | | 19 | | 12 | 19 | 31 | 10 | 5+ | 12? | 10 | 5? | NS3+HS3 | D |
| 1150´04 | | †*K. kipkechi* | | 10+10+10+10? | | 15 | | 18 | | 12? | 19 | 31? | 11 | 5 | 14 | 11? | 5 | NS2+HS2 | D |
| 1151´04 | | †*K. kipkechi* | | – | | 14 or 15 | | 18 | | 12 | 18 | 30 | 10 | 6 | 15 | 11? | 5? | NS2+HS2 | D |
| 1152´04 | | †*K. kipkechi* | | – | | 15 | | 18 | | 12 | 18 | 30 | 10 | 6 | 15 | 11? | 5? | NS2+HS2 | D |
| 1153´04 | | †*K. kipkechi* | | ?+?+9+10 | | 14+ | | 18 | | 11+ | 18+ | 29+ | – | 6? | 12+ | 7+ | 6 | – |  |
| 1154a´04 | | †*K. kipkechi* | | – | | 13 | | 16+ | | 13 | 19 | 32 | 11 | 5 | 13 | 9+ | ? | – |  |
| 1154b´04 | | †*K. kipkechi* | | – | | 13 | | 17 | | 13 | 19 | 32 | 11 | 5 | 13 | 12? | ? | – |  |
| 1155´04 | | †*K. kipkechi* | | – | | 15 | | 19 | | 13 | 19 | 32 | 10 | 7 | 14 | 11 | 6 | – |  |
| 1156´04 | | †*K. kipkechi* | | – | | 12+ | | 13+ | | 12? | 19 | 31? | 10 | 6? | 14 | 10? | ? | – |  |
| 1157(1)´04 | | †*K. kipkechi* | | – | | 16 | | 19 | | 12 | 20 | 32 | 11 | 5 | 15 | 10? | 6 | – |  |
| 1158(1)´04 | | †*K. kipkechi* | | 12+11+?+? | | 16 | | 19 | | 12 | 20 | 32 | 11 | 4+ | 15 | 11 | 6 | – |  |
| 1157(2)´04 | | †*K.* cf*. kipkechi* | | – | | 12+ | | 13+ | | 13 | 6+ | 19+ | 10 | 6 | 14 | 8+ | – | – |  |
| 1158(2)´04 | | †*K.* cf*. kipkechi* | | – | | 13+ | | 10+ | | 13 | – | – | 10 | 5+ | 12+ | 11 | – | – |  |
| 1157R´04 | | †*K.* cf*. kipkechi* | | – | | 16 | | 17 | | – | 19 | 19+ | – | 4+ | 9+ | 8+ | 5 | – |  |
| 1159a(1)´04 | | †*K. kipkechi* | | – | | – | | – | | – | – | – | – | 5? | 12 | 10 | 5 | NS2 | D |
| 1159b(1)´04 | | †*K. kipkechi* | | 11+10+?+? | | – | | – | | – | – | – | – | – | 5+ | 7+ | 5 | NS2 | D |
| 1159a(2)´04 | †*K. kipkechi* | | – | | 13 | | 17 | | 12 | | 17 | 29 | 10 | 6 | 15 | 9 | 6 | – |  |
| 1159b(2)´04 | †*K. kipkechi* | | – | | 13 | | 17 | | 12 | | 17 | 29 | 10 | 5 or 6 | 15 | 9 | 6 | – |  |
| 1160a´04 | †*K. kipkechi* | | – | | 16 | | 16+ | | 12 | | 19 | 31 | 11 | 6 | 6+ | 10 | 5 | – |  |

S3 Table. (Continued)

| 1161a´04 | | †*K. kipkechi* | – | 16 | 18 | 12 | 19 | 31 | 11 | 4+ | 11+ | 10 | 5 | – |  |
| --- | --- | --- | --- | --- | --- | --- | --- | --- | --- | --- | --- | --- | --- | --- | --- |
| 1160b´04 | | †*K.* cf*. kipkechi* | ?+?+8+12 | 14 | 17? | 12 | 18 | 30 | 10 | 6 | 13? | 11 | 5 | – |  |
| 1161b´04 | | †*K.* cf*. kipkechi* | ?+?+8+13 | 14 | 18 | 12 | 18 | 30 | 10 | 6 | 10+ | 10+ | 5 | – |  |
| 1162´04 | | †*K.* cf*. kipkechi* | 11+10+10+15 | 16 | 22 | 12 | 18 | 30 | 9 | 6 | 16? | 10? | 6 | – |  |
| 1163a(1)´04 | | †*K. kipkechi* | – | 14 | 17+ | 13 | 19 | 32 | 11 | 6 | 14 | 11? | 5 | NS3 | S |
| 1163b(2)´04 | | †*K. kipkechi* | – | 14 | 14+ | 13 | 19 | 32 | 11 | 6 or 7 | 14 | 11 | 5 | NS3 | S |
| 1163a(2)´04 | | †*K. kipkechi* | 11+10+?+? | 14 | 18 | 13 | 20 | 33 | 11 | 5 or 6 | 12 | 11 | 6 | – |  |
| 1163b(1)´04 | | †*K. kipkechi* | 10+10+?+? | 14 | 18 | 13 | 20 | 33 | 11 | 6 | 12 | 11 | 6 | – |  |
| 1164a´04 | | †*K. kipkechi* | 9+10+?+? | 10+ | 20 | 13 | 18 | 31 | 11 | 6 or 7 | 16 | 12 | 5 | NS2+HS2 | D |
| 1164b´04 | | †*K. kipkechi* | 6+10+?+? | 11+ | 20 | 13 | 18 | 31 | 11 | 7 | 16 | 12 | 5 | NS2+HS2 | D |
| 1165a´04 | | †*K. kipkechi* | 9?+11+?+? | 16 | 18 | 12 | 19 | 31 | 11 | 6? | 14 | 10? | 5? | – |  |
| 1165b´04 | | †*K. kipkechi* | – | 14+ | 13+ | 12 | 19 | 31 | 11 | 5? | 10+ | 10? | 5? | – |  |
| 1166a´04 | | †*K. kipkechi* | ?+?+10+10 | 14 | 18 | 13 | 20 | 33 | 11 | 6 or 7 | 15 | 10+ | 6 | – |  |
| 1166b´04 | | †*K. kipkechi* | – | 15 | 20 | 12 | 19 | 31 | 10 | 7 | 14 | 11 | 5 | – |  |
| 1167´04 | | †*K. kipkechi* | – | 15 | 18 | 12 | 18 | 30 | 11 | 6 | 14 | 11 | 5 | NS2 | D |
| 1168´04 | | †*K. kipkechi* | – | 16 | 19 | 13 | 20 | 33 | 11 | 6? | 16 | 11 | 6 | – |  |
| 1170´04 | | †*K. kipkechi* | 12+9+9+11 | 15 | 17 | 9+ | 19 | 28+ | – | – | 8+ | 9 | 6 | HS2 | S |
| 1171´04 | | †*K. kipkechi* | – | 14 | 13+ | 13 | 19 | 32 | 11 | 6 | 4+ | 10? | 5? | – |  |
| 1171R´04 | | †*K.* cf*. kipkechi* | – | 15 | – | 6+ | 18+ | 24+ | – | – | – | 4+ | – | – |  |
| 1172´04 | | †*K. kipkechi* | ?+?+9+9 | 16 | 18 | 13 | 18 | 31 | – | – | 10+ | 7+ | 5 | – |  |
| 1173´04 | | †*K.* cf*. kipkechi* | – | 14 | – | 12 | – | 12+ | 10 | 6? | 13 | 9+ | – | – |  |
| 1174´04 | | †*K. kipkechi* | 13+9+9+9 | 15 | 18 | 13 | 20 | 33 | 12 | 6 or 7 | 16 | 8+ | 6 | – |  |
| 1175´04 | | †*K. kipkechi* | – | 14 | 17 | 12 | 19 | 31 | 8 | 5+ | 14 | 10? | 5 | – |  |
| 1176a´04 | †*K. kipkechi* | | – | 14 | 19 | 12 | 20 | 32 | 10 | 4+ | 10+ | 10? | 6 | – |  |
| 1176b´04 | †*K. kipkechi* | | 12+11+?+? | 14 | 19 | 12 | 20 | 32 | 10 | 5 | 11+ | 11 | 6 | – |  |
| 1177´04 | †*K. kipkechi* | | 13+9+9+10 | 15 | 20 | 12 | 19 | 31 | 10 | 6 or 7 | 16 | 11? | 6 | – |  |
| 1178(1)´04 | †*K. kipkechi* | | – | 15 | 19 | 12? | 18? | 30? | 10? | 5? | – | 9+ | 5? | NS2+HS2 | D |

S3 Table. (Continued)

| 1178(2)´04 | | †*K.* cf*. kipkechi* | – | 16 | 20 | 12 | 18 | 30 | 11 | 4+ | 10+ | 7+ | 5? | – |  |
| --- | --- | --- | --- | --- | --- | --- | --- | --- | --- | --- | --- | --- | --- | --- | --- |
| 1178(3)´04 | | †*K.* cf*. kipkechi* | – | 13 | 14+ | 8+ | 20 | 28+ | – | 6 | – | 4+ | 5? | – |  |
| 1178(4)´04 | | †*K.* cf*. kipkechi* | – | 15 | 8+ | – | – | – | – | – | – | – | – | – |  |
| 1178R´04 | | †*K.* cf*. kipkechi* ***** | – | ? | ? | – | ? | 17+ | – | – | – | – | 5? | ?PU2 | S |
| 1179´04 | | †*K.* cf*. kipkechi* | – | 15 | 19 | 12 | – | 12+ | 10 | 5? | 8+ | 9+ | – | – |  |
| 1180(1)´04 | | †*K. kipkechi* | ?+?+9+15 | 16 | 19 | 13 | 18 | 31 | 11 | 6? | 13 | 7+ | 6 | – |  |
| 1180(2)´04 | | †*K.* cf*. kipkechi* | – | 15 | 20 | 13 | 9+ | 22+ | 11 | 5 | 14 | 10? | – | – |  |
| 1180(3)´04 | | †*K.* cf*. kipkechi* | – | – | – | – | – | 13+ | – | 4+ | 8+ | 8+ | – | – |  |
| 1180(4)´04 | | †*K.* cf*. kipkechi* | – | 12+ | – | 9+ | – | – | – | – | 10+ | 4+ | – | – |  |
| 1180R´04 | | †*K.* cf*. kipkechi* | – | 15 | 20 | – | 18? | 18+ | – | 5 | 11? | 7+ | – | NS2+HS2 | S |
| 1181(1)´04 | | †*K. kipkechi* | 11+9+?+? | 15 | 19 | 12? | 19 | 31? | 10? | 5 or 6 | 14? | 10 | 6 | HS2 | D |
| 1181(2)´04 | | †*K. kipkechi* | ?+?+9+8 | 14 | 16+ | 14? | 18 | 32? | 12? | 4+ | 12? | 10+ | 5 | – |  |
| 1183(1)´04 | | †*K. kipkechi* | 7+9+9+8 | 14 | 16+ | 13+ | 18 | 31+ | – | – | 10+ | 8+ | 5 | – |  |
| 1181(3)´04 | | †*K.* cf*. kipkechi* | – | 14 | 17 | 12? | 16+ | 28+ | 12 | 6? | 12 | 10? | – | – |  |
| 1182´04 | | †*K. kipkechi* | – | 15 | 19 | 12 | 19 | 31 | 11 | 7? | 9+ | 10 | – | – |  |
| 1183(2)´04 | | †*K.* cf*. kipkechi* | – | – | – | 6+ | – | 6+ | – | – | 15 | 5+ | – | – |  |
| 1184(1)´04 | | †*K. kipkechi* | – | 7+ | 14+ | – | 20? | 20+ | – | 5 | 11 | 10 | 6 | – |  |
| 1184(2)´04 | | †*K.* cf*. kipkechi* | – | 4+ | 20 | 13 | 14+ | 27+ | 11 | 5 | 14 | 12? | – | – |  |
| 1184R´04 | | †*K.* cf*. kipkechi* | – | 16 | 10+ | 13? | – | 13+ | 11? | 7 | 14 | 11? | – | – |  |
| 1185´04 | | †*K. kipkechi* | 12+10+9+12 | 14 | 18 | 12 | 19 | 31 | 10 | 6 | 11+ | 9+ | 6 | – |  |
| 1186´04 | | †*K. kipkechi* | 10+10+9+7 | 14 | 18 | 12 | 19 | 31 | 10 | 6 | 11+ | 9+ | 6 | – |  |
| 1187´04 | †*K. kipkechi* | | ?+?+10+10 | 13? | 18 | 13 | 19 | 32 | 11 | 6 | 14 | 9 or 10 | 6 | – |  |
| 1188´04 | †*K. kipkechi* | | – | 14 | 19 | 13 | 18 | 31 | 10 | 6 | 12? | 9 | 5 | NS3 | S |
| 1189´04 | †*K. kipkechi* | | 12+11+10+12 | 13 | 18 | 13 | 19 | 32 | – | – | – | – | 6 | – |  |
| 1190´04 | †*K. kipkechi* | | – | 13 | 17 | 13 | 19 | 32 | 11 | 4+ | 6+ | 8? | 6 | – |  |
| 1191´04 | †*K.* cf*. kipkechi* ***** | | – | – | – | – | ? | 12+ | – | – | – | – | 6 | – |  |
| 1192´04 | †*K. kipkechi* | | 7+8+8+11 | 14+ | 18 | 13 | 19 | 32 | 10 | 5 | 16 | 12 | 5 | – |  |

S3 Table. (Continued)

| 1192a´05 | †*K. kipkechi* | – | 10+ | 15+ | 12 | 19 | 31 | – | – | 11 | 11 or 12 | 5 | NS2 | D |
| --- | --- | --- | --- | --- | --- | --- | --- | --- | --- | --- | --- | --- | --- | --- |
| 1192b´05 | †*K. kipkechi* | – | 10+ | 11+ | 12 | 19 | 31 | – | – | 11 | 11 | 5 | NS2 | D |
| 1193´04 | †*K. kipkechi* | – | 13 | 16 | 13 | 20 | 33 | 10 | 4+ | 12? | 10+ | 6 | – |  |
| 1194´04 | †*K. kipkechi* | – | 13 | 17 | 13 | 19 | 32 | 10 | 5 | 13 | 11 | 6 | – |  |
| 1195(1)´04 | †*K.* cf*. kipkechi* | ?+9?+10+5 | 14 | 19 | 12 | 20 | 32 | 10 | 5? | 9+ | 8+ | 5? | – |  |
| 1195(2)´04 | †*K.* cf*. kipkechi* | – | 11+ | 18 | 14 | 19 | 33 | 12 | 6 | 15 | 9 | 5? | – |  |
| 1196´04 | †*K.* cf*. kipkechi* | – | 13 | 17 | 12 | 19+ | 31+ | – | – | 14 | 10 | – | – |  |
| 1196R´04 | †*K.* cf*. kipkechi* | – | – | – | 13 | 17+ | 30+ | 11 | 6 | 9+ | 8? | – | – |  |
| 1197(1)´04 | †*K.* cf*. kipkechi* | – | 14 | – | 13 | 19 | 32 | 11 | 5 | 8+ | 10 | – | – |  |
| 1197(2)´04 | †*K.* cf*. kipkechi* ***** | – | – | – | – | ? | 19+ | – | – | – | – | 6 | – |  |
| 1198a´04 | †*K. kipkechi* | ?+?+10+12 | 17 | 17 | 13 | 18 | 31 | 11 | 7 | 14 | 12? | 5 | NS2 | S |
| 1198b´04 | †*K. kipkechi* | ?+?+10+12 | 17 | 17 | 13 | 18 | 31 | 11 | 7 | 14 | 12? | 5 | NS2 | S |
| 1199a´04 | †*K. kipkechi* | 12+10+?+? | 14 | 19 | – | 19 | 19+ | – | 6 | 15 | 9 | 5 | NS2 | S |
| 1199b´04 | †*K. kipkechi* | 11+10+?+? | 14 | 17+ | – | 19 | 19+ | – | 6 | 15 | 7+ | 5 | NS2 | S |
| 1199bR´04 | †*K.* cf*. kipkechi* | – | – | – | 9+ | – | – | – | – | 13? | 8+ | – | – |  |
| 1200´04 | †*K. kipkechi* | – | 11+ | 16+ | 13 | 19 | 32 | 10 | 4+ | 16 | 9 | 5 | – |  |
| 1201´04 | †*K.* cf*. kipkechi* | – | 4+ | – | 12? | – | 12+ | – | 4+ | 15 | 10+ | – | – |  |
| 1202´04 | †*K. kipkechi* | – | 15 | 20 | 13? | 18 | 31? | 11? | 6 | 16 | 9+ | 5 | – |  |
| 1203´04 | †*K.* cf*. kipkechi* | – | 11+ | 14+ | 12? | 19 | 31? | 10? | 5 | 6+ | 11? | 6 | – |  |
| 1203a´05 | †*K. kipkechi* | – | 14 | 15+ | 13? | 19? | 32? | 10 | 6 | 12+ | 10? | – | – |  |
| 1203b´05 | †*K. kipkechi* | ?+?+10+8? | 14 | 18 | 13 | 19 | 32 | 10 | 6 | 14 | 10? | 5 | – |  |
| 1204´04 | †*K. kipkechi* | – | 13+ | 17 | 12 | 19 | 31 | 10 | 5? | 12+ | 9? | 5? | – |  |
| 1204´05 | †*K. kipkechi* | – | 14 | 14+ | 12 | 20 | 32 | 9 | 6 | 12 | 10? | 5 | – |  |
| 1205´04 | †*K.* cf*. kipkechi* | – | 11+ | 18? | 13? | 12+ | 25+ | 11? | 4+ | 9+ | 7+ | – | – |  |
| 1206(1)´04 | †*K. kipkechi* | 15+9+9+15 | 15 | 19 | 13 | 19 | 32 | 11 | 6 | 15 | 12? | 6 | NS2 | D |
| 1211´04 | †*K. kipkechi* | 13+9+9+12 | 15 | 19 | 13 | 19 | 32 | 11 | 6 | 15 | 8+ | 6 | NS2 | D |
| 1206(2)´04 | †*K.* cf*. kipkechi* | 10+9+?+? | 15 | 19 | 9+ | 18 | 27+ | – | 6 | 11+ | 8+ | 5 | – |  |

S3 Table. (Continued)

| 1207(1)´04 | †*K.* cf*. kipkechi* | | – | 13 | 16+ | 9+ | 19 | 28+ | – | 5 | – | 8? | 5? | NS2 | S |
| --- | --- | --- | --- | --- | --- | --- | --- | --- | --- | --- | --- | --- | --- | --- | --- |
| 1207(2)´04 | †*K.* cf*. kipkechi* | | – | – | – | – | – | 32? | – | – | – | – | ­­– | – |  |
| 1208´04 | †*K.* cf*. kipkechi* | | – | 15 | 19 | 13 | 19 | 32 | 10 | 4+ | 15 | 9 | 6 | – |  |
| 1209´04 | †*K. kipkechi* | | – | – | 8+ | 12? | 13+ | 25+ | 10? | 5+ | 10+ | 8+ | 5 | NS2+HS2 | D |
| 1209a´05 | †*K. kipkechi* | | ?+?+8+11 | 14 | 18 | 15 | 18 | 33 | 10 | 6 or 7 | 15 | 13 | 5 | NS2+HS2 | D |
| 1209b´05 | †*K. kipkechi* | | – | 11+ | 14+ | 15 | 18 | 33 | 10 | 6 | 15 | 12? | 5 | NS2+HS2 | D |
| 1210a´04 | †*K.* cf*. kipkechi* | | 14+9+9+11 | 14 | 19 | 14 | 19 | 33 | 11 | 5 | 15 | 11 | 5 | – |  |
| 1210b´04 | †*K.* cf*. kipkechi* | | 12+9+9+14 | 14 | 19 | 14 | 19 | 33 | 11 | 5 | 15 | 11 | 5 | – |  |
| 1212a´04 | †*K.* cf*. kipkechi* | | 11+11+?+? | 10+ | 16+ | – | – | – | – | – | – | – | 6 | – |  |
| 1212b´04 | †*K.* cf*. kipkechi* | | ?+?+10+10 | 12+ | 15+ | – | – | – | – | – | – | – | 6 | – |  |
| 1213(1)´04 | †*K. kipkechi* | | – | 13+ | 17 | 13 | 18 | 31 | 11 | 6 | 6+ | 9 | 5? | – |  |
| 1213(2)´04 | †*K.* cf*. kipkechi* | | ?+?+10+12 | 9+ | 15+ | 6+ | – | 6+ | – | 7 | 9+ | 8+ | – | – |  |
| 1214´04 | †*K.* cf*. kipkechi* | | – | 8+ | 10+ | 13? | 19 | 32? | 12? | 5 | 8+ | 12? | 5? | – |  |
| 1215(1)´04 | †*K. kipkechi* | | ?+?+11+15 | 15 | 19 | 12? | 20 | 32? | 11? | 5? | 12 | 10? | 6 | NS2+HS2 | D |
| 1215(2)´04 | †*K.* cf*. kipkechi* | | _ | 15 | 19 | 12? | 19 | 31? | 10? | 5? | 14 | 7+ | 5? | NS2+HS2 | D |
| 1216(1)´04 | †*K.* cf*. kipkechi* | | – | 15 | 17+ | 14? | 20 | 34? | 12 | 4+ | 10+ | 10? | 6 | NS5 | D |
| 1216(2)´04 | †*K.* cf*. kipkechi* ***** | | – | – | – | – | – | 25+ | – | – | – | – | 6 | – |  |
| 1217a(1)´04 | †*K. kipkechi* | | – | 14 | 16+ | 13 | 18 | 31 | 11 | 6? | 11+ | 8+ | 5 | – |  |
| 1217b(1)´04 | †*K. kipkechi* | | – | 14 | 16+ | 13 | 18 | 31 | 11 | 6? | 12+ | 9+ | 5 | – |  |
| 1217a(2)´04 | †*K.* cf*. kipkechi* | | – | 14+ | 19 | 12 | 11+ | 23+ | 11 | 6 | 10+ | 11 | – | – |  |
| 1217b(2)´04 | †*K.* cf*. kipkechi* | | – | – | – | 8+ | – | 8+ | – | – | 13 | 6+ | – | – |  |
| 1217a(3)´04 | †*K.* cf*. kipkechi* | |  | – | – | – | – | – | – | – | – | – | – | – |  |
| 1217b(3)´04 | | †*K.* cf*. kipkechi* |  | – | – | – | – | – | – | – | – | – | – | – |  |
| 1218´04 | | †*K. kipkechi* | 8+10+10+15 | 15 | 18? | 13 | 19 | 32 | 10 | 6? | 13 | 11 | 5 | – |  |
| 1218a´05 | | †*K. kipkechi* | 10+10+?+? | 13 | 17 | – | 18 | 18+ | – | 6 | 9+ | 9+ | 5 | – |  |
| 1218b´05 | | †*K. kipkechi* | 8+10+?+? | 13 | 17 | 13? | 18 | 31? | 12? | 6 | – | 9+ | 5 | – |  |
| 1219(1)´04 | | †*K. kipkechi* | – | 10+ | 17 | 13 | 18 | 31 | 11 | 5 | 15 | 10+ | 5 | HS2 | D |

S3 Table. (Continued)

| 1219(2)´04 | †*K.* cf*. kipkechi* | – | – | 10+ | 13? | 20? | 33? | – | 5 | 9+ | 8+ | 5? | – |  |
| --- | --- | --- | --- | --- | --- | --- | --- | --- | --- | --- | --- | --- | --- | --- |
| 1219(3)´04 | †*K.* cf*. kipkechi* | – | 12+ | 14+ | 6+ | 18 | 24+ | – | 4+ | – | 5+ | 5? | – |  |
| 1219R´04 | †*K.* cf*. kipkechi* | – | 3+ | 14+ | 10+ | 13+ | 23+ | – | – | 6+ | 8+ | – | – |  |
| 1220(1)´04 | †*K. kipkechi* | – | 15? | 19 | 12 | 19 | 31 | 11 | 4+ | 13 | 8+ | 5 | – |  |
| 1220(2)´04 | †*K.* cf*. kipkechi* | ?+?+9+10 | 12+ | 19 | 13 | 19 | 32 | 10 | 5 | 14 | 10 | 5 | – |  |
| 1220R´04 | †*K. kipkechi* | – | 14 | 20 | 13? | 19 | 32? | 12? | 6 | 14 | 11 | 5 | – |  |
| 1221(1)´04 | †*K. kipkechi* | – | 13+ | 18 | 14? | 17 | 31? | 12? | 5 | – | 12 | 5 | – |  |
| 1221(2)´04 | †*K.* cf*. kipkechi* | – | 11+ | 12+ | 7+ | 6+ | 13+ | – | 7? | 13 | 8+ | – | – |  |
| 1221R(1)´04 | †*K. cf. kipkechi* | – | 16 | 17 | 13 | 15+ | 28+ | 11 | 4+ | – | 8+ | – | – |  |
| 1221R(2)´04 | †*K.* cf*. kipkechi* ***** | ? | ? | ? | ? | ? | 23+ | – | – | – | – | 6? | – |  |
| 1221R(3)´04 | †*K.* cf*. kipkechi* | – | – | 8+ | 12 | 3+ | 15+ | 10 | 4+ | 13 | 8+ | – | – |  |
| 1221R(4)´04 | †*K.* cf*. kipkechi* | – | – | – | – | 4+ | 4+ | – | – | – | – | – | – |  |
| 1222(1)´04 | †*K.* cf*. kipkechi* | – | 14 | 17 | 12+ | 19 | 31+ | – | – | 13 | 11? | 5 | NS2+HS2 | D |
| 1222(2)´04 | †*K.* cf*. kipkechi* | – | 13+ | 13+ | 10+ | 12+ | 22+ | – | – | 13 | 6+ | – | – |  |
| 1223´04 | †*K.* cf*. kipkechi* | – | – | – | – | – | – | – | – | – | 4+ | – | – |  |
| 1223R´04 | †*K.* cf*. kipkechi* | – | 15+ | 21 | 13 | 19 | 32 | 11 | 5 | 14 | 8+ | 5? | – |  |
| 1224´04 | †*K.* cf*. kipkechi* | 10+9+?+? | 14 | 19 | 13? | 19? | 32? | 11? | 6? | 10+ | 9+ | 5 | – |  |
| 1224R´04 | †*K.* cf*. kipkechi* | – | 11+ | 16+ | 12 | 12+ | 24+ | 10 | 6? | 14 | 9 | – | – |  |
| 1225´04 | †*K.* cf*. kipkechi* | – | 14+ | 16 | 12 | 16+ | 28+ | 11 | 6 | 9+ | 11 | – | – |  |
| 1232´04 | †*K.* cf*. kipkechi* | – | 13+ | 14+ | 12 | 16+ | 28+ | 11 | 6? | 14? | 10? | – | – |  |
| 1226a(1)´04 | †*K.* cf*. kipkechi* | – | 15 | 20 | 9+ | 19 | 28+ | – | 4+ | 7+ | 6+ | 5 | – |  |
| 1226b(1)´04 | †*K.* cf*. kipkechi* | – | 14+ | 20 | 9+ | 19 | 28+ | – | 4+ | 8+ | 10? | 5 | – |  |
| 1226a(2)´04 | †*K.* cf*. kipkechi* | 12+10+?+? | 15 | – | 7+ | 10+ | 17+ | – | – | – | 5+ | 6 | – |  |
| 1226b(2)´04 | †*K.* cf*. kipkechi* | 11+10+?+? | 15 | 7+ | 12? | 21 | 33? | – | – | 11 | 10+ | 6 | – |  |
| 1227(1)´04 | †*K. kipkechi* | – | 15 | 19 | 13 | 19 | 32 | – | – | 10+ | 9 | 5 | – |  |
| 1227(2)´04 | †*K.* cf*. kipkechi* | – | – | – | 6+ | – | 6+ | – | – | 13? | 4+ | – | – |  |
| 1227(3)´04 | †*K.* cf*. kipkechi* | – | 15 | 13+ | 6+ | 19 | 25+ | – | – | 6+ | 4+ | – | – |  |

S3 Table. (Continued)

| 1228(1)´04 | †*K. kipkechi* | 8+9+9+13 | 15 | 18? | 14 | | 18 | 32 | 11 | 5? | 15 | 11 | 6 | – |  |
| --- | --- | --- | --- | --- | --- | --- | --- | --- | --- | --- | --- | --- | --- | --- | --- |
| 1237R(1)´04 | †*K. kipkechi* | – | 15 | 20 | 14 | | 18 | 32 | 11 | 5? | 15 | 11 | 6 | – |  |
| 1228(2)´04 | †*K.* cf*. kipkechi* | – | 6+ | – | – | | – | 16+ | – | – | – | – | – | – |  |
| 1228(3)´04 | †*K.* cf*. kipkechi* | 10+9+9+8 | 7+ | 10+ | – | | 18? | 18+ | – | – | – | 5+ | 5? | – |  |
| 1228R´04 | †*K.* cf*. kipkechi* | – | 13 | 18 | 14 | | 8+ | 22+ | 11 | 6 | 14 | 10? | – | – |  |
| 1229´04 | †*K.* cf*. kipkechi* | – | 15 | 18 | 13 | | 17+ | 30+ | 11 | 6 | 13 | 9+ | – | – |  |
| 1229R(1)´04 | †*K.* cf*. kipkechi* | – | 14 | 8+ | 10+ | | 5+ | 15+ | – | – | 5+ | 4+ | – | – |  |
| 1229R(2)´04 | †*K.* cf*. kipkechi* | – | – | – | – | | 8+ | 8+ | – | – | – | – | – | – |  |
| 1229R(3)´04 | †*K.* cf*. kipkechi* | – | – | – | 6+ | | – | 6+ | – | – | – | – | – | – |  |
| 1230a´04 | †*K.* cf*. kipkechi* | – | – | – | 6+ | | – | 6+ | – | – | – | – | – | – |  |
| 1230b´04 | †*K.* cf*. kipkechi* | – | – | – | 6+ | | – | 6+ | – | – | – | – | – | – |  |
| 1231´04 | †*K.* cf*. kipkechi* | – | 14 | 19 | 12 | | 11+ | 23+ | 10 | 5? | 12? | 10 | – | – |  |
| 1233´04 | †*K. kipkechi* | – | – | – | – | | – | – | – | 3+ | 13 | 8+ | – | – |  |
| 1234(1)´04 | †*K. kipkechi* | – | 10+ | 12+ | 12 | | 19 | 31 | – | – | 13 | 9+ | – | – |  |
| 1234(2)´04 | †*K. kipkechi* | – | 10+ | 17 | 13 | | 19 | 32 | 12 | 5+ | 13 | 9+ | 5 | NS2 | D |
| 1234R´04 | †*K. kipkechi* | – | 12+ | 17 | 11 | | 19 | 30 | 9 | 4+ | 11+ | 8 | 5? | – |  |
| 1235(1)´04 | †*K.* cf*. kipkechi* | – | – | 9+ | 12? | | 3+ | 15+ | 10? | 4+ | 13 | 8+ | – | – |  |
| 1235(2)´04 | †*K.* cf*. kipkechi* | – | 14 | 14+ | – | | 18+ | 18+ | 10? | – | – | – | – | – |  |
| 1236(1)´04 | †*K. kipkechi* | – | 14 | 18 | 13 | | 19 | 32 | 10 | 5+ | 13 | 10 | 5 | – |  |
| 1236(2)´04 | †*K.* cf*. kipkechi* | – | 14 | 17 | 11 | | 19 | 30 | 10 | 6 or 7 | 13 | 8? | 6 | – |  |
| 1236(3)´04 | †*K.* cf*. kipkechi* | – | – | – | 9+ | | – | 9+ | – | – | 14 | 6+ | – | – |  |
| 1236(4)´04 | †*K.* cf*. kipkechi* | – | 10+ | 17? | 13 | | 19 | 32 | 12 | 4+ | 10+ | 7? | 6 | – |  |
| 1236R(1)´04 | †*K.* cf*. kipkechi* | – | 14 | 7+ | 12 | 18 | | 30 | 10 | 4+ | 7+ | 9 | – | – |  |
| 1236R(2)´04 | †*K.* cf*. kipkechi* | – | 11+ | 17 | 7+ | 19 | | 26+ | – | – | – | 7+ | 5 | – |  |
| 1236R(3)´04 | †*K.* cf*. kipkechi* | – | 13 | 14+ | 12? | 18+ | | 30+ | – | – | 14 | – | 5 | – |  |
| 1236R(4)´04 | †*K.* cf*. kipkechi* | – | 16+ | 20 | 11? | 18 | | 29? | 9? | 7 | 6+ | 8+ | 5 | NS2+HS2 | D |
| 1236R(5)´04 | †*K.* cf*. kipkechi* | – | 15 | 19 | 12 | 15+ | | 27+ | 10 | 6? | 13 | 5+ | – | – |  |

S3 Table. (Continued)

| 1236R(6)´04 | †*K.* cf*. kipkechi* | – | – | – | – | – | – | – | – | – | 5+ | – | – |  |
| --- | --- | --- | --- | --- | --- | --- | --- | --- | --- | --- | --- | --- | --- | --- |
| 1237(1)´04 | †*K. kipkechi* | – | 13 | 20 | 12 | 19 | 31 | 10 | 6? | 15 | 10 | 5 | – |  |
| 1237(2)´04 | †*K. kipkechi* | – | 14 | 17 | 13 | 19 | 32 | 10 | 5 | 16 | 8 | 6 | – |  |
| 1237(3)´04 | †*K.* cf*. kipkechi* ***** | – | ? | ? | – | 15+ | 15+ | – | – | – | – | 5 | ?PU2 | S |
| 1237(4)´04 | †*K.* cf*. kipkechi* | – | 14 | 11+ | 4+ | 14+ | 18+ | – | 5 or 6 | – | 4+ | – | – |  |
| 1237(5)´04 | †*K.* cf*. kipkechi* | – | 13? | 10+ | 14 | 18 | 32 | 10 | 5 | 7+ | 8 | 5? | – |  |
| 1237(6)´04 | †*K.* cf*. kipkechi* | – | 12+ | 17 | 13 | 19 | 32 | 10 | 7? | 8+ | 11 | 5 | – |  |
| 1237(7)´04 | †*K.* cf*. kipkechi* | – | 13 | 18 | 13 | 20 | 33 | 11 | 5? | 8+ | 9 | 5? | – |  |
| 1237(8)´04 | †*K.* cf*. kipkechi* | – | – | – | – | – | – | – | – | 9+ | 3+ | – | – |  |
| 1237R(2)´04 | †*K.* cf*. kipkechi* | ?+?+9+9 | 14 | 20 | 12? | 20 | 32? | 11? | 6? | 14 | 11 | 6 | NS5 | D |
| 1237R(3)´04 | †*K.* cf*. kipkechi* | – | – | – | – | 10+ | 10+ | – | – | – | – | 5? | – |  |
| 1324´04 | †*K. kipkechi* | – | 15 | 19 | 12 | 19 | 31 | 10 | 6 | 14 | 10 | 6 | – |  |
| 1325´04 | †*K. kipkechi* | 10+10+10+8 | 11+ | 19 | 13 | 19 | 32 | 11 | 4+ | 12? | 9+ | 5 | NS2+HS2 | D |

Abbreviations: AFR, anal fin rays; AV, abdominal vertebrae; CFF, caudal fin formula; CV, caudal vertebrae; D, duplicated spine; DFR, dorsal fin rays; PFR, pectoral fin rays; PO, pelvic origin; PU#, number of preural vertebrae; R, ribs; S, split spine; TV, total number of vertebrae; VFR, pelvic fin rays; *****, uncertain dorsoventral orientation of specimen.
