# Supplementary material for: †Kenyaichthyidae fam. nov. and †Kenyaichthys gen. nov. – First Record of a Fossil Aplocheiloid Killifish (Teleostei, Cyprinodontiformes)
Source: PLoS One. 2015 Apr 29;10(4):e0123056. doi: 10.1371/journal.pone.0123056 (PMC4414574; doi:10.1371/journal.pone.0123056)
Supplement: S4 Table — (DOC) [file pone.0123056.s004.doc]

**S4 Table. Spine-ratios of †*Kenyaichthys* gen. et sp. nov.**

| ID | Species | NS2 | HS2 | NS2/NS4 | NS2/NS5 | HS2/HS4 | HS2/HS5 | NS3 | HS3 |
| --- | --- | --- | --- | --- | --- | --- | --- | --- | --- |
| 1141´04 | †*K.* cf*. kipkechi* | – | 0.14 | – | – | 1.40 | – | – | 0.13 |
| 1142´04 | †*K. kipkechi* | 0.05 | 0.06 | 1.25 | 1.00 | 1.00 | 1.50 | 0.06 | 0.07 |
| 1144´04 | †*K. kipkechi* | 0.06 | – | 1.20 | 1.50 | – | – | 0.06 | – |
| 1146´04 | †*K. kipkechi* | 0.07 | 0.07 | 1.75 | 1.40 | 3.50 | 1.40 | 0.06 | 0.05 |
| **1144/1146´04** | **†*K. kipkechi*** | **0.07** | **0.07** | **1.40** | **1.40** | **1.75** | **1.17** | **0.06** | **0.05** |
| 1145´04 | †*K. kipkechi* | 0.09 | 0.08 | 1.13 | 1.80 | 1.33 | 1.60 | 0.07 | 0.10 |
| 1147´04 | †*K. kipkechi* | 0.06 | 0.09 | 1.20 | 1.50 | 1.29 | 1.50 | 0.05 | 0.09 |
| 1148(1)´04 | †*K. kipkechi* | 0.13 | 0.16 | – | 1.44 | 1.23 | 2.00 | – | 0.20 |
| 1148(2)´04 | †*K.* cf*. kipkechi* | 0.07 | 0.10 | 1.75 | 1.40 | 2.00 | 1.67 | 0.07 | 0.09 |
| 1150´04 | †*K. kipkechi* | 0.12 | 0.11 | 1.50 | 2.00 | 2.75 | 1.83 | 0.13 | 0.10 |
| 1151´04 | †*K. kipkechi* | 0.10 | 0.10 | 1.11 | 1.67 | – | – | 0.11 | – |
| 1152´04 | †*K. kipkechi* | 0.08 | 0.05 | 2.00 | 1.33 | – | – | 0.10 | 0.09 |
| **1151/1152´04** | **†*K. kipkechi*** | **0.10** | **0.10** | **1.11** | **1.67** | **–** | **–** | **0.11** | **0.09** |
| 1153´04 | †*K. kipkechi* | 0.09 | 0.03 | – | 2.25 | 0.60 | 0.60 | 0.06 | 0.08 |
| 1154a´04 | †*K. kipkechi* | – | – | – | – | – | – | 0.06 | – |
| 1154b´04 | †*K. kipkechi* | – | – | – | – | – | – | 0.04 | 0.05 |
| **1154a/b´04** | **†*K. kipkechi*** | **–** | **–** | **–** | **–** | **–** | **–** | **0.06** | **0.05** |
| 1155´04 | †*K. kipkechi* | 0.10 | 0.14 | 1.25 | 1.67 | 1.27 | 1.75 | 0.08 | 0.10 |
| 1156´04 | †*K. kipkechi* | – | – | – | – | – | – | 0.06 | – |
| 1157(1)´04 | †*K. kipkechi* | 0.11 | 0.07 | 1.38 | 1.38 | 1.17 | 0.64 | 0.09 | – |
| 1158(1)´04 | †*K. kipkechi* | 0.09 | – | 2.25 | 1.13 | – | – | 0.08 | – |
| **1157(1)/1158(1)´04** | **†*K. kipkechi*** | **0.11** | **0.07** | **1.38** | **1.38** | **1.00** | **0.64** | **0.09** | **–** |
| 1157R´04 | †*K.* cf*. kipkechi* | 0.05 | 0.05 | 1.25 | 1.25 | 1.00 | 1.25 | 0.04 | 0.05 |
| 1159a(1)´04 | †*K. kipkechi* | 0.15 | 0.12 | 1.88 | 2.14 | 1.09 | 2.00 | 0.12 | 0.10 |
| 1159b(1)´04 | †*K. kipkechi* | 0.10 | 0.08 | 1.43 | 1.43 | 1.33 | 1.60 | – | 0.08 |
| **1159a(1)/b(1)´04** | **†*K. kipkechi*** | **0.15** | **0.12** | **1.88** | **2.14** | **1.09** | **2.00** | **0.12** | **0.10** |
| 1159a(2)´04 | †*K*. *kipkechi* | 0.13 | 0.12 | 1.86 | 1.44 | 1.09 | 1.33 | 0.12 | 0.10 |
| 1159b(2)´04 | †*K. kipkechi* | 0.08 | 0.12 | 1.33 | 0.89 | 1.09 | 1.50 | 0.12 | 0.07 |

S4 Table. (Continued)

| **1159a(2)/b(2)´04** | **†*K*. *kipkechi*** | **0.13** | **0.12** | **1.86** | **1.44** | **1.09** | **1.33** | **0.12** | **0.10** |
| --- | --- | --- | --- | --- | --- | --- | --- | --- | --- |
| 1160a´04 | †*K*. *kipkechi* | 0.13 | 0.14 | 1.18 | 1.63 | 1.17 | 1.56 | 0.17 | 0.14 |
| 1161a´04 | †*K*. *kipkechi* | 0.10 | 0.10 | 1.00 | 1.11 | 2.00 | 1.25 | 0.11 | 0.08 |
| **1160a/1161a´04** | **†*K*. *kipkechi*** | **0.13** | **0.14** | **1.18** | **1.44** | **1.17** | **1.56** | **0.17** | **0.14** |
| 1160b´04 | †*K*. *kipkechi* | 0.15 | 0.16 | 5.00 | 3.75 | 2.00 | 3.20 | 0.11 | 0.13 |
| 1161b´04 | †*K*. *kipkechi* | 0.08 | 0.17 | 1.14 | 1.60 | 2.43 | 2.83 | 0.11 | 0.15 |
| **1160b/1161b´04** | **†*K*. *kipkechi*** | **0.15** | **0.17** | **2.14** | **3.00** | **2.13** | **2.83** | **0.11** | **0.15** |
| 1162´04 | †*K*. *kipkechi* | 0.17 | 0.19 | 1.31 | 1.89 | 1.46 | 2.11 | 0.14 | 0.18 |
| 1163a(1)´04 | †*K*. *kipkechi* | 0.11 | 0.09 | 2.75 | 1.57 | 0.82 | 1.29 | 0.08 | 0.11 |
| 1163b(2)´04 | †*K*. *kipkechi* | 0.04 | 0.09 | 0.80 | 0.80 | 1.50 | 1.29 | 0.06 | 0.09 |
| **1163a(1)/b(2)´04** | **†*K*. *kipkechi*** | **0.11** | **0.09** | **2.20** | **1.57** | **0.82** | **1.29** | **0.08** | **0.11** |
| 1163a(2)´04 | †*K*. *kipkechi* | – | 0.04 | – | – | 1.33 | 0.80 | 0.06 | 0.06 |
| 1163b(1)´04 | †*K*. *kipkechi* | 0.04 | 0.09 | 2.00 | 1.00 | 2.25 | 1.50 | 0.04 | 0.07 |
| **1163a(2)/b(1)´04** | **†*K*. *kipkechi*** | **0.04** | **0.09** | **1.33** | **1.00** | **2.25** | **1.50** | **0.06** | **0.07** |
| 1164a´04 | †*K*. *kipkechi* | 0.13 | 0.09 | 2.17 | – | 0.82 | 1.29 | 0.10 | 0.03 |
| 1164b´04 | †*K*. *kipkechi* | 0.13 | 0.15 | 2.60 | – | 1.50 | 2.14 | 0.13 | 0.16 |
| **1164a/b´04** | **†*K*. *kipkechi*** | **0.13** | **0.09** | **2.17** | **–** | **0.82** | **1.29** | **0.10** | **0.11** |
| 1165a´04 | †*K*. *kipkechi* | 0.13 | 0.14 | 4.33 | 1.44 | 2.80 | 1.27 | 0.09 | 0.14 |
| 1165b´04 | †*K*. *kipkechi* | – | 0.07 | – | – | 0.64 | 0.64 | – | 0.10 |
| **1165a/b´04** | **†*K*. *kipkechi*** | **0.13** | **0.14** | **4.33** | **1.44** | **1.27** | **1.27** | **0.09** | **0.14** |
| 1166a´04 | †*K*. *kipkechi* | – | 0.09 | – | – | 1.29 | 1.80 | – | 0.06 |
| 1166b´04 | †*K*. *kipkechi* | 0.04 | – | 1.33 | 0.57 | – | – | 0.04 | 0.03 |
| 1167´04 | †*K*. *kipkechi* | – | – | – | – | – | – | 0.12 | 0.09 |
| 1168´04 | †*K*. *kipkechi* | 0.04 | 0.05 | 1.33 | 0.67 | 2.50 | 0.63 | 0.02 | 0.04 |
| 1170´04 | †*K*. *kipkechi* | 0.08 | 0.09 | 1.14 | 1.33 | 1.50 | 1.29 | 0.08 | 0.06 |
| 1171´04 | †*K*. *kipkechi* | 0.11 | 0.07 | 1.38 | 2.20 | 0.78 | 0.88 | 0.06 | 0.12 |
| 1172´04 | †*K*. *kipkechi* | 0.07 | – | 1.17 | 1.00 | – | – | 0.10 | 0.10 |
| 1174´04 | †*K*. *kipkechi* | 0.14 | 0.09 | 1.27 | 1.27 | 0.69 | 0.75 | 0.09 | 0.14 |
| 1177´04 | †*K. kipkechi* | 0.06 | 0.12 | 0.50 | 0.75 | 1.00 | 1.71 | 0.13 | 0.10 |

S4 Table. (Continued)

| 1178(1)´04 | †*K. kipkechi* | 0.09 | 0.06 | 1.80 | – | 3.00 | 1.00 | 0.08 | 0.04 |
| --- | --- | --- | --- | --- | --- | --- | --- | --- | --- |
| 1178(2)´04 | †*K.* cf*. kipkechi* | 0.12 | – | 2.40 | 1.50 | – | – | 0.07 | – |
| 1178(3)´04 | †*K.* cf*. kipkechi* | 0.07 | – | 1.00 | 1.00 | – | – | 0.11 | – |
| 1180R´04 | †*K.* cf*. kipkechi* | 0.07 | 0.10 | 1.17 | 1.17 | 1.25 | 1.67 | 0.06 | 0.12 |
| 1181(1)´04 | †*K. kipkechi* | 0.09 | 0.12 | 1.13 | 1.29 | 1.71 | 1.50 | 0.10 | 0.09 |
| 1181(2)´04 | †*K. kipkechi* | 0.05 | 0.07 | 0.83 | 0.83 | 1.75 | 0.78 | 0.09 | 0.06 |
| 1183(1)´04 | †*K. kipkechi* | 0.16 | 0.14 | 1.60 | 3.20 | 1.00 | 2.33 | 0.15 | 0.15 |
| **1181(2)/1183(1)´04** | **†*K. kipkechi*** | **0.16** | **0.14** | **1.60** | **2.67** | **1.00** | **1.56** | **0.15** | **0.15** |
| 1182´04 | †*K. kipkechi* | – | 0.07 | – | – | 1.17 | 0.88 | 0.10 | 0.07 |
| 1184(1)´04 | †*K. kipkechi* | 0.08 | 0.07 | 1.14 | 1.60 | 1.40 | 1.75 | 0.05 | 0.07 |
| 1185´04 | †*K. kipkechi* | 0.10 | 0.12 | 1.67 | 1.67 | 1.33 | 1.71 | 0.10 | 0.09 |
| 1186´04 | †*K. kipkechi* | 0.15 | 0.09 | 1.15 | 5.00 | 1.13 | 1.29 | 0.14 | 0.08 |
| **1185/1186´04** | **†*K. kipkechi*** | **0.15** | **0.12** | **1.15** | **2.50** | **1.33** | **1.71** | **0.14** | **0.09** |
| 1187´04 | †*K. kipkechi* | 0.08 | 0.10 | 1.33 | 1.60 | 1.67 | 2.00 | 0.12 | 0.08 |
| 1188´04 | †*K. kipkechi* | 0.14 | 0.10 | 1.17 | 2.00 | 1.43 | 1.43 | 0.13 | 0.09 |
| 1189´04 | †*K. kipkechi* | 0.15 | 0.11 | 1.67 | 3.00 | 1.57 | 2.20 | 0.12 | 0.13 |
| 1190´04 | †*K. kipkechi* | 0.18 | 0.16 | 2.00 | 2.00 | 2.00 | 2.00 | – | 0.09 |
| 1192´04 | †*K. kipkechi* | 0.13 | 0.09 | 2.17 | 2.60 | 1.80 | 1.50 | 0.13 | 0.10 |
| 1192a´05 | †*K. kipkechi* | 0.12 | 0.13 | 1.50 | 1.33 | 1.18 | 1.86 | 0.16 | 0.12 |
| 1192b´05 | †*K. kipkechi* | 0.12 | 0.14 | 1.20 | 1.50 | 1.08 | 2.80 | 0.18 | 0.11 |
| **1192a/b´05** | **†*K. kipkechi*** | **0.12** | **0.14** | **1.20** | **1.33** | **1.08** | **2.00** | **0.18** | **0.12** |
| 1193´04 | †*K. kipkechi* | 0.07 | 0.12 | 1.17 | 1.17 | 1.33 | 2.40 | 0.09 | 0.06 |
| 1194´04 | †*K. kipkechi* | 0.08 | 0.07 | 1.60 | 1.00 | 0.88 | 0.70 | 0.09 | 0.10 |
| 1195(1)´04 | †*K.* cf*. kipkechi* | 0.05 | 0.10 | – | – | – | – | 0.06 | 0.09 |
| 1195(2)´04 | †*K.* cf*. kipkechi* | 0.13 | 0.11 | 1.86 | – | 1.10 | 1.83 | 0.08 | 0.14 |
| 1199a´04 | †*K. kipkechi* | 0.15 | – | 1.50 | 2.14 | – | – | 0.13 | – |
| 1199b´04 | †*K. kipkechi* | 0.16 | 0.12 | 1.60 | 2.67 | 1.50 | 1.50 | 0.19 | 0.09 |
| **1199a/b´04** | **†*K. kipkechi*** | **0.16** | **0.12** | **1.60** | **2.29** | **1.50** | **1.50** | **0.19** | **0.09** |
| 1200´04 | †*K. kipkechi* | 0.17 | 0.16 | 1.70 | 1.89 | 1.78 | 1.45 | 0.14 | 0.18 |

S4 Table. (Continued)

| 1202´04 | †*K. kipkechi* | 0.12 | 0.10 | 2.00 | 2.40 | 1.43 | 1.67 | 0.13 | – |
| --- | --- | --- | --- | --- | --- | --- | --- | --- | --- |
| 1203´04 | †*K.* cf*. kipkechi* | 0.11 | – | – | 1.22 | – | – | 0.12 | – |
| 1203a´05 | †*K. kipkechi* | – | – | – | – | – | – | – | – |
| 1203b´05 | †*K. kipkechi* | 0.07 | 0.11 | 1.40 | 1.00 | 3.67 | 2.20 | 0.08 | 0.05 |
| **1203a/b´05** | **†*K. kipkechi*** | **0.07** | **0.11** | **1.40** | **1.00** | **3.67** | **3.67** | **0.08** | **0.05** |
| 1204´04 | †*K. kipkechi* | 0.22 | – | 2.75 | 3.14 | – | – | 0.20 | – |
| 1204´05 | †*K. kipkechi* | 0.05 | – | 0.63 | 0.71 | – | – | 0.06 | – |
| 1206(1)´04 | †*K. kipkechi* | 0.08 | 0.09 | 0.80 | 1.14 | 1.13 | 1.50 | 0.06 | 0.08 |
| 1211´04 | †*K. kipkechi* | 0.08 | 0.10 | 1.60 | 1.60 | 1.25 | 1.25 | 0.09 | 0.08 |
| **1206(1)/1211´04** | **†*K. kipkechi*** | **0.08** | **0.10** | **0.80** | **1.14** | **1.25** | **1.25** | **0.09** | **0.08** |
| 1206(2)´04 | †*K.* cf*. kipkechi* | 0.09 | 0.13 | 1.50 | 1.50 | 1.18 | 2.60 | 0.09 | 0.13 |
| 1209´04 | †*K. kipkechi* | 0.09 | 0.06 | 1.13 | 1.80 | 1.20 | 1.20 | 0.05 | 0.09 |
| 1209a´05 | †*K. kipkechi* | 0.13 | 0.16 | 1.30 | 1.86 | 1.60 | 2.29 | 0.16 | 0.15 |
| 1209b´05 | †*K. kipkechi* | 0.14 | 0.15 | 1.40 | 2.00 | 1.15 | 2.50 | 0.10 | 0.13 |
| **1209a/b´05** | **†*K. kipkechi*** | **0.14** | **0.16** | **1.40** | **2.00** | **1.23** | **2.29** | **0.16** | **0.15** |
| 1210a´04 | †*K.* cf*. kipkechi* | 0.13 | 0.12 | 1.86 | 2.17 | 1.71 | 1.71 | 0.08 | 0.09 |
| 1210b´04 | †*K.* cf*. kipkechi* | 0.15 | 0.12 | 1.36 | 2.14 | 1.71 | 1.71 | 0.09 | 0.09 |
| **1210a/b´04** | **†*K.* cf*. kipkechi*** | **0.15** | **0.12** | **1.36** | **2.14** | **1.71** | **1.71** | **0.09** | **0.09** |
| 1212a´04 | †*K.* cf*. kipkechi* | 0.10 | 0.09 | 1.00 | 1.43 | 1.29 | 1.29 | 0.13 | 0.06 |
| 1212b´04 | †*K.* cf*. kipkechi* | 0.14 | 0.09 | 1.75 | 1.56 | 1.50 | 1.29 | 0.11 | 0.06 |
| **1212a/b´04** | **†*K.* cf*. kipkechi*** | **0.14** | **0.09** | **1.40** | **1.56** | **1.29** | **1.29** | **0.13** | **0.06** |
| 1213(1)´04 | †*K. kipkechi* | – | 0.09 | – | – | – | – | 0.06 | 0.09 |
| 1213(2)´04 | †*K.* cf*. kipkechi* | – | 0.11 | – | – | 1.10 | 1.57 | – | 0.07 |
| 1214´04 | †*K.* cf*. kipkechi* | – | 0.13 | – | – | 1.18 | – | – | 0.09 |
| 1215(1)´04 | †*K.* cf*. kipkechi* | – | – | – | – | – | – | 0.10 | – |
| 1215(2)´04 | †*K. kipkechi* | 0.10 | 0.11 | 1.25 | 1.25 | 1.57 | 1.57 | 0.08 | 0.10 |
| 1216(1)´04 | †*K.* cf*. kipkechi* | 0.08 | – | 1.14 | 0.80 | – | – | 0.11 | 0.12 |
| 1217a(1)´04 | †*K. kipkechi* | 0.09 | 0.09 | 1.50 | 1.50 | 1.29 | 1.29 | 0.07 | 0.07 |
| 1217b(1)´04 | †*K. kipkechi* | 0.07 | 0.08 | 2.33 | 1.40 | 1.00 | 1.60 | 0.10 | 0.08 |

S4 Table. (Continued)

| **1217a(1)/b(1)´04** | **†*K. kipkechi*** | **0.09** | **0.09** | **1.50** | **1.50** | **1.13** | **1.29** | **0.10** | **0.08** |
| --- | --- | --- | --- | --- | --- | --- | --- | --- | --- |
| 1218´04 | †*K. kipkechi* | 0.08 | – | 1.33 | 1.00 | – | – | 0.07 | 0.08 |
| 1218a´05 | †*K. kipkechi* | 0.08 | 0.10 | 0.89 | 0.89 | 2.50 | 1.43 | 0.05 | 0.07 |
| 1218b´05 | †*K. kipkechi* | 0.11 | 0.09 | 1.57 | 1.38 | 0.82 | 1.29 | 0.14 | 0.10 |
| **1218a/b´05** | **†*K. kipkechi*** | **0.11** | **0.10** | **1.22** | **1.22** | **0.91** | **1.43** | **0.14** | **0.10** |
| 1219(1)´04 | †*K. kipkechi* | 0.11 | 0.12 | 1.10 | 1.83 | 1.20 | 2.00 | 0.12 | 0.16 |
| 1219(2)´04 | †*K.* cf*. kipkechi* | 0.12 | 0.09 | 1.33 | – | 1.29 | 1.29 | 0.09 | 0.09 |
| 1219(3)´04 | †*K.* cf*. kipkechi* | 0.13 | – | 1.86 | 2.60 | – | – | 0.12 | 0.13 |
| 1220(1)´04 | †*K. kipkechi* | 0.10 | 0.13 | 1.43 | 1.43 | 1.86 | 1.63 | 0.10 | 0.14 |
| 1220(2)´04 | †*K.* cf*. kipkechi* | 0.06 | 0.13 | 1.20 | 2.00 | 2.17 | 2.17 | 0.07 | 0.11 |
| 1220R´04 | †*K. kipkechi* | – | – | – | – | – | – | – | 0.06 |
| 1221(1)´04 | †*K. kipkechi* | 0.08 | 0.13 | 2.00 | 1.33 | 2.17 | 1.63 | 0.08 | 0.12 |
| 1222(1)´04 | †*K.* cf*. kipkechi* | 0.13 | 0.14 | 1.63 | – | 2.00 | 1.56 | 0.13 | 0.12 |
| 1223R´04 | †*K.* cf*. kipkechi* | – | 0.13 | – | – | 1.18 | 1.63 | – | 0.14 |
| 1224´04 | †*K.* cf*. kipkechi* | 0.11 | 0.08 | 1.22 | 1.83 | 1.14 | 1.33 | 0.12 | 0.08 |
| 1226a(1)´04 | †*K.* cf*. kipkechi* | 0.11 | 0.12 | 1.57 | 1.57 | 2.40 | 2.00 | 0.12 | 0.09 |
| 1226b(1)´04 | †*K.* cf*. kipkechi* | 0.17 | 0.09 | 2.43 | – | 1.29 | 1.80 | 0.14 | 0.09 |
| **1226a(1)/b(1)´04** | **†*K.* cf*. kipkechi*** | **0.17** | **0.12** | **2.43** | **2.43** | **1.71** | **2.00** | **0.14** | **0.09** |
| 1226a(2)´04 | †*K.* cf*. kipkechi* | 0.11 | 0.14 | 1.57 | 1.83 | 1.27 | 2.00 | 0.12 | 0.10 |
| 1226b(2)´04 | †*K.* cf*. kipkechi* | 0.16 | 0.15 | 2.00 | – | 1.25 | 1.36 | 0.15 | 0.12 |
| **1226a(2)/b(2)´04** | **†*K.* cf*. kipkechi*** | **0.16** | **0.15** | **2.00** | **2.67** | **1.25** | **1.36** | **0.15** | **0.12** |
| 1227(1)´04 | †*K. kipkechi* | 0.17 | – | 1.00 | 2.13 | – | – | 0.14 | 0.12 |
| 1227(3)´04 | †*K.* cf*. kipkechi* | – | – | – | – | – | – | – | 0.09 |
| 1228(1)´04 | †*K. kipkechi* | 0.13 | 0.13 | 1.18 | 2.17 | 1.63 | 2.17 | 0.18 | 0.18 |
| 1237R(1)´04 | †*K. kipkechi* | 0.13 | 0.14 | 0.81 | 3.25 | 1.56 | 2.80 | 0.17 | 0.10 |
| **1228(1)/1237R(1)´04** | **†*K. kipkechi*** | **0.13** | **0.14** | **0.81** | **2.17** | **1.56** | **2.33** | **0.18** | **0.18** |
| 1228(3)´04 | †*K.* cf*. kipkechi* | – | – | – | – | – | – | 0.07 | – |
| 1233´04 | †*K. kipkechi* | 0.08 | – | 1.33 | – | – | – | 0.08 | – |
| 1234(1)´04 | †*K. kipkechi* | – | – | – | – | – | – | – | – |

S4 Table. (Continued)

| **1233/1234(1)´04** | | **†*K. kipkechi*** | | **0.08** | | **–** | **1.33** | **–** | **–** | **–** | **0.08** | **–** |
| --- | --- | --- | --- | --- | --- | --- | --- | --- | --- | --- | --- | --- |
| 1234R´04 | | †*K. kipkechi* | | – | | – | – | – | – | – | – | – |
| 1234(2)´04 | | †*K. kipkechi* | | – | | 0.12 | – | – | 2.40 | 3.00 | – | 0.11 |
| 1236(1)´04 | | †*K. kipkechi* | | 0.18 | | – | 1.80 | 3.00 | – | – | 0.17 | – |
| 1236R(2)´04 | | †*K.* cf*. kipkechi* | | – | | – | – | – | – | – | 0.13 | 0.05 |
| 1236R(3)´04 | | †*K.* cf*. kipkechi* | | 0.10 | | – | 1.11 | 1.25 | – | – | 0.09 | – |
| 1236R(4)´04 | | †*K.* cf*. kipkechi* | | 0.04 | | 0.06 | – | – | 0.86 | 1.20 | 0.05 | 0.07 |
| 1237(1)´04 | | †*K. kipkechi* | | 0.10 | | – | 1.25 | 1.11 | – | – | 0.08 | 0.10 |
| 1237(2)´04 | | †*K. kipkechi* | | 0.08 | | 0.08 | 1.33 | 1.33 | 1.60 | 2.00 | 0.12 | 0.05 |
| 1237(5)´04 | | †*K.* cf*. kipkechi* | | – | | – | – | – | – | – | 0.10 | – |
| 1237(6)´04 | | †*K.* cf*. kipkechi* | | 0.12 | | – | 0.75 | 1.33 | – | – | 0.16 | 0.09 |
| 1237(7)´04 | | †*K.* cf*. kipkechi* | | 0.07 | | – | 1.75 | 1.75 | – | – | 0.06 | – |
| 1237R(2)´04 | | †*K.* cf*. kipkechi* | | 0.08 | | 0.11 | 1.14 | 0.89 | 0.92 | 1.38 | 0.13 | 0.17 |
| 1237R(3)´04 | | †*K.* cf*. kipkechi* | | – | | 0.13 | – | – | 1.18 | 1.44 | – | 0.07 |
| 1324´04 | | †*K. kipkechi* | | 0.09 | | 0.09 | 2.25 | 1.50 | 2.25 | 1.50 | 0.06 | 0.05 |
| 1325´04 | | †*K. kipkechi* | | 0.11 | | 0.10 | 2.20 | 2.20 | 0.91 | 2.00 | 0.06 | 0.11 |
| ID | Species | | NS4 | | HS4 | | NS3/NS5 | HS3/HS5 | NS5 | HS5 |  |  |
| 1141´04 | †*K.* cf*. kipkechi* | | – | | 0.10 | | – | – | – | – |  |  |
| 1142´04 | †*K. kipkechi* | | 0.04 | | 0.06 | | 1.20 | 1.75 | 0.05 | 0.04 |  |  |
| 1144´04 | †*K. kipkechi* | | 0.05 | | 0.04 | | 1.50 | – | 0.04 | 0.06 |  |  |
| 1146´04 | †*K. kipkechi* | | 0.04 | | 0.02 | | 1.20 | 1.00 | 0.05 | 0.05 |  |  |
| **1144/1146´04** | **†*K. kipkechi*** | | **0.05** | | **0.04** | | **1.20** | **0.83** | **0.05** | **0.06** |  |  |
| 1145´04 | †*K. kipkechi* | | 0.08 | | 0.06 | | 1.40 | 2.00 | 0.05 | 0.05 |  |  |
| 1147´04 | †*K. kipkechi* | | 0.05 | | 0.07 | | 1.25 | 1.50 | 0.04 | 0.06 |  |  |
| 1148(1)´04 | †*K. kipkechi* | | – | | 0.13 | | – | 2.50 | 0.09 | 0.08 |  |  |
| 1148(2)´04 | †*K.* cf*. kipkechi* | | 0.04 | | 0.05 | | 1.40 | 1.50 | 0.05 | 0.06 |  |  |
| 1150´04 | †*K. kipkechi* | | 0.08 | | 0.04 | | 2.17 | 1.67 | 0.06 | 0.06 |  |  |
| 1151´04 | †*K. kipkechi* | | 0.09 | | – | | 1.83 | – | 0.06 | – |  |  |
| 1152´04 | †*K. kipkechi* | | 0.04 | | – | | 1.67 | – | 0.06 | – |  |  |

S4 Table. (Continued)

| **1151/1152´04** | **†*K. kipkechi*** | **0.09** | **–** | **1.83** | **–** | **0.06** | **–** |  |  |
| --- | --- | --- | --- | --- | --- | --- | --- | --- | --- |
| 1153´04 | †*K. kipkechi* | – | 0.05 | 1.50 | 1.60 | 0.04 | 0.05 |  |  |
| 1154a´04 | †*K. kipkechi* | 0.05 | 0.04 | – | – | – | 0.08 |  |  |
| 1154b´04 | †*K. kipkechi* | 0.04 | – | 0.80 | – | 0.05 | – |  |  |
| **1154a/b´04** | **†*K. kipkechi*** | **0.05** | **0.04** | **1.20** | **0.63** | **0.05** | **0.08** |  |  |
| 1155´04 | †*K. kipkechi* | 0.08 | 0.11 | 1.33 | 1.25 | 0.06 | 0.08 |  |  |
| 1156´04 | †*K. kipkechi* | 0.05 | – | – | – | – | 0.08 |  |  |
| 1157(1)´04 | †*K. kipkechi* | 0.08 | 0.06 | 1.13 | – | 0.08 | 0.11 |  |  |
| 1158(1)´04 | †*K. kipkechi* | 0.04 | 0.07 | 1.00 | – | 0.08 | 0.10 |  |  |
| **1157(1)/1158(1)´04** | **†*K. kipkechi*** | **0.08** | **0.07** | **1.13** | **–** | **0.08** | **0.11** |  |  |
| 1157R´04 | †*K.* cf*. kipkechi* | 0.04 | 0.05 | 1.00 | 1.25 | 0.04 | 0.04 |  |  |
| 1159a(1)´04 | †*K. kipkechi* | 0.08 | 0.11 | 1.71 | 1.67 | 0.07 | 0.06 |  |  |
| 1159b(1)´04 | †*K. kipkechi* | 0.07 | 0.06 | – | 1.60 | 0.07 | 0.05 |  |  |
| **1159a(1)/b(1)´04** | **†*K. kipkechi*** | **0.08** | **0.11** | **1.71** | **1.67** | **0.07** | **0.06** |  |  |
| 1159a(2)´04 | †*K. kipkechi* | 0.07 | 0.11 | 1.33 | 1.11 | 0.09 | 0.09 |  |  |
| 1159b(2)´04 | †*K. kipkechi* | 0.06 | 0.11 | 1.33 | 0.88 | 0.09 | 0.08 |  |  |
| **1159a(2)/b(2)´04** | **†*K. kipkechi*** | **0.07** | **0.11** | **1.33** | **1.11** | **0.09** | **0.09** |  |  |
| 1160a´04 | †*K. kipkechi* | 0.11 | 0.12 | 2.13 | 1.56 | 0.08 | 0.09 |  |  |
| 1161a´04 | †*K. kipkechi* | 0.10 | 0.05 | 1.22 | 1.00 | 0.09 | 0.08 |  |  |
| **1160a/1161a´04** | **†*K. kipkechi*** | **0.11** | **0.12** | **1.89** | **1.56** | **0.09** | **0.09** |  |  |
| 1160b´04 | †*K. kipkechi* | 0.03 | 0.08 | 2.75 | 2.60 | 0.04 | 0.05 |  |  |
| 1161b´04 | †*K. kipkechi* | 0.07 | 0.07 | 2.20 | 2.50 | 0.05 | 0.06 |  |  |
| **1160b/1161b´04** | **†*K. kipkechi*** | **0.07** | **0.08** | **2.20** | **2.50** | **0.05** | **0.06** |  |  |
| 1162´04 | †*K. kipkechi* | 0.13 | 0.13 | 1.56 | 2.00 | 0.09 | 0.09 |  |  |
| 1163a(1)´04 | †*K. kipkechi* | 0.04 | 0.11 | 1.14 | 1.57 | 0.07 | 0.07 |  |  |
| 1163b(2)´04 | †*K. kipkechi* | 0.05 | 0.06 | 1.20 | 1.29 | 0.05 | 0.07 |  |  |
| **1163a(1)/b(2)´04** | **†*K. kipkechi*** | **0.05** | **0.11** | **1.14** | **1.57** | **0.07** | **0.07** |  |  |
| 1163a(2)´04 | †*K. kipkechi* | 0.03 | 0.03 | 1.50 | 1.20 | 0.04 | 0.05 |  |  |
| 1163b(1)´04 | †*K. kipkechi* | 0.02 | 0.04 | 1.00 | 1.17 | 0.04 | 0.06 |  |  |

S4 Table. (Continued)

| **1163a(2)/b(1)´04** | **†*K. kipkechi*** | **0.03** | **0.04** | **1.5** | **1.17** | **0.04** | **0.06** |  |  |
| --- | --- | --- | --- | --- | --- | --- | --- | --- | --- |
| 1164a´04 | †*K. kipkechi* | 0.06 | 0.11 | – | 0.43 | – | 0.07 |  |  |
| 1164b´04 | †*K. kipkechi* | 0.05 | 0.10 | – | 2.29 | – | 0.07 |  |  |
| **1164a/b´04** | **†*K. kipkechi*** | **0.06** | **0.11** | **–** | **1.57** | **–** | **0.07** |  |  |
| 1165a´04 | †*K. kipkechi* | 0.03 | 0.05 | 1.00 | 1.27 | 0.09 | 0.11 |  |  |
| 1165b´04 | †*K. kipkechi* | – | 0.11 | – | 0.91 | – | 0.11 |  |  |
| **1165a/b´04** | **†*K. kipkechi*** | **0.03** | **0.11** | **1.00** | **1.27** | **0.09** | **0.11** |  |  |
| 1166a´04 | †*K. kipkechi* | – | 0.07 | – | 1.20 | 0.04 | 0.05 |  |  |
| 1166b´04 | †*K. kipkechi* | 0.03 | 0.03 | 0.57 | 0.60 | 0.07 | 0.05 |  |  |
| 1167´04 | †*K. kipkechi* | 0.09 | 0.08 | 2.40 | 2.25 | 0.05 | 0.04 |  |  |
| 1168´04 | †*K. kipkechi* | 0.03 | 0.02 | 0.33 | 0.50 | 0.06 | 0.08 |  |  |
| 1170´04 | †*K. kipkechi* | 0.07 | 0.06 | 1.33 | 0.86 | 0.06 | 0.07 |  |  |
| 1171´04 | †*K. kipkechi* | 0.08 | 0.09 | 1.20 | 1.50 | 0.05 | 0.08 |  |  |
| 1172´04 | †*K. kipkechi* | 0.06 | 0.09 | 1.43 | 1.25 | 0.07 | 0.08 |  |  |
| 1174´04 | †*K. kipkechi* | 0.11 | 0.13 | 0.82 | 1.17 | 0.11 | 0.12 |  |  |
| 1177´04 | †*K. kipkechi* | 0.12 | 0.12 | 1.63 | 1.43 | 0.08 | 0.07 |  |  |
| 1178(1)´04 | †*K. kipkechi* | 0.05 | 0.02 | – | 0.67 | – | 0.06 |  |  |
| 1178(2)´04 | †*K.* cf*. kipkechi* | 0.05 | 0.12 | 0.88 | – | 0.08 | 0.08 |  |  |
| 1178(3)´04 | †*K.* cf*. kipkechi* | 0.07 | – | 1.57 | – | 0.07 | 0.05 |  |  |
| 1180R´04 | †*K.* cf*. kipkechi* | 0.06 | 0.08 | 1.00 | 2.00 | 0.06 | 0.06 |  |  |
| 1181(1)´04 | †*K. kipkechi* | 0.08 | 0.07 | 1.43 | 1.13 | 0.07 | 0.08 |  |  |
| 1181(2)´04 | †*K. kipkechi* | 0.06 | 0.04 | 1.50 | 0.67 | 0.06 | 0.09 |  |  |
| 1183(1)´04 | †*K. kipkechi* | 0.10 | 0.14 | 3.00 | 2.50 | 0.05 | 0.06 |  |  |
| **1181(2)/1183(1)´04** | **†*K. kipkechi*** | **0.10** | **0.14** | **2.50** | **1.67** | **0.06** | **0.09** |  |  |
| 1182´04 | †*K. kipkechi* | 0.08 | 0.06 | 1.25 | 0.88 | 0.08 | 0.08 |  |  |
| 1184(1)´04 | †*K. kipkechi* | 0.07 | 0.05 | 1.00 | 1.75 | 0.05 | 0.04 |  |  |
| 1185´04 | †*K. kipkechi* | 0.06 | 0.09 | 1.67 | 1.29 | 0.06 | 0.07 |  |  |
| 1186´04 | †*K. kipkechi* | 0.13 | 0.08 | 4.67 | 1.14 | 0.03 | 0.07 |  |  |
| **1185/1186´04** | **†*K. kipkechi*** | **0.13** | **0.09** | **2.33** | **1.29** | **0.06** | **0.07** |  |  |

S4 Table. (Continued)

| 1187´04 | †*K. kipkechi* | 0.06 | 0.06 | 2.40 | 1.60 | 0.05 | 0.05 |  |  |
| --- | --- | --- | --- | --- | --- | --- | --- | --- | --- |
| 1188´04 | †*K. kipkechi* | 0.12 | 0.07 | 1.86 | 1.29 | 0.07 | 0.07 |  |  |
| 1189´04 | †*K. kipkechi* | 0.09 | 0.07 | 2.40 | 2.60 | 0.05 | 0.05 |  |  |
| 1190´04 | †*K. kipkechi* | 0.09 | 0.08 | – | 1.13 | 0.09 | 0.08 |  |  |
| 1192´04 | †*K. kipkechi* | 0.06 | 0.05 | 2.60 | 1.67 | 0.05 | 0.06 |  |  |
| 1192a´05 | †*K. kipkechi* | 0.08 | 0.11 | 1.78 | 1.71 | 0.09 | 0.07 |  |  |
| 1192b´05 | †*K. kipkechi* | 0.10 | 0.13 | 2.25 | 2.20 | 0.08 | 0.05 |  |  |
| **1192a/b´05** | **†*K. kipkechi*** | **0.10** | **0.13** | **2.00** | **1.71** | **0.09** | **0.07** |  |  |
| 1193´04 | †*K. kipkechi* | 0.06 | 0.09 | 1.50 | 1.20 | 0.06 | 0.05 |  |  |
| 1194´04 | †*K. kipkechi* | 0.05 | 0.08 | 1.13 | 1.00 | 0.08 | 0.10 |  |  |
| 1195(1)´04 | †*K.* cf*. kipkechi* | – | – | – | – | – | – |  |  |
| 1195(2)´04 | †*K.* cf*. kipkechi* | 0.07 | 0.10 | – | 2.33 | – | 0.06 |  |  |
| 1199a´04 | †*K. kipkechi* | 0.10 | 0.08 | 1.86 | – | 0.07 | 0.08 |  |  |
| 1199b´04 | †*K. kipkechi* | 0.10 | 0.08 | 3.17 | 1.13 | 0.06 | 0.08 |  |  |
| **1199a/b´04** | **†*K. kipkechi*** | **0.10** | **0.08** | **2.71** | **1.13** | **0.07** | **0.08** |  |  |
| 1200´04 | †*K. kipkechi* | 0.10 | 0.09 | 1.56 | 1.64 | 0.09 | 0.11 |  |  |
| 1202´04 | †*K. kipkechi* | 0.06 | 0.07 | 2.60 | – | 0.05 | 0.06 |  |  |
| 1203´04 | †*K.* cf*. kipkechi* | – | – | 1.33 | – | 0.09 | 0.09 |  |  |
| 1203a´05 | †*K. kipkechi* | – | – | – | – | – | – |  |  |
| 1203b´05 | †*K. kipkechi* | 0.05 | 0.03 | 1.14 | 1.00 | 0.07 | 0.05 |  |  |
| **1203a/b´05** | **†*K. kipkechi*** | **0.05** | **0.03** | **1.14** | **1.67** | **0.07** | **0.05** |  |  |
| 1204´04 | †*K. kipkechi* | 0.08 | – | 2.86 | – | 0.07 | – |  |  |
| 1204´05 | †*K. kipkechi* | 0.08 | – | 0.86 | – | 0.07 | 0.07 |  |  |
| 1206(1)´04 | †*K. kipkechi* | 0.10 | 0.08 | 0.86 | 1.33 | 0.07 | 0.06 |  |  |
| 1211´04 | †*K. kipkechi* | 0.05 | 0.08 | 1.80 | 1.00 | 0.05 | 0.08 |  |  |
| **1206(1)/1211´04** | **†*K. kipkechi*** | **0.10** | **0.08** | **1.29** | **1.00** | **0.07** | **0.08** |  |  |
| 1206(2)´04 | †*K.* cf*. kipkechi* | 0.06 | 0.11 | 1.50 | 2.60 | 0.06 | 0.05 |  |  |
| 1209´04 | †*K. kipkechi* | 0.08 | 0.05 | 1.00 | 1.80 | 0.05 | 0.05 |  |  |
| 1209a´05 | †*K. kipkechi* | 0.10 | 0.10 | 2.29 | 2.14 | 0.07 | 0.07 |  |  |

S4 Table. (Continued)

| 1209b´05 | †*K. kipkechi* | 0.10 | 0.13 | 1.43 | 2.17 | 0.07 | 0.06 |  |  |
| --- | --- | --- | --- | --- | --- | --- | --- | --- | --- |
| **1209a/b´05** | **†*K. kipkechi*** | **0.10** | **0.13** | **2.29** | **2.14** | **0.07** | **0.07** |  |  |
| 1210a´04 | †*K.* cf*. kipkechi* | 0.07 | 0.07 | 1.33 | 1.29 | 0.06 | 0.07 |  |  |
| 1210b´04 | †*K.* cf*. kipkechi* | 0.101 | 0.07 | 1.29 | 1.29 | 0.07 | 0.07 |  |  |
| **1210a/b´04** | **†*K.* cf*. kipkechi*** | **0.11** | **0.07** | **1.29** | **1.29** | **0.07** | **0.07** |  |  |
| 1212a´04 | †*K.* cf*. kipkechi* | 0.10 | 0.07 | 1.86 | 0.86 | 0.07 | 0.07 |  |  |
| 1212b´04 | †*K.* cf*. kipkechi* | 0.08 | 0.06 | 1.22 | 0.86 | 0.09 | 0.07 |  |  |
| **1212a/b´04** | **†*K.* cf*. kipkechi*** | **0.1** | **0.07** | **1.44** | **0.86** | **0.09** | **0.07** |  |  |
| 1213(1)´04 | †*K. kipkechi* | 0.08 | – | 1.00 | – | 0.06 | – |  |  |
| 1213(2)´04 | †*K.* cf*. kipkechi* | 0.10 | 0.10 | – | 1.00 | 0.07 | 0.07 |  |  |
| 1214´04 | †*K.* cf*. kipkechi* | – | 0.11 | – | – | – | – |  |  |
| 1215(2)´04 | †*K.* cf*. kipkechi* | 0.08 | 0.07 | 1.00 | 1.43 | 0.08 | 0.07 |  |  |
| 1215(1)´04 | †*K. kipkechi* | 0.09 | 0.05 | 1.43 | – | 0.07 | 0.07 |  |  |
| 1216(1)´04 | †*K.* cf*. kipkechi* | 0.07 | 0.10 | 1.10 | 1.50 | 0.10 | 0.08 |  |  |
| 1217a(1)´04 | †*K. kipkechi* | 0.06 | 0.07 | 1.17 | 1.00 | 0.06 | 0.07 |  |  |
| 1217b(1)´04 | †*K. kipkechi* | 0.03 | 0.08 | 2.00 | 1.60 | 0.05 | 0.05 |  |  |
| **1217a(1)/b(1)´04** | **†*K. kipkechi*** | **0.06** | **0.08** | **1.67** | **1.14** | **0.06** | **0.07** |  |  |
| 1218´04 | †*K. kipkechi* | 0.06 | 0.06 | 0.88 | 1.14 | 0.08 | 0.07 |  |  |
| 1218a´05 | †*K. kipkechi* | 0.09 | 0.04 | 0.56 | 1.00 | 0.09 | 0.07 |  |  |
| 1218b´05 | †*K. kipkechi* | 0.07 | 0.11 | 1.75 | 1.43 | 0.08 | 0.07 |  |  |
| **1218a/b´05** | **†*K. kipkechi*** | **0.09** | **0.11** | **1.56** | **1.43** | **0.09** | **0.07** |  |  |
| 1219(1)´04 | †*K. kipkechi* | 0.10 | 0.10 | 2.00 | 2.67 | 0.06 | 0.06 |  |  |
| 1219(2)´04 | †*K.* cf*. kipkechi* | 0.09 | 0.07 | – | 1.29 | – | 0.07 |  |  |
| 1219(3)´04 | †*K.* cf*. kipkechi* | 0.07 | 0.07 | 2.40 | 1.86 | 0.05 | 0.07 |  |  |
| 1220(1)´04 | †*K. kipkechi* | 0.07 | 0.07 | 1.43 | 1.75 | 0.07 | 0.08 |  |  |
| 1220(2)´04 | †*K.* cf*. kipkechi* | 0.05 | 0.06 | 2.33 | 1.83 | 0.03 | 0.06 |  |  |
| 1220R´04 | †*K. kipkechi* | – | 0.04 | – | 0.86 | – | 0.07 |  |  |
| 1221(1)´04 | †*K. kipkechi* | 0.04 | 0.06 | 1.33 | 1.50 | 0.06 | 0.08 |  |  |
| 1222(1)´04 | †*K.* cf*. kipkechi* | 0.08 | 0.07 | – | 1.33 | – | 0.09 |  |  |

S4 Table. (Continued)

| 1223R´04 | †*K.* cf*. kipkechi* | 0.08 | 0.11 | – | 1.75 | 0.06 | 0.08 |  |  |
| --- | --- | --- | --- | --- | --- | --- | --- | --- | --- |
| 1224´04 | †*K.* cf*. kipkechi* | 0.09 | 0.07 | 2.00 | 1.33 | 0.06 | 0.06 |  |  |
| 1226a(1)´04 | †*K.* cf*. kipkechi* | 0.07 | 0.05 | 1.71 | 1.50 | 0.07 | 0.06 |  |  |
| 1226b(1)´04 | †*K.* cf*. kipkechi* | 0.07 | 0.07 | – | 1.80 | – | 0.05 |  |  |
| **1226a(1)/b(1)´04** | **†*K.* cf*. kipkechi*** | **0.07** | **0.07** | **2.00** | **1.50** | **0.07** | **0.06** |  |  |
| 1226a(2)´04 | †*K.* cf*. kipkechi* | 0.07 | 0.11 | 2.00 | 1.43 | 0.06 | 0.07 |  |  |
| 1226b(2)´04 | †*K.* cf*. kipkechi* | 0.08 | 0.12 | – | 1.09 | – | 0.11 |  |  |
| **1226a(2)/b(2)´04** | **†*K.* cf*. kipkechi*** | **0.08** | **0.12** | **2.50** | **1.09** | **0.06** | **0.11** |  |  |
| 1227(1)´04 | †*K. kipkechi* | 0.17 | 0.07 | 1.75 | 1.33 | 0.08 | 0.09 |  |  |
| 1227(3)´04 | †*K.* cf*. kipkechi* | – | 0.04 | – | 1.50 | 0.06 | 0.06 |  |  |
| 1228(1)´04 | †*K. kipkechi* | 0.11 | 0.08 | 3.00 | 3.00 | 0.06 | 0.06 |  |  |
| 1237R(1)´04 | †*K. kipkechi* | 0.16 | 0.09 | 4.25 | 2.00 | 0.04 | 0.05 |  |  |
| **1228(1)/1237R(1)´04** | **†*K. kipkechi*** | **0.16** | **0.09** | **3.00** | **3.00** | **0.06** | **0.06** |  |  |
| 1228(3)´04 | †*K.* cf*. kipkechi* | 0.05 | 0.08 | 1.40 | – | 0.05 | 0.07 |  |  |
| 1233´04 | †*K. kipkechi* | 0.06 | 0.13 | – | – | – | – |  |  |
| 1234(1)´04 | †*K. kipkechi* | – | – | – | – | – | – |  |  |
| **1233/1234(1)´04** | **†*K. kipkechi*** | **0.06** | **0.13** | **–** | **–** | **–** | **–** |  |  |
| 1234R´04 | †*K. kipkechi* | 0.07 | 0.06 | – | – | – | 0.05 |  |  |
| 1234(2)´04 | †*K. kipkechi* | – | 0.05 | – | 2.75 | – | 0.04 |  |  |
| 1236(1)´04 | †*K. kipkechi* | 0.10 | 0.05 | 2.83 | – | 0.06 | – |  |  |
| 1236R(3)´04 | †*K.* cf*. kipkechi* | 0.09 | – | 1.13 | – | 0.08 | – |  |  |
| 1236R(2)´04 | †*K.* cf*. kipkechi* | 0.05 | 0.04 | 3.25 | 1.25 | 0.04 | 0.04 |  |  |
| 1236R(4)´04 | †*K.* cf*. kipkechi* | – | 0.07 | – | 1.40 | – | 0.05 |  |  |
| 1237(1)´04 | †*K. kipkechi* | 0.08 | 0.05 | 0.89 | – | 0.09 | – |  |  |
| 1237(2)´04 | †*K. kipkechi* | 0.06 | 0.05 | 2.00 | 1.25 | 0.06 | 0.04 |  |  |
| 1237(5)´04 | †*K.* cf*. kipkechi* | 0.06 | – | 1.43 | – | 0.07 | – |  |  |
| 1237(6)´04 | †*K.* cf*. kipkechi* | 0.16 | 0.06 | 1.78 | 1.29 | 0.09 | 0.07 |  |  |
| 1237(7)´04 | †*K.* cf*. kipkechi* | 0.04 | – | 1.50 | – | 0.04 | 0.06 |  |  |
| 1237R(2)´04 | †*K.* cf*. kipkechi* | 0.07 | 0.12 | 1.44 | 2.13 | 0.09 | 0.08 |  |  |

S4 Table. (Continued)

| 1237R(3)´04 | †*K.* cf*. kipkechi* | – | 0.11 | – | 0.78 | – | 0.09 |  |  |
| --- | --- | --- | --- | --- | --- | --- | --- | --- | --- |
| 1324´04 | †*K. kipkechi* | 0.04 | 0.04 | 1.00 | 0.83 | 0.06 | 0.06 |  |  |
| 1325´04 | †*K. kipkechi* | 0.05 | 0.11 | 1.20 | 2.20 | 0.05 | 0.05 |  |  |

Bold values indicate the combination of part and counterpart measurements. Abbreviations: NS, neural spine of preural centrum; HS, haemal spine of preural centrum.
