# Supplementary material for: †Kenyaichthyidae fam. nov. and †Kenyaichthys gen. nov. – First Record of a Fossil Aplocheiloid Killifish (Teleostei, Cyprinodontiformes)
Source: PLoS One. 2015 Apr 29;10(4):e0123056. doi: 10.1371/journal.pone.0123056 (PMC4414574; doi:10.1371/journal.pone.0123056)
Supplement: S5 Table — (DOC) [file pone.0123056.s005.doc]

**S5 Table. Dimensions of key scales of four specimens of †*Kenyaichthys* gen. et sp. nov**.

| ID | Species | SL (mm) | length1 | width1 | length2 | width2 |
| --- | --- | --- | --- | --- | --- | --- |
| 1171R´04 | †*K*. cf. *kipkechi* | – | 0.47 | 0.51 | 0.52 | 0.50 |
| 1199b´4 | †*K. kipkechi* | 26.20 | 0.46 | 0.53 | 0.41 | 0.39 |
| 1223R´04 | †*K*. cf. *kipkechi* | – | 0.36 | 0.37 | 0.38 | 0.36 |
| 1237R(1)´04 | †*K. kipkechi* | 31.20 | 0.43 | 0.44 | 0.45 | 0.37 |
