# Supplementary material for: †Kenyaichthyidae fam. nov. and †Kenyaichthys gen. nov. – First Record of a Fossil Aplocheiloid Killifish (Teleostei, Cyprinodontiformes)
Source: PLoS One. 2015 Apr 29;10(4):e0123056. doi: 10.1371/journal.pone.0123056 (PMC4414574; doi:10.1371/journal.pone.0123056)
Supplement: S6 Table — (DOC) [file pone.0123056.s006.doc]

**S6 Table. Polymorph characters of †*Kenyaichthys* gen. et sp. nov.** and detectability of the neural spine on the first vertebra (NS 1).

| ID | Species | Proximal part of parhypural | Shape of parhypural | Shape of epural | Dorsal fin pattern | NS 1 |
| --- | --- | --- | --- | --- | --- | --- |
| 1141´04 | †*K.* cf*. kipkechi* | reduced | straight | – | 1/2 | – |
| 1142´04 | †*K. kipkechi* | reduced | straight | curved + | 2/2 | √ |
| 1143/1169´04 | †*K.* cf*. kipkechi* | reduced | _ | straight | 2/2 | √ |
| 1144/1146´04 | †*K. kipkechi* | – | – | – | 2/– | – |
| 1145´05 | †*K. kipkechi* | reduced | straight | curved | 1/– | – |
| 1147´04 | †*K. kipkechi* | reduced | straight | straight | 2/2 | √ |
| 1148(1)´04 | †*K. kipkechi* | reduced | straight * | curved | 1/1 | – |
| 1148(2)´04 | †*K.* cf*. kipkechi* | reduced | straight | straight | 1?/– | – |
| 1149´04 | †*K. kipkechi* | – | – | straight | – | – |
| 1150´04 | †*K. kipkechi* | – | _ | _ | 2/1 | – |
| 1151/52´04 | †*K. kipkechi* | reduced | straight | curved | 1/1 | – |
| 1153´04 | †*K. kipkechi* | – | – | – | – | – |
| 1154a/b´04 | †*K. kipkechi* | – | – | – | 1/1 | √ |
| 1155´04 | †*K. kipkechi* | reduced | straight | curved | 2/– | – |
| 1156´04 | †*K. kipkechi* | – | – | – | – | – |
| 1157(1)/1158(1)´04 | †*K. kipkechi* | – | – | curved | 2/2 | √ |
| 1157(2)/1158(2)´04 | †*K.* cf*. kipkechi* | – | – | – | 1/2 | √ |
| 1157R´04 | †*K.* cf*. kipkechi* | – | – | – | 2/1 | – |
| 1159a(1)/b(1)´04 | †*K. kipkechi* | reduced | straight * | curved | – | – |
| 1159a(2)/b(2)´04 | †*K. kipkechi* | reduced + | curved | curved | 1/_ | – |
| 1160a/1161a´04 | †*K. kipkechi* | reduced + | straight | curved | 2/– | √ |
| 1160b/1161b´04 | †*K. kipkechi* | reduced | straight | curved | 1/– | – |
| 1162´04 | †*K. kipkechi* | reduced + | straight | curved | 2/1 | – |
| 1163a(1)/b(2)´04 | †*K. kipkechi* | reduced | straight | curved | 2/– | √ |
| 1163a(2)/b(1)´04 | †*K. kipkechi* | – | – | – | 1/1 | √ |

S6 Table. (Continued)

| 1164a/b´04 | †*K. kipkechi* | reduced + | – * | curved * | – | – |
| --- | --- | --- | --- | --- | --- | --- |
| 1165a/b´04 | †*K. kipkechi* | reduced + | straight | straight | 1/2 | √ |
| 1166a´04 | †*K. kipkechi* | reduced + | straight | curved | 2/1 | √ |
| 1166b´04 | †*K. kipkechi* | reduced | straight | straight | 1/1 | – |
| 1167´04 | †*K. kipkechi* | – | – | – | 1/1 | – |
| 1168´04 | †*K. kipkechi* | reduced | – | curved | 2/3 | – |
| 1170´04 | †*K. kipkechi* | reduced + | straight | straight + | 1/1 | – |
| 1171´04 | †*K. kipkechi* | – | straight | – | – | √ |
| 1171R´04 | †*K.* cf*. kipkechi* | – | – | – | 2/1? | – |
| 1172´04 | †*K. kipkechi* | – | – | – | 2/1 | – |
| 1173´04 | †*K.* cf*. kipkechi* | – | – | – | 2/1? | – |
| 1174´04 | †*K. kipkechi* | reduced | straight | curved | 1/2 | √ |
| 1175´04 | †*K. kipkechi* | – | – | – | 2?/3 | – |
| 1176a/b´04 | †*K. kipkechi* | reduced | – | – | 1/1 | √ |
| 1177´04 | †*K. kipkechi* | reduced | straight ^#^ | straight | 2/1 | – |
| 1178(1)´04 | †*K. kipkechi* | reduced | straight | straight | 2/1 | – |
| 1178(2)´04 | †*K.* cf*. kipkechi* | – | – | – | 2/2 | – |
| 1178(3)´04 | †*K.* cf*. kipkechi* | – | – | curved + | 2/2 | – |
| 1178(4)´04 | †*K.* cf*. kipkechi* | – | – | – | 2/2 | – |
| 1178R´04 | †*K.* cf*. kipkechi* **°** | ? | ? | ? | – | – |
| 1179´04 | †*K.* cf*. kipkechi* | – | – | – | 1/1 | √ |
| 1180(1)´04 | †*K. kipkechi* | reduced | straight | straight | 1/2 | – |
| 1180(2)´04 | †*K.* cf*. kipkechi* | – | – | – | 2/2 | – |
| 1180(3)´04 | †*K.* cf*. kipkechi* | – | – | – | – | – |
| 1180(4)´04 | †*K.* cf*. kipkechi* | – | – | – | 1/1 | – |
| 1180R´04 | †*K.* cf*. kipkechi* | reduced + | straight | curved | 1/– | – |
| 1181(1)´04 | †*K. kipkechi* | reduced + | straight | curved | 2– | – |

S6 Table. (Continued)

| 1181(2)/1183(1)´04 | †*K. kipkechi* | reduced + | curved | curved | 2/1 | – |
| --- | --- | --- | --- | --- | --- | --- |
| 1181(3)´04 | †*K.* cf*. kipkechi* | – | – | – | 2/1 | – |
| 1182´04 | †*K. kipkechi* | reduced | curved | curved + | 1/2 | – |
| 1183(2)´04 | †*K.* cf*. kipkechi* | – | – | – | – | – |
| 1184(1)´04 | †*K. kipkechi* | – | straight | straight + | – | – |
| 1184(2)´04 | †*K.* cf*. kipkechi* | – |  |  | – | – |
| 1184R´04 | †*K.* cf*. kipkechi* | – |  |  | 2/1? | – |
| 1185/1186´04 | †*K. kipkechi* | reduced | straight * | curved | 2 /1 | √ |
| 1187´04 | †*K. kipkechi* | reduced | straight | straight | 2?/1 | – |
| 1188´04 | †*K. kipkechi* | reduced + | straight * | curved + | – | √ |
| 1189´04 | †*K. kipkechi* | reduced | curved | straight | 2/– | – |
| 1190´04 | †*K. kipkechi* | reduced | curved | curved | 2/– | – |
| 1191´04 | †*K.* cf*. kipkechi* **°** | ? | ? | ? | – | – |
| 1192´04 | †*K. kipkechi* | overlapping | straight | straight + | 2?/1 | √ |
| 1192a/b´05 | †*K. kipkechi* | reduced + | straight | straight + | 2?/– | √ |
| 1193´04 | †*K. kipkechi* | reduced + | straight | straight | 2/1 | – |
| 1194´04 | †*K. kipkechi* | reduced | – | curved + | 1/– | √ |
| 1195(1)´04 | †*K.* cf*. kipkechi* | reduced | straight | curved | – | – |
| 1195(2)´04 | †*K.* cf*. kipkechi* | reduced | straight | curved | – | – |
| 1196´04 | †*K.* cf*. kipkechi* | – | – | – | ?/1 | – |
| 1196R´04 | †*K.* cf*. kipkechi* | – | – | – | 2/1 | – |
| 1197(1)´04 | †*K.* cf*. kipkechi* | – | – | – | 1/2 | – |
| 1197(2)´04 | †*K.* cf*. kipkechi* **°** | ? | ? | ? | – | – |
| 1198a/b´04 | †*K. kipkechi* | reduced | curved | curved | 2/– | √ |
| 1199a/b´04 | †*K. kipkechi* | reduced + | straight | straight | ?/1 | – |
| 1199bR´04 | †*K.* cf*. kipkechi* | – | – | – | – | – |
| 1200´04 | †*K. kipkechi* | reduced + | straight | straight + | 2?/– | √ |

S6 Table. (Continued)

| 1201´04 | †*K.* cf*. kipkechi* | – | – | – | – | √ |
| --- | --- | --- | --- | --- | --- | --- |
| 1202´04 | †*K. kipkechi* | reduced | straight *? | straight | 2/1 | – |
| 1203´04 | †*K.* cf*. kipkechi* | – | – | curved | – | – |
| 1203a/b´05 | †*K. kipkechi* | reduced | curved | straight | 1?/– | – |
| 1204´04 | †*K. kipkechi* | – | – | straight + | – | – |
| 1204´05 | †*K. kipkechi* | reduced | straight | straight + | 2/– | √ |
| 1205´04 | †*K.* cf*. kipkechi* | – | – | – | –/1? | – |
| 1206(1)/1211´04 | †*K. kipkechi* | reduced + | curved | straight + | 2/2 | – |
| 1206(2)´04 | †*K.* cf*. kipkechi* | reduced | straight | straight | 2/2 | – |
| 1207(1)´04 | †*K.* cf*. kipkechi* | – | – | – | 2/– | – |
| 1207(2)´04 | †*K.* cf*. kipkechi* | – | – | – | – | – |
| 1208´04 | †*K.* cf*. kipkechi* | – | – | – | 1/2 | – |
| 1209´04 | †*K. kipkechi* | reduced | straight | straight + | – | – |
| 1209a/b´05 | †*K. kipkechi* | reduced + | straight | curved | 2?/– | √ |
| 1210a/b´04 | †*K.* cf*. kipkechi* | reduced | straight ^#^? | curved | 1/2 | – |
| 1212a/b´04 | †*K.* cf*. kipkechi* | reduced + | straight | straight + | – | – |
| 1213(1)´04 | †*K. kipkechi* | reduced | straight | – | – | – |
| 1213(2)´04 | †*K.* cf*. kipkechi* | reduced | straight * | – | – | – |
| 1214´04 | †*K.* cf*. kipkechi* | reduced + | straight | – | –/1? | – |
| 1215(1)´04 | †*K. kipkechi* | – | – | – | 2?/– | – |
| 1215(2)´04 | †*K.* cf*. kipkechi* | – | – | straight | 1?/– | – |
| 1216(1)´04 | †*K.* cf*. kipkechi* | reduced + | straight | curved | 2?/– | – |
| 1216(2)´04 | †*K.* cf*. kipkechi* **°** | ? | ? | ? | – | – |
| 1217a(1)/b(1)´04 | †*K. kipkechi* | reduced + | Straight ^#^? | Straight + | 2?/2 | √ |
| 1217a(2)/b(2)´04 | †*K.* cf*. kipkechi* | – | – | – | 2/2 | – |
| 1217a(3)/b(3) | †*K.* cf*. kipkechi* | – | – | – | – | – |
| 1218´04 | †*K. kipkechi* | – | – | curved + | 2/2 | – |

S6 Table. (Continued)

| 1218a/b´05 | †*K. kipkechi* | reduced + | straight | curved | 2/1 | √ |
| --- | --- | --- | --- | --- | --- | --- |
| 1219(1)´04 | †*K. kipkechi* | reduced | straight * | curved | – | √ |
| 1219(2)´04 | †*K.* cf*. kipkechi* | reduced + | straight | – | – | – |
| 1219(3)´04 | †*K.* cf*. kipkechi* | reduced | curved | straight | – | – |
| 1219R´04 | †*K.* cf*. kipkechi* | – | – | – | – | – |
| 1220(1)´04 | †*K. kipkechi* | overlapping | straight * | curved | – | – |
| 1220(2)´04 | †*K.* cf*. kipkechi* | – | – | curved | – | – |
| 1220R´04 | †*K. kipkechi* | – | – | – | 2/– | – |
| 1221(1)´04 | †*K. kipkechi* | reduced + | straight | straight + | – | √ |
| 1221(2)´04 | †*K.* cf*. kipkechi* | – | – | – | 2?/– | – |
| 1221R(1)´04 | †*K.* cf*. kipkechi* | – | – | – | 2?/– | – |
| 1221R(2)´04 | †*K.* cf*. kipkechi* **°** | ? | ? | ? | – | – |
| 1221R(3)´04 | †*K.* cf*. kipkechi* | – | – | – | – | – |
| 1221R(4)´04 | †*K.* cf*. kipkechi* | – | – | – | – | – |
| 1222(1)´04 | †*K.* cf*. kipkechi* | reduced | straight | curved + | 2/2? | – |
| 1222(2)´04 | †*K. cf. kipkechi* | – | – | – | 1?/– | – |
| 1223´04 | †*K. cf. kipkechi* | – | – | – | – | – |
| 1223R´04 | †*K.* cf*. kipkechi* | reduced + | straight | straight + | 1?/1 | √ |
| 1224´04 | †*K.* cf*. kipkechi* | reduced | curved * | curved | – | – |
| 1224R´04 | †*K.* cf*. kipkechi* | – | – | – | – | – |
| 1225/1232´04 | †*K.* cf*. kipkechi* | – | – | – | 2?– | √ |
| 1226a(1)/b(1)´04 | †*K.* cf*. kipkechi* | reduced + | straight | straight + | – | – |
| 1226a(2)/b(2)´04 | †*K.* cf*. kipkechi* | reduced + | straight | curved | 2/3 | – |
| 1227(1)´04 | †*K. kipkechi* | reduced + | straight | straight | 2/1? | – |
| 1227(2)´04 | †*K.* cf*. kipkechi* | – | – | – | – | – |
| 1227(3)´04 | †*K.* cf*. kipkechi* | – | – | – | – | – |
| 1228(1)/1237R(1)´04 | †*K. kipkechi* | reduced + | straight | straight + | 2/– | √ |

S6 Table. (Continued)

| 1228(2)´04 | †*K.* cf*. kipkechi* | – | – | – | – | – |
| --- | --- | --- | --- | --- | --- | --- |
| 1228(3)´04 | †*K.* cf*. kipkechi* | – | – | – | – | – |
| 1228R´04 | †*K.* cf*. kipkechi* | – | – | – | 1/1 | – |
| 1229´04 | †*K.* cf*. kipkechi* | – | – | – | 2/2 | – |
| 1229R(1)´04 | †*K.* cf*. kipkechi* | – | – | – | – | – |
| 1229R(2)´04 | †*K.* cf*. kipkechi* | – | – | – | – | – |
| 1229R(3)´04 | †*K.* cf*. kipkechi* | – | – | – | – | – |
| 1230a/b´04 | †*K.* cf*. kipkechi* | – | – | – | – | – |
| 1231´04 | †*K.* cf*. kipkechi* | – | – | – | 1/2 | – |
| 1233/1234(1)´04 | †*K. kipkechi* | – | – | – | – | – |
| 1234(2)´04 | †*K. kipkechi* | reduced | straight | straight + | – | – |
| 1234R´04 | †*K. kipkechi* | – | – | – | 1?/– | √ |
| 1235(1)´04 | †*K.* cf*. kipkechi* | – | – | – | – | – |
| 1235(2)´04 | †*K.* cf*. kipkechi* | – | – | – | 2?/– | – |
| 1236(1)´04 | †*K. kipkechi* | – | – | straight + | 2?/– | – |
| 1236(2)´04 | †*K.* cf*. kipkechi* | – | – | – | 1/1? | – |
| 1236(3)´04 | †*K.* cf*. kipkechi* | – | – | – | – | – |
| 1236(4)´04 | †*K.* cf*. kipkechi* | – | – | – | – | – |
| 1236R(1)´04 | †*K.* cf*. kipkechi* | – | – | – | 1?/2 | √ |
| 1236R(2)´04 | †*K.* cf*. kipkechi* | – | – | – | – | – |
| 1236R(3)´04 | †*K.* cf*. kipkechi* | – | – | curved | 2/– | – |
| 1236R(4)´04 | †*K.* cf*. kipkechi* | reduced | straight | curved | –/2 | – |
| 1236R(5)´04 | †*K.* cf*. kipkechi* | – | – | – | – | – |
| 1236R(6)´04 | †*K.* cf*. kipkechi* | – | – | – | – | – |
| 1237(1)´04 | †*K. kipkechi* | – | – | – | 1/2 | – |
| 1237(2)´04 | †*K. kipkechi* | – | – | straight + | 2/– | – |
| 1237(3)´04 | †*K.* cf*. kipkechi* **°** | ? | ? | ? | – | – |

S6 Table. (Continued)

| 1237(4)´04 | †*K.* cf*. kipkechi* | – | – | – | 2?/– | – |
| --- | --- | --- | --- | --- | --- | --- |
| 1237(5)´04 | †*K.* cf*. kipkechi* | reduced | curved | – | – | – |
| 1237(6)´04 | †*K.* cf*. kipkechi* | – | – | – | – | – |
| 1237(7)´04 | †*K.* cf*. kipkechi* | – | – | – | 2?/2 | – |
| 1237(8)´04 | †*K.* cf*. kipkechi* | – | – | – | – | – |
| 1237R(2)´04 | †*K.* cf*. kipkechi* | reduced | curved | straight | 1?/– | – |
| 1237R(3)´04 | †*K.* cf*. kipkechi* | reduced | straight | – | – | – |
| 1324´04 | †*K. kipkechi* | reduced | straight | curved + | 2/1 | – |
| 1325´04 | †*K. kipkechi* | reduced + | straight | straight + | 1/2 | √ |

Abbreviations: √, first neural spine discernible; +, proximal part of the bone with projection; *, bone partially fused to hypural plate; ^#^, bone completely fused to hypural plate; **°**, uncertain dorsoventral orientation of specimen; 1/1, one short ray supported by two proximal radials; 1/2, one short ray and second ray supported by two proximal radials; 2/1, two short rays and first ray supported by two proximal radials; 2/2, two short rays and second ray supported by two proximal radials; 2/3, two short rays and third ray supported by two proximal radials.
