# Supplementary material for: †Kenyaichthyidae fam. nov. and †Kenyaichthys gen. nov. – First Record of a Fossil Aplocheiloid Killifish (Teleostei, Cyprinodontiformes)
Source: PLoS One. 2015 Apr 29;10(4):e0123056. doi: 10.1371/journal.pone.0123056 (PMC4414574; doi:10.1371/journal.pone.0123056)
Supplement: S7 Table — (DOC) [file pone.0123056.s007.doc]

**S7 Table**. Hypural plate length (lH) and width (wH), and numbers of preural vertebrae obtained from the extant cyprinodontoid and aplocheiloid specimens used for comparison.

| ID | Species | Locality | Sex | SL (mm) | lH (mm) | wH (mm) |
| --- | --- | --- | --- | --- | --- | --- |
| ZM-CBSU C227 | *Aphanius sophiae* | Iran, Kor Basin | Male | 29.50 | 1.86 | 2.48 |
| ZM-CBSU C295 |  |  | Male | 32.30 | 1.78 | 2.85 |
| ZM-CBSU C316 |  |  | Female | 28.60 | 1.37 | 2.00 |
| ZM-CBSU 10962 |  |  | Male | 18.60 | 1.09 | 1.63 |
| ZM-CBSU 8296 |  |  | Male | 28.10 | 1.66 | 2.30 |
| ZM-CBSU 8401 |  |  | Female | 30.60 | 1.66 | 2.45 |
| ZM-CBSU 281 |  |  | Male | 28.30 | 1.71 | 2.45 |
| ZM-CBSU 283 |  |  | Female | 34.60 | 2.06 | 2.84 |
| ZM-CBSU 6193 |  |  | Male | 25.30 | 1.41 | 2.02 |
| ZM-CBSU 6171 |  |  | Female | 31.40 | 1.76 | 2.39 |
| ZM-CBSU 10883 |  |  | Male | 26.20 | 1.57 | 2.02 |
| ZM-CBSU 10884 |  |  | Female | 27.50 | 1.38 | 2.20 |
| ZM-CBSU 284 |  |  | Female | 19.60 | 1.06 | 1.53 |
| ZM-CBSUZG 188 |  |  | Female | 42.80 | 2.13 | 3.76 |
| ZM-CBSUZG 177 |  |  | Female | 38.40 | 2.49 | 3.74 |
| ZM-CBSUZG 178 |  |  | Female | 25.80 | 1.09 | 1.97 |
| ZM-CBSUZG 185 |  |  | Male | 30.20 | 1.56 | 2.48 |
| ZM-CBSUZG 184 |  |  | Female | 31.50 | 1.61 | 2.35 |
| ZM-CBSUZG 183 |  |  | Male | 34.90 | 2.15 | 3.24 |
| ZM-CBSUZG 13 | *Aphanius farsicus* | Iran, Marharlu Basin | Male | 20.60 | 1.03 | 1.57 |
| ZM-CBSUZG 140 |  |  | Male | 22.10 | 1.28 | 1.72 |
| ZM-CBSUZG 141 |  |  | Female | 27.90 | 1.47 | 2.16 |
| ZM-CBSUZG 142 |  |  | Female | 32.00 | 1.81 | 2.91 |
| ZM-CBSUZG 1 |  |  | Male | 22.80 | 1.34 | 2.04 |
| ZM-CBSUZG 8 |  |  | Female | 23.20 | 1.25 | 1.72 |
| ZM-CBSUZG 359 | *Aphanius arakensis* | Iran, Namek Basin | Female | 34.80 | 1.77 | 2.92 |
| ZM-CBSUZG 361 |  |  | Female | 23.30 | 1.38 | 2.03 |
| ZM-CBSUZG 350 |  |  | Male | 30.90 | 1.50 | 2.14 |
| ZM-CBSUZG 352 |  |  | Female | 24.60 | 1.48 | 1.81 |

S7 Table. (Continued)

| ZM-CBSUZG 354 |  | |  | Male | 28.40 | 1.55 | 2.05 |
| --- | --- | --- | --- | --- | --- | --- | --- |
| ZM-CBSUZG 356 |  | |  | Male | 29.40 | 1.65 | 2.15 |
| ZM-CBSUZG 363 | *Aphanius mesopotamicus* | | Iran, Karun Basin | Male | 21.40 | 1.05 | 1.64 |
| ZM-CBSUZG 362 |  | |  | Male | 21.90 | 1.08 | 1.47 |
| ZM-CBSUZG 365 |  | |  | Female | 25.30 | 1.16 | 1.77 |
| ZM-CBSUZG 364 |  | |  | Female | 22.40 | 0.98 | 1.59 |
| P. 188937-188938 (A) | *Pachypanchax playfairii* | | Seychelles | Male | 42.20 | 2.23 | 2.69 |
| P. 188937-188938 (B) |  | |  | Female | 31.40 | 1.50 | 1.70 |
| A4-039-P-0133-0134 (A) | *Nothobranchius orthonotus* | | Mosambique | ? | 42.30 | 2.64 | 3.64 |
| A4-039-P-0133-0134 (B) |  | |  | ? | 33.60 | 2.00 | 2.87 |
| 91-100-P-0050-0051 (A) | *Fundulopanchax sjoestedti* | | Nigeria | ? | 38.30 | 1.93 | 2.25 |
| 91-100-P-0050-0051 (B) |  | |  | ? | 33.80 | 1.87 | 2.15 |
| 92-052-P-0512-0513 (A) | *Epiplatys sexfasciatus* | |  | ? | 39.20 | 2.47 | 2.69 |
| 92-052-P-0512-0513 (B) |  | |  | ? | 36.10 | 2.21 | 2.46 |
| 91-080-P-0063-0064 (A) | *Aphyosemion castaneum* | | Zaire | ? | 27.00 | 1.52 | 1.72 |
| 91-080-P-0063-0064 (B) |  | |  | ? | 28.10 | 1.56 | 1.59 |
| 91-001-P-0378-0379 (A) | *Foerschichthys flavipinnis* | | Nigeria | ? | 13.70 | 0.60 | 0.70 |
| 91-001-P-0378-0379 (B) |  | |  | ? | 13.90 | 0.68 | 0.68 |
| ID | Species | | Locality | lH (%SL) | wH (%SL) | PU no. |  |
| ZM-CBSU C227 | *Aphanius sophiae* | | Iran, Kor Basin | 6.31 | 8.41 | 4 |  |
| ZM-CBSU C295 |  | |  | 5.51 | 8.82 | n.a. |  |
| ZM-CBSU C316 |  | |  | 4.79 | 6.99 | 4 |  |
| ZM-CBSU 10962 |  | |  | 5.86 | 8.76 | 4 |  |
| ZM-CBSU 8296 |  | |  | 5.91 | 8.19 | 3 |  |
| ZM-CBSU 8401 |  | |  | 5.49 | 8.10 | n.a. |  |
| ZM-CBSU 281 |  | |  | 6.04 | 8.66 | 4 |  |
| ZM-CBSU 283 |  | |  | 5.95 | 8.21 | n.a. |  |
| ZM-CBSU 6193 |  | |  | 5.57 | 7.98 | 3 |  |
| ZM-CBSU 6171 |  |  | | 5.61 | 7.61 | n.a. |  |
| ZM-CBSU 10883 |  |  | | 5.99 | 7.71 | 3 |  |
| ZM-CBSU 10884 |  | |  | 5.02 | 8.00 | n.a. |  |

S7 Table. (Continued)

| ZM-CBSU 284 |  |  | 5.41 | 7.81 | n.a. |  |
| --- | --- | --- | --- | --- | --- | --- |
| ZM-CBSUZG 188 |  |  | 4.98 | 8.79 | 4 |  |
| ZM-CBSUZG 177 |  |  | 6.49 | 9.73 | 4? |  |
| ZM-CBSUZG 178 |  |  | 4.22 | 7.64 | 4 |  |
| ZM-CBSUZG 185 |  |  | 5.15 | 8.23 | 3 |  |
| ZM-CBSUZG 184 |  |  | 5.10 | 7.45 | 4 |  |
| ZM-CBSUZG 183 |  |  | 6.16 | 9.27 | 4 |  |
| ZM-CBSUZG 13 | *Aphanius farsicus* | Iran, Marharlu Basin | 4.99 | 7.62 | 3 |  |
| ZM-CBSUZG 140 |  |  | 5.77 | 7.79 | 4 |  |
| ZM-CBSUZG 141 |  |  | 5.26 | 7.73 | 4 |  |
| ZM-CBSUZG 142 |  |  | 5.66 | 9.08 | 3 |  |
| ZM-CBSUZG 1 |  |  | 5.87 | 8.95 | 3 |  |
| ZM-CBSUZG 8 |  |  | 5.38 | 7.41 | 3 |  |
| ZM-CBSUZG 359 | *Aphanius arakensis* | Iran, Namek Basin | 5.10 | 8.38 | 3 |  |
| ZM-CBSUZG 361 |  |  | 5.93 | 8.71 | 4 |  |
| ZM-CBSUZG 350 |  |  | 4.85 | 6.92 | 4 |  |
| ZM-CBSUZG 352 |  |  | 6.02 | 7.36 | 3 |  |
| ZM-CBSUZG 354 |  |  | 5.45 | 7.21 | 3 |  |
| ZM-CBSUZG 356 |  |  | 5.60 | 7.31 | 4 |  |
| ZM-CBSUZG 363 | *Aphanius mesopotamicus* | Iran, Karun Basin | 4.90 | 7.65 | 4 |  |
| ZM-CBSUZG 362 |  |  | 4.95 | 6.72 | 3 |  |
| ZM-CBSUZG 365 |  |  | 4.58 | 6.98 | 4 |  |
| ZM-CBSUZG 364 |  |  | 4.36 | 7.11 | 3 |  |
| P. 188937-188938 (A) | *Pachypanchax playfairii* | Seychelles | 5.28 | 6.37 | 5 |  |
| P. 188937-188938 (B) |  |  | 4.78 | 5.41 | 5 |  |
| A4-039-P-0133-0134 (A) | *Nothobranchius orthonotus* | Mosambique | 6.24 | 8.61 | 4 |  |
| A4-039-P-0133-0134 (B) |  |  | 5.95 | 8.54 | 5 |  |
| 91-100-P-0050-0051 (A) | *Fundulopanchax sjoestedti* | Nigeria | 5.04 | 5.87 | 4 |  |
| 91-100-P-0050-0051 (B) |  |  | 5.53 | 6.36 | 4 |  |
| 92-052-P-0512-0513 (A) | *Epiplatys sexfasciatus* |  | 6.30 | 6.86 | 4 |  |
| 92-052-P-0512-0513 (B) |  |  | 6.12 | 6.81 | 4 |  |

S7 Table. (Continued)

| 91-080-P-0063-0064 (A) | *Aphyosemion castaneum* | Zaire | 5.63 | 6.37 | 4 |  |
| --- | --- | --- | --- | --- | --- | --- |
| 91-080-P-0063-0064 (B) |  |  | 5.56 | 5.67 | 4 |  |
| 91-001-P-0378-0379 (A) | *Foerschichthys flavipinnis* | Nigeria | 4.38 | 5.11 | 4 |  |
| 91-001-P-0378-0379 (B) |  |  | 4.89 | 4.89 | 4 |  |

Determination of sex was only possible for *Aphanius sophiae*, *Aphanius farsicus*, *Aphanius arakensis*, *Aphanius mesopotamicus* and *Pachypanchax playfairii*. Abbreviations: PU no., number of preural vertebrae.
