# Supplementary material for: †Kenyaichthyidae fam. nov. and †Kenyaichthys gen. nov. – First Record of a Fossil Aplocheiloid Killifish (Teleostei, Cyprinodontiformes)
Source: PLoS One. 2015 Apr 29;10(4):e0123056. doi: 10.1371/journal.pone.0123056 (PMC4414574; doi:10.1371/journal.pone.0123056)
Supplement: S8 Table — (DOC) [file pone.0123056.s008.doc]

**S8 Table. Spine-**ratios of the recent cyprinodontoid and aplocheiloid specimens used in this study.

| ID | Species | Suborder | Family | N2 | H2 | NS2/NS4 | NS2/NS5 | HS2/HS4 |
| --- | --- | --- | --- | --- | --- | --- | --- | --- |
| ZM-CBSU 6193 | *Aphanius sophiae* | A | 1 | 0.19 | 0.17 | 3.80 | 3.80 | 4.25 |
| ZM-CBSU 10883 |  | A | 1 | 0.20 | 0.20 | 5.00 | 4.00 | 3.33 |
| ZM-CBSU 281 |  | A | 1 | 0.23 | 0.21 | 3.29 | 5.75 | 2.63 |
| ZM-CBSU 8296 |  | A | 1 | 0.20 | 0.23 | 4.00 | 4.00 | 3.83 |
| ZM-CBSU 10962 |  | A | 1 | 0.14 | 0.13 | 3.50 | 4.67 | 2.60 |
| ZM-CBSU C227 |  | A | 1 | 0.32 | 0.31 | 2.29 | 8.00 | 3.44 |
| ZM-CBSU C316 |  | A | 1 | 0.16 | 0.18 | 2.29 | 5.33 | 2.57 |
| ZM-CBSU 8401 |  | A | 1 | 0.24 | 0.22 | 3.43 | 4.00 | 3.14 |
| ZM-CBSU 6171 |  | A | 1 | 0.21 | 0.22 | 5.25 | 3.50 | 4.40 |
| ZM-CBSU 10884 |  | A | 1 | 0.18 | 0.20 | 2.25 | 3.00 | 2.22 |
| ZM-CBSU 283 |  | A | 1 | 0.27 | 0.26 | 5.40 | 5.40 | 4.33 |
| ZM-CBSU C295 |  | A | 1 | 0.24 | 0.21 | 4.80 | 4.80 | 3.00 |
| ZM-CBSU 284 |  | A | 1 | 0.13 | 0.13 | 1.86 | 3.25 | 2.60 |
| ZM-CBSUZG 188 |  | A | 1 | 0.19 | 0.20 | 1.58 | 3.80 | 1.82 |
| ZM-CBSUZG 177 |  | A | 1 | 0.39 | 0.39 | 2.79 | 3.00 | 2.17 |
| ZM-CBSUZG 178 |  | A | 1 | 0.21 | 0.27 | 2.10 | 2.63 | 3.38 |
| ZM-CBSUZG 185 |  | A | 1 | 0.25 | 0.28 | 4.17 | 5.00 | 3.50 |
| ZM-CBSUZG 184 |  | A | 1 | 0.31 | 0.36 | 1.72 | 3.10 | 2.00 |
| ZM-CBSUZG 183 |  | A | 1 | 0.34 | 0.35 | 1.89 | 4.86 | 1.46 |
| ZM-CBSUZG 13 | *Aphanius farsicus* | A | 1 | 0.13 | 0.14 | 2.60 | 4.33 | 2.00 |
| ZM-CBSUZG 140 |  | A | 1 | 0.16 | 0.16 | 2.29 | 4.00 | 2.00 |
| ZM-CBSUZG 141 |  | A | 1 | 0.16 | 0.17 | 2.67 | 5.33 | 2.43 |
| ZM-CBSUZG 142 |  | A | 1 | 0.14 | 0.16 | 7.00 | 3.50 | 5.33 |
| ZM-CBSUZG 1 |  | A | 1 | 0.30 | 0.31 | 6.00 | 7.50 | 6.20 |
| ZM-CBSUZG 8 |  | A | 1 | 0.16 | 0.19 | 4.00 | 5.33 | 1.90 |
| ZM-CBSUZG 359 | *Aphanius arakensis* | A | 1 | 0.24 | 0.21 | 12.00 | 4.80 | 10.50 |
| ZM-CBSUZG 361 |  | A | 1 | 0.22 | 0.28 | 3.14 | 2.75 | 3.50 |
| ZM-CBSUZG 350 |  | A | 1 | 0.15 | 0.17 | 2.14 | – | 2.13 |
| ZM-CBSUZG 352 |  | A | 1 | 0.21 | 0.25 | 2.33 | 5.25 | 2.78 |
| ZM-CBSUZG 354 |  | A | 1 | 0.23 | 0.30 | 4.60 | 7.67 | 4.29 |

S8 Table. (Continued)

| ZM-CBSUZG 356 |  | A | 1 | 0.33 | 0.31 | 2.06 | 6.60 | 1.72 |
| --- | --- | --- | --- | --- | --- | --- | --- | --- |
| ZM-CBSUZG 363 | *Aphanius mesopotamicus* | A | 1 | 0.15 | 0.16 | 1.00 | 2.14 | 1.00 |
| ZM-CBSUZG 362 |  | A | 1 | 0.17 | 0.16 | 3.40 | 5.67 | 2.67 |
| ZM-CBSUZG 365 |  | A | 1 | 0.14 | 0.18 | 2.33 | 2.80 | 2.57 |
| ZM-CBSUZG 364 |  | A | 1 | 0.21 | 0.18 | 3.50 | 3.50 | 3.00 |
| P. 188937-188938 (A) | *Pachypanchax playfairii* | B | 2 | 0.44 | 0.36 | 4.40 | 6.29 | 2.25 |
| P. 188937-188938 (B) |  | B | 2 | 0.19 | 0.22 | 1.90 | 3.80 | 1.57 |
| A4-039-P-0133-0134 (A) | *Nothobranchius orthonotus* | B | 3 | 0.27 | 0.32 | 3.00 | 5.40 | 3.56 |
| A4-039-P-0133-0134 (B) |  | B | 3 | 0.23 | 0.22 | 2.56 | 2.56 | 2.00 |
| 91-100-P-0050-0051 (A) | *Fundulopanchax sjoestedti* | B | 3 | 0.20 | 0.20 | 2.86 | 3.33 | 2.86 |
| 91-100-P-0050-0051 (B) |  | B | 3 | 0.17 | 0.11 | 2.13 | 3.40 | 1.38 |
| 92-052-P-0512-0513 (A) | *Epiplatys sexfasciatus* | B | 3 | 0.14 | 0.20 | 2.80 | 2.33 | 4.00 |
| 92-052-P-0512-0513 (B) |  | B | 3 | 0.15 | 0.16 | 3.00 | 3.00 | 3.20 |
| 91-080-P-0063-0064 (A) | *Aphyosemion castaneum* | B | 3 | 0.11 | 0.08 | 3.67 | 2.75 | 2.67 |
| 91-080-P-0063-0064 (B) |  | B | 3 | 0.07 | 0.09 | 1.75 | 1.40 | 2.25 |
| 91-001-P-0378-0379 (A) | *Foerschichthys flavipinnis* | B | 3 | 0.04 | 0.03 | 2.00 | 2.00 | 1.50 |
| 91-001-P-0378-0379 (B) |  | B | 3 | 0.04 | 0.03 | 2.00 | – | 1.50 |
| ID | Species | Suborder | Family | NS3 | HS3 | HS2/HS5 | NS3/NS5 | HS3/HS5 |
| ZM-CBSU 6193 | *Aphanius sophiae* | A | 1 | 0.16 | 0.13 | 2.83 | 3.20 | 2.17 |
| ZM-CBSU 10883 |  | A | 1 | 0.15 | 0.17 | 3.33 | 3.00 | 2.83 |
| ZM-CBSU 281 |  | A | 1 | 0.22 | 0.20 | 4.20 | 5.50 | 4.00 |
| ZM-CBSU 8296 | *Aphanius sophiae* | A | 1 | 0.27 | 0.20 | 3.29 | 5.40 | 2.86 |
| ZM-CBSU 10962 |  | A | 1 | 0.08 | 0.11 | 3.25 | 2.67 | 2.75 |
| ZM-CBSU C227 |  | A | 1 | 0.26 | 0.33 | 6.20 | 6.50 | 6.60 |
| ZM-CBSU C316 |  | A | 1 | 0.17 | 0.17 | 4.50 | 5.67 | 4.25 |
| ZM-CBSU 8401 |  | A | 1 | 0.24 | 0.23 | 3.67 | 4.00 | 3.83 |
| ZM-CBSU 6171 |  | A | 1 | 0.14 | 0.16 | 3.67 | 2.33 | 2.67 |
| ZM-CBSU 10884 |  | A | 1 | 0.20 | 0.20 | 5.00 | 3.33 | 5.00 |
| ZM-CBSU 283 |  | A | 1 | 0.29 | 0.23 | 5.20 | 5.80 | 4.60 |
| ZM-CBSU C295 |  | A | 1 | 0.18 | 0.09 | 5.25 | 3.60 | 2.25 |
| ZM-CBSU 284 |  | A | 1 | 0.11 | 0.10 | 3.25 | 2.75 | 2.50 |

S8 Table. (Continued)

| ZM-CBSUZG 188 |  | A | 1 | 0.15 | 0.18 | 4.00 | 3.00 | 3.60 |
| --- | --- | --- | --- | --- | --- | --- | --- | --- |
| ZM-CBSUZG 177 |  | A | 1 | 0.41 | 0.43 | 3.55 | 3.15 | 3.91 |
| ZM-CBSUZG 178 |  | A | 1 | 0.19 | 0.27 | 3.86 | 2.38 | 3.86 |
| ZM-CBSUZG 185 |  | A | 1 | 0.25 | 0.27 | 5.60 | 5.00 | 5.40 |
| ZM-CBSUZG 184 |  | A | 1 | 0.36 | 0.33 | 7.20 | 3.60 | 6.60 |
| ZM-CBSUZG 183 |  | A | 1 | 0.31 | 0.33 | 3.89 | 4.43 | 3.67 |
| ZM-CBSUZG 13 | *Aphanius farsicus* | A | 1 | 0.14 | 0.13 | 3.50 | 4.67 | 3.25 |
| ZM-CBSUZG 140 |  | A | 1 | 0.17 | 0.18 | 4.00 | 4.25 | 4.50 |
| ZM-CBSUZG 141 |  | A | 1 | 0.16 | 0.17 | 5.67 | 5.33 | 5.67 |
| ZM-CBSUZG 142 |  | A | 1 | 0.06 | 0.08 | 5.33 | 1.50 | 2.67 |
| ZM-CBSUZG 1 |  | A | 1 | 0.23 | 0.29 | 7.75 | 5.75 | 7.25 |
| ZM-CBSUZG 8 |  | A | 1 | 0.13 | 0.17 | 6.33 | 4.33 | 5.67 |
| ZM-CBSUZG 359 | *Aphanius arakensis* | A | 1 | 0.22 | 0.13 | 5.25 | 4.40 | 3.25 |
| ZM-CBSUZG 361 |  | A | 1 | 0.24 | 0.29 | 4.00 | 3.00 | 4.14 |
| ZM-CBSUZG 350 |  | A | 1 | 0.19 | 0.26 | 4.25 | – | 6.50 |
| ZM-CBSUZG 352 |  | A | 1 | 0.20 | 0.25 | 6.25 | 5.00 | 6.25 |
| ZM-CBSUZG 354 |  | A | 1 | 0.20 | 0.17 | 10.00 | 6.67 | 5.67 |
| ZM-CBSUZG 356 |  | A | 1 | 0.39 | 0.36 | 6.20 | 7.80 | 7.20 |
| ZM-CBSUZG 363 | *Aphanius mesopotamicus* | A | 1 | 0.17 | 0.14 | 2.67 | 2.43 | 2.33 |
| ZM-CBSUZG 362 |  | A | 1 | 0.12 | 0.17 | 5.33 | 4.00 | 5.67 |
| ZM-CBSUZG 365 |  | A | 1 | 0.16 | 0.17 | 3.00 | 3.20 | 2.83 |
| ZM-CBSUZG 364 |  | A | 1 | 0.17 | 0.19 | 3.60 | 2.83 | 3.80 |
| P. 188937-188938 (A) | *Pachypanchax playfairii* | B | 2 | 0.22 | 0.34 | 3.60 | 3.14 | 3.40 |
| P. 188937-188938 (B) |  | B | 2 | 0.16 | 0.18 | 2.75 | 3.20 | 2.25 |
| A4-039-P-0133-0134 (A) | *Nothobranchius orthonotus* | B | 3 | 0.18 | 0.27 | 5.33 | 3.60 | 4.50 |
| A4-039-P-0133-0134 (B) |  | B | 3 | 0.14 | 0.16 | 2.00 | 1.56 | 1.45 |
| 91-100-P-0050-0051 (A) | *Fundulopanchax sjoestedti* | B | 3 | 0.12 | 0.12 | 5.00 | 2.00 | 3.00 |
| 91-100-P-0050-0051 (B) |  | B | 3 | 0.10 | 0.09 | 2.20 | 2.00 | 1.80 |
| 92-052-P-0512-0513 (A) | *Epiplatys sexfasciatus* | B | 3 | 0.08 | 0.14 | 2.86 | 1.33 | 2.00 |
| 92-052-P-0512-0513 (B) |  | B | 3 | 0.06 | 0.13 | 3.20 | 1.20 | 2.60 |
| 91-080-P-0063-0064 (A) | *Aphyosemion castaneum* | B | 3 | 0.05 | 0.06 | 1.33 | 1.25 | 1.00 |

S8 Table. (Continued)

| 91-080-P-0063-0064 (B) |  | B | 3 | 0.04 | 0.06 | 1.80 | 0.80 | 1.20 |
| --- | --- | --- | --- | --- | --- | --- | --- | --- |
| 91-001-P-0378-0379 (A) | *Foerschichthys flavipinnis* | B | 3 | 0.02 | 0.04 | 1.00 | 1.00 | 1.33 |
| 91-001-P-0378-0379 (B) |  | B | 3 | 0.03 | 0.03 | 1.50 | – | 1.50 |
| ID | *Species* | Suborder | Family | NS4 | HS4 | NS5 | HS5 |  |
| ZM-CBSU 6193 | *Aphanius sophiae* | A | 1 | 0.05 | 0.04 | 0.05 | 0.06 |  |
| ZM-CBSU 10883 |  | A | 1 | 0.04 | 0.06 | 0.05 | 0.06 |  |
| ZM-CBSU 281 |  | A | 1 | 0.07 | 0.08 | 0.04 | 0.05 |  |
| ZM-CBSU 8296 |  | A | 1 | 0.05 | 0.06 | 0.05 | 0.07 |  |
| ZM-CBSU 10962 |  | A | 1 | 0.04 | 0.05 | 0.03 | 0.04 |  |
| ZM-CBSU C227 |  | A | 1 | 0.14 | 0.09 | 0.04 | 0.05 |  |
| ZM-CBSU C316 |  | A | 1 | 0.07 | 0.07 | 0.03 | 0.04 |  |
| ZM-CBSU 8401 |  | A | 1 | 0.07 | 0.07 | 0.06 | 0.06 |  |
| ZM-CBSU 6171 | *Aphanius sophiae* | A | 1 | 0.04 | 0.05 | 0.06 | 0.06 |  |
| ZM-CBSU 10884 |  | A | 1 | 0.08 | 0.09 | 0.06 | 0.04 |  |
| ZM-CBSU 283 |  | A | 1 | 0.05 | 0.06 | 0.05 | 0.05 |  |
| ZM-CBSU C295 |  | A | 1 | 0.05 | 0.07 | 0.05 | 0.04 |  |
| ZM-CBSU 284 |  | A | 1 | 0.07 | 0.05 | 0.04 | 0.04 |  |
| ZM-CBSUZG 188 |  | A | 1 | 0.12 | 0.11 | 0.05 | 0.05 |  |
| ZM-CBSUZG 177 |  | A | 1 | 0.14 | 0.18 | 0.13 | 0.11 |  |
| ZM-CBSUZG 178 |  | A | 1 | 0.10 | 0.08 | 0.08 | 0.07 |  |
| ZM-CBSUZG 185 |  | A | 1 | 0.06 | 0.08 | 0.05 | 0.05 |  |
| ZM-CBSUZG 184 |  | A | 1 | 0.18 | 0.18 | 0.10 | 0.05 |  |
| ZM-CBSUZG 183 |  | A | 1 | 0.18 | 0.24 | 0.07 | 0.09 |  |
| ZM-CBSUZG 13 | *Aphanius farsicus* | A | 1 | 0.05 | 0.07 | 0.03 | 0.04 |  |
| ZM-CBSUZG 140 |  | A | 1 | 0.07 | 0.08 | 0.04 | 0.04 |  |
| ZM-CBSUZG 141 |  | A | 1 | 0.06 | 0.07 | 0.03 | 0.03 |  |
| ZM-CBSUZG 142 |  | A | 1 | 0.02 | 0.03 | 0.04 | 0.03 |  |
| ZM-CBSUZG 1 |  | A | 1 | 0.05 | 0.05 | 0.04 | 0.04 |  |
| ZM-CBSUZG 8 |  | A | 1 | 0.04 | 0.10 | 0.03 | 0.03 |  |
| ZM-CBSUZG 359 | *Aphanius arakensis* | A | 1 | 0.02 | 0.02 | 0.05 | 0.04 |  |
| ZM-CBSUZG 361 |  | A | 1 | 0.07 | 0.08 | 0.08 | 0.07 |  |

S8 Table. (Continued)

| ZM-CBSUZG 350 |  | A | 1 | 0.07 | 0.08 | – | 0.04 |  |
| --- | --- | --- | --- | --- | --- | --- | --- | --- |
| ZM-CBSUZG 352 |  | A | 1 | 0.09 | 0.09 | 0.04 | 0.04 |  |
| ZM-CBSUZG 354 |  | A | 1 | 0.05 | 0.07 | 0.03 | 0.03 |  |
| ZM-CBSUZG 356 |  | A | 1 | 0.16 | 0.18 | 0.05 | 0.05 |  |
| ZM-CBSUZG 363 | *Aphanius mesopotamicus* | A | 1 | 0.15 | 0.16 | 0.07 | 0.06 |  |
| ZM-CBSUZG 362 |  | A | 1 | 0.05 | 0.06 | 0.03 | 0.03 |  |
| ZM-CBSUZG 365 |  | A | 1 | 0.06 | 0.07 | 0.05 | 0.06 |  |
| ZM-CBSUZG 364 |  | A | 1 | 0.06 | 0.06 | 0.06 | 0.05 |  |
| P. 188937-188938 (A) | *Pachypanchax playfairii* | B | 2 | 0.10 | 0.16 | 0.07 | 0.10 |  |
| P. 188937-188938 (B) |  | B | 2 | 0.10 | 0.14 | 0.05 | 0.08 |  |
| A4-039-P-0133-0134 (A) | *Nothobranchius orthonotus* | B | 3 | 0.09 | 0.09 | 0.05 | 0.06 |  |
| A4-039-P-0133-0134 (B) |  | B | 3 | 0.09 | 0.11 | 0.09 | 0.11 |  |
| 91-100-P-0050-0051 (A) | *Fundulopanchax sjoestedti* | B | 3 | 0.07 | 0.07 | 0.06 | 0.04 |  |
| 91-100-P-0050-0051 (B) |  | B | 3 | 0.08 | 0.08 | 0.05 | 0.05 |  |
| 92-052-P-0512-0513 (A) | *Epiplatys sexfasciatus* | B | 3 | 0.05 | 0.05 | 0.06 | 0.07 |  |
| 92-052-P-0512-0513 (B) |  | B | 3 | 0.05 | 0.05 | 0.05 | 0.05 |  |
| 91-080-P-0063-0064 (A) | *Aphyosemion castaneum* | B | 3 | 0.03 | 0.03 | 0.04 | 0.06 |  |
| 91-080-P-0063-0064 (B) |  | B | 3 | 0.04 | 0.04 | 0.05 | 0.05 |  |
| 91-001-P-0378-0379 (A) | *Foerschichthys flavipinnis* | B | 3 | 0.02 | 0.02 | 0.02 | 0.03 |  |
| 91-001-P-0378-0379 (B) |  | B | 3 | 0.02 | 0.02 | – | 0.02 |  |

Ratios of spines are given as relative values. Abbreviations: A, Cyprinodontoidei; B, Aplocheiloidei; 1, Cyprinodontidae; 2, Aplocheilidae; 3, Nothobranchiidae.
