# Supplementary material for: †Kenyaichthyidae fam. nov. and †Kenyaichthys gen. nov. – First Record of a Fossil Aplocheiloid Killifish (Teleostei, Cyprinodontiformes)
Source: PLoS One. 2015 Apr 29;10(4):e0123056. doi: 10.1371/journal.pone.0123056 (PMC4414574; doi:10.1371/journal.pone.0123056)
Supplement: S9 Table — Characters are compiled from the following literature: [1], [48], [49], [50], and [55]. (DOC) [file pone.0123056.s009.doc]

**S9 Table. Description of characters used for phylogenetic analysis, and distribution of character states.** Characters are compiled from the following literature: [1], [48], [49], [50], and [55].

(1) Corresponds to character 1 sensu [48]: Size of dorsal process of maxilla: (0) long, medially directed, approximately parallel to ventral process; (1) short anteriorly directed, not parallel to ventral process; (2) vestigial.

(2) Corresponds to character 2 sensu [48]: Shape of dorsal process of maxilla: (0) narrow; (1) broad.

(3) Corresponds to character 3 sensu [48]: Distinct groove on the dorsal process of maxilla: (0) absent; (1) present.

(4) Corresponds to character 6 sensu [48]: Shape of the distal arm of the maxilla: (0) narrow to slightly widened; (1) distinctively widened.

(5) Corresponds to character 13 sensu [48]: Distinct anterior expansion of the alveolar arm of the premaxilla: (0) absent; (1) present.

(6) Corresponds to character 14 sensu [48]: Posterior indentation of the alveolar arm of the premaxilla: (0) absent; (1) present.

(7) Corresponds to character character 1 sensu [50]: Posterior border of distal portion of premaxilla: (0) indented; (1) straight.

(8) Corresponds to character 15 sensu [48]: Shape of the posterior tip of the ascending process of the premaxilla: (0) not curved medially; (1) curved medially.

(9) Corresponds to character 16 sensu [48]: Size of the premaxillary ascending process: (0) long; (1) shortened.

(10) Corresponds to character 17 sensu [48]: Shape of the premaxilla: (0) robust; (1) elongate.

(11) Corresponds to character 18 sensu [48]: Shape of the premaxillary ascending process: (0) not widened; (1) widened.

(12) Corresponds to character 19 sensu [48]: Shape of the dentary: (0) slender; (1) deep.

(13) Corresponds to character 20 sensu [48]: Reduction of the posteroventral process of the dentary: (0) not reduced; (1) reduced.

(14) Corresponds to character 21 sensu [48]: Shape of the retroarticular and ventral process of the anguloarticular: (0) short; (1) long.

(15) Corresponds to character 6 sensu [50]: Ventral process of the anguloarticular: (0) short; (1) median; (2) expanded.

(16) Corresponds to character 23 sensu [48]: Reduction of the coronoid process of the anguloarticular: (0) not reduced; (1) reduced.

(17) Corresponds to character 5 sensu [50]: Coronoid process of dentary: (0) broad; (1) narrow.

(18) Corresponds to character 24 sensu [48]: Pronounced indentation on the anterior base of the coronoid process of the anguloarticular: (0) absent; (1) present.

(19) Corresponds to character 25 sensu [48]: Shape of the teeth: (0) conical; (1) tricuspid.

(20) Corresponds to character 27 sensu [48]: Position and shape of the head of the autopalatinum: (0) slightly curved anteriorly, continuous with the main longitudinal axis of the bone; (1) bent anteriorly, displaced laterally relative to the main axis of the bone.

(21) Corresponds to character 28 sensu [48]: Extent of the ventral portion of the autopalatinum: (0) long, overlapping upper portion of quadratum; (1) short, not contacting quadratum.

(22) Corresponds to character 11 sensu [50]: Angle between anterior border and main axis of posterior process of quadratum: (0) 90-130; (1) 140-160.

(23) Corresponds to character 32 sensu [48]: Reduction of the dorsal edge of the mesopterygoid: (0) expanded to the infraorbital region; (1) reduced to a thin laminar bone.

(24) Corresponds to character 10 sensu [50]: Mesopterygoid: (0) large posterior tip close to or contacting metapterygoid; (1) small, posterior tip not contacting metapterygoid.

(25) Corresponds to character 33 sensu [48]: Concavity on the posterior margin of the quadratum: (0) absent; (1) present.

(26) Corresponds to character 34 sensu [48]: Metapterygoid: (0) present; (1) absent.

(27) Corresponds to character 36 sensu [48]: Reduction of the preoperculum: (0) preoperculum robust, L-shaped, with expanded anterior rim and well-developed sensory canal; (1) preoperculum thin, C-shaped, with reduced median rim and vestigial sensory canal.

(28) Corresponds to character 66 sensu [48] and character 30 sensu [50]: Shape of the neurocranium: (0) not flattened; (1) flattened.

(29) Corresponds to character 29 sensu [50]: Lateral rim of frontal: (0) well developed and well defined; (1) shortened and ill-defined.

(30) Corresponds to character 31 sensu [50]: Lacrimal: (0) flat, posterior rim wide; (1) slightly twisted, posterior rim reduced, bone formed mainly by canal; (2) very twisted and narrow, slender, canal vestigial.

(31) Corresponds to character 74 sensu [48]: Pectoral fin insertion: (0) lateral; (1) ventrolateral.

(32) Corresponds to character 52 sensu [50]: Pectoral radials: (0) robust, cubical; (1) thin, scale-like.

(33) Corresponds to character 53 sensu [50]: Fourth pectoral radial: (0) not expanded ventrally; (1) expanded ventrally.

(34) Corresponds to character 75 sensu [48]: Shape of the supracleithrum: (0) narrow to slightly broad ventrally; (1) extremely broad ventrally.

(35) Corresponds to character 47 sensu [50]: Supracleithrum: (0) short, about half longitudinal length of posttemporal; (1) long, approximately as long as posttemporal.

(36) Corresponds to character 76 sensu [48]: Fusion of the supracleithrum with the posttemporal: (0) absent; (1) present.

(37) Corresponds to character 79 sensu [48]: Notch on the posterior border of the cleithrum: (0) absent; (1) present.

(38) Corresponds to character 80 sensu [48]: Concavity on the posterior region of the coracoid: (0) moderate; (1) deep.

(39) Corresponds to character 83 sensu [48]: Medial process of the pelvic girdle: (0) long; (1) short.

(40) Corresponds to character 54 sensu [50]: Pelvic fin rays: (0) five or six; (1) seven or eight.

(41) Corresponds to character 84 sensu [48]: Anteromedial process of the pelvic girdle: (0) present; (1) absent.

(42) Corresponds to character 85 sensu [48]: Placement of the pelvic fins: (0) usually between the pleural ribs of vertebrae eight and 11, sometimes six and eight; (1) between the pleural ribs of vertebrae three and six.

(43) Corresponds to character 86 sensu [48]: Symmetry of the caudal fin skeleton: (0) asymmetrical; (1) symmetrical.

(44) Corresponds to character 87 sensu [48]: Shape of the caudal fin: (0) forked; (1) usually truncate or rounded.

(45) Corresponds to character 3 sensu [1]: Caudal fin rays, zone between upper and lower hypural plates, arrangement: (0) separated by broad interspace; (1) continuously arranged.

(46) Corresponds to character 7 sensu [1]: Upper hypurals and terminal centrum, degree of fusion: (0) attached, limited by cartilage edge; (1) complete ankylosis.

(47) Corresponds to character 8 sensu [1]: Upper and lower hypural plates, degree of fusion: (0) unfused; (1) partially fused (anterior portion unfused, posterior portion fused); (2) completely fused.

(48) Corresponds to character 18 sensu [1]: Hypurals 4 and 5, degree of fusion: (0) unfused; (1) fused.

(49) Corresponds to character 20 sensu [1]: Terminal centrum, central portion of side, keel-shaped process: (0) absent; (1) present.

(50) Corresponds to character 5 sensu [1]: Stegural development: (0) well-developed; (1) minute.

(51) Corresponds to character 10 sensu [1]: Stegural, ventral portion, lateral process: (0) absent; (1) present.

(52) Corresponds to character 12 sensu [1]: Parhypural, proximal part, relative position to terminal centrum: (0) overlapped; (1) not overlapped.

(53) Corresponds to character 1 sensu [1]: Epurals, number: (0) three or two; (1) one.

(54) Corresponds to character 2 sensu [1]: Epural, shape: (0) rod-like; (1) blade-like.

(55) Corresponds to character 21 sensu [1]: Epural, proximal region, width relative to distal region: (0) wider to slightly narrower; (1) conspicuously narrower.

(56) Corresponds to character 44 sensu [50]: First dorsal-fin ray: (0) single long first ray attached to two proximal radials; (1) long fin ray attached to two proximal radials, preceded by one or two short fin rays.

(57) Corresponds to character 45 sensu [50]: Medial anal radials: (0) ossified; (1) cartilaginous to weakly ossified.

(58) Corresponds to character 95 sensu [48]: Position of the first pleural rib: (0) on the third vertebra; (1) on the second vertebra.

(59) Corresponds to character 18 sensu [55]: Epipleural ribs: (0) rod-like; (1) distally widening, often bifid.

(60) Corresponds to character 35 sensu [50]: Neural prezygapophyses of first vertebra: (0) present; (1) absent.

(61) Corresponds to character 34 sensu [50]: Neural spine on first vertebra: (0) absent; (1) narrow; (2) broad, anteriorly expanded.

(62) Corresponds to character 36 sensu [50]: Neural prezygapophyses of caudal vertebrae: (0) moderate; (1) vestigial or absent; (2) long.

(63) Corresponds to character 42 sensu [50] and character 6 sensu [1]: Preural vertebra 2, neural spine, width relative to neural spines of preural vertebrae 4 and 5: (0) about equal; (1) wider.

(64) Corresponds to character 4 sensu [1]: Preural vertebra 2, neural spine: (0) absent; (1) well-developed, distal tip acting in support of caudal fin rays.

(65) Corresponds to character 14 sensu [1]: Preural vertebra 2, haemal spine, sub-basal region, deep constriction: (0) absent; (1) present.

(66) Corresponds to character 15 sensu [1]: Preural vertebra 2, neural spine, sub-basal region, deep constriction: (0) absent; (1) present.

(67) Corresponds to character 13 sensu [13]: Caudal skeleton preural vertebrae, number: (0) 4–5; (1) 6.

(68) Corresponds to character 164 sensu [49]: Association between the branchiostegal and opercular membranes: (0) not united; (1) united.

(69) Corresponds to character 59 sensu [50]: Orbital rim: (0) free; (1) attached ventrally; (2) completely attached.

(70) Corresponds to character 105 sensu [48]: Number of radii on the anterior abdominal scales: (0) four to six; (1) 12 to 16; (2) 20 to 25.

(71) Corresponds to character 64 sensu [50]: Squamation between eye and supraorbital series of neuromasts: (0) densely scaled; (1) one to three small scales or naked.

(72) Corresponds to character 65 sensu [50]: Caudal fin squamation: (0) body squamation extending on caudal fin through minute, vertically elongated scales; (1) body squamation abruptly interrupted on caudal fin base.
