# Supplementary material for: †Kenyaichthyidae fam. nov. and †Kenyaichthys gen. nov. – First Record of a Fossil Aplocheiloid Killifish (Teleostei, Cyprinodontiformes)
Source: PLoS One. 2015 Apr 29;10(4):e0123056. doi: 10.1371/journal.pone.0123056 (PMC4414574; doi:10.1371/journal.pone.0123056)
Supplement: S10 Table — (DOC) [file pone.0123056.s010.doc]

**S10 Table. Character-taxon matrix used in the phylogenetic analysis shown in Figs. 14 and 15 based on 72 characters of** 13 terminal taxa and two outgroups.

| Taxon | | 1–5 | | 6–10 | | 11–15 | | 16–20 | | 21–25 | | 26–30 | | 31–35 | | 36–40 | | 41–45 | | 46–50 | | 51–55 | | 56–60 |
| --- | --- | --- | --- | --- | --- | --- | --- | --- | --- | --- | --- | --- | --- | --- | --- | --- | --- | --- | --- | --- | --- | --- | --- | --- |
| Atheriniformes | | 00000 | | 00000 | | 00000 | | 00000 | | 00000 | | 00000 | | 00000 | | 00000 | | 00000 | | 00000 | | 00000 | | 00000 |
| Belonoidei | | 00000 | | 00000 | | 00000 | | 00000 | | 00000 | | 00000 | | 00000 | | 00000 | | 00000 | | 0000A | | 00000 | | 00000 |
| Aplocheilidae | | 10001 | | 00100 | | 00002 | | 10000 | | 00100 | | 00100 | | A0000 | | 10010 | | 10111 | | 1DA01 | | 00110 | | 00100 |
| Nothobranchiidae | | 10001 | | 00100 | | 00002 | | 10000 | | 00100 | | 00101 | | 10000 | | 10010 | | 10111 | | 1DA11 | | 01110 | | 10110 |
| Rivulidae | | 10001 | | 01000 | | 0000C | | 11000 | | 1A1A0 | | 01112 | | 1AA0A | | 0001A | | 10111 | | 1D101 | | 0111A | | 1A1AA |
| Fundulidae | | 2?001 | | 10001 | | 01000 | | 00001 | | 00100 | | 10000 | | 10000 | | 00100 | | 10111 | | 12101 | | 1A110 | | 00100 |
| Profundulidae | | 2?001 | | 10000 | | 01000 | | 00001 | | 00101 | | 10000 | | 10010 | | 01000 | | 10111 | | 1B101 | | 10110 | | 00100 |
| Goodeidae | | 2?001 | | 100A0 | | 01000 | | 00001 | | 00101 | | 10000 | | 10010 | | 01000 | | 10111 | | 12101 | | 1A110 | | 00100 |
| Valenciidae | | 00001 | | 10000 | | 01000 | | 00001 | | 00100 | | 10000 | | 10000 | | 00000 | | 10111 | | 12101 | | 10110 | | 00100 |
| Cyprinodontidae | | 01101 | | 10010 | | 01000 | | 00001 | | 00100 | | 10000 | | 10000 | | 00000 | | 11111 | | 12101 | | A1110 | | 00100 |
| Anablepidae | | 01011 | | 10010 | | 11110 | | 00011 | | 00100 | | 10000 | | 10000 | | 00000 | | 10111 | | 1B101 | | 10110 | | 00100 |
| Poecilidae | | 01011 | | 10010 | | 11110 | | 00101 | | 00100 | | 10000 | | 10000 | | 00000 | | 11111 | | 1C101 | | 10110 | | 00100 |
| †*Kenyaichthys* | 10001 | | 01000 | | 00002 | | 1000A | | 0010A | | 0010B | | 100?? | | ?001A | | 10111 | | 12101 | | 0A1?0 | | 10100 | |

S10 Table. (Continued)

| Taxon | 61–65 | 66–70 | 71–72 |
| --- | --- | --- | --- |
| Atheriniformes | 00000 | 00000 | 00 |
| Belonoidei | 000A0 | 00000 | 00 |
| Aplocheilidae | 00110 | 00012 | 00 |
| Nothobranchiidae | 20110 | 00022 | 00 |
| Rivulidae | BC110 | 00122 | AA |
| Fundulidae | 00110 | 0A001 | 00 |
| Profundulidae | 00110 | 10001 | 00 |
| Goodeidae | 00110 | 00001 | 00 |
| Valenciidae | 00110 | 00001 | 00 |
| Cyprinodontidae | 0011A | AA001 | 00 |
| Anablepidae | 00110 | 1A001 | 00 |
| Poecilidae | 00110 | 10001 | 00 |
| †*Kenyaichthys* | 10A10 | 0A0?? | ?1 |

Character states are as follows: 0, plesiomorph; 1, apomorph; 2, apomorph. Polymorph characters are coded as follows: A, (0,1), B (1,2), C (0,1,2), D (0,2). ? indicates missing or inapplicable character. All characters were treated as unordered.
