# Supplementary figures and images for: †Kenyaichthyidae fam. nov. and †Kenyaichthys gen. nov. – First Record of a Fossil Aplocheiloid Killifish (Teleostei, Cyprinodontiformes)
Source: PLoS One. 2015 Apr 29;10(4):e0123056. doi: 10.1371/journal.pone.0123056 (PMC4414574; doi:10.1371/journal.pone.0123056)

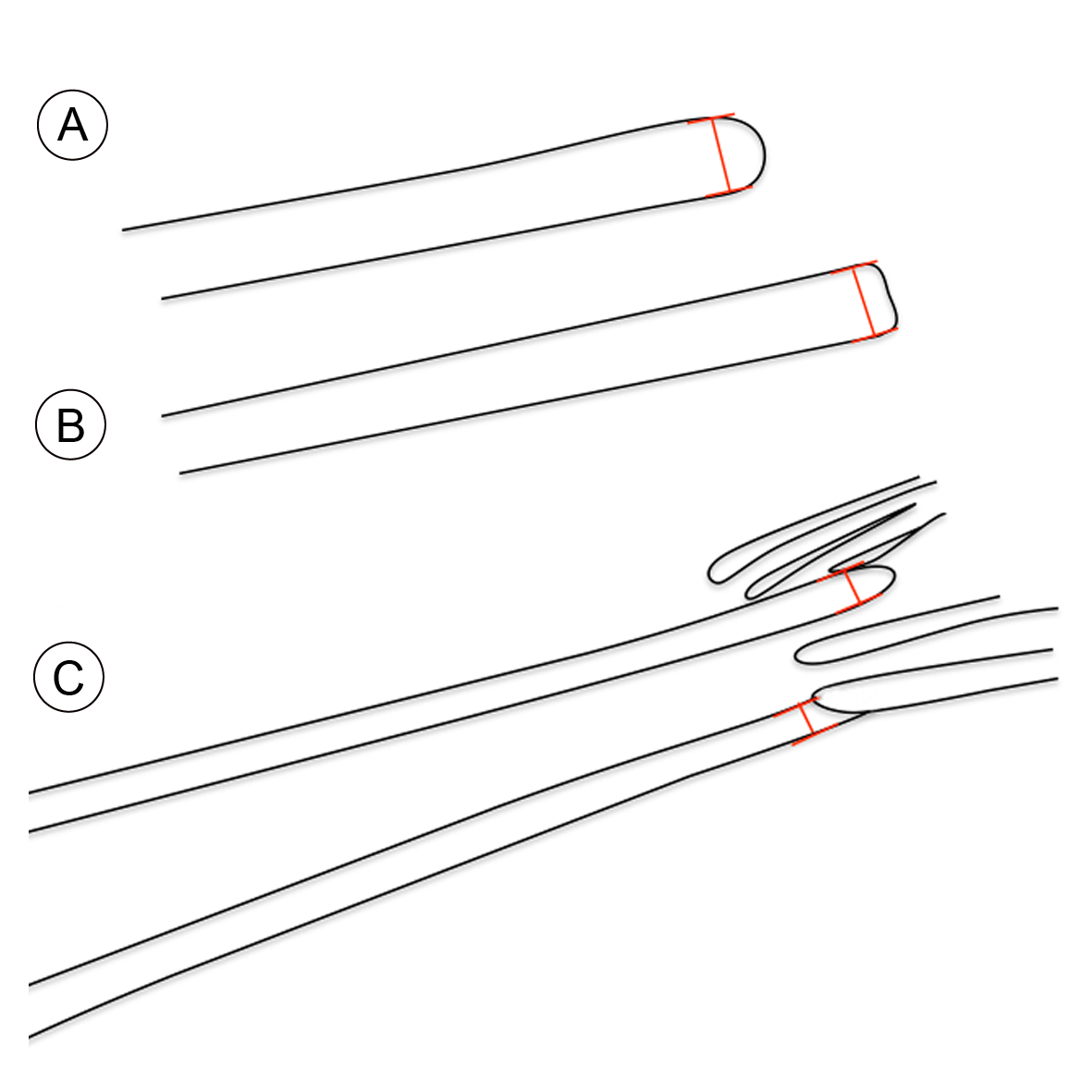

Supplement: S1 Fig — A on a rounded tip; B on a blunt tip; C on spines with tip not covered by caudal fin rays and on spine covered by fin rays. (TIF) [file pone.0123056.s012.tif]
